# Evaluating efficacy and mechanism of traditional Chinese medicine in diabetes treatment: a meta-analysis and network pharmacology study

**DOI:** 10.3389/fendo.2025.1605091

**Published:** 2025-10-07

**Authors:** Shuai Tang, Jie Lin, Gangyi Li, Huaijuan Guo, Chang Liu, Fuju Wu

**Affiliations:** ^1^ Department of Pathology, Deyang People’s Hospital, Deyang, China; ^2^ Department of Hepatobiliary and Pancreatic Surgery, The Second Hospital of Jilin University, Changchun, China; ^3^ Department of Ophthalmology, First People’s Hospital of Zigong, Zigong, Sichuan, China; ^4^ Department of Oncology, the Affiliated Hospital of Yangzhou University, Yangzhou University, Yangzhou, China; ^5^ Department of Obstetrics and Gynecology, The Second Hospital of Jilin University, Changchun, China

**Keywords:** diabetes, traditional Chinese medicine, meta-analysis, network pharmacology analysis, efficacy

## Abstract

**Background:**

Diabetes is a prevalent chronic metabolic disorder, and the rising rates of this condition, along with its complications, significantly threaten public health. Traditional treatments for diabetes have certain limitations in practical applications, and it is particularly important to find new, effective treatments with fewer side effects. With a long history and rich experience, traditional Chinese medicine (TCM) effectively treats diabetes.

**Methods:**

Data from randomized controlled trials concerning TCM and its effects on diabetes were gathered and analyzed from various databases. A meta-analysis was conducted on the 58 selected articles, and the potential mechanisms of action of the active ingredients in TCM were examined using network pharmacology techniques.

**Results:**

Meta-analysis of 58 randomized trials (n=7,318) demonstrated significant improvements in fasting glucose (MD=-0.53 mmol/L [-0.67,-0.39], P<0.00001), HbA1c (MD=-0.40% [-0.61,-0.20], P = 0.0001), and insulin resistance (HOMA-IR: MD=-0.90 [-1.51,-0.29], P = 0.004), alongside favorable lipid modulation (LDL: MD=-0.14 mmol/L, P = 0.0002). Network pharmacology revealed six core herbs (*Astragalus membranaceus*, *Coptis chinensis*, etc.) targeting 32 hub genes (AKT1, IL1B, PPARG, etc.) through three key pathways: insulin signaling (PI3K-AKT), inflammatory regulation (TNF/IL-17), and oxidative stress response (HIF-1/NRF2 axis). The polypharmacological effects were mediated by multi-component interactions involving quercetin, kaempferol, and stigmasterol.

**Conclusion:**

TCM has demonstrated considerable effectiveness in managing diabetes. Through meta-analysis and network pharmacology research, this translational study establishes Level 1a evidence for TCM’s antidiabetic efficacy while decoding its systems-level mechanisms. The integrated methodology provides a paradigm for evaluating complex herbal interventions in metabolic disorders.

**Systematic Review Registration:**

https://www.crd.york.ac.uk/PROSPERO, identifier CRD42024572433.

## 1 Introduction

Diabetes, a chronic metabolic disease prevalent worldwide, is marked by persistently elevated blood glucose levels. This condition results from either inadequate insulin secretion or diminished insulin sensitivity. As per the International Diabetes Federation (IDF), the global adult population affected by diabetes exceeded 536.6 million in 2021, and this figure is expected to rise in the future (1). Diabetes has profound effects on patients’ quality of life and can lead to several complications, such as cardiovascular disease, nephropathy, retinopathy, and neuropathy. These complications present significant challenges to public health (2).

Currently, traditional diabetes treatment strategies primarily include lifestyle interventions (dietary control, exercise therapy) (3) and pharmacological interventions (oral hypoglycemic agents such as metformin, sulfonylureas, thiazolidinediones, SGLT2 inhibitors, GLP-1 RAs, DPP-4 inhibitors, as well as insulin injections, etc.) (4). Although these methods have achieved certain efficacy in glycemic control, they still face numerous challenges and limitations in clinical application. Long-term medication may induce a series of side effects, such as hypoglycemia risk (especially with sulfonylureas and insulin therapy), weight gain, gastrointestinal discomfort (metformin, GLP-1 receptor agonists), genitourinary tract infections (SGLT2 inhibitors), and potential hepatorenal toxicity (5–9). Furthermore, poor long-term treatment adherence is also a widespread issue; complex medication regimens, frequency of drug administration, frequent blood glucose monitoring, and the discomfort associated with injection therapy all impact treatment effectiveness (10–13). More critically, diabetes and its complications impose a substantial economic burden on patients and society, including high drug costs, frequent medical visits, hospitalization expenses, and disability and reduced work capacity due to complications (14). These challenges and limitations in clinical practice underscore the urgent need for effective, safe, economical, and patient-friendly alternative or complementary therapies.

Against this backdrop, traditional Chinese medicine (TCM), with its millennia-long history and unique theoretical framework, offers valuable insights and perspectives for diabetes management. Accumulating clinical and preclinical evidence, particularly in recent years, continues to highlight the potential advantages of TCM interventions in diabetes care. Firstly, unlike single-target Western drugs, TCM formulas—typically comprising multiple herbs—exert synergistic effects on multiple pathways involved in glucose metabolism, insulin resistance, beta-cell function, inflammation, and oxidative stress. This multi-target action aligns with TCM’s holistic philosophy and may address diabetes’ complex pathophysiology more comprehensively (15, 16). Critically, modern phytochemical research has identified a plethora of bioactive metabolites isolated from TCM herbs that underpin these therapeutic effects. Key compounds such as berberine (from *Coptis chinensis*), astragaloside IV (from *Astragalus membranaceus*), ginsenosides (from *Panax ginseng*), and polyphenols (e.g., from *Quinoa*) have demonstrated significant anti-diabetic properties in mechanistic studies. These include enhancing insulin sensitivity, promoting β-cell regeneration, and suppressing inflammatory cascades (17–19). This scientific validation of active constituents provides a molecular basis for TCM’s efficacy and bridges traditional knowledge with modern pharmacology. Secondly, TCM employs individualized treatment through Syndrome Differentiation and Treatment (A core principle of TCM that involves identifying syndromes based on clinical manifestations and formulating corresponding therapies). Diagnosis classifies diabetic patients into distinct patterns (e.g., Yin Deficiency with Dryness-Heat, Qi and Yin Deficiency, Spleen Deficiency with Dampness), enabling customized herbal prescriptions, potentially yielding better personalized outcomes (20–22). Thirdly, TCM demonstrates potential for reducing complications. Specific herbs and formulas show protective effects against diabetic nephropathy, retinopathy, and neuropathy in preclinical and clinical studies, primarily through anti-inflammatory, antioxidant, and microcirculation-improving mechanisms (23–25).Finally, systematic reviews and meta-analyses indicate TCM interventions, when properly administered, exhibit a relatively favorable safety profile. They are associated with lower incidence of adverse events—particularly severe hypoglycemia and gastrointestinal issues—compared to conventional hypoglycemic agents. This suggests suitability for long-term management or adjunctive therapy (26, 27).

However, despite its promising prospects, the application of TCM in diabetes remains challenging. Clinical evidence remains heterogeneous due to differences in study design (e.g., sample size, duration of treatment, and control settings), TCM formulations (standardization, batch variability), and populations (ethnicity, region, and syndrome type). Furthermore, the complex multi-component and multi-target nature of TCM poses significant challenges to elucidating its precise mechanisms of action using traditional single-target approaches, which, to a certain extent, limits its wider understanding and acceptance. In order to more systematically evaluate clinical efficacy, overcome the limitations of individual studies, and deeply explore its complex mechanism of action, this study employs an integrated strategy combining meta-analysis and network pharmacology. This methodological choice is critical: Meta-analysis provides a rigorous quantitative synthesis of existing randomized controlled trial (RCT) data to derive more precise and generalizable estimates of TCM’s overall clinical efficacy and safety profile in DM management (27, 28), overcoming the limitations of individual studies and establishing robust clinical evidence. Meanwhile, network pharmacology offers a powerful systems biology framework to systematically predict and analyze the interactions between bioactive TCM components, their potential targets, and the associated biological pathways and networks involved in DM pathogenesis (29, 30), uniquely suited to decipher the complex, multi-target mechanisms underlying TCM’s therapeutic effects.

Therefore, the objective of this study is to comprehensively assess the effectiveness and safety of TCM for treating diabetes mellitus, and to investigate its mechanism of action through the integration of meta-analysis and network pharmacology. We will analyze the advantages and shortcomings of TCM in diabetes management by reviewing relevant literature and experimental studies, providing scientific basis and new ideas for comprehensive treatment of diabetes, and promoting the application and development of TCM in modern medicine.

## 2 Materials and methods

### 2.1 Meta-analysis

#### 2.1.1 Literature search strategy

In line with the Preferred Reporting Items for Systematic Reviews and Meta-Analyses (PRISMA) guidelines, the meta-analysis ensured methodological rigor and high-quality reporting, enhancing the study’s reliability and transparency. Electronic and manual literature searches were conducted independently by two authors in PubMed, Embase, and Cochrane Library databases for reports published up to December 15, 2024, with no language restrictions. The comprehensive search strategy is outlined in the **Supplementary Material**. The protocol for this review was registered with PROSPERO (CRD42024572433).

#### 2.1.2 Criteria of eligibility

The criteria for inclusion and exclusion are detailed in **Table 1**.

**Table 1:** Article inclusion and exclusion criteria.

PICOS	Inclusion criteria	Exclusion criteria
Participants	1. Age 18 years or older	1. Younger than 18 years
	2. The diagnostic criteria for type 2 diabetes were in accordance with the WHO (1985, 1999 or 1998, 2010) or ADA (1997 or 1996, 2007, 2009) diagnostic criteria	2. Patients with concurrent serious primary diseases, including cardiovascular, renal, hematopoietic, immune or psychiatric diseases
	3. The patient's vital signs are stable and compliance is good.	3. Women who are breastfeeding, pregnant, or taking birth control pills
Intervention	The intervention group received Chinese medicine treatment, including oral Chinese medicine decoctions and Chinese patent medicines [oral or (and) external application treatment for diabetic foot patients], with no limit on dosage and frequency	The intervention group was treated with acupuncture, tuina, or acupoint application and other external therapies of Chinese medicine
Comparison	The control group received conventional treatment (including diabetes education, proper diet, regular exercise, and blood sugar reduction. Symptomatic treatment such as blood pressure reduction and lipid regulation can be given according to specific circumstances)	The control group was treated with TCM treatment
Outcome	FBG, PBG, HbA1c, FI, HOMA-IR, Proteinuria/24h, UAER, SCr, BUN, TG, TC, LDL, HDL, Ulcer Area of diabetes foot, Vascular Endothelial Growth Factor in patients with diabetes foot	Incomplete or unidentified data
Study design	Randomized controlled trial (RCT)	Non-RCTs
Others	None	Duplicate publications, abstracts, reviews, case reports, and letters

Fasting Blood Glucose (FBG), Postprandial Plasma Glucose (PBG), Glycated Hemoglobin (HbA1c), Fasting Insulin (FI), Homeostatic Model Assessment of Insulin Resistance (HOMA-IR), Urinary Albumin Excretion Rate (UAER), Serum Creatinine (SCr), Blood Urea Nitrogen (BUN), Triglyceride (TG), total cholesterol (TC), Low-density Lipoprotein (LDL), High-density Lipoprotein (HDL), Traditional Chinese medicine (TCM).

#### 2.1.3 Study selection and data extraction

In alignment with PRISMA guidelines, the literature search and screening processes were conducted independently by two researchers using the predefined inclusion and exclusion criteria. For doubtful pilot studies, which could not be determined after full discussion, the corresponding author ruled on inclusion. Extracted information for inclusion in the study included: first author, year of publication, sample content, mean age or age range, commonly used treatments (protocols), TCM interventions, control interventions, duration, and outcome indicators.

#### 2.1.4 Risk of methodological bias assessment

To assess the methodological rigor of the selected literature, two investigators independently appraised it using the Cochrane Handbook for Evaluating Randomized Controlled Trials (version 5.1.0). Results were cross-verified to ensure consistency. The manual covers randomization of sequence generation (selection bias), allocation concealment (selection bias), blinding of participants and personal (performance bias), blinding of outcome assessment (detection bias), incomplete outcome data (attrition bias), selective reporting (reporting bias), and other biases. To complete this section, we utilized the risk assessment tool in Review Manager 5.3 software. Disagreements in risk evaluations were resolved through discussion or, if necessary, consultation with an impartial third party.

#### 2.1.5 Statistical analysis

Statistical analysis of the data was performed using RevMan 5.3 software for meta-analysis and STATA SE18.0 for comprehensive statistical evaluation. This included data summarization and the creation of forest plots. Continuous variables were represented as mean difference (MD), and the effect size indicator was presented with a 95% confidence interval (CI). If significant heterogeneity was detected between the groups (p < 0.1 or I² > 50%), either a subgroup analysis or sensitivity analysis was performed to identify and address potential sources of heterogeneity. In cases where heterogeneity persisted despite clinical homogeneity, a random effects model was employed; otherwise, a fixed effects model was utilized. Sensitivity analysis, conducted using STATA SE18.0, assessed the robustness of the included studies against various methodological biases. Additionally, publication bias was evaluated through Begg’s test (31) and Egger’s test (32), using STATA SE18.0 software.

### 2.2 Network pharmacology

#### 2.2.1 Network pharmacology study of effective TCM components for diabetes

TCM prescriptions identified from the meta-analyses were organized according to their frequency of use. Herbs that appeared more than seven times were selected as primary research targets, including Huangqi (*Astragalus mongholicus* Bunge), Fuling (*Wolfiporia* cocos (*F.A. Wolf*) Ryvarden & Gilb.), Shanzhuyu (*Cornus officinalis* Siebold & Zucc.), Huanglian (*Coptis chinensis* Franch.), Zexie (*Alisma gramineum* Lej.), and Dangshen (*Codonopsis pilosula* Nannf.). The bioactive compounds of the herbal medicines identified from the meta-analysis were retrieved from the Traditional Chinese Medicine Systems Pharmacology Database and Analysis Platform (TCMSP, http://www.tcmsp-e.com/) (33). For each herb, all compounds listed in TCMSP were collected. Compounds were then screened according to the commonly applied pharmacokinetic parameters: oral bioavailability (OB) ≥ 30% and drug-likeness (DL) ≥ 0.18. These criteria are recommended in TCMSP to select compounds with favorable absorption and drug-like properties. The herb–compound relationships were directly obtained from the TCMSP records, ensuring that each bioactive compound was accurately linked to its source herb. After screening, compounds present in multiple herbs and/or with high degree values in the subsequent network analysis, such as quercetin, kaempferol and stigmasterol, were identified as representative constituents for further network pharmacology analysis. Human gene information was then obtained from The Universal Protein Database (UniProt, https://www.uniprot.org/) (34) to annotate the target sites of these bioactive components.

#### 2.2.2 Identifying disease targets for diabetes

Our team employed the keyword “diabetes” to identify disease targets related to diabetes across several databases, including DisGeNet (https://www.disgenet.org/), GeneCards (https://www.genecards.org/), OMIM (http://omim.org/), TTD (http://db.idrblab.net/ttd/), and CTD (https://ctdbase.org/).

#### 2.2.3 Acquisition of TCM-diabetes intersection genes and construction of protein interaction network

The Venn package was utilized to identify intersecting genes between TCM components and diabetes. These intersecting genes were subsequently imported into the STRING database (https://stringdb.org/), with the selection criteria set to humans as the genus group and a confidence score > 0.4, while excluding independent protein molecules. The resulting data were then imported into Cytoscape 3.8.2 (35) to construct a molecular network diagram illustrating the interactions between TCM components and the identified intersecting genes.

#### 2.2.4 TCM-Determination of core genes in diabetes

Hub genes were identified using the cytoHubba plug-in within Cytoscape software. To evaluate and select the core genes, six commonly used algorithms were applied: MNC (Maximum Neighborhood Component), Degree, Closeness, Radiality, Stress, and EPC (Edge Percolated Component).

#### 2.2.5 GO and KEGG pathway enrichment analysis

To elucidate the enriched pathways associated with drug targets and diabetes-related genes, we performed functional enrichment analysis on significant gene clusters. This included Gene Ontology (GO) analysis, which covered Biological Processes (BP), Cellular Components (CC), and Molecular Functions (MF). Additionally, KEGG pathway analysis and Disease Ontology (DO) analysis was conducted. The analyses were carried out using several R packages, including clusterProfiler, org.Hs.eg.db, enrichplot, circlize, RColorBrewer, and ComplexHeatmap. Filtering criteria were applied with p-value < 0.05 and q-value < 0.05.

## 3 Results

### 3.1 Meta-analysis

#### 3.1.1 Identification and selection

**Figure 1:**
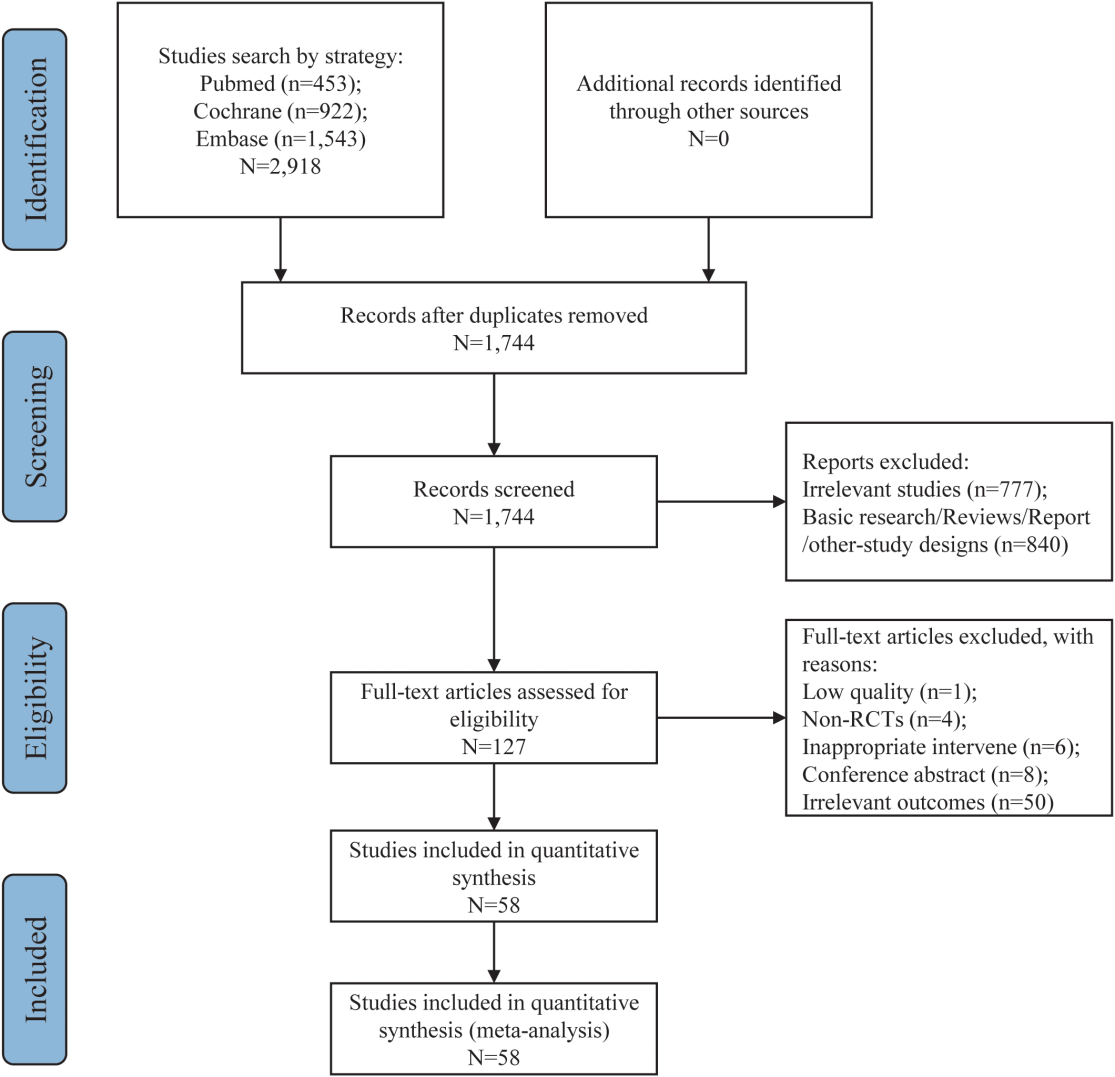
Flow chart of this study.

From an initial pool of 2,918 documents, 1,744 candidate articles were identified following the removal of 1,174 duplicates. After reviewing titles and abstracts, 1,617 irrelevant papers were excluded. The remaining 127 articles were further evaluated in full text according to the predefined inclusion and exclusion criteria. This process led to the selection of 58 articles for inclusion in the meta-analysis (36–93). The study selection flowchart is illustrated in **Figure 1**, and **Table 2** provides a summary of the key characteristics of these 58 articles.

**Table 2:** Characteristics of the 14 studies included in the meta-study.

Author	Year	Sample size		Intervention measurement		Age		Therapy time	Main outcome
		T	C	T	C	T	C		
Asadi, S. (36)	2019	40	40	Nano curcumin capsules	Placebo	53.3±6.5	54.6±6.2	8 weeks	①②
Ataabadi, G. (37)	2019	28	28	Otostegia persica	Placebo	60.6±7.03	59.07±8.6	12 weeks	①④⑥⑦
Cao, W. H. (38)	2005	36	36	Fengbei Huayu Recipe	No additional Tx	52.2±5.2	56.1±5.7	8 weeks	③④⑤⑫
Chan, S. W. (39)	2020	20	20	Bilberry	Placebo	54.9±11.7	56.6±7.5	4 weeks	①③④⑥⑦
Chen, H. W. (40)	2006	40	40	Ligusticum chuanxiong	No additional Tx	54.8±11.6	53.6±12.7	4 weeks	①④⑤⑦⑬⑭
Chen, Y. B. (41)	1995	34	34	Bushenhuoxue Tablet	No additional Tx	54.19±8.45		8 weeks	①④⑤⑧⑧
Cho, Y. Y. (42)	2012	33	33	Artemisia princeps Pampanini	Placebo	53.74±3.37	55.15±2.84	9 weeks	①④⑤⑧⑧
Ebrahimi, F. (43)	2019	40	40	saffron	Placebo	55.2±7.3	53±10.6	12 weeks	⑭
Fan, W. (44)	2022	44	42	enriching pus for tissue growth(EPTG)	Nano silver antibacterial dressing for medical purposes	70.95±7.70	71.12±6.52	12 weeks	⑩⑪
Fang, Z. (45)	2023	44	46	Danzhi Jiangtang capsule + lifestyle intervention therapy	lifestyle intervention therapy	49.21±7.41	49.17±7.63	12 weeks	①②③⑤
Fang, Z. (46)	2014	223	216	Shenzhu Tiaopi granule + lifestyle intervention therapy	lifestyle intervention therapy	54.95±9.5	54.61±10.51	12 months	①②③④⑤⑥⑦
Ge, J. (47)	2015	54	54	TCM	No additional Tx	55.6±8.7	55.9±8.9	Not Mentioned	①②
Guo, D. Z. (48)	2008	39	37	safflower yellow pigment powder injection + Benazepril	Benazepril	47.3±12.6	46.9±13.2	30 days	⑫
Guo, Q. (49)	2016	47	49	Sancai powder	Metformin	52.0±9.7	53.7±9.4	12 weeks	①②③④⑤⑥⑦⑧
Guo, X. Y. (50)	2022	39	39	Jianpi Yishen formula + Candesartan ester tablets	Candesartan ester tablets	58.84±10.23	58.28±12.12	12 weeks	①⑬⑭
Guo, Z. A. (51)	2014	81	80	Qizhi Jiangtang Capsules	Valsartan capsules	52.38±9.67	50.62±9.34	24 weeks	⑮
Huang, Y. H. (52)	2019	23	23	YH1	Placebo	50±26.67	56±17.78	12 weeks	①②⑤⑥⑦⑧
Jiang, L. (53)	2023	16	15	Shenlian formula	Placebo	52.4±8.4	56.9±12.4	12 weeks	①②④⑥⑧⑧
Jin, S. Y. (54)	2021	53	51	Shenxie Zhitong Capsule	lipoic acid	64.36±7.08	62.23±7.32	12 weeks	①②④⑤⑧⑧
Jin, Y. H. (55)	2015	40	40	Sanhuang Jiedu Tongluo Decoction	No additional Tx	50.88±8.64	51.58±8.63	12 weeks	①②④⑤⑧⑧
Ke, B. (56)	2012	45	40	Modified Linggui Zhugan Decoction + Modified Linggui Zhugan Decoction	No additional Tx	46.5±7.3	45.7±7.5	6 months	①②③④⑤⑥⑦⑧
Li, B. Y. (57)	2015	22	23	Shenshuaining Granule + Telmisartan tablets	Telmisartan tablets	52.5±20.8	52±20.6	12 weeks	⑬
Li, J. P. (58)	2006	41	40	Tangshenling + Telmisartan tablets	Telmisartan tablets	50.3±16.7	51.2±17.2	8 weeks	①④⑤
Li, P. (59)	2015	56	26	Tangshen Formula	Placebo	58.88±8.96	60.81±9.91	24 weeks	④⑤⑥⑦⑫⑬⑭⑮
Li, X. S. (60)	2007	34	29	Extract of Gingko biloba	No additional Tx	66.9±7.3	68.2±7.7	8 weeks	①④⑤⑥⑦
Li, Y. S. (61)	2014	116	100	Compound fluid of Cortex Phellodendri	Kangfuxin liquid	57.12±11.65		4 weeks	⑩⑪
Li, Y. S. (62)	2016	540	180	Compound fluid of Cortex Phellodendri	Kangfuxin liquid	63.7±9.56	63.12±10.53	4 weeks	⑩⑪
Li, Z. Q. (63)	2013	58	58	San Xiao Decoction	No additional Tx	54.9±7.3	56.5±6.4	12 weeks	①②
Lian, F. (64)	2015	92	94	Jinlida Granule	Placebo	55.18±9.13	55.81±9.93	12 weeks	①②⑧
Liu, H. (65)	2015	50	16	‘Spleen-kidney-care’ Yiqi Huayu and Jiangzhuo decoction	No additional Tx	61±9	60±11	Not Mentioned	⑬
Liu, Y. H. (66)	2005	23	23	Milkvetch injection	Captopril	44.8±10.1	48.1±12.8	12 weeks	①②③④⑤⑫⑬⑭
Liu, Y. N. (67)	2016	50	50	Xiaoke Decoction	No additional Tx	52.3±3.4		4 weeks	①②④⑤
Liu, Z. Q. (68)	2001	86	50	Milkvetch injection	No additional Tx	41.0±6.7	40.0±5.9	3 weeks	⑫
Lu, T. (69)	2012	46	20	Cinnamon extract	Placebo	60.5±7.36	60±5.9	12 weeks	④⑤⑥⑦
Mehrzadi, S. (70)	2018	27	29	Boswellia serrata Gum Resin	Placebo	57.07±10.08	52.68±10.69	8 weeks	①④⑤⑥⑦⑧
Mirfeizi, M. (71)	2015	57	45	Cinnamon/Whortleberry	Placebo	53.58±11.51	54±12	90 days	①②④⑤⑥⑧⑧
Moein, S. (72)	2020	27	25	salvia mirzayanii	Placebo	53.37±7.68	55.4±8.48	12 weeks	④⑤⑥⑦⑧
Nematollahi, S. (73)	2022	25	25	berberine and fenugreek seed co-supplementation	Placebo	Not Mentioned	Not Mentioned	12 weeks	①④⑥⑦⑧
Ni, Q. (74)	2012	76	40	Qiyao Xiaoke Capsule	No additional Tx	48.2±10.1	45.8±10.5	3 months	①②③④⑤⑥⑦⑧
Pang, J. (75)	2023	39	38	modified Zuoguiwan + Perindopril tert-butylamine tablets	Perindopril tert-butylamine tablets	62±9.0	61±9.1	12 weeks	①②③
Park, K. (76)	2020	30	31	Korean Red ginseng	Placebo	59.3±8.79	59.7±7.22	24 weeks	①②③④⑤⑥⑦⑧
Shi, R. (77)	2019	266	260	Liuwei Dihuang Pills and Ginkgo Biloba Tablets	Placebo	60.45±6.19	60.81±6.36	24 months	①②③④⑦
Shi, Y. L. (78)	2016	32	29	Jinlida Granule	No additional Tx	47.1±7.1	49.9±7.2	12 weeks	①②③④⑤⑥⑦
Song, J. (79)	2009	30	30	Bailing Capsule + Benazepril	Benazepril	51.2±17.2	50±16.7	16 weeks	①⑫⑭⑮
Tong, X. L. (80)	2013	292	107	Tang Min Ling Wan	Placebo	54.4±7.7	54.5±7.6	12 weeks	①②
Vuksan, V. (81)	2008	25	20	Korean red ginseng	Placebo	18∼65		12 weeks	①③
Wainstein, J. (82)	2016	23	27	Purslane Extract	Placebo	52.4±7.9	58.3±10.8	12 weeks	①③④⑤⑥⑦⑧⑧
Wang, H. Y. (83)	2004	31	23	Compound Fructus Arctii mixture	losartan	60.30±10.45	58.22±14.11	12 weeks	①②④⑤⑥⑦
Wang, W. J. (84)	2015	31	22	Shenfu Yishen Capsule	No additional Tx	41.7±13.6	44.1±12.9	4 weeks	⑭
Wang, X. (85)	2015	50	52	Shenluoan Decoction + Irbesartan	Irbesartan	61.26±5.14	60.85±4.15	24 weeks	①④⑤⑬⑭
Wang, X. B. (86)	1997	32	25	Tangshenkang Capsule	No additional Tx	50.55±13.06	49.48±12.98	6 weeks	①
Wang, Y. (87)	2023	50	50	Sangzhi total alkaloid tablets	No additional Tx	53.11±9.69	52.64±10.53	16 weeks	①②④⑤⑥⑫⑬⑭
Wang, Y. H. (88)	2023	58	59	Buyang Huanwu Decoction combined with Shenqi Dihuang Decoction	No additional Tx	53.19±10.13	52.59±10.41	12 weeks	①②⑬
Wang, Y. Z. (89)	2007	54	54	TCM + Benazepril	Benazepril	67.8±7.1	67.7±7.5	12 weeks	①②④⑤⑧
Wang, Z. (90)	2013	30	30	Qingjie Tongluo prescription	No additional Tx	60.2±5.7	58.2±7.3	12 weeks	⑩
Xiong, C. (91)	2020	62	62	Tripterygium Wilfordii Hook F	Valsartane	50.3±11.8	49.6±12.3	24 weeks	①④⑤⑭⑮
Zhang, X.	2015	109	110	Shen Qi Formula	Insulin	57.1	56.9	12 weeks	①②③④⑤
Zhao, Y. (93)	2005	35	28	Tongxinluo capsule	No additional Tx	55.36±12.36	53.54±12.92	8 weeks	①

T: Experimental group, C: Control group, ① Fasting Plasma Glucose, ② Postprandial Plasma Glucose, ③ Glycated Hemoglobin, ④ Triglyceride, ⑤ Total Cholesterol, ⑥ Low-density Lipoprotein, ⑦ High-density Lipoprotein, ⑧ Homeostatic Model Assessment of Insulin Resistance, ⑧ Fasting Insulin, ⑩ Ulcer Area of diabetes foot, ⑪ Vascular Endothelial Growth Factor in patients, ⑫ Urinary Albumin Excretion Rate, ⑬ Blood Urea Nitrogen, ⑭ Serum Creatinine, ⑮ 24-hours Proteinuria.

#### 3.1.2 Assessment of risk of bias

**Figure 2:**
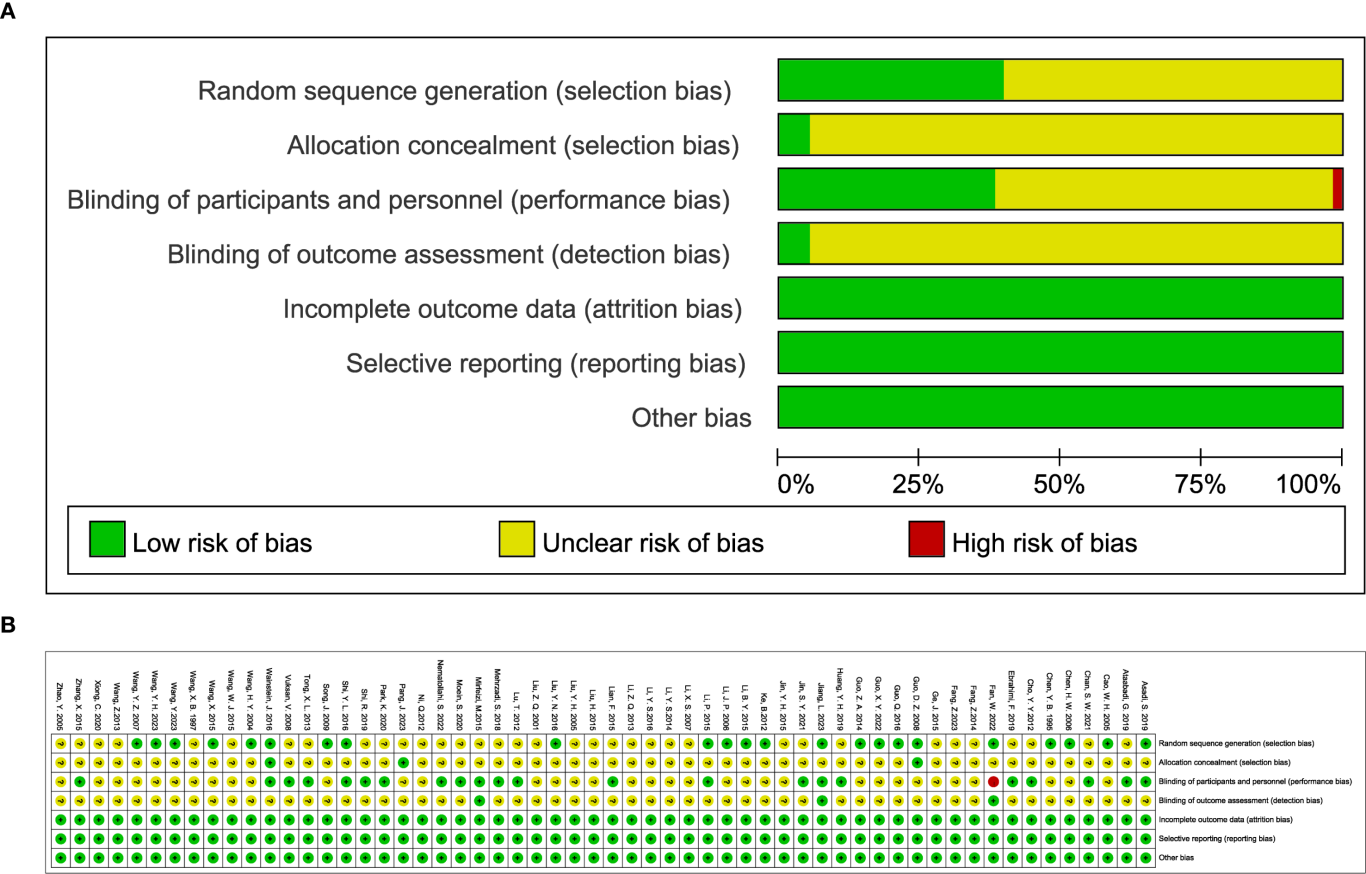
**(A)** classification of bias risk of included articles and **(B)** bias characteristics of each included article.

The results of the risk of bias assessment are detailed in **Figure 2**. All studies included in the analysis employed randomization techniques. Specifically, 18 studies used the random number table method, while the remaining 4 used various techniques: permuted block randomization (36), paired randomization (41), random allocation software (78), and stratified randomization (88). These studies were classified as having a low risk of bias. Studies that did not specify their randomization methods were categorized as having an unclear risk of bias. Three studies (48, 75, 82) implemented allocation concealment through group randomization, whereas other studies did not report this approach. Additionally, 20 studies specified the use of double-blinding, while 3 studies (44, 53, 71) indicated blinding of outcome assessors. The remaining studies did not provide details on blinding procedures for investigators, patients, or outcome assessors. All studies with available data were generally assessed as having a low risk of bias. Furthermore, there was no evidence of other biases or selective reporting across the trials.

#### 3.1.3 Glycemic indicators

**Figure 3:**
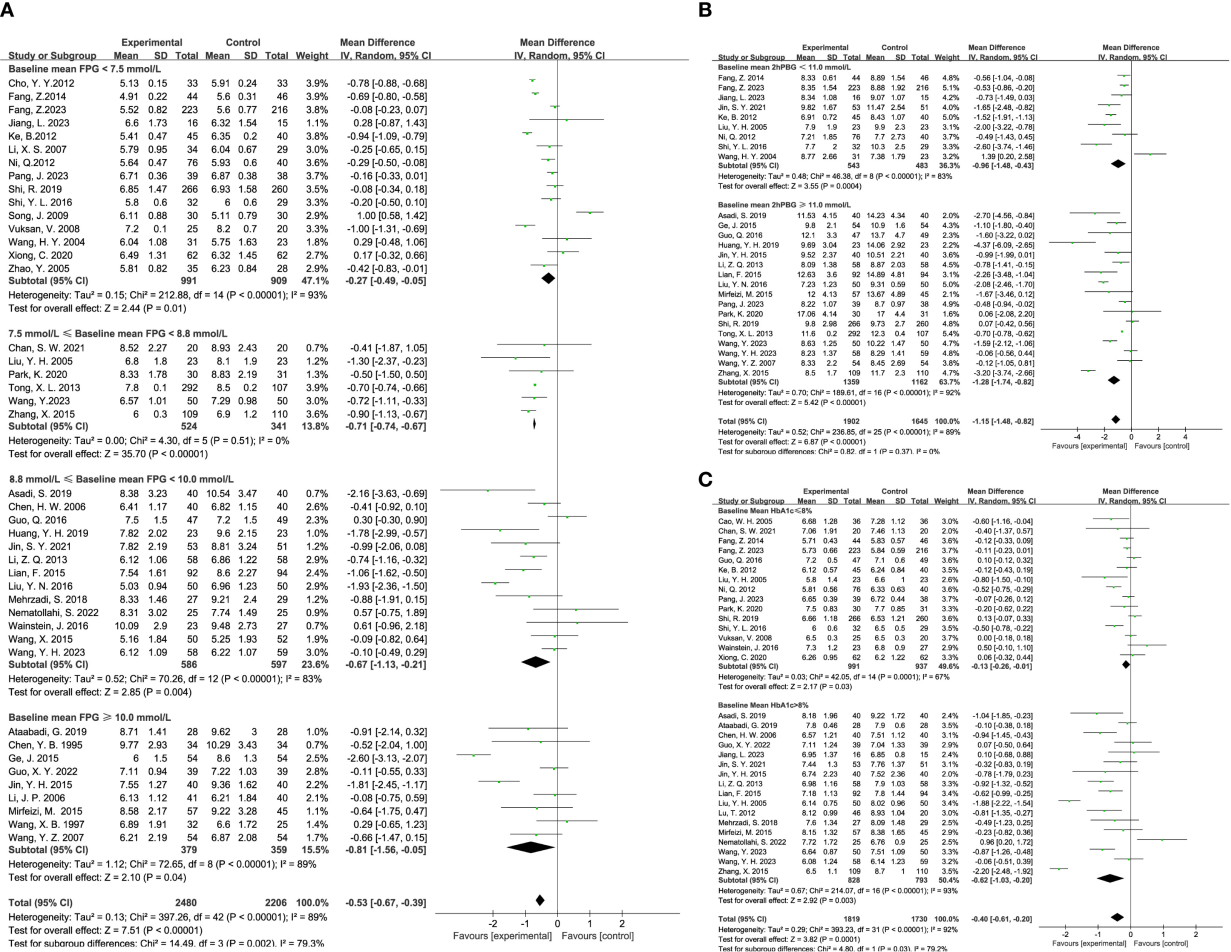
Results of a meta-analysis: **(A)** Forest plot and subgroup analysis of Fasting Plasma Glucose comparison between TCM and control group, **(B)** Forest plot and subgroup analysis of Postprandial Plasma Glucose comparison between TCM and control group, **(C)** Forest plot and subgroup analysis of Glycated Hemoglobin comparison between TCM and control group.

The impact of TCM on blood glucose levels was assessed across several key metrics. Fasting blood glucose levels were analyzed across 43 studies, encompassing 2,480 participants in the experimental group and 2,206 in the control group. The analysis demonstrated that TCM treatment significantly reduced fasting blood glucose levels in diabetic patients compared to the control group (MD = -0.53, 95% CI = [-0.67, -0.39], P < 0.00001), as shown in **Figure 3A**. For 2-hour postprandial blood glucose levels, data from 26 studies were evaluated, involving 1,902 participants in the experimental group and 1,645 in the control group. The results indicated that TCM treatment significantly decreased 2-hour postprandial blood glucose levels in diabetic patients compared to the control group (MD = -1.15, 95% CI = [-1.48, -0.82], P < 0.00001), as illustrated in **Figure 3B**. Twenty-two studies assessed HbA1c levels, including 1,123 participants in the experimental group and 977 in the control group. The analysis revealed that TCM treatment significantly lowered HbA1c levels in diabetic patients compared to the control group (MD = -0.40, 95% CI = [-0.61, -0.20], P = 0.0001), as depicted in **Figure 3C**.

#### 3.1.4 Insulin levels

**Figure 4:**
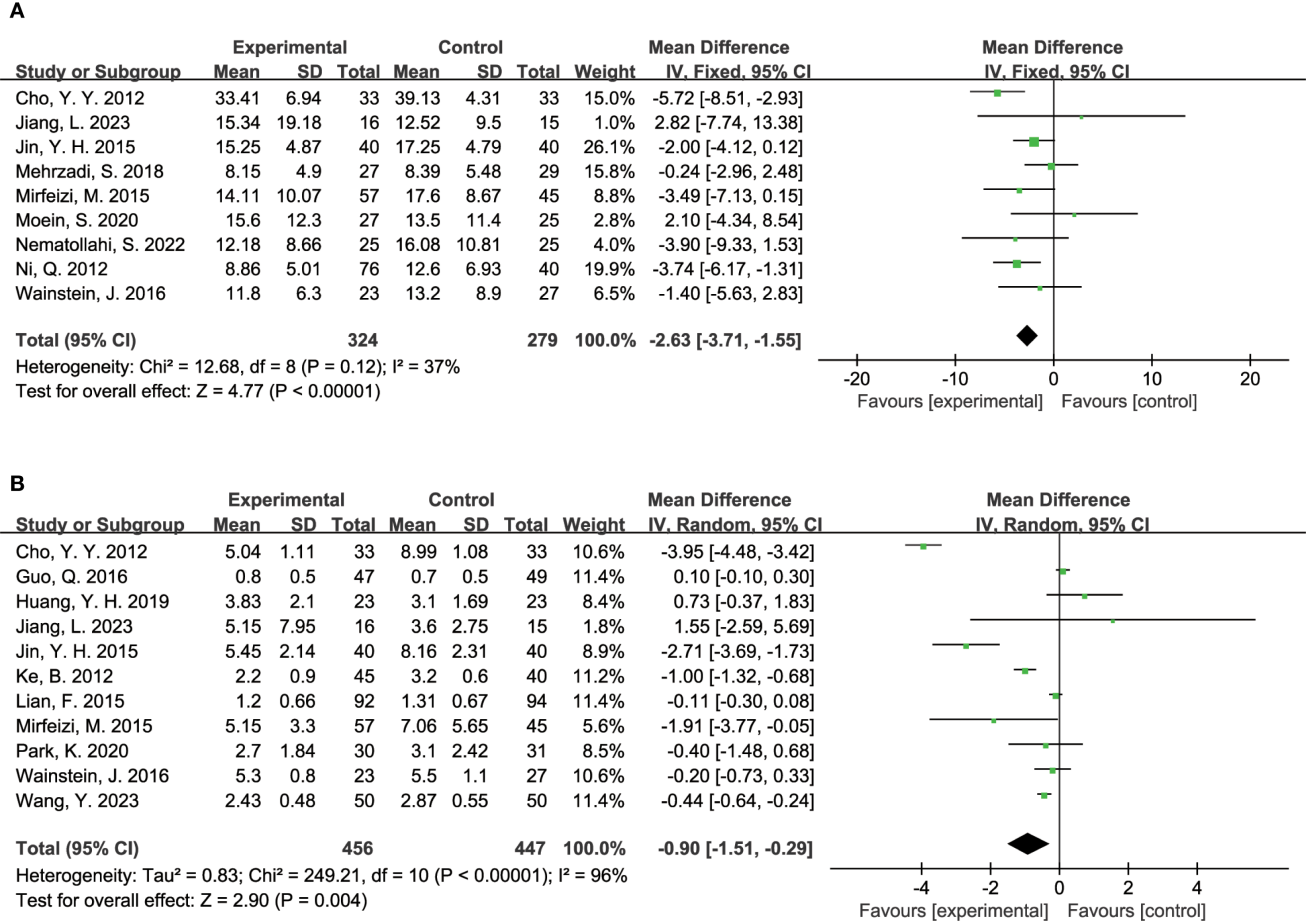
Results of a meta-analysis: **(A)** Forest plot of Fasting Insulin comparison between TCM and control group, **(B)** Forest plot of Homeostatic Model Assessment of Insulin Resistance comparison between TCM and control group.

The effect of TCM on insulin-related metrics was evaluated through several studies. Eight studies reported on fasting insulin levels, including 324 participants in the experimental group and 279 in the control group. The analysis revealed that TCM significantly reduced fasting insulin levels in diabetic patients compared to the control group (MD = -2.63, 95% CI = [-3.71, -1.55], P < 0.00001), as illustrated in **Figure 4A**. Additionally, 11 studies assessed HOMA-IR (Homeostasis Model Assessment of Insulin Resistance), with 1,123 participants in the experimental group and 977 in the control group. The results indicated that TCM treatment significantly decreased HOMA-IR levels in diabetic patients compared to the control group (MD = -0.90, 95% CI = [-1.51, -0.29], P = 0.004), as depicted in **Figure 4B**.

#### 3.1.5 Kidney function level

**Figure 5:**
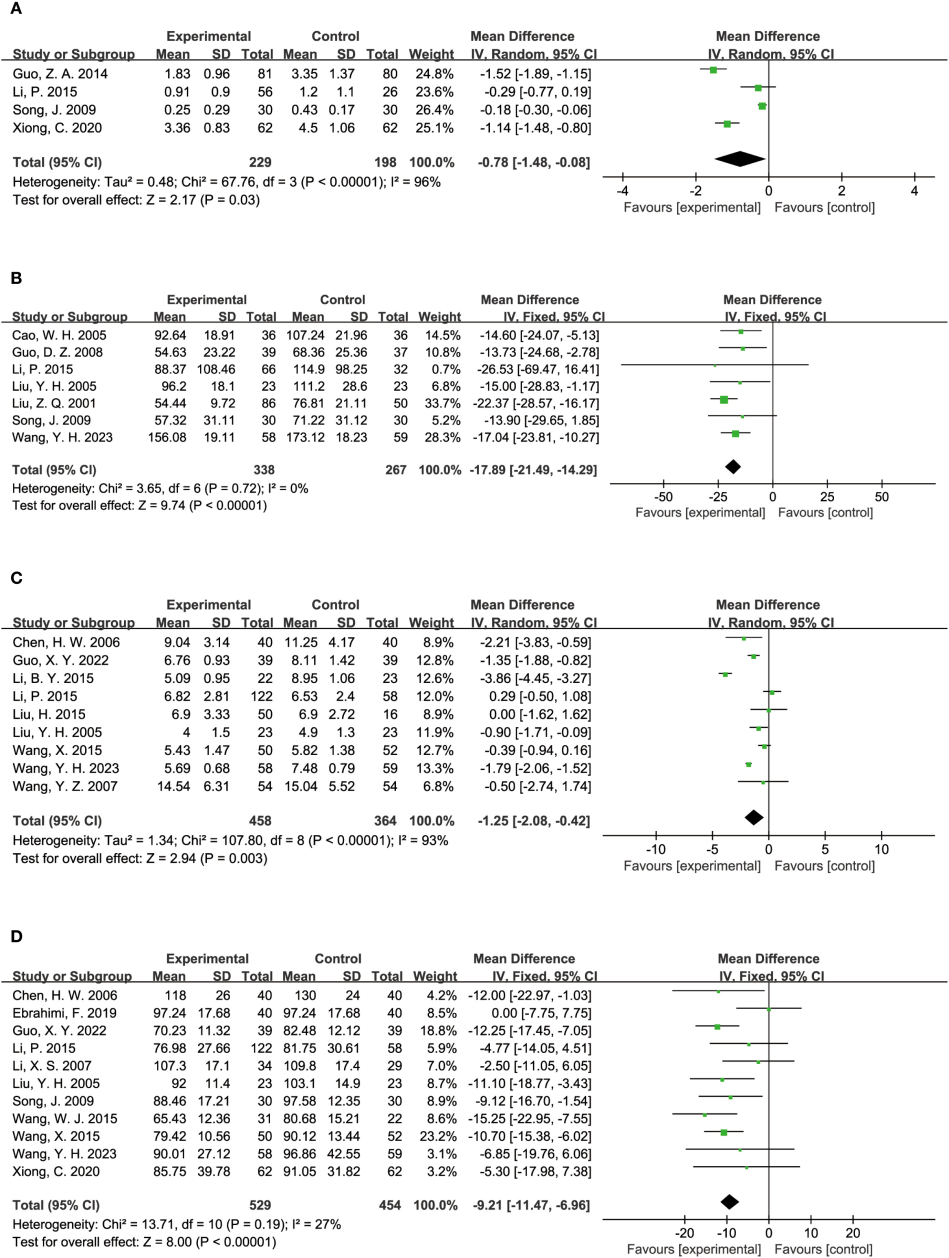
Results of a meta-analysis: **(A)** Forest plot of 24-hours Proteinuria comparison between TCM and control group, **(B)** Forest plot of Urinary Albumin Excretion Rate comparison between TCM and control group, **(C)** Forest plot of Blood Urea Nitrogen between TCM and control group, **(D)** Forest plot of Serum Creatinine comparison between TCM and control group.

The impact of TCM on kidney function was assessed through several key indicators across various studies. Four studies reported on 24-hour proteinuria, with 229 participants in the experimental group and 198 in the control group. The analysis demonstrated that TCM significantly reduced 24-hour proteinuria levels compared to the control group (MD = -0.78, 95% CI = [-1.48, -0.08], P = 0.03), as shown in **Figure 5A**. UAER (Urinary Albumin Excretion Rate) was evaluated in 7 studies, including 338 participants in the experimental group and 267 in the control group. The results indicated that TCM significantly lowered UAER levels compared to the control group (MD = -17.89, 95% CI = [-21.49, -14.29], P < 0.00001), as depicted in **Figure 5B**. BUN (Blood Urea Nitrogen) levels were assessed in 9 studies, involving 458 participants in the experimental group and 364 in the control group. The findings revealed that TCM significantly reduced BUN levels compared to the control group (MD = -1.25, 95% CI = [-2.08, -0.42], P = 0.003), as illustrated in **Figure 5C**. Scr (Serum Creatinine) levels were reported in 11 studies, including 529 participants in the experimental group and 454 in the control group. The analysis showed that TCM significantly lowered Scr levels compared to the control group (MD = -9.21, 95% CI = [-11.47, -6.96], P < 0.00001), as shown in **Figure 5D**.

#### 3.1.6 Blood lipid indicators

**Figure 6:**
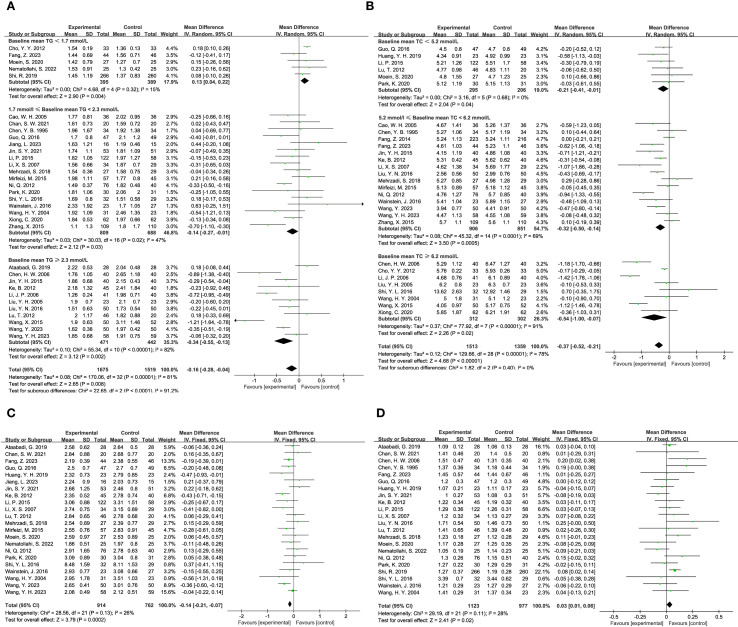
Results of a meta-analysis: **(A)** Forest plot and subgroup analysis of Triglyceride comparison between TCM and control group, **(B)** Forest plot and subgroup analysis of Total Cholesterol comparison between TCM and control group, **(C)** Forest plot of Low-density Lipoprotein between TCM and control group, **(D)** Forest plot of High-density Lipoprotein between TCM and control group.

The influence of TCM on blood lipid levels was examined across several studies. Triglyceride levels were reported in 33 studies, involving 1,675 participants in the experimental group and 1,519 in the control group. The analysis indicated that TCM significantly reduced triglyceride levels compared to the control group (MD = -0.16, 95% CI = [-0.28, -0.04], P = 0.008), as shown in **Figure 6A**. Total cholesterol levels were assessed in 29 studies, with 1,513 participants in the experimental group and 1,359 in the control group. The results demonstrated that TCM significantly lowered total cholesterol levels compared to the control group (MD = -0.37, 95% CI = [-0.52, -0.21], P < 0.00001), as depicted in **Figure 6B**. LDL (Low-Density Lipoprotein) levels were reported in 22 studies, including 914 participants in the experimental group and 762 in the control group. The findings revealed that TCM significantly reduced LDL levels compared to the control group (MD = -0.14, 95% CI = [-0.21, -0.07], P = 0.0002), as illustrated in **Figure 6C**. HDL (High-Density Lipoprotein) levels were analyzed in 22 studies, with 1,123 participants in the experimental group and 762 in the control group. The results indicated that TCM significantly improved HDL levels compared to the control group (MD = 0.03, 95% CI = [0.01, 0.06], P = 0.02), as shown in **Figure 6D**.

#### 3.1.7 Others

**Figure 7:**
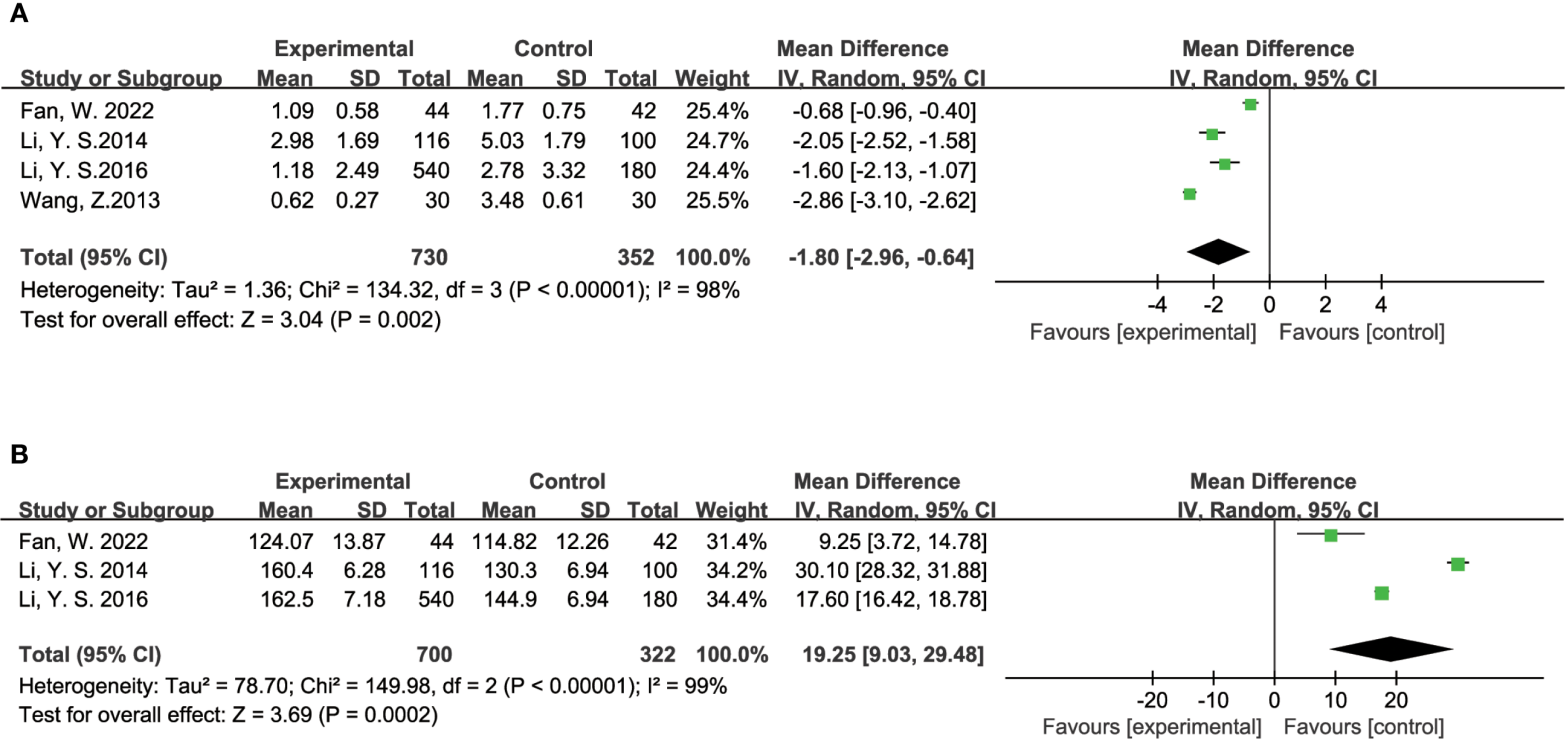
Results of a meta-analysis: **(A)** Forest plot of Ulcer Area of diabetes foot comparison between TCM and control group, **(B)** Forest plot of Vascular Endothelial Growth Factor in patients with diabetes foot comparison between TCM and control group.

The efficacy of TCM in managing diabetic foot was evaluated through various indicators. Five studies examined the ulcer area in diabetic foot patients, with a total of 780 participants in the experimental group and 402 in the control group. The analysis showed a significant reduction in ulcer area for those receiving TCM compared to the control group (MD = -1.80, 95% CI = [-2.96, -0.64], P = 0.002), as illustrated in **Figure 7A**. In addition to ulcer area, three studies investigated the levels of Vascular Endothelial Growth Factor (VEGF), which plays a crucial role in wound healing and vascularization. This analysis included 700 participants in the experimental group and 322 in the control group. The findings indicated that TCM treatment substantially lowered VEGF levels in diabetic foot patients compared to the control group (MD = -19.25, 95% CI = [-29.48, -9.03], P = 0.0002), as shown in **Figure 7B**.

#### 3.1.8 Sensitivity analysis and publication bias

To evaluate the robustness of our meta-analysis results, sensitivity analysis was conducted by systematically excluding each study one at a time and re-assessing the remaining studies. This approach ensured that no single study had a disproportionate impact on the overall outcomes of the meta-analysis, confirming the stability and reliability of our findings. In addition, publication bias was assessed using both Begg’s and Egger’s tests. The results of these tests indicated no evidence of publication bias, as shown by the relevant P values in **Supplementary Table S1**. The outcomes of the sensitivity analysis, along with the corresponding funnel plots, are presented in **Supplementary Figure S1** through S5.

### 3.2 Network pharmacology

#### 3.2.1 Effective herbs extraction

We examined the frequency of herbs in the TCM formulas cited in the included studies. Based on their occurrence, the most commonly used and effective herbs were *Astragalus mongholicus* Bunge (Fabaceae, Astragali radix), *Codonopsis pilosula* Nannf. (Campanulaceae, Codonopsis pilosulae radix), *Wolfiporia cocos (F.A. Wolf)* Ryvarden & Gilb. (Polyporacea, *Wolfiporia cocos* sclerotium), Cornus officinalis Siebold & Zucc. (Cornaceae, *Cornus officinalis* fruit), *Coptis chinensis* Franch. (Ranunculaceae, *Coptis chinensis* radix), Alisma *gramineum Lej*. (Alismataceae, *Alisma gramineum* tuber) (**Table 3**). A new formula, incorporating these six herbs, was created for network pharmacology analysis.

**Table 3:** The high-frequency Chinese herbs in each study.

Chinese name	Full botanical plant names	Counts	Frequency 1 (counts/total herb counts)	Frequency 2 (counts/study numbers)
Huangqi	*Astragalus mongholicus* Bunge	25	5.76%	43.10%
Dangshen	*Codonopsis pilosula* Nannf.	16	3.69%	27.59%
Fuling	*Wolfiporia cocos (F.A. Wolf)* Ryvarden & Gilb.	16	3.69%	27.59%
Shanzhuyu	*Cornus officinalis* Siebold & Zucc.	13	3.00%	22.41%
Huanglian	*Coptis chinensis* Franch.	12	2.76%	20.69%
Zexie	*Alisma gramineum* Lej.	9	2.07%	15.52%

The plant names have been verified through https://www.worldfloraonline.org on March 13, 2025.

#### 3.2.2 Screening of drug-diabetes genes and construction of drug-ingredient-target

**Figure 8:**
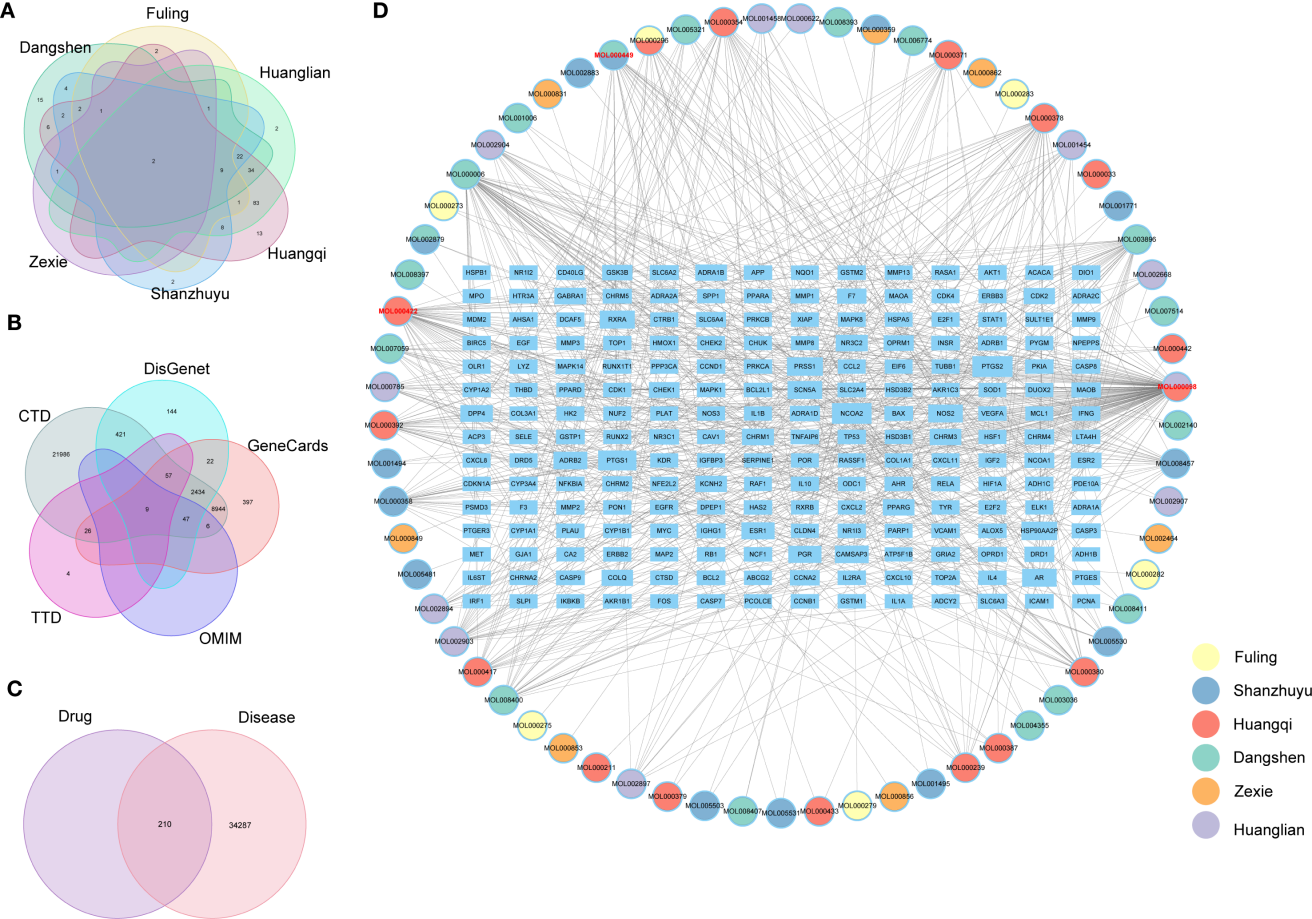
**(A)** Venetian diagram of drug targets of important traditional Chinese medicines, **(B)** Venetian diagram of diabetes disease targets, **(C)** Venetian diagram of traditional Chinese medicine-disease intersection genes, **(D)** Network diagram of drug ingredients and drug-disease targets.

Initially, we pinpointed the target genes associated with the active ingredients of TCM. Our search of the prescription database identified six effective TCMs, encompassing 67 compounds and 210 target genes. A Venn diagram illustrating the overlap of drug target genes across these six TCMs was constructed (**Figure 8A**). This analysis revealed that several TCMs share common target genes, suggesting a potential for multiple TCMs to influence the same genetic targets. Subsequently, we identified disease-related genes associated with diabetes, retrieving a total of 34,497 diabetes-related targets from five public disease databases (**Figure 8B**). The Venn diagram analysis indicated an intersection of 210 drug-disease targets (**Figure 8C**). Finally, we utilized Cytoscape to develop a network diagram that integrates TCM components with their corresponding disease targets, as shown in **Figure 8D**. The results suggest that a specific component of traditional Chinese medicine often corresponds to multiple molecular targets of diabetes. We ranked them according to the frequency of the corresponding targets. The top three are quercetin, kaempferol, and Stigmasterol. For the rest of the results, please refer to the **Supplementary Table S2**.

#### 3.2.3 Screening of core genes and functional enrichment analysis

**Figure 9:**
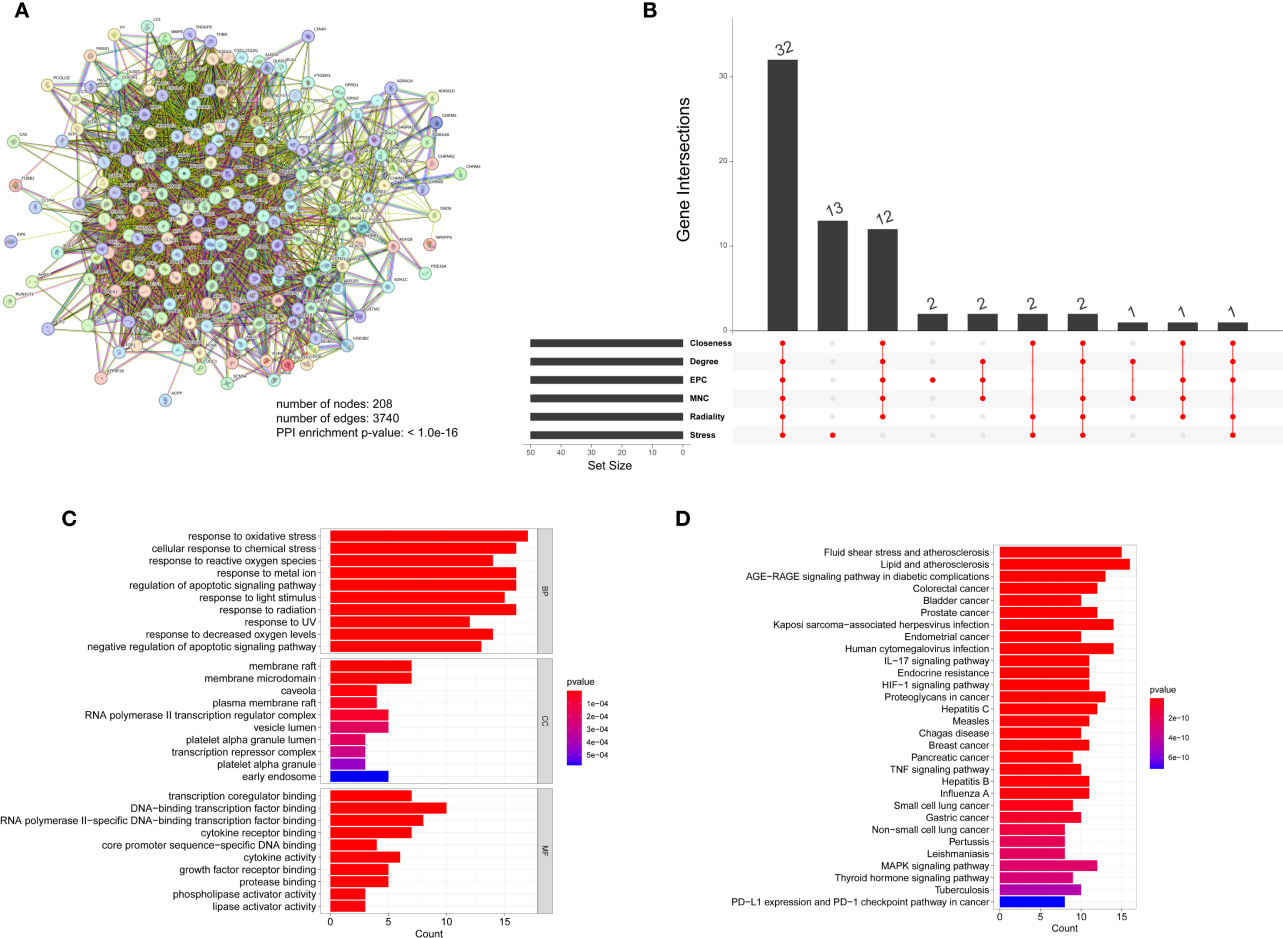
**(A)** Protein molecular network diagram of TCM-disease intersection genes, **(B)** Venetian display, 6 algorithms screened out 32 core genes in total, **(C)** GO analysis of core genes, **(D)** KEGG analysis of core genes.

The TCM-targets were subsequently uploaded to the STRING database, which produced a network consisting of 208 nodes and 3,740 edges, and a PPI enrichment p-value of ≤ 1.0e-16 (**Figure 9A**). By applying seven algorithms from the cytoHubba plugin, we identified the top 50 genes, detailed in **Supplementary Table S3**. Through intersection analysis, we isolated 32 core genes that fulfilled all criteria. These include AKT1, IL1B, TP53, PTGS2, ESR1, CASP3, MMP9, EGFR, BCL2, HIF1A, FOS, MYC, PPARG, GSK3B, CCND1, EGF, ERBB2, IL10, CCL2, IFNG, CXCL8, IL1A, ICAM1, RELA, MMP2, HMOX1, NFE2L2, APP, CASP9, MAPK1, SERPINE1, and CAV1 (**Figure 9B**). Gene Ontology (GO) analysis demonstrated that these core genes are predominantly involved in the cellular responses to oxidative stress and oxygen levels (**Figure 9C**, **Supplementary Table S4**). Additionally, KEGG pathway analysis revealed that these genes are mainly associated with the TNF signaling pathway, the IL-17 signaling pathway, and the HIF-1 signaling pathway (**Figure 9D**, **Supplementary Table S5**). These pathways are intricately connected to diabetes.

## 4 Discussion

Diabetes is a global chronic metabolic condition with a rising incidence, presenting significant health risks and economic challenges for patients. Traditional Chinese medicine (TCM) adheres to a holistic view of health, regarding the human body as an organic whole influenced by both internal and external factors. Its various components are interconnected and mutually influential, forming a complex dynamic system. TCM, with its multi-target, multi-component, and multi-pathway approach, offers considerable promise as a comprehensive treatment for diabetes (94). Research indicates that the potential mechanisms by which TCM regulates blood glucose include: improving insulin resistance (95–97), enhancing insulin sensitivity (98–100), promoting insulin secretion (101–103), and stimulating glucose uptake (104–106). Notably, recent studies have found that TCM can also improve glucose metabolism disorders by remodeling the balance of the gut microbiota (101, 107, 108), working through the perspective of the “Gut-Pancreas” axis. However, although existing retrospective studies have confirmed the clinical value of TCM (26, 28), there remain significant gaps in screening the optimal medication regimens and elucidating the molecular mechanisms of action. Therefore, this study aims to provide new scientific evidence for TCM in treating diabetes, in order to promote more precise individualized treatment strategies.

This meta-analysis provides robust evidence that TCM can significantly improve glycemic control (e.g., HbA1c, fasting blood glucose), lipid profile (e.g., total cholesterol, triglycerides, low-density lipoprotein cholesterol), and renal function indicators (e.g., serum creatinine, urinary albumin excretion rate) in diabetic patients. These improvements are directly linked to the pathophysiological mechanisms of core diabetic complications: optimizing glycemic fluctuations reduces microvascular damage (109); regulating dyslipidemia delays atherosclerosis (110); and protecting nephron function lowers the risk of nephropathy (111). This suggests that TCM intervention holds significant clinical value in the comprehensive management of diabetes and its common complications. This conclusion is supported by the consistently observed effects in the included studies and the results obtained after our rigorous assessment of potential biases and heterogeneity.

The synergistic improvements in blood glucose, lipids, and renal function observed in this study possess dual clinical significance. On one hand, they pertain to the restoration of metabolic homeostasis: the simultaneous optimization of blood glucose and lipids may directly delay vascular endothelial damage by reducing glucolipotoxicity (112, 113). On the other hand, they indicate a forward shift in the window for complication prevention: improvements in Scr and BUN suggest TCM may potentially block the “hyperglycemia-glomerular hypertension-renal fibrosis” pathway, offering a novel strategy for the primary prevention of diabetic nephropathy (114). This provides evidence-based support for an integrated approach combining Chinese and Western medicine, aligning with the “individualized metabolic goal management” proposed in the ADA/EASD guidelines.

The meta-analysis results further indicate that *Astragali Radix* (Huangqi), *Codonopsis Radix* (Dangshen), *Poria* (Fuling), *Corni Fructus* (Shanzhuyu), *Coptidis Rhizoma* (Huanglian), and *Alismatis Rhizoma* (Zexie) are the high-frequency herbs used in TCM for diabetes treatment. Relevant studies support their efficacy. For instance, Chao et al. found that a traditional Chinese herbal compound (containing *Coptidis Rhizoma*, *Astragali Radix, and Lonicerae Japonicae Flos*) ameliorated insulin resistance in T2D patients and also improved glucose metabolism (including FPG, PPG, and HbA1c) and blood pressure to some extent (115). A multicenter study by Chan et al. demonstrated that adding Astragali Radix to standard treatment significantly slowed the decline in renal function in patients with diabetic nephropathy (116). The mechanisms by which these herbs treat diabetes align with those described previously: by optimizing glycemic fluctuations, regulating dyslipidemia, and protecting renal function to reduce the risk of nephropathy. Specifically, *Corni Fructus* ameliorates diabetic nephropathy through its effects of lowering blood glucose, regulating lipids, and reducing oxidative stress; its renal protective mechanism is closely associated with activating the PPARγ signaling pathway (117). The aqueous extract of *Codonopsis Radix* ameliorates insulin resistance (IR) by increasing Akt and GSK-3β phosphorylation, reduces hepatic triglyceride content via AMPK phosphorylation, and protects β-cell function by reducing β-cell apoptosis (118). Furthermore, extracts of *Codonopsis Radix* and *Polygonati Rhizoma* (Huangjing) can also ameliorate IR, lower blood glucose, and reduce lipid levels by activating the IRS1/PI3K/AKT pathway (119). For *Astragali Radix*, its polysaccharide component (APS) promotes GLUT4 translocation and upregulates PPAR-γ expression by activating the AMPK/PI3K/AKT pathway, thereby ameliorating IR. It also possesses anti-inflammatory effects, inhibits pancreatic β-cell apoptosis, and promotes insulin secretion (120). Meanwhile, both the saponin (ASS) and flavonoid (ASF) components of Astragali Radix combat hyperglycemia by activating the adiponectin-AMPK pathway and its downstream factors, although their effect intensities vary across different tissues (121).

Notably, the network pharmacology analysis in this study identified quercetin, kaempferol, and stigmasterol as the core active components of the aforementioned high-frequency TCM herbs (e.g., *Astragali Radix, Codonopsis Radix, Corni Fructus*). A total of 32 potential targets for TCM in treating diabetes were identified. These active components exert their therapeutic effects through synergistic regulation of core pathological pathways in diabetes. Specifically, quercetin activates the AMPK/PI3K/Akt pathway to enhance GLUT4 translocation efficiency in skeletal muscle, promoting glucose uptake and increase insulin sensitivity (122, 123). It also inhibits the activation of the TNF-α/NF-κB pathway by enhancing Akt phosphorylation, thereby alleviating inflammatory insulin resistance (124, 125). Furthermore, quercetin inhibits ferroptosis by activating the Nrf2 pathway (e.g., upregulating GPX4/xCT) and chelating iron ions. In the kidneys, this manifests as protection of renal tubular mitochondria and reduction of proteinuria. In the pancreas, it improves insulin secretion by reducing iron deposition in β-cells, thereby providing protection against diabetic kidney disease (DKD) and for pancreatic β-cells (126, 127). By activating PPARγ, kaempferol enhances downstream PI3K/AKT signaling activity to promote glucose uptake in peripheral tissues and inhibit hepatic gluconeogenesis, thereby effectively lowering blood glucose and improving glucose tolerance, while ameliorating lipid metabolism disorders and reducing lipotoxicity through activation of the PPARγ/LXRα/ABCA1 pathway (128, 129). Kaempferol also inhibits IKKβ/IKKα phosphorylation and activation, blocking the TNF-α/NF-κB pathway, reducing serum pro-inflammatory cytokines, and increasing IRS-1 protein expression, thereby improving insulin resistance (130). It has also been found to modulate the gut microbiota (e.g., reversing the relative abundance of Firmicutes and Bacteroidetes), which may be related to its amelioration of high-fat diet-induced lipid metabolism abnormalities (131). In terms of renal protection, kaempferol inhibits RhoA/ROCK signaling, downregulates pro-fibrotic factors such as TGF-β1 and CTGF, reduces extracellular matrix (ECM) accumulation, protects podocyte structure, and delays renal fibrosis while improving renal function (132). Stigmasterol acts by increasing the expression of SREBP2 and its target gene LDLR while decreasing the expression of the cholesterol efflux transporter ABCA1. This reduces free cholesterol levels induced by glucolipotoxicity. Combined with its antioxidant effects to lower ROS, stigmasterol restores glucose-stimulated insulin secretion (GSIS) capacity, increases total insulin content, and alleviates pancreatic β-cell dysfunction (133). Additionally, stigmasterol increases GLUT4 translocation and expression, enhancing insulin sensitivity to improve insulin resistance (134).

This association pattern of “TCM - active component clusters - multi-target pathways - synergistic effects” profoundly elucidates the material basis and mechanisms of action underlying TCM in treating diabetes. The core components (quercetin, kaempferol, stigmasterol) identified by network pharmacology and their 32 regulated targets not only validate the efficacy mechanisms of the high-frequency TCM herbs but also reveal, at the molecular level, how different herbs collectively target core pathological aspects of diabetes. This collective action occurs through shared or complementary active components and action pathways, addressing key pathological processes including insulin resistance, glucose and lipid metabolism disorders, inflammatory responses, oxidative stress, β-cell dysfunction, and renal injury. Ultimately, this leads to the synergistic improvement of blood glucose, lipid profile, and renal function. This provides a critical scientific foundation for understanding the holistic effects of TCM formulas and for developing novel therapeutic strategies based on active component clusters.

Beyond the small-molecule active components mentioned above, macromolecular components in TCM (such as polysaccharides and polypeptides) have been widely confirmed to possess significant anti-diabetic activity. Particularly in the core herbs screened in this study, the effects of APS on improving insulin resistance and modulating gut microbiota (120, 135), the hypoglycemic and renal protective effects of Coptidis Rhizoma polysaccharides (CCPW) (136, 137), and the antioxidant and anti-inflammatory activities of Codonopsis Radix polysaccharides (CPPS) and Corni Fructus polysaccharides (COPs) (138, 139) are all significant contributors to their overall therapeutic efficacy. However, it should be noted that the network pharmacology methodology applied subsequently in this study has an inherent design primarily reliant on small-molecule-oriented databases (e.g., TCMSP, PubChem) and prediction tools (e.g., molecular docking). Consequently, it is challenging to systematically analyze the complex mechanisms of action of these macromolecular components. The primary value of this study lies in its focus on the clusters of small-molecule compounds within these same core herbs (e.g., quercetin/kaempferol in *Astragali Radix*, berberine/palmatine in *Coptidis Rhizoma*, alisol derivatives in *Alismatis Rhizoma*). It reveals the potential molecular mechanisms by which they exert anti-diabetic effects through the regulation of key targets (e.g., AKT1, PPARG) and signaling pathways (e.g., TNF signaling pathway). This provides an important perspective for understanding the small-molecule pharmacodynamic material basis of the core herbs. We emphasize that the overall efficacy of high-frequency herbs like *Astragali Radix* and *Codonopsis Radix* stems from the synergistic action of their multiple components. This includes both the small-molecule mechanisms predicted in this study (e.g., targeted regulation of inflammatory factors like TNF or the PI3K-Akt pathway) and the well-documented macromolecular components (e.g., the immune/gut microbiota modulatory functions of polysaccharides). Together, they constitute a modern scientific interpretation of TCM’s characteristic “multi-component, multi-target” integrative regulation. Future studies should integrate multi-omics approaches and macromolecule-specific methodologies to more comprehensively parse the synergistic networks of both large and small molecular components within the core herbs.

This multi-target and multi-component characteristic is the core of the unique advantages of TCM in treating diabetes. Unlike Western medicine with a single target, TCM for the treatment of diabetes usually does not rely on a single ingredient or a single medicine, but adopts a compound form. TCM can intervene in the complex pathological mechanism of diabetes in an all-round way through the synergistic effect of multiple active ingredients. The multiple drugs in the combination can synergize or inhibit each other, enhancing the efficacy and reducing the side effects. This not only helps to lower blood sugar, but also may have a protective effect on diabetes-related complications such as neuropathy, nephropathy and cardiovascular disease. While this study highlights the considerable potential of TCM in treating diabetes, its clinical application continues to encounter several challenges. First of all, the efficacy and safety of TCM are affected by many factors, including the origin of the medicinal materials, processing methods and individual differences. Secondly, most current clinical trials have problems such as small sample sizes and loose designs, which limit the wide application of the results. Therefore, future research should include more large-scale, high-quality randomized controlled trials to further validate the effectiveness and safety of traditional Chinese medicine for treating diabetes. In addition, diabetes is a highly heterogeneous disease with significant differences in pathophysiology between patients. The personalized treatment approach of TCM, which involves modifying drug formulas based on the patient’s unique condition, constitution, and other factors, may lead to more effective outcomes. Additionally, since diabetes is a chronic condition necessitating ongoing management, it is important to assess the long-term efficacy and safety of TCM treatments.

It is important to specifically note that the meta-analysis in this study only included adult patients aged 18 and above. While this aligns with the population scope of most current randomized controlled trials, it may limit the applicability of the study conclusions to adolescent patients with youth-onset type 2 diabetes mellitus (T2DM). Notably, the disease burden of this special population has been rapidly increasing worldwide in recent years, with overweight/obesity further elevating the risk (140, 141). Existing evidence indicates that, compared to youth-onset type 1 diabetes mellitus (T1DM), youth-onset T2DM not only has a worse clinical prognosis but also exhibits more pronounced metabolic abnormalities and potentially distinct pathogenesis compared to adult-onset T2DM. These characteristics collectively lead to an elevated risk of vascular complications in young patients (142). More concerningly, the current treatment options approved by the U.S. FDA, the European Medicines Agency (EMA), and Health Canada for youth-onset T2DM are very limited. This scarcity of treatment choices may lead to more adverse clinical outcomes for this population. Based on this, our future research will expand the cohort of young patients to systematically evaluate the synergistic effects of TCM-modern drug combination therapy, analyze differential treatment responses among different age groups, and elucidate the mechanisms of action of TCM interventions for youth-onset T2DM. These studies will provide crucial evidence for developing targeted intervention strategies, holding significant clinical value for preventing disease progression and reducing the risk of complications in adolescent patients.

Of course, this study also has some limitations. First, our meta-analysis and network pharmacology analysis are based on existing literature and databases, which may have problems with publication bias and incomplete data. Second, the prediction results of network pharmacology analysis need further experimental verification. Moreover, the complexity of TCM involves various components that may undergo intricate metabolic processes and interactions within the body. These processes not only affect the efficacy of the drug, but may also cause adverse reactions. Our understanding of its multi-component and multi-target mechanisms is still limited, and further research is needed in combination with more systems biology approaches. Future research should focus on the following aspects: First, conducting large-scale, high-quality clinical trials to provide more reliable evidence support; Second, conducting an in-depth analysis of the multi-component and multi-target mechanisms of TCM using multi-omics technologies and systems biology approaches.; Third, exploring the combined application of TCM and modern drugs to give play to their synergistic and enhancing effects; Fourth, strengthening the standardization and quality control of TCM to ensure the safety and effectiveness of clinical application.

## 5 Conclusions

This study systematically evaluated the efficacy and potential mechanisms of TCM in the treatment of diabetes through meta-analysis and network pharmacology. The results indicated that TCM can significantly improve blood glucose control in diabetic patients, reduce glycated hemoglobin levels, and alleviate insulin resistance. Additionally, network pharmacology analysis revealed that TCM exerts its effects through multiple targets and pathways, including the regulation of insulin signaling pathways, inflammatory responses, and oxidative stress. These findings provide scientific evidence for the use of TCM in diabetes treatment, supporting its clinical application.

## Data Availability

The original contributions presented in the study are included in the article/**Supplementary Material**. Further inquiries can be directed to the corresponding author.
