# Supplementary material for: Evaluating efficacy and mechanism of traditional Chinese medicine in diabetes treatment: a meta-analysis and network pharmacology study
Source: Front Endocrinol (Lausanne). 2025 Oct 7;16:1605091. doi: 10.3389/fendo.2025.1605091 (PMC12537397; doi:10.3389/fendo.2025.1605091)

The summarization and selection of articles for meta-analysis to systematically evaluate the relationship between traditional Chinese medicine and diabetes (using medicines mentioned at least seven times for further analysis).

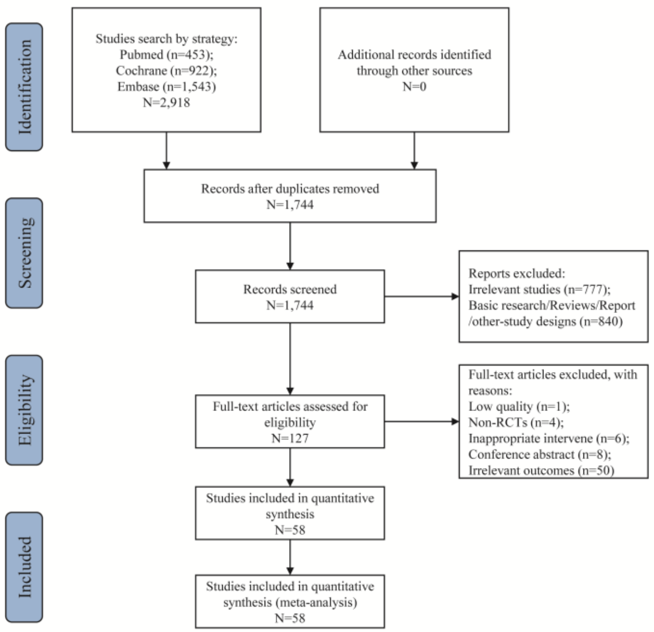

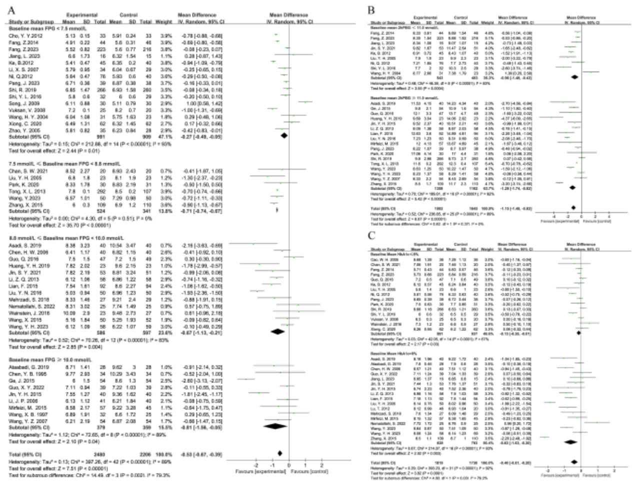

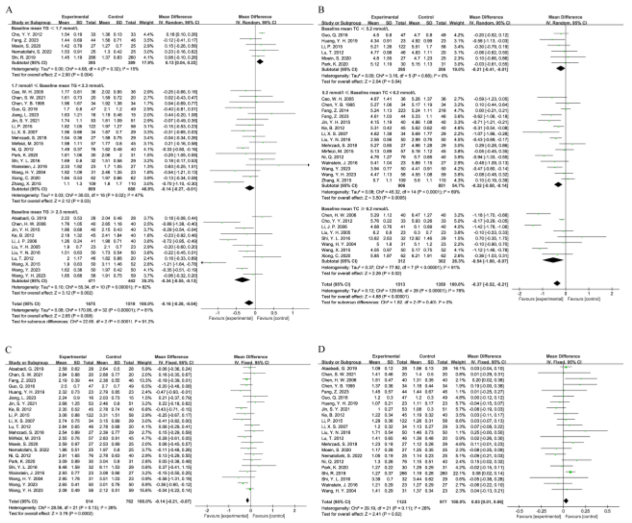

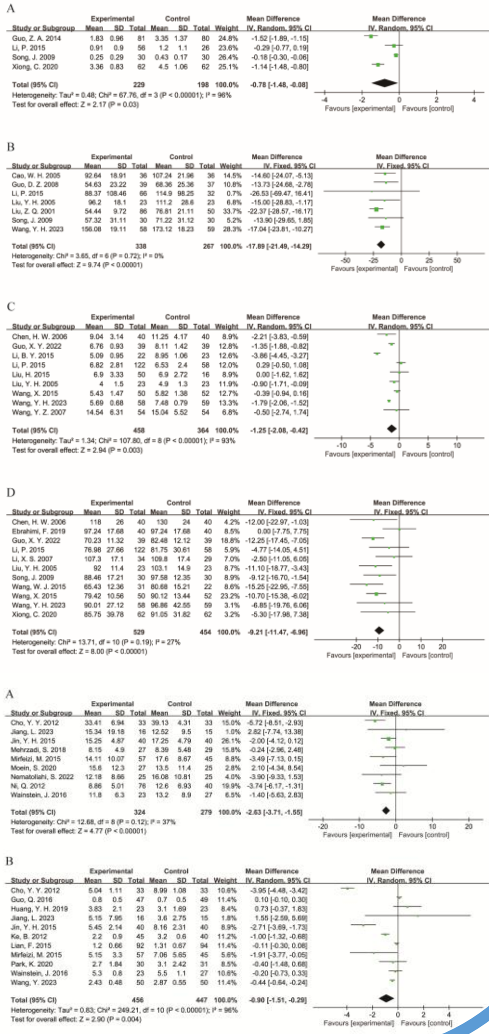

The retrieval of drug targets for six traditional Chinese medicines from the TCMSF.

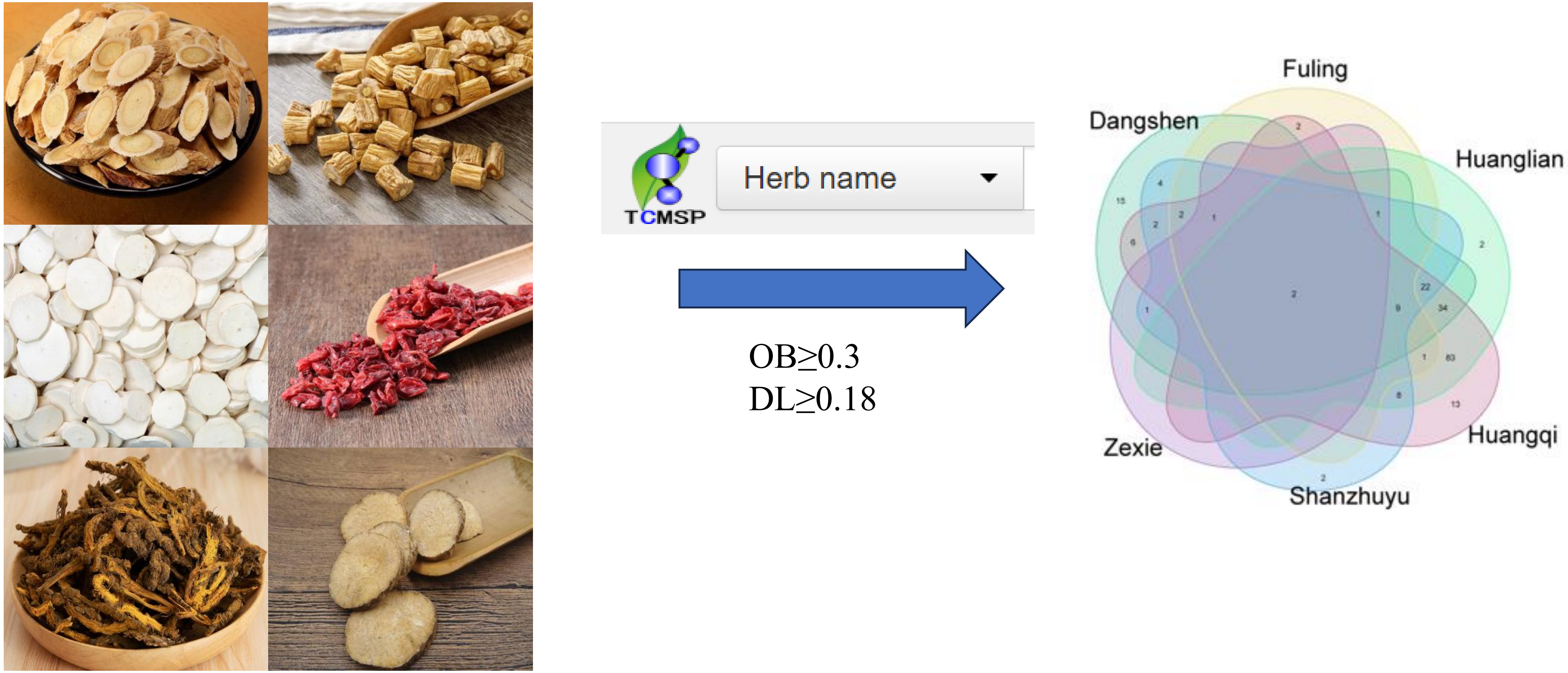

Obtain diabetes targets through multiple databases.

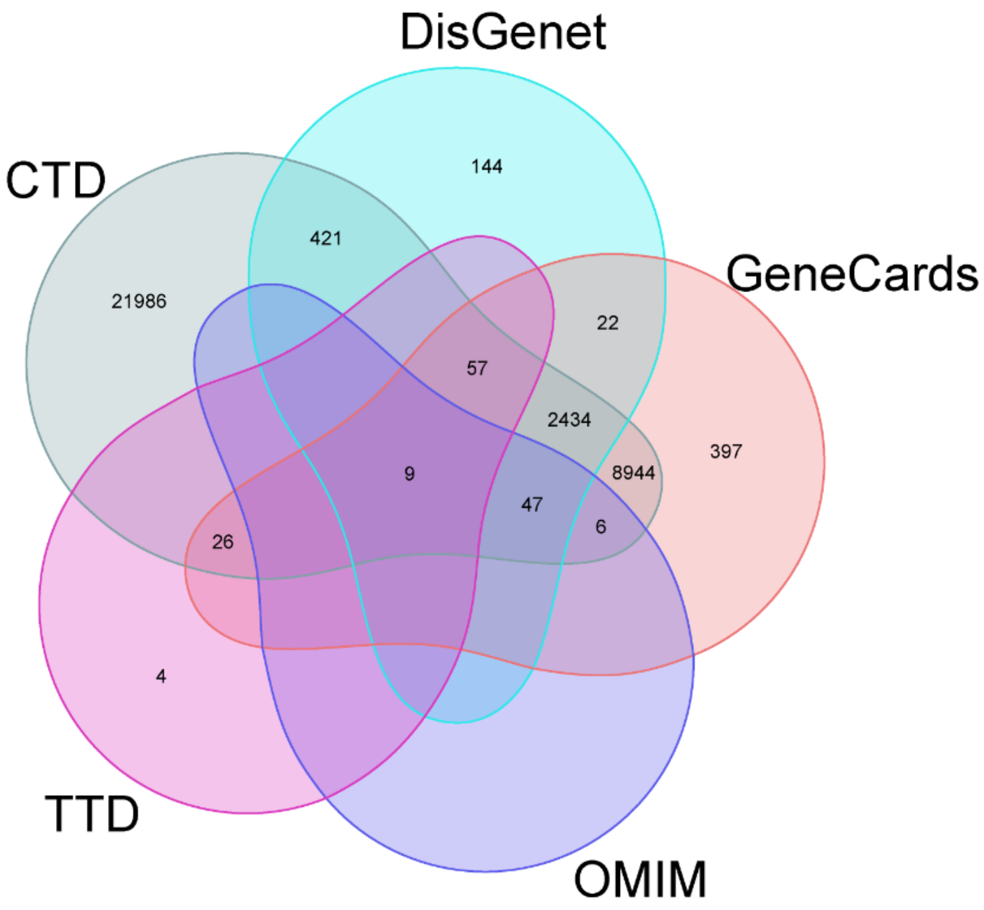

The identification of core genes associated with traditional Chinese medicine and diseases, and the construction of a network diagram illustrating their relationship with medicinal components.

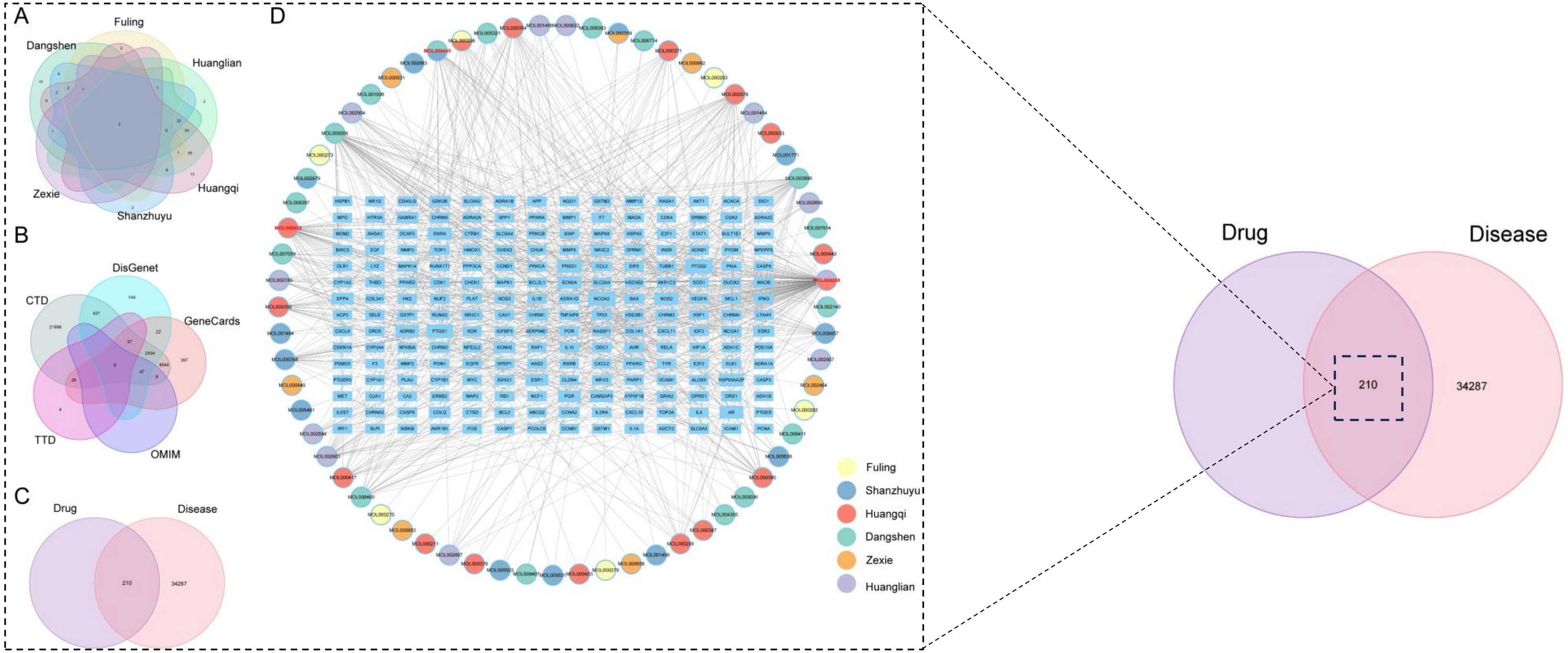

The core genes of diabetes and traditional Chinese medicine are determined by various algorithms.

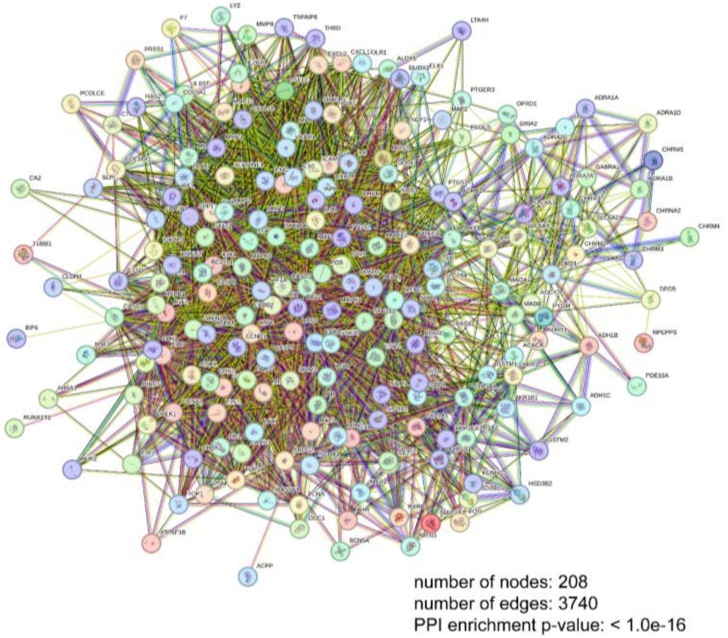

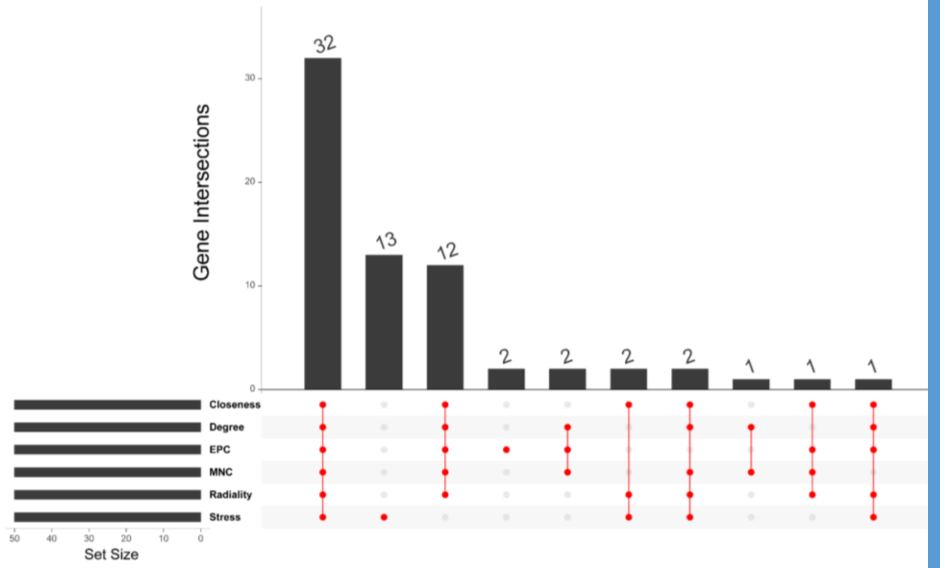

Enrichment analysis of core gene functions.

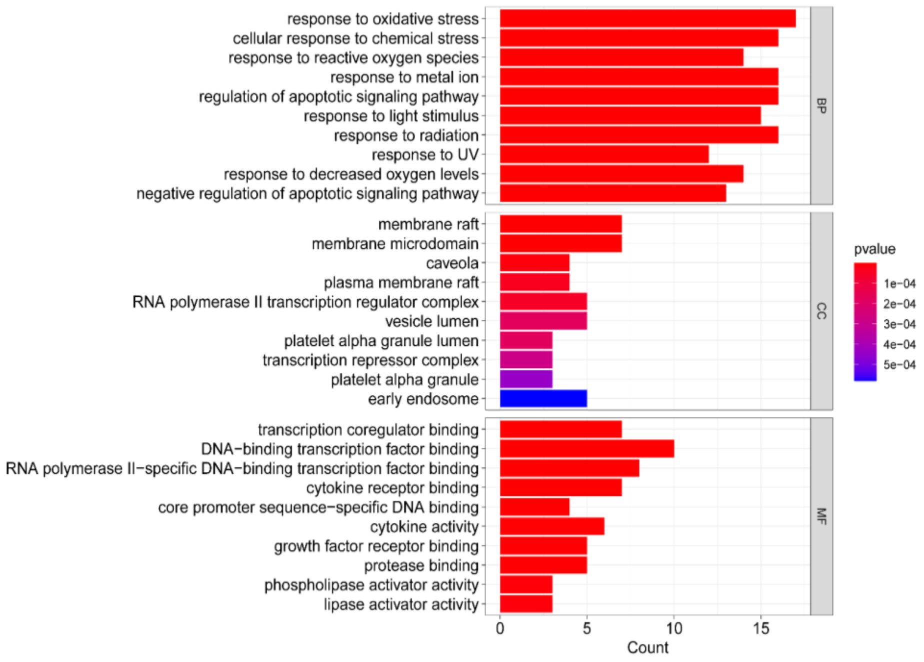

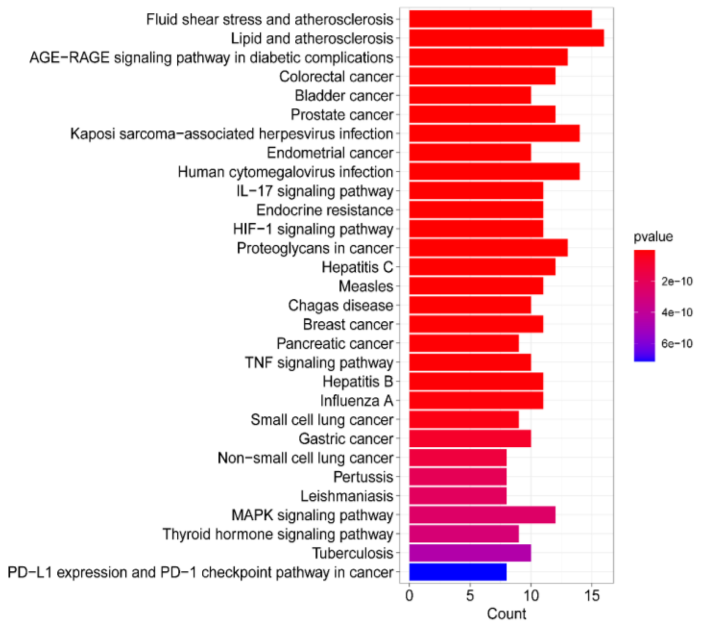

Supplement: Supplementary file 1 [file DataSheet1.pdf]
