# Supplementary material for: Evaluating efficacy and mechanism of traditional Chinese medicine in diabetes treatment: a meta-analysis and network pharmacology study
Source: Front Endocrinol (Lausanne). 2025 Oct 7;16:1605091. doi: 10.3389/fendo.2025.1605091 (PMC12537397; doi:10.3389/fendo.2025.1605091)
Supplement: Supplementary file 2 [file DataSheet2.docx]

***SUPPLEMENTARY MATERIAL***

1. **Supplementary Methods**

**Supplementary Method 1**

1. **Supplementary Figures**

**Supplementary Figure S1** – (A) Sensitivity analysis of Fasting Plasma Glucose in included RCTs. (B) Sensitivity analysis of Postprandial Plasma Glucose in included RCTs. (C) Sensitivity analysis of Fasting Glycated Hemoglobin in included RCTs. (D) Begg’s funnel plot of Fasting Plasma Glucose in included RCTs. (E) Begg’s funnel plot of Postprandial Plasma in included RCTs. (F) Begg’s funnel plot of Glycated Hemoglobin in included RCTs.

**Supplementary Figure S2** – (A) Sensitivity analysis of Fasting Insulin in included RCTs. (B) Sensitivity analysis of Homeostatic Model Assessment of Insulin Resistance in included RCTs. (C) Begg’s funnel plot of Fasting Insulin in included RCTs. (D) Begg’s funnel plot of Homeostatic Model Assessment of Insulin Resistance in included RCTs.

**Supplementary Figure S3** – (A) Sensitivity analysis of 24-hours Proteinuria in included RCTs. (B) Sensitivity analysis of Urinary Albumin Excretion Rate in included RCTs. (C) Sensitivity analysis of Blood Urea Nitrogen in included RCTs. (D) Sensitivity analysis of Serum Creatinine in included RCTs. (E) Begg’s funnel plot of 24-hours Proteinuria in included RCTs. (F) Begg’s funnel plot of Urinary Albumin Excretion Rate in included RCTs. (G) Begg’s funnel plot of Blood Urea Nitrogen in included RCTs. (H) Begg’s funnel plot of Serum Creatinine in included RCTs.

**Supplementary Figure S4** – (A) Sensitivity analysis of Triglyceride in included RCTs. (B) Sensitivity analysis of Total Cholesterol Excretion Rate in included RCTs. (C) Sensitivity analysis of Low-density Lipoprotein in included RCTs. (D) Sensitivity analysis of High-density Lipoprotein in included RCTs. (E) Begg’s funnel plot of Triglyceride in included RCTs. (F) Begg’s funnel plot of Total Cholesterol Excretion Rate in included RCTs. (G) Begg’s funnel plot of Low-density Lipoprotein in included RCTs. (H) Begg’s funnel plot of High-density Lipoprotein in included RCTs.

**Supplementary Figure S5** – (A) Sensitivity analysis of diabetes foot in included RCTs. (B) Sensitivity analysis of Vascular Endothelial Growth Factor in patients with diabetes foot in included RCTs. (C) Begg’s funnel plot of diabetes foot in included RCTs. (D) Begg’s funnel plot of Vascular Endothelial Growth Factor in patients with diabetes foot in included RCTs.

1. **Supplementary Table**

**Supplementary Table S1** – Publication bias of the combined diabetes related outcomes

**Supplementary Table S2** **–** Frequency table of corresponding compounds of six major TCMs

**Supplementary Table S3** – Top 50 genes of six algorithms

**Supplementary Table S4** – GO analysis of 32 core genes

**Supplementary Table S5** – KEGG analysis

**Supplementary Method 1**

**PubMed：**

The combined text and medical subject heading (MeSH) terms were cross-searched using MeSHand free word as follows: (Diabetes Mellitus [Mesh] OR Diabetes Mellitus, Experimental [Title/Abstract] OR Diabetes Mellitus, Type 1 [Title/Abstract] OR Wolfram Syndrome [Title/Abstract] OR Diabetes Mellitus, Type 2 [Title/Abstract] OR Diabetes Mellitus, Lipoatrophic [Title/Abstract] OR Diabetes, Gestational [Title/Abstract] OR Donohue Syndrome [Title/Abstract] OR Latent Autoimmune Diabetes in Adults [Title/Abstract] OR Prediabetic State [Title/Abstract] OR hyperglycemia [Title/Abstract] OR Diabetes, Gestational [Mesh] OR Diabetes, Pregnancy-Induced [Title/Abstract] OR Diabetes, Pregnancy Induced [Title/Abstract] OR Pregnancy-Induced Diabetes [Title/Abstract] OR Gestational Diabetes [Title/Abstract] OR Diabetes Mellitus, Gestational [Title/Abstract] OR Gestational Diabetes Mellitus [Title/Abstract]) AND (Medicine, Chinese Traditional [Mesh] OR traditional medicine [Title/Abstract] OR traditional Chinese medicine [Title/Abstract] OR Traditional Medicine, Chinese [Title/Abstract] OR TCM [Title/Abstract] OR Chinese medicine [Title/Abstract] OR Chinese Traditional Medicine [Title/Abstract] OR Chinese Medicine, Traditional [Title/Abstract] OR Chung I Hsueh [Title/Abstract] OR Hsueh, Chung I [Title/Abstract] OR Zhong Yi Xue [Title/Abstract] OR Traditional Tongue Diagnosis [Title/Abstract] OR Tongue Diagnoses, Traditional [Title/Abstract] OR Tongue Diagnosis, Traditional [Title/Abstract] OR Traditional Tongue Diagnoses [Title/Abstract] OR Traditional Tongue Assessment [Title/Abstract] OR Tongue Assessment, Traditional [Title/Abstract] OR Traditional Tongue Assessments [Title/Abstract] OR alternative medicine [Title/Abstract] OR complementary medicine [Title/Abstract] OR Drugs, Chinese Herbal [Title/Abstract] OR Chinese Drugs, Plant [Title/Abstract] OR Chinese Herbal Drugs [Title/Abstract] OR Herbal Drugs, Chinese [Title/Abstract] OR Chinese herbal medicine [Title/Abstract] OR herbal medicine [Title/Abstract] OR herbs [Title/Abstract] OR Chinese Herbal Drug [Title/Abstract] OR Chinese Herbal Medicine [Title/Abstract] OR Chinese Plant Extracts [Title/Abstract] OR Plant Extracts, Chinese [Title/Abstract] OR Extracts, Chinese Plant [Title/Abstract]) AND (Randomized Controlled Trial [Publication Type] OR Controlled Clinical Trial [Publication Type] OR Clinical Trials as Topic [Mesh:NoExp] OR randomized[Title/Abstract] OR placebo [Title/Abstract] OR randomly[Title/Abstract] OR trial[Title/Abstract]) NOT (Animals[Mesh] NOT Humans[Mesh])

**Cocrhane：**

ID Search

#1 MeSH descriptor: [Diabetes Mellitus] explode all trees

#2 MeSH descriptor: [Diabetes, Gestational] explode all trees

#3 (Diabetes Mellitus, Experimental):ti,ab,kw OR (Diabetes Mellitus, Type 1):ti,ab,kw OR (Wolfram Syndrome):ti,ab,kw OR (Diabetes Mellitus, Type 2):ti,ab,kw OR (Diabetes Mellitus, Lipoatrophic):ti,ab,kw OR (Diabetes, Gestational):ti,ab,kw OR (Donohue Syndrome):ti,ab,kw OR (Latent Autoimmune Diabetes in Adults):ti,ab,kw OR (Prediabetic State):ti,ab,kw OR (Diabetes, Pregnancy-Induced):ti,ab,kw OR (Diabetes, Pregnancy Induced):ti,ab,kw OR (Pregnancy-Induced Diabetes):ti,ab,kw OR (Gestational Diabetes):ti,ab,kw OR (Diabetes Mellitus, Gestational):ti,ab,kw OR (Gestational Diabetes Mellitus):ti,ab,kw

#4 #1 OR #2 OR #3

#5 MeSH descriptor: [Medicine, Chinese Traditional] explode all trees

#6 (traditional medicine):ti,ab,kw OR (traditional Chinese medicine):ti,ab,kw OR (Traditional Medicine, Chinese):ti,ab,kw OR (TCM):ti,ab,kw OR (Chinese medicine):ti,ab,kw OR (Chinese Traditional Medicine):ti,ab,kw OR (Chinese Medicine, Traditional):ti,ab,kw OR (Chung I Hsueh):ti,ab,kw OR (Hsueh, Chung I):ti,ab,kw OR (Zhong Yi Xue):ti,ab,kw OR (Traditional Tongue Diagnosis):ti,ab,kw OR (Tongue Diagnoses, Traditional):ti,ab,kw OR (Tongue Diagnosis, Traditional):ti,ab,kw OR (Traditional Tongue Diagnoses):ti,ab,kw OR (Traditional Tongue Assessment):ti,ab,kw OR (Tongue Assessment, Traditional):ti,ab,kw OR (Traditional Tongue Assessments):ti,ab,kw OR (alternative medicine):ti,ab,kw OR (complementary medicine):ti,ab,kw OR (Drugs, Chinese Herbal):ti,ab,kw OR (Chinese Drugs, Plant):ti,ab,kw OR (Chinese Herbal Drugs):ti,ab,kw OR (Herbal Drugs, Chinese):ti,ab,kw OR (Chinese herbal medicine):ti,ab,kw OR (herbal medicine):ti,ab,kw OR (herbs):ti,ab,kw OR (Chinese Herbal Drug):ti,ab,kw OR (Chinese Herbal Medicine):ti,ab,kw OR (Chinese Plant Extracts):ti,ab,kw OR (Plant Extracts, Chinese):ti,ab,kw OR (Extracts, Chinese Plant):ti,ab,kw

#7 #5 OR #6

#8 #4 AND #7

**Embase：**

| No. | Query | Results |
| --- | --- | --- |
| #8 | #5 AND #6 AND #7 | 1543 |
| #7 | 'randomized controlled trial':ab,ti OR 'controlled clinical trial':ab,ti OR 'randomized':ab,ti OR 'clinical trials as topic':ab,ti OR 'placebo':ab,ti OR 'randomly':ab,ti OR 'trial':ab,ti | 2213245 |
| #6 | #3 OR #4 | 170218 |
| #5 | #1 OR #2 | 1331494 |
| #4 | 'traditional medicine':ab,ti OR 'traditional chinese medicine':ab,ti OR 'traditional medicine, chinese':ab,ti OR 'tcm':ab,ti OR 'chinese medicine':ab,ti OR 'chinese traditional medicine':ab,ti OR 'chinese medicine, traditional':ab,ti OR 'chung i hsueh':ab,ti OR 'hsueh, chung i':ab,ti OR 'zhong yi xue':ab,ti OR 'traditional tongue diagnosis':ab,ti OR 'tongue diagnoses, traditional':ab,ti OR 'tongue diagnosis, traditional':ab,ti OR 'traditional tongue diagnoses':ab,ti OR 'traditional tongue assessment':ab,ti OR 'tongue assessment, traditional':ab,ti OR 'traditional tongue assessments':ab,ti OR 'alternative medicine':ab,ti OR 'complementary medicine':ab,ti OR 'drugs, chinese herbal':ab,ti OR 'chinese drugs, plant':ab,ti OR 'chinese herbal drugs':ab,ti OR 'herbal drugs, chinese':ab,ti OR 'herbal medicine':ab,ti OR 'herbs':ab,ti OR 'chinese herbal drug':ab,ti OR 'chinese herbal medicine':ab,ti OR 'chinese plant extracts':ab,ti OR 'plant extracts, chinese':ab,ti OR 'extracts, chinese plant':ab,ti | 135281 |
| #3 | 'chinese medicine'/exp | 77299 |
| #2 | 'diabetes mellitus, experimental':ab,ti OR 'diabetes mellitus, type 1':ab,ti OR 'wolfram syndrome':ab,ti OR 'diabetes mellitus, type 2':ab,ti OR 'diabetes mellitus, lipoatrophic':ab,ti OR 'diabetes, gestational':ab,ti OR 'donohue syndrome':ab,ti OR 'latent autoimmune diabetes in adults':ab,ti OR 'prediabetic state':ab,ti OR 'diabetes, pregnancy-induced':ab,ti OR 'diabetes, pregnancy induced':ab,ti OR 'pregnancy-induced diabetes':ab,ti OR 'gestational diabetes':ab,ti OR 'diabetes mellitus, gestational':ab,ti OR 'gestational diabetes mellitus':ab,ti | 44825 |
| #1 | 'diabetes mellitus'/exp OR 'gestational diabetes'/exp | 1328744 |

**Supplementary Figure S1** – (A) Sensitivity analysis of Fasting Plasma Glucose in included RCTs. (B) Sensitivity analysis of Postprandial Plasma Glucose in included RCTs. (C) Sensitivity analysis of Fasting Glycated Hemoglobin in included RCTs. (D) Begg’s funnel plot of Fasting Plasma Glucose in included RCTs. (E) Begg’s funnel plot of Postprandial Plasma in included RCTs. (F) Begg’s funnel plot of Glycated Hemoglobin in included RCTs.

**
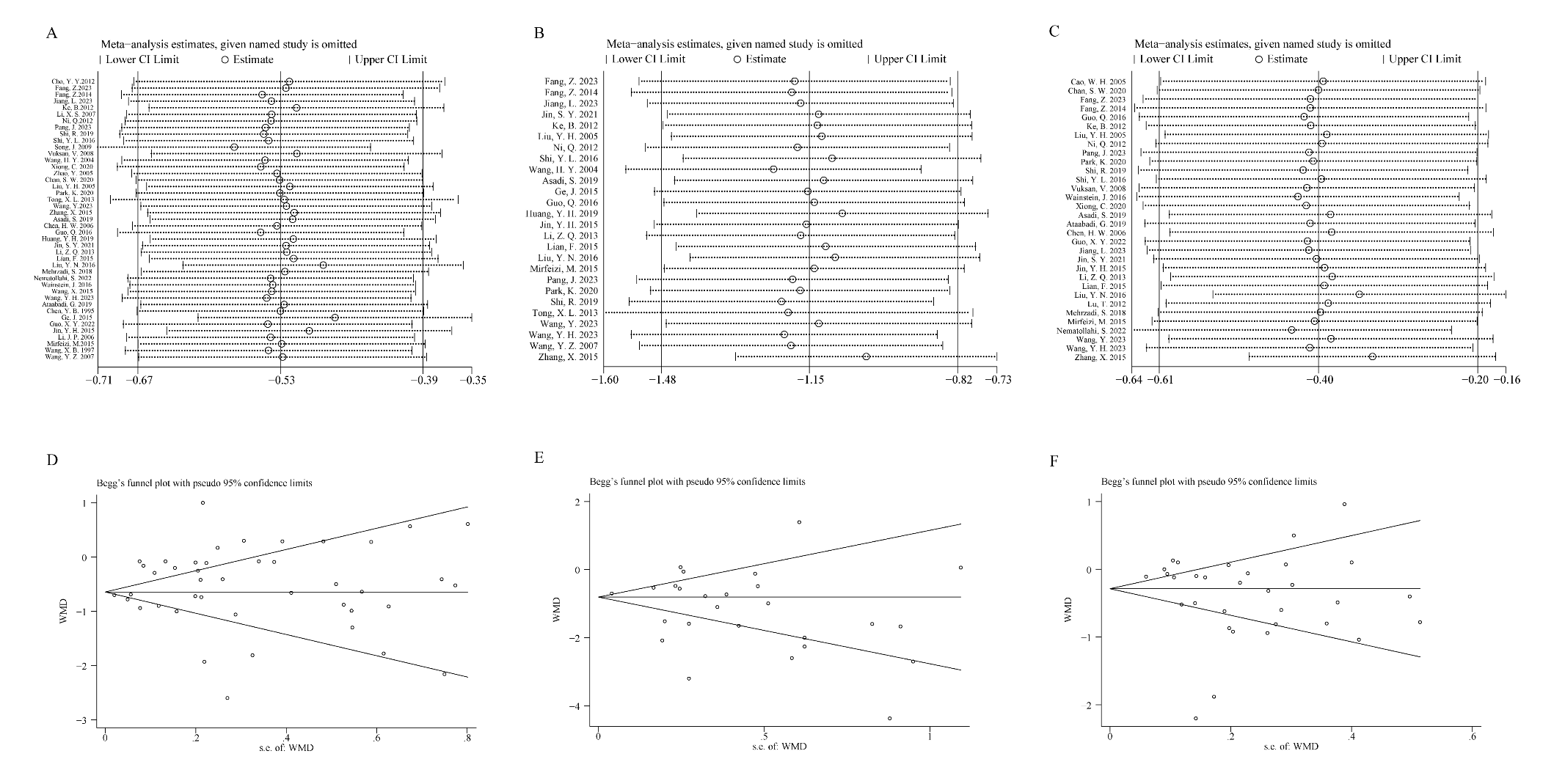
**

**Supplementary Figure S2** – (A) Sensitivity analysis of Fasting Insulin in included RCTs. (B) Sensitivity analysis of Homeostatic Model Assessment of Insulin Resistance in included RCTs. (C) Begg’s funnel plot of Fasting Insulin in included RCTs. (D) Begg’s funnel plot of Homeostatic Model Assessment of Insulin Resistance in included RCTs.

**
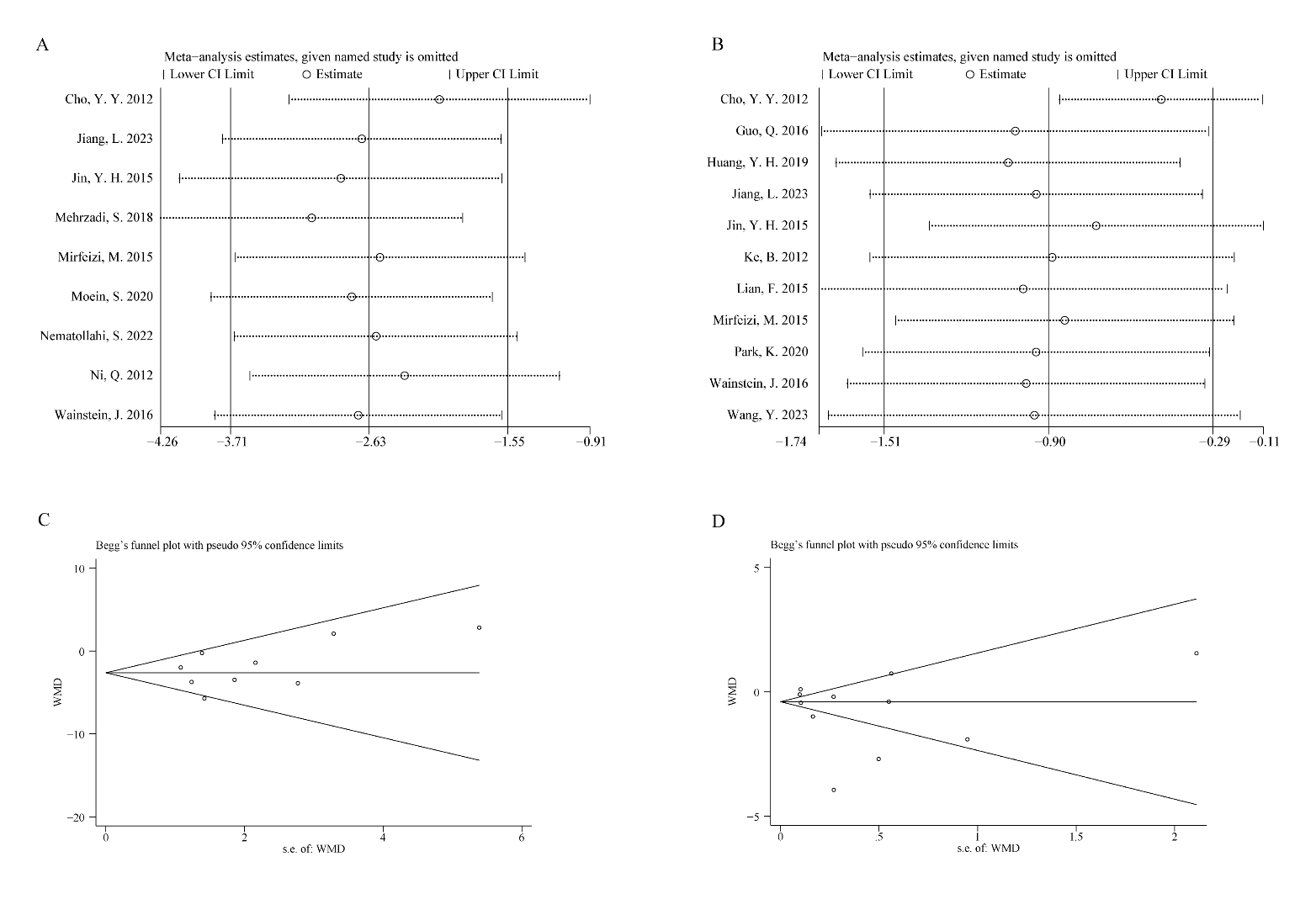
**

**Supplementary Figure S3** – (A) Sensitivity analysis of 24-hours Proteinuria in included RCTs. (B) Sensitivity analysis of Urinary Albumin Excretion Rate in included RCTs. (C) Sensitivity analysis of Blood Urea Nitrogen in included RCTs. (D) Sensitivity analysis of Serum Creatinine in included RCTs. (E) Begg’s funnel plot of 24-hours Proteinuria in included RCTs. (F) Begg’s funnel plot of Urinary Albumin Excretion Rate in included RCTs. (G) Begg’s funnel plot of Blood Urea Nitrogen in included RCTs. (H) Begg’s funnel plot of Serum Creatinine in included RCTs.

**
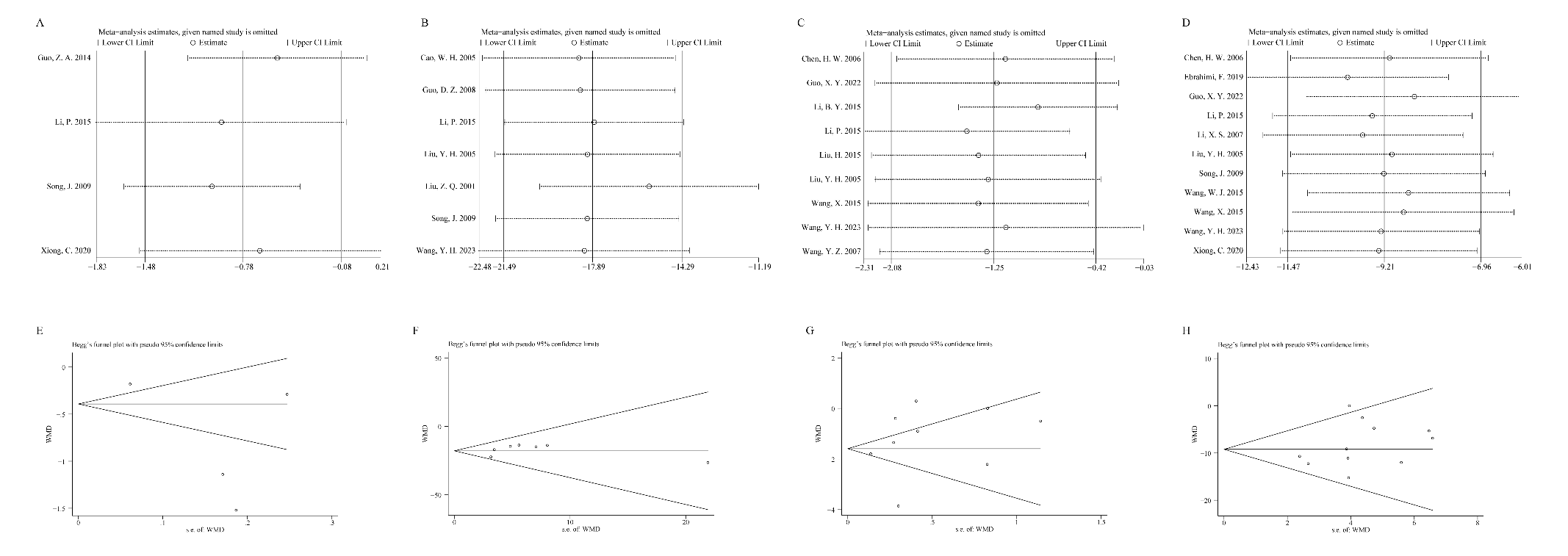
**

**Supplementary Figure S4** – (A) Sensitivity analysis of Triglyceride in included RCTs. (B) Sensitivity analysis of Total Cholesterol Excretion Rate in included RCTs. (C) Sensitivity analysis of Low-density Lipoprotein in included RCTs. (D) Sensitivity analysis of High-density Lipoprotein in included RCTs. (E) Begg’s funnel plot of Triglyceride in included RCTs. (F) Begg’s funnel plot of Total Cholesterol Excretion Rate in included RCTs. (G) Begg’s funnel plot of Low-density Lipoprotein in included RCTs. (H) Begg’s funnel plot of High-density Lipoprotein in included RCTs.

**
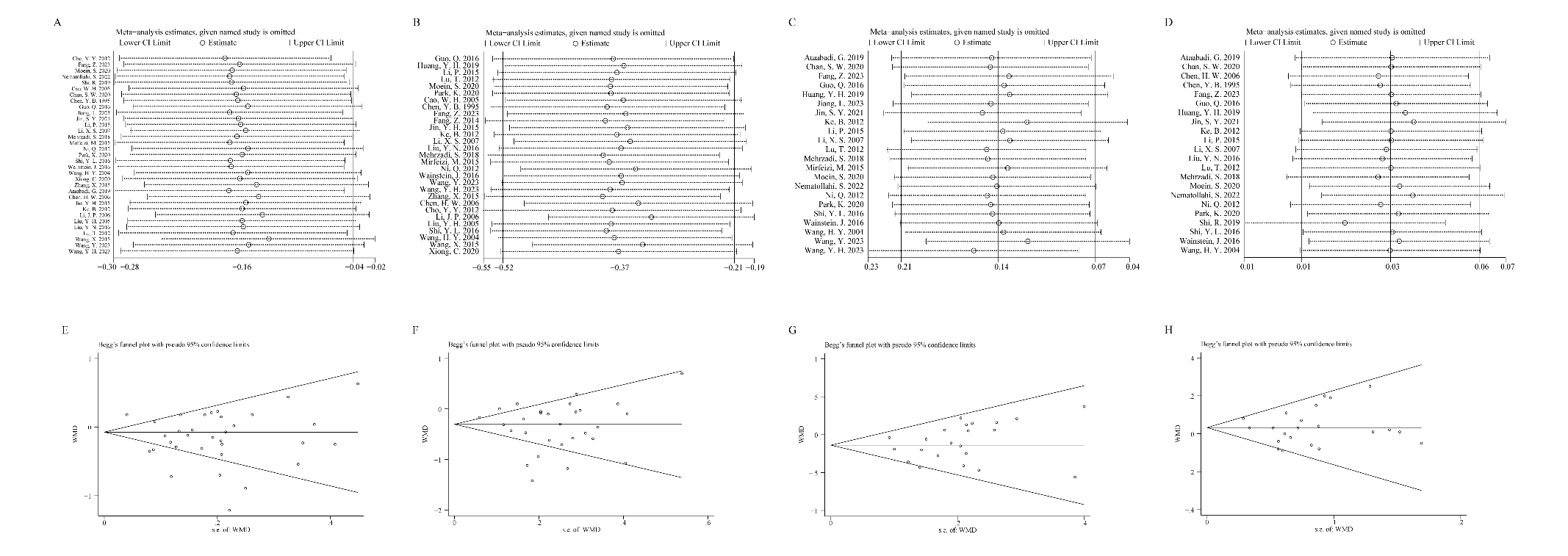
**

**Supplementary Figure S5** – (A) Sensitivity analysis of diabetes foot in included RCTs. (B) Sensitivity analysis of Vascular Endothelial Growth Factor in patients with diabetes foot in included RCTs. (C) Begg’s funnel plot of diabetes foot in included RCTs. (D) Begg’s funnel plot of Vascular Endothelial Growth Factor in patients with diabetes foot in included RCTs.


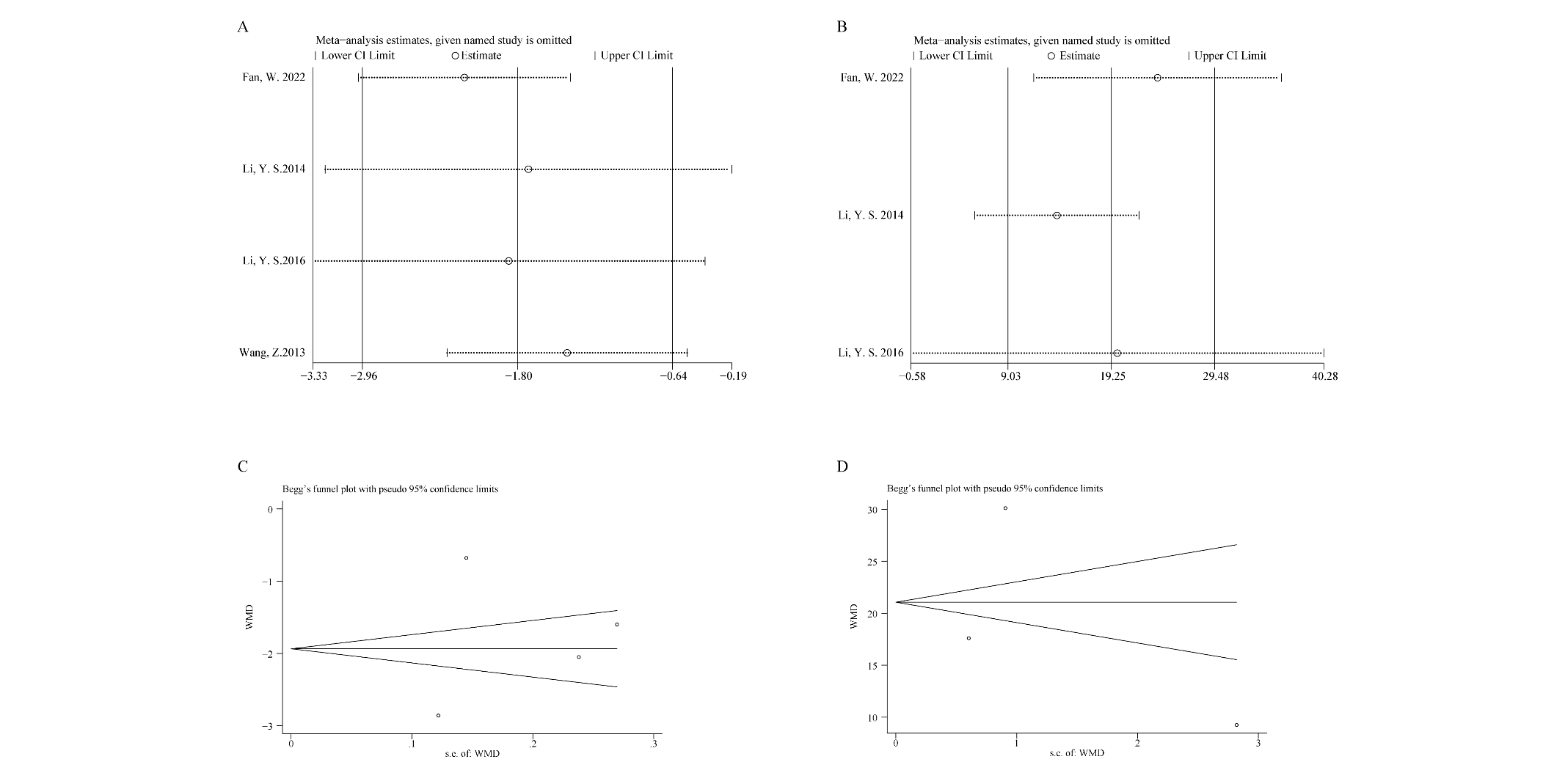


**Supplementary Table S1** Publication bias of the combined diabetes related outcomes

| Feature | No. of study | No. of patients | Begg's test P-value | Egger's test P-value |
| --- | --- | --- | --- | --- |
| FBG | 43 | 4686 | 0.53 | 0.232 |
| PBG | 26 | 3547 | 0.234 | 0.098 |
| HbA1c | 32 | 3549 | 0.2 | 0.19 |
| FI | 9 | 603 | 0.466 | 0.401 |
| HOMA-IR | 11 | 903 | 0.64 | 0.267 |
| 24-hours Proteinuria | 4 | 427 | 0.734 | 0.246 |
| UAER | 7 | 605 | 0.764 | 0.432 |
| BUN | 9 | 822 | 0.602 | 0.484 |
| Scr | 11 | 983 | 0.35 | 0.219 |
| TG | 33 | 3194 | 0.609 | 0.059 |
| TC | 29 | 2872 | 0.837 | 0.28 |
| LDL | 22 | 1676 | 0.114 | 0.248 |
| HDL | 22 | 2100 | 0.499 | 0.96 |
| Ulcer Area | 4 | 1082 | 0.734 | 0.743 |
| VEGF | 3 | 1022 | 1 | 0.997 |

**Supplementary Table S2** **–** Frequency table of corresponding compounds of six major TCMs

| MolName | MolId | Frequency |
| --- | --- | --- |
| quercetin | MOL000098 | 308 |
| kaempferol | MOL000422 | 63 |
| Stigmasterol | MOL000449 | 62 |
| luteolin | MOL000006 | 57 |
| hederagenin | MOL000296 | 48 |
| 7-O-methylisomucronulatol | MOL000378 | 45 |
| 7-Methoxy-2-methyl isoflavone | MOL003896 | 43 |
| formononetin | MOL000392 | 39 |
| beta-sitosterol | MOL000358 | 38 |
| isorhamnetin | MOL000354 | 37 |
| (R)-Canadine | MOL002903 | 31 |
| Tetrahydroalstonine | MOL008457 | 28 |
| 3,9-di-O-methylnissolin | MOL000371 | 23 |
| glycitein | MOL008400 | 23 |
| (6aR,11aR)-9,10-dimethoxy-6a,11a-dihydro-6H-benzofurano[3,2-c]chromen-3-ol | MOL000380 | 22 |
| Calycosin | MOL000417 | 22 |
| Berlambine | MOL002904 | 20 |
| palmatine | MOL000785 | 19 |
| berberine | MOL001454 | 17 |
| 3-beta-Hydroxymethyllenetanshiquinone | MOL007059 | 17 |
| Frutinone A | MOL005321 | 16 |
| Jaranol | MOL000239 | 13 |
| berberrubine | MOL002894 | 13 |
| epiberberine | MOL002897 | 11 |
| Hydroxygenkwanin | MOL005530 | 10 |
| coptisine | MOL001458 | 9 |
| Bifendate | MOL000387 | 7 |
| Worenine | MOL002668 | 7 |
| sitosterol | MOL000359 | 6 |
| Diop | MOL002879 | 6 |
| 1,7-Dihydroxy-3,9-dimethoxy pterocarpene | MOL000442 | 4 |
| Magnograndiolide | MOL000622 | 4 |
| Perlolyrine | MOL002140 | 4 |
| 11-Hydroxyrankinidine | MOL008411 | 4 |
| 9,10-dimethoxypterocarpan-3-O-β-D-glucoside | MOL000379 | 3 |
| FA | MOL000433 | 3 |
| poriferasta-7,22E-dien-3beta-ol | MOL001006 | 3 |
| Mandenol | MOL001494 | 3 |
| ZINC03978781 | MOL003036 | 3 |
| Leucanthoside | MOL003137 | 3 |
| Spinasterol | MOL004355 | 3 |
| Cornudentanone | MOL005503 | 3 |
| (2R)-2-[(3S,5R,10S,13R,14R,16R,17R)-3,16-dihydroxy-4,4,10,13,14-pentamethyl-2,3,5,6,12,15,16,17-octahydro-1H-cyclopenta[a]phenanthren-17-yl]-6-methylhept-5-enoic acid | MOL000273 | 2 |
| Ethyl linolenate | MOL001495 | 2 |
| poriferast-5-en-3beta-ol | MOL001771 | 2 |
| Corchoroside A_qt | MOL002907 | 2 |
| Telocinobufagin | MOL005531 | 2 |
| stigmast-7-enol | MOL006774 | 2 |
| (8S,9S,10R,13R,14S,17R)-17-[(E,2R,5S)-5-ethyl-6-methylhept-3-en-2-yl]-10,13-dimethyl-1,2,4,7,8,9,11,12,14,15,16,17-dodecahydrocyclopenta[a]phenanthren-3-one | MOL008407 | 2 |
| (3S,8S,9S,10R,13R,14S,17R)-10,13-dimethyl-17-[(2R,5S)-5-propan-2-yloctan-2-yl]-2,3,4,7,8,9,11,12,14,15,16,17-dodecahydro-1H-cyclopenta[a]phenanthren-3-ol | MOL000033 | 1 |
| Mairin | MOL000211 | 1 |
| trametenolic acid | MOL000275 | 1 |
| Cerevisterol | MOL000279 | 1 |
| ergosta-7,22E-dien-3beta-ol | MOL000282 | 1 |
| Ergosterol peroxide | MOL000283 | 1 |
| isomucronulatol-7,2'-di-O-glucosiole | MOL000439 | 1 |
| Alisol B monoacetate | MOL000831 | 1 |
| 16β-methoxyalisol B monoacetate | MOL000849 | 1 |
| alisol B | MOL000853 | 1 |
| alisol C monoacetate | MOL000856 | 1 |
| [(1S,3R)-1-[(2R)-3,3-dimethyloxiran-2-yl]-3-[(5R,8S,9S,10S,11S,14R)-11-hydroxy-4,4,8,10,14-pentamethyl-3-oxo-1,2,5,6,7,9,11,12,15,16-decahydrocyclopenta[a]phenanthren-17-yl]butyl] acetate | MOL000862 | 1 |
| 1-Monolinolein | MOL002464 | 1 |
| Ethyl oleate (NF) | MOL002883 | 1 |
| 2,6,10,14,18-pentamethylicosa-2,6,10,14,18-pentaene | MOL005481 | 1 |
| methyl icosa-11,14-dienoate | MOL007514 | 1 |
| 7-(beta-Xylosyl)cephalomannine_qt | MOL008393 | 1 |
| Daturilin | MOL008397 | 1 |

**Supplementary Table S3** – Top 50 genes of six algorithms

| Closeness | Degree | EPC | MNC | Radiality | Stress |
| --- | --- | --- | --- | --- | --- |
| AKT1 | AKT1 | AKT1 | AKT1 | AKT1 | AKT1 |
| IL1B | TP53 | TP53 | TP53 | IL1B | PTGS2 |
| TP53 | IL1B | PTGS2 | IL1B | TP53 | IL1B |
| PTGS2 | PTGS2 | BCL2 | PTGS2 | PTGS2 | ESR1 |
| ESR1 | MMP9 | HIF1A | MMP9 | ESR1 | TP53 |
| CASP3 | ESR1 | IL1B | ESR1 | CASP3 | FOS |
| MMP9 | CASP3 | ESR1 | CASP3 | MMP9 | DRD5 |
| EGFR | EGFR | CASP3 | EGFR | EGFR | MAOA |
| BCL2 | HIF1A | MYC | HIF1A | BCL2 | EGFR |
| HIF1A | BCL2 | EGFR | BCL2 | HIF1A | MMP9 |
| FOS | MYC | MMP9 | MYC | FOS | MAOB |
| MYC | PPARG | IL10 | PPARG | MYC | SLC6A4 |
| PPARG | FOS | FOS | FOS | PPARG | CASP3 |
| GSK3B | CCND1 | CXCL8 | CCND1 | GSK3B | HIF1A |
| CCND1 | EGF | CCND1 | EGF | CCND1 | GSK3B |
| EGF | CCL2 | EGF | CCL2 | EGF | HSPA5 |
| ERBB2 | ERBB2 | NFKBIA | ERBB2 | ERBB2 | DRD1 |
| IL10 | IL10 | IFNG | IL10 | IL10 | PPARG |
| CCL2 | IFNG | ERBB2 | IFNG | CCL2 | MYC |
| IFNG | GSK3B | CCL2 | GSK3B | IFNG | BCL2 |
| CXCL8 | CXCL8 | STAT1 | CXCL8 | CXCL8 | IL10 |
| IL1A | IL1A | PPARG | IL1A | IL1A | CYP3A4 |
| ICAM1 | MMP2 | ICAM1 | MMP2 | ICAM1 | APP |
| RELA | ICAM1 | MMP2 | RELA | RELA | AR |
| NFKBIA | RELA | GSK3B | ICAM1 | NFKBIA | NR3C1 |
| MMP2 | NFKBIA | IL1A | NFKBIA | MMP2 | ESR2 |
| BCL2L1 | STAT1 | BCL2L1 | STAT1 | BCL2L1 | NFE2L2 |
| HMOX1 | BCL2L1 | CDKN1A | BCL2L1 | HMOX1 | RELA |
| STAT1 | CASP8 | CASP9 | CASP8 | STAT1 | ICAM1 |
| CASP8 | CDKN1A | RELA | CDKN1A | CASP8 | EGF |
| CDKN1A | HMOX1 | PARP1 | HMOX1 | CDKN1A | CCL2 |
| NFE2L2 | VCAM1 | CASP8 | VCAM1 | NFE2L2 | PRKCA |
| APP | PARP1 | IL4 | PARP1 | APP | CXCL8 |
| CASP9 | IL4 | MAPK14 | IL4 | CASP9 | OPRM1 |
| IL4 | CASP9 | MAPK1 | CASP9 | IL4 | ERBB2 |
| MAPK1 | NFE2L2 | KDR | NFE2L2 | MAPK1 | CCND1 |
| NR3C1 | MAPK1 | HMOX1 | MAPK1 | NR3C1 | SLC6A3 |
| PARP1 | SERPINE1 | SERPINE1 | SERPINE1 | PARP1 | ADRB2 |
| VCAM1 | MDM2 | MCL1 | MDM2 | VCAM1 | HMOX1 |
| MDM2 | MAPK14 | MDM2 | MAPK14 | MDM2 | GSTP1 |
| MAPK14 | APP | VCAM1 | APP | MAPK14 | IL1A |
| MAPK8 | MAPK8 | IKBKB | MAPK8 | MAPK8 | IFNG |
| KDR | CAV1 | NFE2L2 | CAV1 | KDR | CYP1A1 |
| ESR2 | KDR | MAPK8 | KDR | ESR2 | CASP9 |
| SERPINE1 | CDK4 | CDK4 | CDK4 | SERPINE1 | GJA1 |
| HSPA5 | CDK2 | CAV1 | CDK2 | HSPA5 | CAV1 |
| AR | NR3C1 | APP | NR3C1 | AR | MAPK1 |
| PPARA | MCL1 | CCNB1 | MCL1 | PPARA | PGR |
| PRKCA | AR | HSPA5 | AR | PRKCA | MMP2 |
| CAV1 | HSPA5 | PPARA | PPARA | CAV1 | SERPINE1 |

**Supplementary Table S4** – GO analysis of 32 core genes

| ONTOLOGY | ID | Description | GeneRatio | BgRatio | pvalue | p.adjust | qvalue | geneID | Count |
| --- | --- | --- | --- | --- | --- | --- | --- | --- | --- |
| BP | GO:0006979 | response to oxidative stress | 17/32 | 434/18614 | 5.35E-20 | 8.83E-17 | 2.67E-17 | AKT1/TP53/PTGS2/CASP3/MMP9/EGFR/BCL2/HIF1A/FOS/IL10/IL1A/RELA/MMP2/HMOX1/NFE2L2/APP/MAPK1 | 17 |
| BP | GO:0062197 | cellular response to chemical stress | 16/32 | 349/18614 | 7.58E-20 | 8.83E-17 | 2.67E-17 | AKT1/TP53/PTGS2/CASP3/MMP9/EGFR/BCL2/HIF1A/FOS/IL10/RELA/MMP2/HMOX1/NFE2L2/MAPK1/CAV1 | 16 |
| BP | GO:0000302 | response to reactive oxygen species | 14/32 | 205/18614 | 9.77E-20 | 8.83E-17 | 2.67E-17 | AKT1/CASP3/MMP9/EGFR/BCL2/HIF1A/FOS/IL10/IL1A/RELA/MMP2/HMOX1/NFE2L2/MAPK1 | 14 |
| BP | GO:0010038 | response to metal ion | 16/32 | 367/18614 | 1.70E-19 | 1.15E-16 | 3.48E-17 | AKT1/PTGS2/CASP3/MMP9/EGFR/BCL2/HIF1A/FOS/CCND1/IL1A/HMOX1/NFE2L2/APP/CASP9/MAPK1/CAV1 | 16 |
| BP | GO:2001233 | regulation of apoptotic signaling pathway | 16/32 | 381/18614 | 3.10E-19 | 1.68E-16 | 5.08E-17 | AKT1/IL1B/TP53/PTGS2/MMP9/BCL2/HIF1A/MYC/GSK3B/IL1A/ICAM1/RELA/HMOX1/NFE2L2/SERPINE1/CAV1 | 16 |
| BP | GO:0009416 | response to light stimulus | 15/32 | 323/18614 | 1.22E-18 | 5.52E-16 | 1.67E-16 | AKT1/TP53/PTGS2/CASP3/MMP9/EGFR/BCL2/HIF1A/FOS/MYC/CCND1/RELA/MMP2/APP/CASP9 | 15 |
| BP | GO:0009314 | response to radiation | 16/32 | 452/18614 | 4.73E-18 | 1.83E-15 | 5.54E-16 | AKT1/TP53/PTGS2/CASP3/MMP9/EGFR/BCL2/HIF1A/FOS/MYC/CCND1/IL1A/RELA/MMP2/APP/CASP9 | 16 |
| BP | GO:0009411 | response to UV | 12/32 | 154/18614 | 1.30E-17 | 4.42E-15 | 1.34E-15 | AKT1/TP53/PTGS2/CASP3/MMP9/EGFR/BCL2/MYC/CCND1/RELA/MMP2/CASP9 | 12 |
| BP | GO:0036293 | response to decreased oxygen levels | 14/32 | 306/18614 | 2.83E-17 | 8.52E-15 | 2.58E-15 | AKT1/TP53/PTGS2/CASP3/BCL2/HIF1A/FOS/MYC/PPARG/IL1A/MMP2/HMOX1/NFE2L2/CAV1 | 14 |
| BP | GO:2001234 | negative regulation of apoptotic signaling pathway | 13/32 | 233/18614 | 3.73E-17 | 1.01E-14 | 3.05E-15 | AKT1/IL1B/PTGS2/MMP9/BCL2/HIF1A/GSK3B/IL1A/ICAM1/RELA/HMOX1/NFE2L2/SERPINE1 | 13 |
| BP | GO:0070482 | response to oxygen levels | 14/32 | 333/18614 | 9.24E-17 | 2.28E-14 | 6.89E-15 | AKT1/TP53/PTGS2/CASP3/BCL2/HIF1A/FOS/MYC/PPARG/IL1A/MMP2/HMOX1/NFE2L2/CAV1 | 14 |
| BP | GO:0070997 | neuron death | 14/32 | 373/18614 | 4.49E-16 | 1.02E-13 | 3.07E-14 | AKT1/TP53/CASP3/BCL2/HIF1A/FOS/GSK3B/CCND1/IL10/CCL2/IFNG/HMOX1/APP/CASP9 | 14 |
| BP | GO:0034599 | cellular response to oxidative stress | 13/32 | 290/18614 | 6.49E-16 | 1.35E-13 | 4.10E-14 | AKT1/TP53/MMP9/EGFR/BCL2/HIF1A/FOS/IL10/RELA/MMP2/HMOX1/NFE2L2/MAPK1 | 13 |
| BP | GO:0001666 | response to hypoxia | 13/32 | 293/18614 | 7.42E-16 | 1.44E-13 | 4.35E-14 | TP53/PTGS2/CASP3/BCL2/HIF1A/FOS/MYC/PPARG/IL1A/MMP2/HMOX1/NFE2L2/CAV1 | 13 |
| BP | GO:1902893 | regulation of miRNA transcription | 9/32 | 68/18614 | 1.75E-15 | 3.17E-13 | 9.59E-14 | TP53/ESR1/EGFR/HIF1A/FOS/MYC/PPARG/IL10/RELA | 9 |
| BP | GO:0061614 | miRNA transcription | 9/32 | 69/18614 | 2.02E-15 | 3.42E-13 | 1.03E-13 | TP53/ESR1/EGFR/HIF1A/FOS/MYC/PPARG/IL10/RELA | 9 |
| BP | GO:1901214 | regulation of neuron death | 13/32 | 327/18614 | 3.08E-15 | 4.91E-13 | 1.49E-13 | AKT1/TP53/CASP3/BCL2/HIF1A/FOS/GSK3B/CCND1/IL10/CCL2/IFNG/HMOX1/CASP9 | 13 |
| BP | GO:0071453 | cellular response to oxygen levels | 11/32 | 172/18614 | 3.31E-15 | 4.99E-13 | 1.51E-13 | AKT1/TP53/PTGS2/BCL2/HIF1A/FOS/MYC/PPARG/HMOX1/NFE2L2/CAV1 | 11 |
| BP | GO:0032496 | response to lipopolysaccharide | 13/32 | 345/18614 | 6.15E-15 | 8.78E-13 | 2.66E-13 | AKT1/IL1B/PTGS2/CASP3/FOS/IL10/CCL2/CXCL8/IL1A/RELA/CASP9/MAPK1/SERPINE1 | 13 |
| BP | GO:2000628 | regulation of miRNA metabolic process | 9/32 | 81/18614 | 9.15E-15 | 1.24E-12 | 3.75E-13 | TP53/ESR1/EGFR/HIF1A/FOS/MYC/PPARG/IL10/RELA | 9 |
| BP | GO:0002237 | response to molecule of bacterial origin | 13/32 | 366/18614 | 1.32E-14 | 1.70E-12 | 5.14E-13 | AKT1/IL1B/PTGS2/CASP3/FOS/IL10/CCL2/CXCL8/IL1A/RELA/CASP9/MAPK1/SERPINE1 | 13 |
| BP | GO:1902895 | positive regulation of miRNA transcription | 8/32 | 51/18614 | 1.79E-14 | 2.17E-12 | 6.58E-13 | TP53/EGFR/HIF1A/FOS/MYC/PPARG/IL10/RELA | 8 |
| BP | GO:0050673 | epithelial cell proliferation | 14/32 | 488/18614 | 1.84E-14 | 2.17E-12 | 6.58E-13 | AKT1/ESR1/EGFR/HIF1A/MYC/PPARG/CCND1/EGF/ERBB2/IL10/CCL2/HMOX1/MAPK1/CAV1 | 14 |
| BP | GO:1904019 | epithelial cell apoptotic process | 10/32 | 140/18614 | 2.34E-14 | 2.65E-12 | 8.01E-13 | ESR1/CASP3/BCL2/IL10/CCL2/ICAM1/HMOX1/NFE2L2/CASP9/SERPINE1 | 10 |
| BP | GO:0097193 | intrinsic apoptotic signaling pathway | 12/32 | 301/18614 | 4.35E-14 | 4.71E-12 | 1.43E-12 | AKT1/TP53/PTGS2/CASP3/MMP9/BCL2/HIF1A/MYC/HMOX1/NFE2L2/CASP9/CAV1 | 12 |
| BP | GO:0010586 | miRNA metabolic process | 9/32 | 97/18614 | 4.93E-14 | 5.14E-12 | 1.55E-12 | TP53/ESR1/EGFR/HIF1A/FOS/MYC/PPARG/IL10/RELA | 9 |
| BP | GO:2001237 | negative regulation of extrinsic apoptotic signaling pathway | 9/32 | 99/18614 | 5.95E-14 | 5.98E-12 | 1.81E-12 | AKT1/IL1B/BCL2/GSK3B/IL1A/ICAM1/RELA/HMOX1/SERPINE1 | 9 |
| BP | GO:2001236 | regulation of extrinsic apoptotic signaling pathway | 10/32 | 156/18614 | 7.03E-14 | 6.48E-12 | 1.96E-12 | AKT1/IL1B/BCL2/GSK3B/IL1A/ICAM1/RELA/HMOX1/SERPINE1/CAV1 | 10 |
| BP | GO:2000630 | positive regulation of miRNA metabolic process | 8/32 | 60/18614 | 7.10E-14 | 6.48E-12 | 1.96E-12 | TP53/EGFR/HIF1A/FOS/MYC/PPARG/IL10/RELA | 8 |
| BP | GO:0097191 | extrinsic apoptotic signaling pathway | 11/32 | 227/18614 | 7.17E-14 | 6.48E-12 | 1.96E-12 | AKT1/IL1B/BCL2/GSK3B/IFNG/IL1A/ICAM1/RELA/HMOX1/SERPINE1/CAV1 | 11 |
| BP | GO:0036294 | cellular response to decreased oxygen levels | 10/32 | 157/18614 | 7.51E-14 | 6.57E-12 | 1.99E-12 | AKT1/TP53/PTGS2/BCL2/HIF1A/FOS/MYC/PPARG/HMOX1/NFE2L2 | 10 |
| BP | GO:0048732 | gland development | 13/32 | 441/18614 | 1.43E-13 | 1.22E-11 | 3.68E-12 | AKT1/ESR1/EGFR/BCL2/HIF1A/CCND1/EGF/IL10/RELA/MMP2/HMOX1/MAPK1/CAV1 | 13 |
| BP | GO:0071216 | cellular response to biotic stimulus | 11/32 | 264/18614 | 3.76E-13 | 3.09E-11 | 9.35E-12 | AKT1/IL1B/TP53/GSK3B/IL10/CCL2/CXCL8/IL1A/RELA/MAPK1/SERPINE1 | 11 |
| BP | GO:1901653 | cellular response to peptide | 12/32 | 375/18614 | 5.90E-13 | 4.70E-11 | 1.42E-11 | AKT1/IL1B/TP53/FOS/PPARG/GSK3B/ICAM1/RELA/NFE2L2/APP/MAPK1/CAV1 | 12 |
| BP | GO:0050900 | leukocyte migration | 12/32 | 393/18614 | 1.02E-12 | 7.94E-11 | 2.40E-11 | AKT1/IL1B/IL10/CCL2/CXCL8/IL1A/ICAM1/MMP2/HMOX1/APP/MAPK1/SERPINE1 | 12 |
| BP | GO:0050678 | regulation of epithelial cell proliferation | 12/32 | 419/18614 | 2.18E-12 | 1.64E-10 | 4.96E-11 | AKT1/EGFR/HIF1A/MYC/PPARG/CCND1/EGF/ERBB2/IL10/CCL2/HMOX1/CAV1 | 12 |
| BP | GO:0071456 | cellular response to hypoxia | 9/32 | 149/18614 | 2.54E-12 | 1.86E-10 | 5.62E-11 | TP53/PTGS2/BCL2/HIF1A/FOS/MYC/PPARG/HMOX1/NFE2L2 | 9 |
| BP | GO:0034614 | cellular response to reactive oxygen species | 9/32 | 152/18614 | 3.04E-12 | 2.17E-10 | 6.56E-11 | AKT1/MMP9/EGFR/FOS/IL10/RELA/MMP2/NFE2L2/MAPK1 | 9 |
| BP | GO:0009410 | response to xenobiotic stimulus | 12/32 | 440/18614 | 3.86E-12 | 2.68E-10 | 8.12E-11 | IL1B/TP53/PTGS2/CASP3/BCL2/FOS/MYC/CCND1/IL10/MMP2/HMOX1/NFE2L2 | 12 |
| BP | GO:0032768 | regulation of monooxygenase activity | 7/32 | 57/18614 | 5.47E-12 | 3.71E-10 | 1.12E-10 | AKT1/IL1B/EGFR/HIF1A/IFNG/IL1A/CAV1 | 7 |
| BP | GO:2001242 | regulation of intrinsic apoptotic signaling pathway | 9/32 | 173/18614 | 9.80E-12 | 6.48E-10 | 1.96E-10 | AKT1/TP53/PTGS2/MMP9/BCL2/HIF1A/MYC/NFE2L2/CAV1 | 9 |
| BP | GO:0034612 | response to tumor necrosis factor | 10/32 | 257/18614 | 1.04E-11 | 6.74E-10 | 2.04E-10 | AKT1/TP53/PTGS2/CASP3/FOS/CCL2/CXCL8/RELA/NFE2L2/MAPK1 | 10 |
| BP | GO:0071276 | cellular response to cadmium ion | 6/32 | 31/18614 | 1.12E-11 | 6.95E-10 | 2.10E-10 | AKT1/MMP9/EGFR/FOS/HMOX1/MAPK1 | 6 |
| BP | GO:0051402 | neuron apoptotic process | 10/32 | 259/18614 | 1.13E-11 | 6.95E-10 | 2.10E-10 | TP53/CASP3/BCL2/HIF1A/CCND1/IL10/CCL2/HMOX1/APP/CASP9 | 10 |
| BP | GO:0070141 | response to UV-A | 5/32 | 14/18614 | 2.14E-11 | 1.28E-09 | 3.88E-10 | AKT1/MMP9/EGFR/CCND1/MMP2 | 5 |
| BP | GO:0032355 | response to estradiol | 8/32 | 120/18614 | 2.18E-11 | 1.28E-09 | 3.88E-10 | PTGS2/ESR1/CASP3/EGFR/CCND1/IL10/MMP2/CASP9 | 8 |
| BP | GO:0071248 | cellular response to metal ion | 9/32 | 200/18614 | 3.61E-11 | 2.08E-09 | 6.30E-10 | AKT1/PTGS2/MMP9/EGFR/FOS/HMOX1/NFE2L2/APP/MAPK1 | 9 |
| BP | GO:0007159 | leukocyte cell-cell adhesion | 11/32 | 415/18614 | 5.02E-11 | 2.84E-09 | 8.58E-10 | AKT1/IL1B/CASP3/ERBB2/IL10/CCL2/IFNG/IL1A/ICAM1/RELA/CAV1 | 11 |
| BP | GO:0043434 | response to peptide hormone | 11/32 | 427/18614 | 6.81E-11 | 3.77E-09 | 1.14E-09 | AKT1/IL1B/PTGS2/FOS/PPARG/GSK3B/IL10/RELA/NFE2L2/MAPK1/CAV1 | 11 |
| BP | GO:0050999 | regulation of nitric-oxide synthase activity | 6/32 | 42/18614 | 7.89E-11 | 4.28E-09 | 1.29E-09 | AKT1/IL1B/EGFR/HIF1A/IL1A/CAV1 | 6 |
| BP | GO:0043523 | regulation of neuron apoptotic process | 9/32 | 219/18614 | 8.13E-11 | 4.32E-09 | 1.31E-09 | TP53/CASP3/BCL2/HIF1A/CCND1/IL10/CCL2/HMOX1/CASP9 | 9 |
| BP | GO:0071222 | cellular response to lipopolysaccharide | 9/32 | 224/18614 | 9.94E-11 | 5.18E-09 | 1.57E-09 | AKT1/IL1B/IL10/CCL2/CXCL8/IL1A/RELA/MAPK1/SERPINE1 | 9 |
| BP | GO:0050679 | positive regulation of epithelial cell proliferation | 9/32 | 225/18614 | 1.03E-10 | 5.29E-09 | 1.60E-09 | AKT1/EGFR/HIF1A/MYC/CCND1/EGF/ERBB2/IL10/HMOX1 | 9 |
| BP | GO:0002685 | regulation of leukocyte migration | 9/32 | 227/18614 | 1.12E-10 | 5.62E-09 | 1.70E-09 | AKT1/CCL2/CXCL8/IL1A/ICAM1/HMOX1/APP/MAPK1/SERPINE1 | 9 |
| BP | GO:0071241 | cellular response to inorganic substance | 9/32 | 231/18614 | 1.31E-10 | 6.45E-09 | 1.95E-09 | AKT1/PTGS2/MMP9/EGFR/FOS/HMOX1/NFE2L2/APP/MAPK1 | 9 |
| BP | GO:0071219 | cellular response to molecule of bacterial origin | 9/32 | 237/18614 | 1.64E-10 | 7.95E-09 | 2.40E-09 | AKT1/IL1B/IL10/CCL2/CXCL8/IL1A/RELA/MAPK1/SERPINE1 | 9 |
| BP | GO:0070371 | ERK1 and ERK2 cascade | 10/32 | 341/18614 | 1.68E-10 | 8.01E-09 | 2.42E-09 | IL1B/EGFR/MYC/EGF/ERBB2/CCL2/IL1A/ICAM1/APP/MAPK1 | 10 |
| BP | GO:1903829 | positive regulation of protein localization | 11/32 | 468/18614 | 1.81E-10 | 8.42E-09 | 2.55E-09 | AKT1/IL1B/PTGS2/EGFR/HIF1A/PPARG/GSK3B/EGF/ERBB2/IFNG/IL1A | 11 |
| BP | GO:0035094 | response to nicotine | 6/32 | 48/18614 | 1.83E-10 | 8.42E-09 | 2.55E-09 | CASP3/BCL2/RELA/MMP2/HMOX1/MAPK1 | 6 |
| BP | GO:0045765 | regulation of angiogenesis | 10/32 | 349/18614 | 2.11E-10 | 9.54E-09 | 2.89E-09 | IL1B/HIF1A/PPARG/ERBB2/IL10/CXCL8/IL1A/HMOX1/NFE2L2/SERPINE1 | 10 |
| BP | GO:0031667 | response to nutrient levels | 11/32 | 477/18614 | 2.21E-10 | 9.85E-09 | 2.98E-09 | AKT1/TP53/PTGS2/BCL2/FOS/PPARG/CCND1/IL1A/HMOX1/NFE2L2/MAPK1 | 11 |
| BP | GO:1901342 | regulation of vasculature development | 10/32 | 355/18614 | 2.49E-10 | 1.09E-08 | 3.30E-09 | IL1B/HIF1A/PPARG/ERBB2/IL10/CXCL8/IL1A/HMOX1/NFE2L2/SERPINE1 | 10 |
| BP | GO:0033002 | muscle cell proliferation | 9/32 | 249/18614 | 2.55E-10 | 1.10E-08 | 3.31E-09 | AKT1/PTGS2/MMP9/FOS/PPARG/IL10/IFNG/MMP2/HMOX1 | 9 |
| BP | GO:0019221 | cytokine-mediated signaling pathway | 11/32 | 492/18614 | 3.08E-10 | 1.30E-08 | 3.94E-09 | AKT1/IL1B/TP53/HIF1A/PPARG/CCL2/IFNG/CXCL8/IL1A/RELA/CAV1 | 11 |
| BP | GO:0045862 | positive regulation of proteolysis | 10/32 | 365/18614 | 3.27E-10 | 1.36E-08 | 4.12E-09 | AKT1/IL1B/MYC/PPARG/GSK3B/EGF/IFNG/NFE2L2/CASP9/CAV1 | 10 |
| BP | GO:0051098 | regulation of binding | 10/32 | 369/18614 | 3.63E-10 | 1.47E-08 | 4.45E-09 | AKT1/MMP9/PPARG/GSK3B/EGF/IL10/IFNG/HMOX1/APP/CAV1 | 10 |
| BP | GO:0071900 | regulation of protein serine/threonine kinase activity | 10/32 | 369/18614 | 3.63E-10 | 1.47E-08 | 4.45E-09 | AKT1/IL1B/CASP3/EGFR/PPARG/CCND1/EGF/ERBB2/IFNG/CAV1 | 10 |
| BP | GO:0046686 | response to cadmium ion | 6/32 | 54/18614 | 3.83E-10 | 1.53E-08 | 4.62E-09 | AKT1/MMP9/EGFR/FOS/HMOX1/MAPK1 | 6 |
| BP | GO:1903037 | regulation of leukocyte cell-cell adhesion | 10/32 | 378/18614 | 4.59E-10 | 1.80E-08 | 5.45E-09 | AKT1/IL1B/CASP3/ERBB2/IL10/CCL2/IFNG/IL1A/RELA/CAV1 | 10 |
| BP | GO:0010634 | positive regulation of epithelial cell migration | 8/32 | 176/18614 | 4.72E-10 | 1.80E-08 | 5.45E-09 | AKT1/PTGS2/MMP9/HIF1A/EGF/IFNG/HMOX1/NFE2L2 | 8 |
| BP | GO:0048660 | regulation of smooth muscle cell proliferation | 8/32 | 176/18614 | 4.72E-10 | 1.80E-08 | 5.45E-09 | AKT1/PTGS2/MMP9/PPARG/IL10/IFNG/MMP2/HMOX1 | 8 |
| BP | GO:0010469 | regulation of signaling receptor activity | 8/32 | 177/18614 | 4.94E-10 | 1.86E-08 | 5.62E-09 | HIF1A/PPARG/EGF/IL10/CCL2/IFNG/APP/SERPINE1 | 8 |
| BP | GO:0033138 | positive regulation of peptidyl-serine phosphorylation | 7/32 | 108/18614 | 5.43E-10 | 2.02E-08 | 6.10E-09 | AKT1/PTGS2/EGFR/BCL2/IFNG/APP/CAV1 | 7 |
| BP | GO:0048659 | smooth muscle cell proliferation | 8/32 | 180/18614 | 5.64E-10 | 2.07E-08 | 6.25E-09 | AKT1/PTGS2/MMP9/PPARG/IL10/IFNG/MMP2/HMOX1 | 8 |
| BP | GO:1904035 | regulation of epithelial cell apoptotic process | 7/32 | 109/18614 | 5.80E-10 | 2.10E-08 | 6.34E-09 | ESR1/BCL2/CCL2/ICAM1/HMOX1/NFE2L2/SERPINE1 | 7 |
| BP | GO:0051341 | regulation of oxidoreductase activity | 7/32 | 111/18614 | 6.59E-10 | 2.35E-08 | 7.11E-09 | AKT1/IL1B/EGFR/HIF1A/IFNG/IL1A/CAV1 | 7 |
| BP | GO:0001936 | regulation of endothelial cell proliferation | 8/32 | 185/18614 | 7.02E-10 | 2.41E-08 | 7.28E-09 | AKT1/HIF1A/PPARG/EGF/IL10/CCL2/HMOX1/CAV1 | 8 |
| BP | GO:0045766 | positive regulation of angiogenesis | 8/32 | 185/18614 | 7.02E-10 | 2.41E-08 | 7.28E-09 | IL1B/HIF1A/IL10/CXCL8/IL1A/HMOX1/NFE2L2/SERPINE1 | 8 |
| BP | GO:1904018 | positive regulation of vasculature development | 8/32 | 185/18614 | 7.02E-10 | 2.41E-08 | 7.28E-09 | IL1B/HIF1A/IL10/CXCL8/IL1A/HMOX1/NFE2L2/SERPINE1 | 8 |
| BP | GO:0007565 | female pregnancy | 8/32 | 187/18614 | 7.64E-10 | 2.59E-08 | 7.83E-09 | AKT1/IL1B/PTGS2/ESR1/MMP9/BCL2/FOS/MMP2 | 8 |
| BP | GO:0045428 | regulation of nitric oxide biosynthetic process | 6/32 | 62/18614 | 9.02E-10 | 3.02E-08 | 9.14E-09 | AKT1/IL1B/PTGS2/IL10/IFNG/CAV1 | 6 |
| BP | GO:0071887 | leukocyte apoptotic process | 7/32 | 117/18614 | 9.56E-10 | 3.16E-08 | 9.56E-09 | AKT1/TP53/CASP3/BCL2/HIF1A/IL10/CASP9 | 7 |
| BP | GO:0050727 | regulation of inflammatory response | 10/32 | 414/18614 | 1.11E-09 | 3.62E-08 | 1.09E-08 | IL1B/PTGS2/ESR1/MMP9/PPARG/IL10/IFNG/RELA/APP/SERPINE1 | 10 |
| BP | GO:0010632 | regulation of epithelial cell migration | 9/32 | 295/18614 | 1.14E-09 | 3.67E-08 | 1.11E-08 | AKT1/PTGS2/MMP9/HIF1A/PPARG/EGF/IFNG/HMOX1/NFE2L2 | 9 |
| BP | GO:0080164 | regulation of nitric oxide metabolic process | 6/32 | 65/18614 | 1.21E-09 | 3.85E-08 | 1.17E-08 | AKT1/IL1B/PTGS2/IL10/IFNG/CAV1 | 6 |
| BP | GO:0001935 | endothelial cell proliferation | 8/32 | 201/18614 | 1.35E-09 | 4.27E-08 | 1.29E-08 | AKT1/HIF1A/PPARG/EGF/IL10/CCL2/HMOX1/CAV1 | 8 |
| BP | GO:0071375 | cellular response to peptide hormone stimulus | 9/32 | 308/18614 | 1.66E-09 | 5.18E-08 | 1.57E-08 | AKT1/IL1B/FOS/PPARG/GSK3B/RELA/NFE2L2/MAPK1/CAV1 | 9 |
| BP | GO:0044703 | multi-organism reproductive process | 8/32 | 207/18614 | 1.71E-09 | 5.21E-08 | 1.58E-08 | AKT1/IL1B/PTGS2/ESR1/MMP9/BCL2/FOS/MMP2 | 8 |
| BP | GO:0018105 | peptidyl-serine phosphorylation | 9/32 | 309/18614 | 1.71E-09 | 5.21E-08 | 1.58E-08 | AKT1/PTGS2/EGFR/BCL2/GSK3B/IFNG/APP/MAPK1/CAV1 | 9 |
| BP | GO:0042542 | response to hydrogen peroxide | 7/32 | 129/18614 | 1.90E-09 | 5.72E-08 | 1.73E-08 | CASP3/BCL2/IL10/RELA/MMP2/HMOX1/NFE2L2 | 7 |
| BP | GO:0044706 | multi-multicellular organism process | 8/32 | 216/18614 | 2.39E-09 | 7.13E-08 | 2.15E-08 | AKT1/IL1B/PTGS2/ESR1/MMP9/BCL2/FOS/MMP2 | 8 |
| BP | GO:0031331 | positive regulation of cellular catabolic process | 10/32 | 450/18614 | 2.47E-09 | 7.29E-08 | 2.20E-08 | AKT1/IL1B/HIF1A/GSK3B/EGF/IFNG/HMOX1/NFE2L2/APP/CAV1 | 10 |
| BP | GO:1901215 | negative regulation of neuron death | 8/32 | 219/18614 | 2.67E-09 | 7.77E-08 | 2.35E-08 | AKT1/BCL2/HIF1A/GSK3B/CCND1/IL10/CCL2/HMOX1 | 8 |
| BP | GO:0030879 | mammary gland development | 7/32 | 136/18614 | 2.75E-09 | 7.93E-08 | 2.40E-08 | AKT1/ESR1/HIF1A/CCND1/EGF/MAPK1/CAV1 | 7 |
| BP | GO:0018209 | peptidyl-serine modification | 9/32 | 327/18614 | 2.81E-09 | 8.01E-08 | 2.42E-08 | AKT1/PTGS2/EGFR/BCL2/GSK3B/IFNG/APP/MAPK1/CAV1 | 9 |
| BP | GO:0006809 | nitric oxide biosynthetic process | 6/32 | 77/18614 | 3.42E-09 | 9.66E-08 | 2.92E-08 | AKT1/IL1B/PTGS2/IL10/IFNG/CAV1 | 6 |
| BP | GO:0070555 | response to interleukin-1 | 7/32 | 141/18614 | 3.54E-09 | 9.90E-08 | 2.99E-08 | IL1B/HIF1A/CCL2/CXCL8/RELA/MMP2/APP | 7 |
| BP | GO:0051384 | response to glucocorticoid | 7/32 | 142/18614 | 3.72E-09 | 1.03E-07 | 3.11E-08 | PTGS2/CASP3/BCL2/FOS/CCND1/IL10/CASP9 | 7 |
| BP | GO:0048545 | response to steroid hormone | 9/32 | 339/18614 | 3.85E-09 | 1.05E-07 | 3.19E-08 | PTGS2/ESR1/CASP3/BCL2/FOS/CCND1/IL10/CASP9/CAV1 | 9 |
| BP | GO:0033135 | regulation of peptidyl-serine phosphorylation | 7/32 | 143/18614 | 3.91E-09 | 1.06E-07 | 3.20E-08 | AKT1/PTGS2/EGFR/BCL2/IFNG/APP/CAV1 | 7 |
| BP | GO:1903131 | mononuclear cell differentiation | 10/32 | 474/18614 | 4.07E-09 | 1.09E-07 | 3.31E-08 | IL1B/TP53/BCL2/FOS/MYC/PPARG/ERBB2/IL10/IFNG/IL1A | 10 |
| BP | GO:0071356 | cellular response to tumor necrosis factor | 8/32 | 235/18614 | 4.64E-09 | 1.23E-07 | 3.73E-08 | AKT1/TP53/FOS/CCL2/CXCL8/RELA/NFE2L2/MAPK1 | 8 |
| BP | GO:0045785 | positive regulation of cell adhesion | 10/32 | 482/18614 | 4.78E-09 | 1.26E-07 | 3.80E-08 | AKT1/IL1B/GSK3B/ERBB2/IL10/CCL2/IFNG/IL1A/RELA/CAV1 | 10 |
| BP | GO:0010506 | regulation of autophagy | 9/32 | 348/18614 | 4.83E-09 | 1.26E-07 | 3.81E-08 | AKT1/TP53/CASP3/BCL2/HIF1A/GSK3B/IL10/IFNG/HMOX1 | 9 |
| BP | GO:0046209 | nitric oxide metabolic process | 6/32 | 83/18614 | 5.40E-09 | 1.40E-07 | 4.22E-08 | AKT1/IL1B/PTGS2/IL10/IFNG/CAV1 | 6 |
| BP | GO:0001819 | positive regulation of cytokine production | 10/32 | 489/18614 | 5.49E-09 | 1.40E-07 | 4.24E-08 | IL1B/PTGS2/HIF1A/IL10/IFNG/IL1A/RELA/HMOX1/APP/SERPINE1 | 10 |
| BP | GO:0022407 | regulation of cell-cell adhesion | 10/32 | 491/18614 | 5.70E-09 | 1.45E-07 | 4.37E-08 | AKT1/IL1B/CASP3/ERBB2/IL10/CCL2/IFNG/IL1A/RELA/CAV1 | 10 |
| BP | GO:2001057 | reactive nitrogen species metabolic process | 6/32 | 84/18614 | 5.81E-09 | 1.46E-07 | 4.41E-08 | AKT1/IL1B/PTGS2/IL10/IFNG/CAV1 | 6 |
| BP | GO:0001890 | placenta development | 7/32 | 152/18614 | 5.98E-09 | 1.49E-07 | 4.50E-08 | AKT1/PTGS2/EGFR/HIF1A/PPARG/IL10/MAPK1 | 7 |
| BP | GO:0042176 | regulation of protein catabolic process | 9/32 | 361/18614 | 6.65E-09 | 1.64E-07 | 4.96E-08 | AKT1/IL1B/EGFR/GSK3B/IL10/IFNG/RELA/NFE2L2/CAV1 | 9 |
| BP | GO:0010631 | epithelial cell migration | 9/32 | 371/18614 | 8.43E-09 | 2.06E-07 | 6.23E-08 | AKT1/PTGS2/MMP9/HIF1A/PPARG/EGF/IFNG/HMOX1/NFE2L2 | 9 |
| BP | GO:0090132 | epithelium migration | 9/32 | 374/18614 | 9.04E-09 | 2.19E-07 | 6.62E-08 | AKT1/PTGS2/MMP9/HIF1A/PPARG/EGF/IFNG/HMOX1/NFE2L2 | 9 |
| BP | GO:0050863 | regulation of T cell activation | 9/32 | 377/18614 | 9.69E-09 | 2.33E-07 | 7.03E-08 | AKT1/IL1B/CASP3/ERBB2/IL10/CCL2/IFNG/IL1A/CAV1 | 9 |
| BP | GO:1901216 | positive regulation of neuron death | 6/32 | 92/18614 | 1.01E-08 | 2.39E-07 | 7.23E-08 | TP53/CASP3/FOS/GSK3B/IFNG/CASP9 | 6 |
| BP | GO:0090130 | tissue migration | 9/32 | 379/18614 | 1.01E-08 | 2.39E-07 | 7.23E-08 | AKT1/PTGS2/MMP9/HIF1A/PPARG/EGF/IFNG/HMOX1/NFE2L2 | 9 |
| BP | GO:0034644 | cellular response to UV | 6/32 | 93/18614 | 1.08E-08 | 2.52E-07 | 7.62E-08 | TP53/PTGS2/MMP9/MYC/MMP2/CASP9 | 6 |
| BP | GO:0030856 | regulation of epithelial cell differentiation | 7/32 | 166/18614 | 1.10E-08 | 2.53E-07 | 7.66E-08 | IL1B/MMP9/CCND1/IFNG/IL1A/SERPINE1/CAV1 | 7 |
| BP | GO:0031960 | response to corticosteroid | 7/32 | 166/18614 | 1.10E-08 | 2.53E-07 | 7.66E-08 | PTGS2/CASP3/BCL2/FOS/CCND1/IL10/CASP9 | 7 |
| BP | GO:0043620 | regulation of DNA-templated transcription in response to stress | 5/32 | 44/18614 | 1.12E-08 | 2.55E-07 | 7.72E-08 | TP53/HIF1A/RELA/HMOX1/NFE2L2 | 5 |
| BP | GO:0034349 | glial cell apoptotic process | 4/32 | 16/18614 | 1.29E-08 | 2.92E-07 | 8.82E-08 | TP53/CASP3/CCL2/CASP9 | 4 |
| BP | GO:2000273 | positive regulation of signaling receptor activity | 5/32 | 46/18614 | 1.41E-08 | 3.15E-07 | 9.51E-08 | HIF1A/EGF/IL10/CCL2/IFNG | 5 |
| BP | GO:0051091 | positive regulation of DNA-binding transcription factor activity | 8/32 | 271/18614 | 1.41E-08 | 3.15E-07 | 9.51E-08 | AKT1/IL1B/ESR1/PPARG/IL10/RELA/APP/CAV1 | 8 |
| BP | GO:0032642 | regulation of chemokine production | 6/32 | 98/18614 | 1.48E-08 | 3.26E-07 | 9.87E-08 | IL1B/HIF1A/IL10/IFNG/HMOX1/APP | 6 |
| BP | GO:0032602 | chemokine production | 6/32 | 99/18614 | 1.57E-08 | 3.37E-07 | 1.02E-07 | IL1B/HIF1A/IL10/IFNG/HMOX1/APP | 6 |
| BP | GO:0048661 | positive regulation of smooth muscle cell proliferation | 6/32 | 99/18614 | 1.57E-08 | 3.37E-07 | 1.02E-07 | AKT1/PTGS2/MMP9/IL10/MMP2/HMOX1 | 6 |
| BP | GO:2001243 | negative regulation of intrinsic apoptotic signaling pathway | 6/32 | 99/18614 | 1.57E-08 | 3.37E-07 | 1.02E-07 | AKT1/PTGS2/MMP9/BCL2/HIF1A/NFE2L2 | 6 |
| BP | GO:0070849 | response to epidermal growth factor | 5/32 | 47/18614 | 1.58E-08 | 3.37E-07 | 1.02E-07 | AKT1/EGFR/FOS/ERBB2/MAPK1 | 5 |
| BP | GO:0051099 | positive regulation of binding | 7/32 | 176/18614 | 1.65E-08 | 3.50E-07 | 1.06E-07 | MMP9/PPARG/GSK3B/EGF/IFNG/APP/CAV1 | 7 |
| BP | GO:0051348 | negative regulation of transferase activity | 8/32 | 279/18614 | 1.77E-08 | 3.73E-07 | 1.13E-07 | AKT1/IL1B/TP53/CASP3/PPARG/GSK3B/IFNG/CAV1 | 8 |
| BP | GO:0090594 | inflammatory response to wounding | 4/32 | 18/18614 | 2.16E-08 | 4.48E-07 | 1.35E-07 | HIF1A/PPARG/IL1A/HMOX1 | 4 |
| BP | GO:0006109 | regulation of carbohydrate metabolic process | 7/32 | 183/18614 | 2.16E-08 | 4.48E-07 | 1.35E-07 | AKT1/TP53/HIF1A/GSK3B/EGF/IFNG/APP | 7 |
| BP | GO:0030099 | myeloid cell differentiation | 9/32 | 421/18614 | 2.52E-08 | 5.17E-07 | 1.56E-07 | CASP3/MMP9/HIF1A/FOS/MYC/PPARG/IFNG/APP/CASP9 | 9 |
| BP | GO:0052547 | regulation of peptidase activity | 9/32 | 425/18614 | 2.73E-08 | 5.54E-07 | 1.68E-07 | AKT1/PTGS2/MMP9/MYC/PPARG/APP/CASP9/SERPINE1/CAV1 | 9 |
| BP | GO:1901522 | positive regulation of transcription from RNA polymerase II promoter involved in cellular response to chemical stimulus | 4/32 | 19/18614 | 2.74E-08 | 5.54E-07 | 1.68E-07 | TP53/HIF1A/RELA/NFE2L2 | 4 |
| BP | GO:0018107 | peptidyl-threonine phosphorylation | 6/32 | 109/18614 | 2.81E-08 | 5.64E-07 | 1.71E-07 | AKT1/BCL2/GSK3B/EGF/APP/MAPK1 | 6 |
| BP | GO:0001503 | ossification | 9/32 | 429/18614 | 2.96E-08 | 5.90E-07 | 1.78E-07 | AKT1/PTGS2/EGFR/BCL2/HIF1A/PPARG/GSK3B/MMP2/MAPK1 | 9 |
| BP | GO:0071347 | cellular response to interleukin-1 | 6/32 | 111/18614 | 3.13E-08 | 6.20E-07 | 1.88E-07 | IL1B/HIF1A/CCL2/CXCL8/RELA/MMP2 | 6 |
| BP | GO:0030857 | negative regulation of epithelial cell differentiation | 5/32 | 54/18614 | 3.22E-08 | 6.29E-07 | 1.90E-07 | MMP9/CCND1/IFNG/IL1A/CAV1 | 5 |
| BP | GO:1904645 | response to amyloid-beta | 5/32 | 54/18614 | 3.22E-08 | 6.29E-07 | 1.90E-07 | MMP9/GSK3B/ICAM1/MMP2/APP | 5 |
| BP | GO:0071902 | positive regulation of protein serine/threonine kinase activity | 7/32 | 195/18614 | 3.35E-08 | 6.49E-07 | 1.96E-07 | AKT1/IL1B/EGFR/CCND1/EGF/ERBB2/IFNG | 7 |
| BP | GO:0060749 | mammary gland alveolus development | 4/32 | 20/18614 | 3.42E-08 | 6.53E-07 | 1.97E-07 | ESR1/HIF1A/CCND1/EGF | 4 |
| BP | GO:0061377 | mammary gland lobule development | 4/32 | 20/18614 | 3.42E-08 | 6.53E-07 | 1.97E-07 | ESR1/HIF1A/CCND1/EGF | 4 |
| BP | GO:0042060 | wound healing | 9/32 | 439/18614 | 3.61E-08 | 6.84E-07 | 2.07E-07 | CASP3/HIF1A/PPARG/ERBB2/IL1A/HMOX1/NFE2L2/SERPINE1/CAV1 | 9 |
| BP | GO:0051222 | positive regulation of protein transport | 8/32 | 309/18614 | 3.92E-08 | 7.38E-07 | 2.23E-07 | IL1B/PTGS2/HIF1A/PPARG/GSK3B/ERBB2/IFNG/IL1A | 8 |
| BP | GO:0018210 | peptidyl-threonine modification | 6/32 | 119/18614 | 4.76E-08 | 8.90E-07 | 2.69E-07 | AKT1/BCL2/GSK3B/EGF/APP/MAPK1 | 6 |
| BP | GO:0042063 | gliogenesis | 8/32 | 320/18614 | 5.13E-08 | 9.54E-07 | 2.88E-07 | AKT1/IL1B/TP53/ERBB2/CCL2/IFNG/APP/MAPK1 | 8 |
| BP | GO:0022409 | positive regulation of cell-cell adhesion | 8/32 | 321/18614 | 5.26E-08 | 9.70E-07 | 2.93E-07 | AKT1/IL1B/IL10/CCL2/IFNG/IL1A/RELA/CAV1 | 8 |
| BP | GO:0002064 | epithelial cell development | 7/32 | 210/18614 | 5.58E-08 | 1.02E-06 | 3.09E-07 | AKT1/IL1B/ESR1/HIF1A/GSK3B/IL1A/ICAM1 | 7 |
| BP | GO:1904951 | positive regulation of establishment of protein localization | 8/32 | 325/18614 | 5.79E-08 | 1.05E-06 | 3.18E-07 | IL1B/PTGS2/HIF1A/PPARG/GSK3B/ERBB2/IFNG/IL1A | 8 |
| BP | GO:0051090 | regulation of DNA-binding transcription factor activity | 9/32 | 465/18614 | 5.90E-08 | 1.07E-06 | 3.23E-07 | AKT1/IL1B/ESR1/PPARG/IL10/RELA/HMOX1/APP/CAV1 | 9 |
| BP | GO:0051251 | positive regulation of lymphocyte activation | 8/32 | 327/18614 | 6.07E-08 | 1.08E-06 | 3.27E-07 | AKT1/IL1B/BCL2/IL10/CCL2/IFNG/IL1A/CAV1 | 8 |
| BP | GO:0071496 | cellular response to external stimulus | 8/32 | 327/18614 | 6.07E-08 | 1.08E-06 | 3.27E-07 | IL1B/TP53/PTGS2/BCL2/FOS/HMOX1/NFE2L2/MAPK1 | 8 |
| BP | GO:1904036 | negative regulation of epithelial cell apoptotic process | 5/32 | 62/18614 | 6.53E-08 | 1.16E-06 | 3.50E-07 | BCL2/ICAM1/HMOX1/NFE2L2/SERPINE1 | 5 |
| BP | GO:0062012 | regulation of small molecule metabolic process | 8/32 | 331/18614 | 6.66E-08 | 1.17E-06 | 3.55E-07 | AKT1/IL1B/TP53/PTGS2/HIF1A/IFNG/APP/CAV1 | 8 |
| BP | GO:0071482 | cellular response to light stimulus | 6/32 | 127/18614 | 7.02E-08 | 1.23E-06 | 3.71E-07 | TP53/PTGS2/MMP9/MYC/MMP2/CASP9 | 6 |
| BP | GO:0009895 | negative regulation of catabolic process | 8/32 | 337/18614 | 7.65E-08 | 1.33E-06 | 4.02E-07 | AKT1/IL1B/TP53/EGFR/BCL2/IL10/RELA/HMOX1 | 8 |
| BP | GO:0071214 | cellular response to abiotic stimulus | 8/32 | 339/18614 | 8.01E-08 | 1.37E-06 | 4.16E-07 | IL1B/TP53/PTGS2/CASP3/MMP9/MYC/MMP2/CASP9 | 8 |
| BP | GO:0104004 | cellular response to environmental stimulus | 8/32 | 339/18614 | 8.01E-08 | 1.37E-06 | 4.16E-07 | IL1B/TP53/PTGS2/CASP3/MMP9/MYC/MMP2/CASP9 | 8 |
| BP | GO:0072577 | endothelial cell apoptotic process | 5/32 | 65/18614 | 8.31E-08 | 1.42E-06 | 4.29E-07 | IL10/CCL2/ICAM1/NFE2L2/SERPINE1 | 5 |
| BP | GO:0010595 | positive regulation of endothelial cell migration | 6/32 | 131/18614 | 8.44E-08 | 1.42E-06 | 4.30E-07 | AKT1/PTGS2/HIF1A/EGF/HMOX1/NFE2L2 | 6 |
| BP | GO:0038127 | ERBB signaling pathway | 6/32 | 131/18614 | 8.44E-08 | 1.42E-06 | 4.30E-07 | AKT1/MMP9/EGFR/EGF/ERBB2/MAPK1 | 6 |
| BP | GO:0038034 | signal transduction in absence of ligand | 5/32 | 67/18614 | 9.69E-08 | 1.61E-06 | 4.88E-07 | AKT1/IL1B/BCL2/GSK3B/IL1A | 5 |
| BP | GO:0097192 | extrinsic apoptotic signaling pathway in absence of ligand | 5/32 | 67/18614 | 9.69E-08 | 1.61E-06 | 4.88E-07 | AKT1/IL1B/BCL2/GSK3B/IL1A | 5 |
| BP | GO:0070661 | leukocyte proliferation | 8/32 | 348/18614 | 9.79E-08 | 1.62E-06 | 4.90E-07 | IL1B/TP53/CASP3/BCL2/ERBB2/IL10/IL1A/MAPK1 | 8 |
| BP | GO:0043467 | regulation of generation of precursor metabolites and energy | 6/32 | 135/18614 | 1.01E-07 | 1.66E-06 | 5.02E-07 | AKT1/TP53/HIF1A/GSK3B/IFNG/APP | 6 |
| BP | GO:0001667 | ameboidal-type cell migration | 9/32 | 496/18614 | 1.02E-07 | 1.67E-06 | 5.06E-07 | AKT1/PTGS2/MMP9/HIF1A/PPARG/EGF/IFNG/HMOX1/NFE2L2 | 9 |
| BP | GO:0061180 | mammary gland epithelium development | 5/32 | 68/18614 | 1.05E-07 | 1.69E-06 | 5.11E-07 | AKT1/ESR1/HIF1A/CCND1/MAPK1 | 5 |
| BP | GO:0044346 | fibroblast apoptotic process | 4/32 | 26/18614 | 1.05E-07 | 1.69E-06 | 5.11E-07 | TP53/CASP3/MYC/CASP9 | 4 |
| BP | GO:0010594 | regulation of endothelial cell migration | 7/32 | 232/18614 | 1.10E-07 | 1.77E-06 | 5.35E-07 | AKT1/PTGS2/HIF1A/PPARG/EGF/HMOX1/NFE2L2 | 7 |
| BP | GO:0045787 | positive regulation of cell cycle | 8/32 | 354/18614 | 1.12E-07 | 1.78E-06 | 5.39E-07 | AKT1/IL1B/EGFR/CCND1/EGF/IL10/IL1A/APP | 8 |
| BP | GO:0032722 | positive regulation of chemokine production | 5/32 | 70/18614 | 1.21E-07 | 1.91E-06 | 5.77E-07 | IL1B/HIF1A/IFNG/HMOX1/APP | 5 |
| BP | GO:0043627 | response to estrogen | 5/32 | 70/18614 | 1.21E-07 | 1.91E-06 | 5.77E-07 | ESR1/CCND1/MMP2/HMOX1/CAV1 | 5 |
| BP | GO:0010212 | response to ionizing radiation | 6/32 | 143/18614 | 1.42E-07 | 2.21E-06 | 6.69E-07 | TP53/CASP3/BCL2/MYC/CCND1/IL1A | 6 |
| BP | GO:0030595 | leukocyte chemotaxis | 7/32 | 241/18614 | 1.43E-07 | 2.21E-06 | 6.69E-07 | IL1B/IL10/CCL2/CXCL8/MMP2/MAPK1/SERPINE1 | 7 |
| BP | GO:0097529 | myeloid leukocyte migration | 7/32 | 241/18614 | 1.43E-07 | 2.21E-06 | 6.69E-07 | IL1B/CCL2/CXCL8/IL1A/MMP2/MAPK1/SERPINE1 | 7 |
| BP | GO:0062013 | positive regulation of small molecule metabolic process | 6/32 | 146/18614 | 1.61E-07 | 2.48E-06 | 7.50E-07 | AKT1/IL1B/PTGS2/HIF1A/IFNG/APP | 6 |
| BP | GO:0010575 | positive regulation of vascular endothelial growth factor production | 4/32 | 29/18614 | 1.66E-07 | 2.54E-06 | 7.68E-07 | IL1B/PTGS2/HIF1A/IL1A | 4 |
| BP | GO:0002687 | positive regulation of leukocyte migration | 6/32 | 147/18614 | 1.68E-07 | 2.55E-06 | 7.72E-07 | CXCL8/IL1A/ICAM1/APP/MAPK1/SERPINE1 | 6 |
| BP | GO:0048863 | stem cell differentiation | 7/32 | 247/18614 | 1.69E-07 | 2.56E-06 | 7.73E-07 | TP53/ESR1/BCL2/HIF1A/GSK3B/NFE2L2/MAPK1 | 7 |
| BP | GO:0043536 | positive regulation of blood vessel endothelial cell migration | 5/32 | 75/18614 | 1.72E-07 | 2.59E-06 | 7.82E-07 | AKT1/PTGS2/HIF1A/HMOX1/NFE2L2 | 5 |
| BP | GO:0002696 | positive regulation of leukocyte activation | 8/32 | 377/18614 | 1.81E-07 | 2.71E-06 | 8.19E-07 | AKT1/IL1B/BCL2/IL10/CCL2/IFNG/IL1A/CAV1 | 8 |
| BP | GO:0150076 | neuroinflammatory response | 5/32 | 76/18614 | 1.83E-07 | 2.73E-06 | 8.26E-07 | IL1B/PTGS2/MMP9/IFNG/APP | 5 |
| BP | GO:0000082 | G1/S transition of mitotic cell cycle | 7/32 | 251/18614 | 1.88E-07 | 2.79E-06 | 8.43E-07 | AKT1/TP53/EGFR/BCL2/MYC/CCND1/CCL2 | 7 |
| BP | GO:0010821 | regulation of mitochondrion organization | 6/32 | 151/18614 | 1.96E-07 | 2.89E-06 | 8.75E-07 | AKT1/TP53/MMP9/HIF1A/PPARG/GSK3B | 6 |
| BP | GO:0043535 | regulation of blood vessel endothelial cell migration | 6/32 | 152/18614 | 2.04E-07 | 2.99E-06 | 9.05E-07 | AKT1/PTGS2/HIF1A/PPARG/HMOX1/NFE2L2 | 6 |
| BP | GO:0032368 | regulation of lipid transport | 6/32 | 153/18614 | 2.12E-07 | 3.10E-06 | 9.36E-07 | AKT1/IL1B/PPARG/EGF/IL1A/CAV1 | 6 |
| BP | GO:0010039 | response to iron ion | 4/32 | 31/18614 | 2.19E-07 | 3.16E-06 | 9.55E-07 | BCL2/HIF1A/CCND1/HMOX1 | 4 |
| BP | GO:1902042 | negative regulation of extrinsic apoptotic signaling pathway via death domain receptors | 4/32 | 31/18614 | 2.19E-07 | 3.16E-06 | 9.55E-07 | GSK3B/ICAM1/HMOX1/SERPINE1 | 4 |
| BP | GO:0045913 | positive regulation of carbohydrate metabolic process | 5/32 | 79/18614 | 2.23E-07 | 3.20E-06 | 9.67E-07 | AKT1/HIF1A/EGF/IFNG/APP | 5 |
| BP | GO:0043524 | negative regulation of neuron apoptotic process | 6/32 | 155/18614 | 2.29E-07 | 3.27E-06 | 9.89E-07 | BCL2/HIF1A/CCND1/IL10/CCL2/HMOX1 | 6 |
| BP | GO:0034976 | response to endoplasmic reticulum stress | 7/32 | 259/18614 | 2.33E-07 | 3.31E-06 | 1.00E-06 | TP53/BCL2/GSK3B/CCND1/CXCL8/NFE2L2/CAV1 | 7 |
| BP | GO:0070227 | lymphocyte apoptotic process | 5/32 | 80/18614 | 2.38E-07 | 3.36E-06 | 1.01E-06 | AKT1/TP53/BCL2/HIF1A/IL10 | 5 |
| BP | GO:0032770 | positive regulation of monooxygenase activity | 4/32 | 32/18614 | 2.50E-07 | 3.51E-06 | 1.06E-06 | AKT1/IL1B/HIF1A/IFNG | 4 |
| BP | GO:0090068 | positive regulation of cell cycle process | 7/32 | 262/18614 | 2.52E-07 | 3.52E-06 | 1.06E-06 | AKT1/IL1B/EGFR/CCND1/EGF/IL1A/APP | 7 |
| BP | GO:0050867 | positive regulation of cell activation | 8/32 | 394/18614 | 2.53E-07 | 3.52E-06 | 1.06E-06 | AKT1/IL1B/BCL2/IL10/CCL2/IFNG/IL1A/CAV1 | 8 |
| BP | GO:1990776 | response to angiotensin | 4/32 | 33/18614 | 2.84E-07 | 3.93E-06 | 1.19E-06 | PTGS2/RELA/NFE2L2/CAV1 | 4 |
| BP | GO:0070663 | regulation of leukocyte proliferation | 7/32 | 268/18614 | 2.93E-07 | 4.04E-06 | 1.22E-06 | IL1B/CASP3/BCL2/ERBB2/IL10/IL1A/MAPK1 | 7 |
| BP | GO:0034405 | response to fluid shear stress | 4/32 | 34/18614 | 3.22E-07 | 4.39E-06 | 1.33E-06 | AKT1/PTGS2/MMP2/NFE2L2 | 4 |
| BP | GO:0010660 | regulation of muscle cell apoptotic process | 5/32 | 85/18614 | 3.22E-07 | 4.39E-06 | 1.33E-06 | TP53/PPARG/IFNG/HMOX1/NFE2L2 | 5 |
| BP | GO:0032868 | response to insulin | 7/32 | 272/18614 | 3.24E-07 | 4.40E-06 | 1.33E-06 | AKT1/IL1B/FOS/PPARG/GSK3B/IL10/MAPK1 | 7 |
| BP | GO:1903039 | positive regulation of leukocyte cell-cell adhesion | 7/32 | 273/18614 | 3.32E-07 | 4.49E-06 | 1.36E-06 | AKT1/IL1B/CCL2/IFNG/IL1A/RELA/CAV1 | 7 |
| BP | GO:0008625 | extrinsic apoptotic signaling pathway via death domain receptors | 5/32 | 86/18614 | 3.42E-07 | 4.56E-06 | 1.38E-06 | BCL2/GSK3B/ICAM1/HMOX1/SERPINE1 | 5 |
| BP | GO:0033077 | T cell differentiation in thymus | 5/32 | 86/18614 | 3.42E-07 | 4.56E-06 | 1.38E-06 | IL1B/TP53/BCL2/ERBB2/IL1A | 5 |
| BP | GO:0007611 | learning or memory | 7/32 | 276/18614 | 3.58E-07 | 4.71E-06 | 1.42E-06 | PTGS2/CASP3/EGFR/HIF1A/FOS/APP/MAPK1 | 7 |
| BP | GO:0010507 | negative regulation of autophagy | 5/32 | 87/18614 | 3.62E-07 | 4.71E-06 | 1.42E-06 | AKT1/TP53/BCL2/IL10/HMOX1 | 5 |
| BP | GO:0046889 | positive regulation of lipid biosynthetic process | 5/32 | 87/18614 | 3.62E-07 | 4.71E-06 | 1.42E-06 | AKT1/IL1B/PTGS2/IFNG/IL1A | 5 |
| BP | GO:0048145 | regulation of fibroblast proliferation | 5/32 | 87/18614 | 3.62E-07 | 4.71E-06 | 1.42E-06 | TP53/ESR1/EGFR/MYC/CAV1 | 5 |
| BP | GO:1901099 | negative regulation of signal transduction in absence of ligand | 4/32 | 35/18614 | 3.63E-07 | 4.71E-06 | 1.42E-06 | AKT1/IL1B/BCL2/IL1A | 4 |
| BP | GO:2001240 | negative regulation of extrinsic apoptotic signaling pathway in absence of ligand | 4/32 | 35/18614 | 3.63E-07 | 4.71E-06 | 1.42E-06 | AKT1/IL1B/BCL2/IL1A | 4 |
| BP | GO:0007568 | aging | 6/32 | 169/18614 | 3.82E-07 | 4.93E-06 | 1.49E-06 | PTGS2/FOS/IL10/MMP2/NFE2L2/CASP9 | 6 |
| BP | GO:0044843 | cell cycle G1/S phase transition | 7/32 | 281/18614 | 4.04E-07 | 5.19E-06 | 1.57E-06 | AKT1/TP53/EGFR/BCL2/MYC/CCND1/CCL2 | 7 |
| BP | GO:0001892 | embryonic placenta development | 5/32 | 89/18614 | 4.06E-07 | 5.19E-06 | 1.57E-06 | AKT1/EGFR/HIF1A/IL10/MAPK1 | 5 |
| BP | GO:0043542 | endothelial cell migration | 7/32 | 283/18614 | 4.24E-07 | 5.40E-06 | 1.63E-06 | AKT1/PTGS2/HIF1A/PPARG/EGF/HMOX1/NFE2L2 | 7 |
| BP | GO:0010657 | muscle cell apoptotic process | 5/32 | 90/18614 | 4.29E-07 | 5.44E-06 | 1.64E-06 | TP53/PPARG/IFNG/HMOX1/NFE2L2 | 5 |
| BP | GO:0016032 | viral process | 8/32 | 426/18614 | 4.58E-07 | 5.78E-06 | 1.75E-06 | TP53/EGFR/BCL2/GSK3B/CCL2/CXCL8/ICAM1/CAV1 | 8 |
| BP | GO:0043618 | regulation of transcription from RNA polymerase II promoter in response to stress | 4/32 | 38/18614 | 5.10E-07 | 6.40E-06 | 1.93E-06 | TP53/HIF1A/HMOX1/NFE2L2 | 4 |
| BP | GO:0043534 | blood vessel endothelial cell migration | 6/32 | 179/18614 | 5.35E-07 | 6.69E-06 | 2.02E-06 | AKT1/PTGS2/HIF1A/PPARG/HMOX1/NFE2L2 | 6 |
| BP | GO:1904705 | regulation of vascular associated smooth muscle cell proliferation | 5/32 | 95/18614 | 5.62E-07 | 6.99E-06 | 2.11E-06 | MMP9/PPARG/IL10/MMP2/HMOX1 | 5 |
| BP | GO:0048771 | tissue remodeling | 6/32 | 181/18614 | 5.71E-07 | 7.08E-06 | 2.14E-06 | TP53/HIF1A/PPARG/IL1A/MMP2/CAV1 | 6 |
| BP | GO:0045936 | negative regulation of phosphate metabolic process | 8/32 | 439/18614 | 5.75E-07 | 7.08E-06 | 2.14E-06 | AKT1/IL1B/TP53/CASP3/PPARG/GSK3B/IFNG/CAV1 | 8 |
| BP | GO:0010563 | negative regulation of phosphorus metabolic process | 8/32 | 440/18614 | 5.85E-07 | 7.17E-06 | 2.17E-06 | AKT1/IL1B/TP53/CASP3/PPARG/GSK3B/IFNG/CAV1 | 8 |
| BP | GO:1905952 | regulation of lipid localization | 6/32 | 182/18614 | 5.90E-07 | 7.21E-06 | 2.18E-06 | AKT1/IL1B/PPARG/EGF/IL1A/CAV1 | 6 |
| BP | GO:0031349 | positive regulation of defense response | 8/32 | 441/18614 | 5.95E-07 | 7.23E-06 | 2.19E-06 | IL1B/PTGS2/ESR1/IFNG/RELA/APP/SERPINE1/CAV1 | 8 |
| BP | GO:0009266 | response to temperature stimulus | 6/32 | 183/18614 | 6.10E-07 | 7.38E-06 | 2.23E-06 | AKT1/PTGS2/FOS/GSK3B/IL1A/HMOX1 | 6 |
| BP | GO:1990874 | vascular associated smooth muscle cell proliferation | 5/32 | 97/18614 | 6.24E-07 | 7.52E-06 | 2.27E-06 | MMP9/PPARG/IL10/MMP2/HMOX1 | 5 |
| BP | GO:0097696 | receptor signaling pathway via STAT | 6/32 | 184/18614 | 6.29E-07 | 7.52E-06 | 2.27E-06 | PPARG/EGF/IL10/CCL2/IFNG/CAV1 | 6 |
| BP | GO:0071392 | cellular response to estradiol stimulus | 4/32 | 40/18614 | 6.29E-07 | 7.52E-06 | 2.27E-06 | ESR1/EGFR/IL10/MMP2 | 4 |
| BP | GO:0043405 | regulation of MAP kinase activity | 6/32 | 186/18614 | 6.70E-07 | 7.94E-06 | 2.40E-06 | IL1B/EGFR/PPARG/EGF/ERBB2/CAV1 | 6 |
| BP | GO:2000045 | regulation of G1/S transition of mitotic cell cycle | 6/32 | 186/18614 | 6.70E-07 | 7.94E-06 | 2.40E-06 | AKT1/TP53/EGFR/BCL2/CCND1/CCL2 | 6 |
| BP | GO:0036473 | cell death in response to oxidative stress | 5/32 | 99/18614 | 6.91E-07 | 8.14E-06 | 2.46E-06 | AKT1/BCL2/HIF1A/IL10/NFE2L2 | 5 |
| BP | GO:0046651 | lymphocyte proliferation | 7/32 | 306/18614 | 7.17E-07 | 8.41E-06 | 2.54E-06 | IL1B/TP53/CASP3/BCL2/ERBB2/IL10/IL1A | 7 |
| BP | GO:0001660 | fever generation | 3/32 | 11/18614 | 7.54E-07 | 8.78E-06 | 2.65E-06 | IL1B/PTGS2/IL1A | 3 |
| BP | GO:0061418 | regulation of transcription from RNA polymerase II promoter in response to hypoxia | 3/32 | 11/18614 | 7.54E-07 | 8.78E-06 | 2.65E-06 | TP53/HIF1A/NFE2L2 | 3 |
| BP | GO:0045429 | positive regulation of nitric oxide biosynthetic process | 4/32 | 42/18614 | 7.69E-07 | 8.91E-06 | 2.69E-06 | AKT1/IL1B/PTGS2/IFNG | 4 |
| BP | GO:0071478 | cellular response to radiation | 6/32 | 191/18614 | 7.83E-07 | 9.04E-06 | 2.73E-06 | TP53/PTGS2/MMP9/MYC/MMP2/CASP9 | 6 |
| BP | GO:0060326 | cell chemotaxis | 7/32 | 312/18614 | 8.16E-07 | 9.38E-06 | 2.84E-06 | IL1B/IL10/CCL2/CXCL8/MMP2/MAPK1/SERPINE1 | 7 |
| BP | GO:0032872 | regulation of stress-activated MAPK cascade | 6/32 | 193/18614 | 8.32E-07 | 9.50E-06 | 2.87E-06 | IL1B/EGFR/MYC/IL1A/APP/MAPK1 | 6 |
| BP | GO:0032943 | mononuclear cell proliferation | 7/32 | 313/18614 | 8.34E-07 | 9.50E-06 | 2.87E-06 | IL1B/TP53/CASP3/BCL2/ERBB2/IL10/IL1A | 7 |
| BP | GO:0048144 | fibroblast proliferation | 5/32 | 103/18614 | 8.41E-07 | 9.55E-06 | 2.89E-06 | TP53/ESR1/EGFR/MYC/CAV1 | 5 |
| BP | GO:0071364 | cellular response to epidermal growth factor stimulus | 4/32 | 43/18614 | 8.47E-07 | 9.57E-06 | 2.89E-06 | AKT1/EGFR/FOS/ERBB2 | 4 |
| BP | GO:0070372 | regulation of ERK1 and ERK2 cascade | 7/32 | 315/18614 | 8.70E-07 | 9.79E-06 | 2.96E-06 | IL1B/EGFR/ERBB2/CCL2/IL1A/ICAM1/APP | 7 |
| BP | GO:0050890 | cognition | 7/32 | 317/18614 | 9.08E-07 | 1.02E-05 | 3.07E-06 | PTGS2/CASP3/EGFR/HIF1A/FOS/APP/MAPK1 | 7 |
| BP | GO:0070302 | regulation of stress-activated protein kinase signaling cascade | 6/32 | 196/18614 | 9.11E-07 | 1.02E-05 | 3.07E-06 | IL1B/EGFR/MYC/IL1A/APP/MAPK1 | 6 |
| BP | GO:0044772 | mitotic cell cycle phase transition | 8/32 | 467/18614 | 9.16E-07 | 1.02E-05 | 3.08E-06 | AKT1/TP53/EGFR/BCL2/MYC/CCND1/CCL2/APP | 8 |
| BP | GO:1904407 | positive regulation of nitric oxide metabolic process | 4/32 | 44/18614 | 9.30E-07 | 1.03E-05 | 3.11E-06 | AKT1/IL1B/PTGS2/IFNG | 4 |
| BP | GO:0033083 | regulation of immature T cell proliferation | 3/32 | 12/18614 | 1.00E-06 | 1.10E-05 | 3.34E-06 | IL1B/ERBB2/IL1A | 3 |
| BP | GO:0033084 | regulation of immature T cell proliferation in thymus | 3/32 | 12/18614 | 1.00E-06 | 1.10E-05 | 3.34E-06 | IL1B/ERBB2/IL1A | 3 |
| BP | GO:0008631 | intrinsic apoptotic signaling pathway in response to oxidative stress | 4/32 | 45/18614 | 1.02E-06 | 1.12E-05 | 3.37E-06 | AKT1/BCL2/HIF1A/NFE2L2 | 4 |
| BP | GO:0032409 | regulation of transporter activity | 7/32 | 324/18614 | 1.05E-06 | 1.14E-05 | 3.46E-06 | MMP9/BCL2/PPARG/CCL2/IFNG/APP/CAV1 | 7 |
| BP | GO:0043393 | regulation of protein binding | 6/32 | 201/18614 | 1.06E-06 | 1.14E-05 | 3.46E-06 | AKT1/MMP9/GSK3B/IL10/APP/CAV1 | 6 |
| BP | GO:2001239 | regulation of extrinsic apoptotic signaling pathway in absence of ligand | 4/32 | 46/18614 | 1.12E-06 | 1.21E-05 | 3.64E-06 | AKT1/IL1B/BCL2/IL1A | 4 |
| BP | GO:0043281 | regulation of cysteine-type endopeptidase activity involved in apoptotic process | 6/32 | 204/18614 | 1.15E-06 | 1.24E-05 | 3.74E-06 | AKT1/PTGS2/MMP9/MYC/PPARG/CASP9 | 6 |
| BP | GO:0009408 | response to heat | 5/32 | 110/18614 | 1.17E-06 | 1.25E-05 | 3.78E-06 | AKT1/PTGS2/GSK3B/IL1A/HMOX1 | 5 |
| BP | GO:0051051 | negative regulation of transport | 8/32 | 483/18614 | 1.18E-06 | 1.26E-05 | 3.81E-06 | AKT1/IL1B/PTGS2/MMP9/BCL2/EGF/HMOX1/CAV1 | 8 |
| BP | GO:0045861 | negative regulation of proteolysis | 7/32 | 330/18614 | 1.19E-06 | 1.26E-05 | 3.82E-06 | AKT1/TP53/PTGS2/MMP9/IL10/APP/SERPINE1 | 7 |
| BP | GO:0008637 | apoptotic mitochondrial changes | 5/32 | 111/18614 | 1.22E-06 | 1.29E-05 | 3.89E-06 | AKT1/TP53/MMP9/BCL2/GSK3B | 5 |
| BP | GO:2000060 | positive regulation of ubiquitin-dependent protein catabolic process | 5/32 | 111/18614 | 1.22E-06 | 1.29E-05 | 3.89E-06 | AKT1/GSK3B/EGF/NFE2L2/CAV1 | 5 |
| BP | GO:0002683 | negative regulation of immune system process | 8/32 | 487/18614 | 1.25E-06 | 1.32E-05 | 3.99E-06 | AKT1/CASP3/MYC/PPARG/ERBB2/IL10/CCL2/HMOX1 | 8 |
| BP | GO:0033079 | immature T cell proliferation | 3/32 | 13/18614 | 1.30E-06 | 1.36E-05 | 4.11E-06 | IL1B/ERBB2/IL1A | 3 |
| BP | GO:0033080 | immature T cell proliferation in thymus | 3/32 | 13/18614 | 1.30E-06 | 1.36E-05 | 4.11E-06 | IL1B/ERBB2/IL1A | 3 |
| BP | GO:0045732 | positive regulation of protein catabolic process | 6/32 | 209/18614 | 1.32E-06 | 1.38E-05 | 4.16E-06 | AKT1/IL1B/GSK3B/IFNG/NFE2L2/CAV1 | 6 |
| BP | GO:0052548 | regulation of endopeptidase activity | 7/32 | 336/18614 | 1.34E-06 | 1.39E-05 | 4.19E-06 | AKT1/PTGS2/MMP9/MYC/PPARG/CASP9/SERPINE1 | 7 |
| BP | GO:0007173 | epidermal growth factor receptor signaling pathway | 5/32 | 114/18614 | 1.39E-06 | 1.44E-05 | 4.34E-06 | AKT1/MMP9/EGFR/EGF/ERBB2 | 5 |
| BP | GO:0043410 | positive regulation of MAPK cascade | 8/32 | 495/18614 | 1.42E-06 | 1.46E-05 | 4.40E-06 | IL1B/EGFR/EGF/ERBB2/CCL2/IL1A/ICAM1/APP | 8 |
| BP | GO:0001938 | positive regulation of endothelial cell proliferation | 5/32 | 115/18614 | 1.45E-06 | 1.49E-05 | 4.50E-06 | AKT1/HIF1A/EGF/IL10/HMOX1 | 5 |
| BP | GO:0042098 | T cell proliferation | 6/32 | 213/18614 | 1.48E-06 | 1.51E-05 | 4.56E-06 | IL1B/TP53/CASP3/ERBB2/IL10/IL1A | 6 |
| BP | GO:0015908 | fatty acid transport | 5/32 | 116/18614 | 1.52E-06 | 1.53E-05 | 4.63E-06 | AKT1/IL1B/PTGS2/PPARG/IL1A | 5 |
| BP | GO:1904892 | regulation of receptor signaling pathway via STAT | 5/32 | 116/18614 | 1.52E-06 | 1.53E-05 | 4.63E-06 | PPARG/EGF/IL10/IFNG/CAV1 | 5 |
| BP | GO:1902806 | regulation of cell cycle G1/S phase transition | 6/32 | 214/18614 | 1.52E-06 | 1.53E-05 | 4.63E-06 | AKT1/TP53/EGFR/BCL2/CCND1/CCL2 | 6 |
| BP | GO:0019216 | regulation of lipid metabolic process | 7/32 | 344/18614 | 1.57E-06 | 1.57E-05 | 4.74E-06 | AKT1/IL1B/PTGS2/PPARG/IFNG/IL1A/CAV1 | 7 |
| BP | GO:1902041 | regulation of extrinsic apoptotic signaling pathway via death domain receptors | 4/32 | 50/18614 | 1.57E-06 | 1.57E-05 | 4.74E-06 | GSK3B/ICAM1/HMOX1/SERPINE1 | 4 |
| BP | GO:0006469 | negative regulation of protein kinase activity | 6/32 | 217/18614 | 1.65E-06 | 1.64E-05 | 4.96E-06 | AKT1/IL1B/CASP3/PPARG/IFNG/CAV1 | 6 |
| BP | GO:0002065 | columnar/cuboidal epithelial cell differentiation | 5/32 | 118/18614 | 1.65E-06 | 1.64E-05 | 4.96E-06 | AKT1/HIF1A/GSK3B/SERPINE1/CAV1 | 5 |
| BP | GO:0051101 | regulation of DNA binding | 5/32 | 120/18614 | 1.79E-06 | 1.78E-05 | 5.37E-06 | MMP9/PPARG/EGF/IFNG/HMOX1 | 5 |
| BP | GO:0071675 | regulation of mononuclear cell migration | 5/32 | 122/18614 | 1.95E-06 | 1.92E-05 | 5.80E-06 | AKT1/CCL2/APP/MAPK1/SERPINE1 | 5 |
| BP | GO:1901990 | regulation of mitotic cell cycle phase transition | 7/32 | 357/18614 | 2.00E-06 | 1.97E-05 | 5.96E-06 | AKT1/TP53/EGFR/BCL2/CCND1/CCL2/APP | 7 |
| BP | GO:0022612 | gland morphogenesis | 5/32 | 123/18614 | 2.03E-06 | 1.98E-05 | 5.98E-06 | ESR1/EGFR/BCL2/MMP2/CAV1 | 5 |
| BP | GO:0071901 | negative regulation of protein serine/threonine kinase activity | 5/32 | 123/18614 | 2.03E-06 | 1.98E-05 | 5.98E-06 | AKT1/IL1B/CASP3/PPARG/CAV1 | 5 |
| BP | GO:0002573 | myeloid leukocyte differentiation | 6/32 | 227/18614 | 2.14E-06 | 2.08E-05 | 6.29E-06 | MMP9/FOS/MYC/PPARG/IFNG/APP | 6 |
| BP | GO:0008406 | gonad development | 6/32 | 228/18614 | 2.20E-06 | 2.13E-05 | 6.43E-06 | ESR1/CASP3/BCL2/CCND1/IL1A/MMP2 | 6 |
| BP | GO:1903202 | negative regulation of oxidative stress-induced cell death | 4/32 | 55/18614 | 2.31E-06 | 2.22E-05 | 6.72E-06 | AKT1/HIF1A/IL10/NFE2L2 | 4 |
| BP | GO:0031669 | cellular response to nutrient levels | 6/32 | 230/18614 | 2.31E-06 | 2.22E-05 | 6.72E-06 | TP53/BCL2/FOS/HMOX1/NFE2L2/MAPK1 | 6 |
| BP | GO:0045860 | positive regulation of protein kinase activity | 7/32 | 366/18614 | 2.36E-06 | 2.27E-05 | 6.85E-06 | AKT1/IL1B/EGFR/CCND1/EGF/ERBB2/IFNG | 7 |
| BP | GO:0070231 | T cell apoptotic process | 4/32 | 56/18614 | 2.48E-06 | 2.37E-05 | 7.16E-06 | AKT1/TP53/BCL2/HIF1A | 4 |
| BP | GO:0045137 | development of primary sexual characteristics | 6/32 | 233/18614 | 2.49E-06 | 2.37E-05 | 7.16E-06 | ESR1/CASP3/BCL2/CCND1/IL1A/MMP2 | 6 |
| BP | GO:0018108 | peptidyl-tyrosine phosphorylation | 7/32 | 370/18614 | 2.54E-06 | 2.41E-05 | 7.28E-06 | TP53/EGFR/EGF/ERBB2/IFNG/APP/CAV1 | 7 |
| BP | GO:2000116 | regulation of cysteine-type endopeptidase activity | 6/32 | 234/18614 | 2.55E-06 | 2.41E-05 | 7.29E-06 | AKT1/PTGS2/MMP9/MYC/PPARG/CASP9 | 6 |
| BP | GO:0010001 | glial cell differentiation | 6/32 | 235/18614 | 2.62E-06 | 2.46E-05 | 7.43E-06 | AKT1/IL1B/ERBB2/IFNG/APP/MAPK1 | 6 |
| BP | GO:0018212 | peptidyl-tyrosine modification | 7/32 | 372/18614 | 2.63E-06 | 2.46E-05 | 7.43E-06 | TP53/EGFR/EGF/ERBB2/IFNG/APP/CAV1 | 7 |
| BP | GO:0002931 | response to ischemia | 4/32 | 57/18614 | 2.67E-06 | 2.46E-05 | 7.43E-06 | TP53/BCL2/CASP9/CAV1 | 4 |
| BP | GO:0007566 | embryo implantation | 4/32 | 57/18614 | 2.67E-06 | 2.46E-05 | 7.43E-06 | IL1B/PTGS2/MMP9/MMP2 | 4 |
| BP | GO:0010332 | response to gamma radiation | 4/32 | 57/18614 | 2.67E-06 | 2.46E-05 | 7.43E-06 | TP53/BCL2/MYC/IL1A | 4 |
| BP | GO:0043388 | positive regulation of DNA binding | 4/32 | 57/18614 | 2.67E-06 | 2.46E-05 | 7.43E-06 | MMP9/PPARG/EGF/IFNG | 4 |
| BP | GO:0043470 | regulation of carbohydrate catabolic process | 4/32 | 57/18614 | 2.67E-06 | 2.46E-05 | 7.43E-06 | TP53/HIF1A/IFNG/APP | 4 |
| BP | GO:0051403 | stress-activated MAPK cascade | 6/32 | 237/18614 | 2.75E-06 | 2.53E-05 | 7.63E-06 | IL1B/EGFR/MYC/IL1A/APP/MAPK1 | 6 |
| BP | GO:0030336 | negative regulation of cell migration | 7/32 | 375/18614 | 2.78E-06 | 2.54E-05 | 7.69E-06 | AKT1/BCL2/PPARG/CCL2/HMOX1/NFE2L2/SERPINE1 | 7 |
| BP | GO:0001836 | release of cytochrome c from mitochondria | 4/32 | 58/18614 | 2.86E-06 | 2.61E-05 | 7.89E-06 | AKT1/TP53/MMP9/BCL2 | 4 |
| BP | GO:0033673 | negative regulation of kinase activity | 6/32 | 239/18614 | 2.88E-06 | 2.62E-05 | 7.91E-06 | AKT1/IL1B/CASP3/PPARG/IFNG/CAV1 | 6 |
| BP | GO:0050670 | regulation of lymphocyte proliferation | 6/32 | 239/18614 | 2.88E-06 | 2.62E-05 | 7.91E-06 | IL1B/CASP3/BCL2/ERBB2/IL10/IL1A | 6 |
| BP | GO:0010574 | regulation of vascular endothelial growth factor production | 4/32 | 59/18614 | 3.06E-06 | 2.75E-05 | 8.32E-06 | IL1B/PTGS2/HIF1A/IL1A | 4 |
| BP | GO:0070228 | regulation of lymphocyte apoptotic process | 4/32 | 59/18614 | 3.06E-06 | 2.75E-05 | 8.32E-06 | TP53/BCL2/HIF1A/IL10 | 4 |
| BP | GO:2000351 | regulation of endothelial cell apoptotic process | 4/32 | 59/18614 | 3.06E-06 | 2.75E-05 | 8.32E-06 | CCL2/ICAM1/NFE2L2/SERPINE1 | 4 |
| BP | GO:0031649 | heat generation | 3/32 | 17/18614 | 3.09E-06 | 2.76E-05 | 8.36E-06 | IL1B/PTGS2/IL1A | 3 |
| BP | GO:0032944 | regulation of mononuclear cell proliferation | 6/32 | 243/18614 | 3.17E-06 | 2.83E-05 | 8.56E-06 | IL1B/CASP3/BCL2/ERBB2/IL10/IL1A | 6 |
| BP | GO:1902074 | response to salt | 7/32 | 383/18614 | 3.19E-06 | 2.84E-05 | 8.58E-06 | IL1B/PTGS2/FOS/CCND1/IL1A/NFE2L2/CAV1 | 7 |
| BP | GO:0046824 | positive regulation of nucleocytoplasmic transport | 4/32 | 60/18614 | 3.28E-06 | 2.91E-05 | 8.78E-06 | IL1B/PTGS2/GSK3B/IFNG | 4 |
| BP | GO:0031098 | stress-activated protein kinase signaling cascade | 6/32 | 245/18614 | 3.33E-06 | 2.94E-05 | 8.89E-06 | IL1B/EGFR/MYC/IL1A/APP/MAPK1 | 6 |
| BP | GO:1903052 | positive regulation of proteolysis involved in protein catabolic process | 5/32 | 137/18614 | 3.44E-06 | 3.03E-05 | 9.17E-06 | AKT1/GSK3B/EGF/NFE2L2/CAV1 | 5 |
| BP | GO:2000146 | negative regulation of cell motility | 7/32 | 390/18614 | 3.60E-06 | 3.16E-05 | 9.54E-06 | AKT1/BCL2/PPARG/CCL2/HMOX1/NFE2L2/SERPINE1 | 7 |
| BP | GO:0050870 | positive regulation of T cell activation | 6/32 | 249/18614 | 3.65E-06 | 3.20E-05 | 9.66E-06 | AKT1/IL1B/CCL2/IFNG/IL1A/CAV1 | 6 |
| BP | GO:0032310 | prostaglandin secretion | 3/32 | 18/18614 | 3.70E-06 | 3.21E-05 | 9.69E-06 | IL1B/PTGS2/IL1A | 3 |
| BP | GO:0036003 | positive regulation of transcription from RNA polymerase II promoter in response to stress | 3/32 | 18/18614 | 3.70E-06 | 3.21E-05 | 9.69E-06 | TP53/HIF1A/NFE2L2 | 3 |
| BP | GO:0038128 | ERBB2 signaling pathway | 3/32 | 18/18614 | 3.70E-06 | 3.21E-05 | 9.69E-06 | EGFR/EGF/ERBB2 | 3 |
| BP | GO:0010573 | vascular endothelial growth factor production | 4/32 | 63/18614 | 3.99E-06 | 3.44E-05 | 1.04E-05 | IL1B/PTGS2/HIF1A/IL1A | 4 |
| BP | GO:0051353 | positive regulation of oxidoreductase activity | 4/32 | 63/18614 | 3.99E-06 | 3.44E-05 | 1.04E-05 | AKT1/IL1B/HIF1A/IFNG | 4 |
| BP | GO:0038061 | NIK/NF-kappaB signaling | 5/32 | 142/18614 | 4.11E-06 | 3.52E-05 | 1.07E-05 | AKT1/IL1B/EGFR/RELA/APP | 5 |
| BP | GO:0050730 | regulation of peptidyl-tyrosine phosphorylation | 6/32 | 255/18614 | 4.19E-06 | 3.58E-05 | 1.08E-05 | TP53/EGFR/EGF/IFNG/APP/CAV1 | 6 |
| BP | GO:1902176 | negative regulation of oxidative stress-induced intrinsic apoptotic signaling pathway | 3/32 | 19/18614 | 4.39E-06 | 3.74E-05 | 1.13E-05 | AKT1/HIF1A/NFE2L2 | 3 |
| BP | GO:0006879 | intracellular iron ion homeostasis | 4/32 | 65/18614 | 4.52E-06 | 3.83E-05 | 1.16E-05 | HIF1A/MYC/IFNG/HMOX1 | 4 |
| BP | GO:0031663 | lipopolysaccharide-mediated signaling pathway | 4/32 | 65/18614 | 4.52E-06 | 3.83E-05 | 1.16E-05 | AKT1/IL1B/CCL2/MAPK1 | 4 |
| BP | GO:0045786 | negative regulation of cell cycle | 7/32 | 404/18614 | 4.54E-06 | 3.83E-05 | 1.16E-05 | TP53/PTGS2/CASP3/BCL2/CCND1/IL10/CCL2 | 7 |
| BP | GO:0031668 | cellular response to extracellular stimulus | 6/32 | 261/18614 | 4.79E-06 | 4.03E-05 | 1.22E-05 | TP53/BCL2/FOS/HMOX1/NFE2L2/MAPK1 | 6 |
| BP | GO:0034605 | cellular response to heat | 4/32 | 67/18614 | 5.11E-06 | 4.28E-05 | 1.29E-05 | PTGS2/GSK3B/IL1A/HMOX1 | 4 |
| BP | GO:1901224 | positive regulation of NIK/NF-kappaB signaling | 4/32 | 67/18614 | 5.11E-06 | 4.28E-05 | 1.29E-05 | IL1B/EGFR/RELA/APP | 4 |
| BP | GO:0097709 | connective tissue replacement | 3/32 | 20/18614 | 5.16E-06 | 4.29E-05 | 1.30E-05 | HIF1A/PPARG/IL1A | 3 |
| BP | GO:1902004 | positive regulation of amyloid-beta formation | 3/32 | 20/18614 | 5.16E-06 | 4.29E-05 | 1.30E-05 | CASP3/IFNG/RELA | 3 |
| BP | GO:0045834 | positive regulation of lipid metabolic process | 5/32 | 151/18614 | 5.55E-06 | 4.60E-05 | 1.39E-05 | AKT1/IL1B/PTGS2/IFNG/IL1A | 5 |
| BP | GO:0034763 | negative regulation of transmembrane transport | 5/32 | 153/18614 | 5.92E-06 | 4.89E-05 | 1.48E-05 | AKT1/IL1B/MMP9/BCL2/CAV1 | 5 |
| BP | GO:0045821 | positive regulation of glycolytic process | 3/32 | 21/18614 | 6.01E-06 | 4.92E-05 | 1.49E-05 | HIF1A/IFNG/APP | 3 |
| BP | GO:0060353 | regulation of cell adhesion molecule production | 3/32 | 21/18614 | 6.01E-06 | 4.92E-05 | 1.49E-05 | IL1B/CXCL8/CAV1 | 3 |
| BP | GO:0071498 | cellular response to fluid shear stress | 3/32 | 21/18614 | 6.01E-06 | 4.92E-05 | 1.49E-05 | PTGS2/MMP2/NFE2L2 | 3 |
| BP | GO:0030098 | lymphocyte differentiation | 7/32 | 422/18614 | 6.04E-06 | 4.93E-05 | 1.49E-05 | IL1B/TP53/BCL2/ERBB2/IL10/IFNG/IL1A | 7 |
| BP | GO:0014823 | response to activity | 4/32 | 70/18614 | 6.09E-06 | 4.96E-05 | 1.50E-05 | HIF1A/FOS/IL10/MMP2 | 4 |
| BP | GO:0050729 | positive regulation of inflammatory response | 5/32 | 154/18614 | 6.11E-06 | 4.96E-05 | 1.50E-05 | IL1B/PTGS2/IFNG/APP/SERPINE1 | 5 |
| BP | GO:0010959 | regulation of metal ion transport | 7/32 | 423/18614 | 6.13E-06 | 4.96E-05 | 1.50E-05 | AKT1/PTGS2/BCL2/EGF/CCL2/IFNG/CAV1 | 7 |
| BP | GO:0090316 | positive regulation of intracellular protein transport | 5/32 | 155/18614 | 6.30E-06 | 5.09E-05 | 1.54E-05 | IL1B/PTGS2/GSK3B/ERBB2/IFNG | 5 |
| BP | GO:0007584 | response to nutrient | 5/32 | 156/18614 | 6.50E-06 | 5.22E-05 | 1.58E-05 | PTGS2/PPARG/CCND1/IL1A/HMOX1 | 5 |
| BP | GO:0097530 | granulocyte migration | 5/32 | 156/18614 | 6.50E-06 | 5.22E-05 | 1.58E-05 | IL1B/CCL2/CXCL8/IL1A/MAPK1 | 5 |
| BP | GO:0040013 | negative regulation of locomotion | 7/32 | 427/18614 | 6.52E-06 | 5.22E-05 | 1.58E-05 | AKT1/BCL2/PPARG/CCL2/HMOX1/NFE2L2/SERPINE1 | 7 |
| BP | GO:0002260 | lymphocyte homeostasis | 4/32 | 72/18614 | 6.82E-06 | 5.43E-05 | 1.64E-05 | AKT1/CASP3/BCL2/HIF1A | 4 |
| BP | GO:0033674 | positive regulation of kinase activity | 7/32 | 430/18614 | 6.83E-06 | 5.43E-05 | 1.64E-05 | AKT1/IL1B/EGFR/CCND1/EGF/ERBB2/IFNG | 7 |
| BP | GO:1902894 | negative regulation of miRNA transcription | 3/32 | 22/18614 | 6.95E-06 | 5.50E-05 | 1.66E-05 | ESR1/PPARG/RELA | 3 |
| BP | GO:2000310 | regulation of NMDA receptor activity | 3/32 | 22/18614 | 6.95E-06 | 5.50E-05 | 1.66E-05 | CCL2/IFNG/APP | 3 |
| BP | GO:0016241 | regulation of macroautophagy | 5/32 | 159/18614 | 7.14E-06 | 5.63E-05 | 1.70E-05 | AKT1/TP53/CASP3/HIF1A/HMOX1 | 5 |
| BP | GO:0002067 | glandular epithelial cell differentiation | 4/32 | 74/18614 | 7.61E-06 | 5.97E-05 | 1.80E-05 | AKT1/HIF1A/GSK3B/CAV1 | 4 |
| BP | GO:0007548 | sex differentiation | 6/32 | 283/18614 | 7.61E-06 | 5.97E-05 | 1.80E-05 | ESR1/CASP3/BCL2/CCND1/IL1A/MMP2 | 6 |
| BP | GO:2000629 | negative regulation of miRNA metabolic process | 3/32 | 23/18614 | 7.98E-06 | 6.24E-05 | 1.89E-05 | ESR1/PPARG/RELA | 3 |
| BP | GO:0010822 | positive regulation of mitochondrion organization | 4/32 | 75/18614 | 8.02E-06 | 6.24E-05 | 1.89E-05 | TP53/MMP9/HIF1A/GSK3B | 4 |
| BP | GO:0048662 | negative regulation of smooth muscle cell proliferation | 4/32 | 75/18614 | 8.02E-06 | 6.24E-05 | 1.89E-05 | PPARG/IL10/IFNG/HMOX1 | 4 |
| BP | GO:0051100 | negative regulation of binding | 5/32 | 163/18614 | 8.05E-06 | 6.24E-05 | 1.89E-05 | AKT1/GSK3B/IL10/HMOX1/CAV1 | 5 |
| BP | GO:0010721 | negative regulation of cell development | 6/32 | 286/18614 | 8.08E-06 | 6.25E-05 | 1.89E-05 | IL1B/TP53/MYC/GSK3B/ERBB2/IL1A | 6 |
| BP | GO:0022898 | regulation of transmembrane transporter activity | 6/32 | 289/18614 | 8.58E-06 | 6.61E-05 | 2.00E-05 | MMP9/BCL2/CCL2/IFNG/APP/CAV1 | 6 |
| BP | GO:0006869 | lipid transport | 7/32 | 447/18614 | 8.79E-06 | 6.76E-05 | 2.04E-05 | AKT1/IL1B/PTGS2/PPARG/EGF/IL1A/CAV1 | 7 |
| BP | GO:0048143 | astrocyte activation | 3/32 | 24/18614 | 9.11E-06 | 6.94E-05 | 2.10E-05 | IL1B/IFNG/APP | 3 |
| BP | GO:0051043 | regulation of membrane protein ectodomain proteolysis | 3/32 | 24/18614 | 9.11E-06 | 6.94E-05 | 2.10E-05 | IL1B/IL10/IFNG | 3 |
| BP | GO:0060352 | cell adhesion molecule production | 3/32 | 24/18614 | 9.11E-06 | 6.94E-05 | 2.10E-05 | IL1B/CXCL8/CAV1 | 3 |
| BP | GO:0007249 | I-kappaB kinase/NF-kappaB signaling | 6/32 | 293/18614 | 9.28E-06 | 7.05E-05 | 2.13E-05 | AKT1/IL1B/ESR1/IL1A/RELA/HMOX1 | 6 |
| BP | GO:0048568 | embryonic organ development | 7/32 | 453/18614 | 9.59E-06 | 7.25E-05 | 2.19E-05 | AKT1/TP53/EGFR/HIF1A/IL10/CXCL8/MAPK1 | 7 |
| BP | GO:0034250 | positive regulation of amide metabolic process | 5/32 | 169/18614 | 9.60E-06 | 7.25E-05 | 2.19E-05 | CASP3/ERBB2/IFNG/RELA/NFE2L2 | 5 |
| BP | GO:1903201 | regulation of oxidative stress-induced cell death | 4/32 | 79/18614 | 9.87E-06 | 7.40E-05 | 2.24E-05 | AKT1/HIF1A/IL10/NFE2L2 | 4 |
| BP | GO:0009267 | cellular response to starvation | 5/32 | 170/18614 | 9.88E-06 | 7.40E-05 | 2.24E-05 | TP53/BCL2/FOS/NFE2L2/MAPK1 | 5 |
| BP | GO:0050806 | positive regulation of synaptic transmission | 5/32 | 170/18614 | 9.88E-06 | 7.40E-05 | 2.24E-05 | PTGS2/GSK3B/CCL2/APP/MAPK1 | 5 |
| BP | GO:1901987 | regulation of cell cycle phase transition | 7/32 | 457/18614 | 1.02E-05 | 7.59E-05 | 2.29E-05 | AKT1/TP53/EGFR/BCL2/CCND1/CCL2/APP | 7 |
| BP | GO:0010623 | programmed cell death involved in cell development | 3/32 | 25/18614 | 1.03E-05 | 7.67E-05 | 2.32E-05 | IL1B/BCL2/IL1A | 3 |
| BP | GO:0060333 | type II interferon-mediated signaling pathway | 3/32 | 25/18614 | 1.03E-05 | 7.67E-05 | 2.32E-05 | TP53/PPARG/IFNG | 3 |
| BP | GO:1902993 | positive regulation of amyloid precursor protein catabolic process | 3/32 | 25/18614 | 1.03E-05 | 7.67E-05 | 2.32E-05 | CASP3/IFNG/RELA | 3 |
| BP | GO:0002263 | cell activation involved in immune response | 6/32 | 300/18614 | 1.06E-05 | 7.82E-05 | 2.37E-05 | TP53/IL10/IFNG/ICAM1/HMOX1/APP | 6 |
| BP | GO:0030217 | T cell differentiation | 6/32 | 300/18614 | 1.06E-05 | 7.82E-05 | 2.37E-05 | IL1B/TP53/BCL2/ERBB2/IFNG/IL1A | 6 |
| BP | GO:0007259 | receptor signaling pathway via JAK-STAT | 5/32 | 173/18614 | 1.08E-05 | 7.88E-05 | 2.38E-05 | EGF/IL10/CCL2/IFNG/CAV1 | 5 |
| BP | GO:2000058 | regulation of ubiquitin-dependent protein catabolic process | 5/32 | 173/18614 | 1.08E-05 | 7.88E-05 | 2.38E-05 | AKT1/GSK3B/EGF/NFE2L2/CAV1 | 5 |
| BP | GO:0048608 | reproductive structure development | 6/32 | 301/18614 | 1.08E-05 | 7.91E-05 | 2.39E-05 | ESR1/CASP3/BCL2/CCND1/IL1A/MMP2 | 6 |
| BP | GO:0048511 | rhythmic process | 6/32 | 303/18614 | 1.12E-05 | 8.19E-05 | 2.48E-05 | TP53/ESR1/CASP3/PPARG/GSK3B/MMP2 | 6 |
| BP | GO:0061458 | reproductive system development | 6/32 | 305/18614 | 1.17E-05 | 8.47E-05 | 2.56E-05 | ESR1/CASP3/BCL2/CCND1/IL1A/MMP2 | 6 |
| BP | GO:0033598 | mammary gland epithelial cell proliferation | 3/32 | 26/18614 | 1.17E-05 | 8.47E-05 | 2.56E-05 | ESR1/CCND1/MAPK1 | 3 |
| BP | GO:0015718 | monocarboxylic acid transport | 5/32 | 177/18614 | 1.20E-05 | 8.69E-05 | 2.63E-05 | AKT1/IL1B/PTGS2/PPARG/IL1A | 5 |
| BP | GO:0032635 | interleukin-6 production | 5/32 | 178/18614 | 1.23E-05 | 8.88E-05 | 2.69E-05 | IL1B/IL10/IFNG/IL1A/APP | 5 |
| BP | GO:0032675 | regulation of interleukin-6 production | 5/32 | 178/18614 | 1.23E-05 | 8.88E-05 | 2.69E-05 | IL1B/IL10/IFNG/IL1A/APP | 5 |
| BP | GO:0007162 | negative regulation of cell adhesion | 6/32 | 312/18614 | 1.33E-05 | 9.52E-05 | 2.88E-05 | AKT1/CASP3/ERBB2/IL10/MMP2/SERPINE1 | 6 |
| BP | GO:0046890 | regulation of lipid biosynthetic process | 5/32 | 182/18614 | 1.37E-05 | 9.84E-05 | 2.97E-05 | AKT1/IL1B/PTGS2/IFNG/IL1A | 5 |
| BP | GO:2000134 | negative regulation of G1/S transition of mitotic cell cycle | 4/32 | 86/18614 | 1.38E-05 | 9.87E-05 | 2.99E-05 | TP53/BCL2/CCND1/CCL2 | 4 |
| BP | GO:0042129 | regulation of T cell proliferation | 5/32 | 183/18614 | 1.41E-05 | 0.0001 | 3.04E-05 | IL1B/CASP3/ERBB2/IL10/IL1A | 5 |
| BP | GO:0032092 | positive regulation of protein binding | 4/32 | 87/18614 | 1.45E-05 | 0.000102 | 3.09E-05 | MMP9/GSK3B/APP/CAV1 | 4 |
| BP | GO:0032370 | positive regulation of lipid transport | 4/32 | 87/18614 | 1.45E-05 | 0.000102 | 3.09E-05 | IL1B/PPARG/IL1A/CAV1 | 4 |
| BP | GO:0048708 | astrocyte differentiation | 4/32 | 87/18614 | 1.45E-05 | 0.000102 | 3.09E-05 | IL1B/IFNG/APP/MAPK1 | 4 |
| BP | GO:0097194 | execution phase of apoptosis | 4/32 | 87/18614 | 1.45E-05 | 0.000102 | 3.09E-05 | AKT1/TP53/CASP3/CASP9 | 4 |
| BP | GO:0019058 | viral life cycle | 6/32 | 320/18614 | 1.53E-05 | 0.000107 | 3.24E-05 | EGFR/BCL2/CCL2/CXCL8/ICAM1/CAV1 | 6 |
| BP | GO:0060485 | mesenchyme development | 6/32 | 320/18614 | 1.53E-05 | 0.000107 | 3.24E-05 | IL1B/BCL2/HIF1A/MYC/GSK3B/MAPK1 | 6 |
| BP | GO:0055072 | iron ion homeostasis | 4/32 | 89/18614 | 1.59E-05 | 0.000111 | 3.35E-05 | HIF1A/MYC/IFNG/HMOX1 | 4 |
| BP | GO:0061138 | morphogenesis of a branching epithelium | 5/32 | 188/18614 | 1.61E-05 | 0.000112 | 3.39E-05 | ESR1/BCL2/MYC/EGF/IL10 | 5 |
| BP | GO:0010800 | positive regulation of peptidyl-threonine phosphorylation | 3/32 | 29/18614 | 1.64E-05 | 0.000113 | 3.41E-05 | EGF/APP/MAPK1 | 3 |
| BP | GO:0015732 | prostaglandin transport | 3/32 | 29/18614 | 1.64E-05 | 0.000113 | 3.41E-05 | IL1B/PTGS2/IL1A | 3 |
| BP | GO:1902175 | regulation of oxidative stress-induced intrinsic apoptotic signaling pathway | 3/32 | 29/18614 | 1.64E-05 | 0.000113 | 3.41E-05 | AKT1/HIF1A/NFE2L2 | 3 |
| BP | GO:1904385 | cellular response to angiotensin | 3/32 | 29/18614 | 1.64E-05 | 0.000113 | 3.41E-05 | RELA/NFE2L2/CAV1 | 3 |
| BP | GO:2000106 | regulation of leukocyte apoptotic process | 4/32 | 90/18614 | 1.66E-05 | 0.000114 | 3.45E-05 | TP53/BCL2/HIF1A/IL10 | 4 |
| BP | GO:0031099 | regeneration | 5/32 | 190/18614 | 1.69E-05 | 0.000116 | 3.51E-05 | BCL2/CCND1/IL10/MMP2/HMOX1 | 5 |
| BP | GO:0016049 | cell growth | 7/32 | 495/18614 | 1.70E-05 | 0.000117 | 3.53E-05 | AKT1/TP53/EGFR/BCL2/GSK3B/ERBB2/APP | 7 |
| BP | GO:0010876 | lipid localization | 7/32 | 497/18614 | 1.75E-05 | 0.000119 | 3.61E-05 | AKT1/IL1B/PTGS2/PPARG/EGF/IL1A/CAV1 | 7 |
| BP | GO:0006913 | nucleocytoplasmic transport | 6/32 | 328/18614 | 1.76E-05 | 0.00012 | 3.62E-05 | AKT1/IL1B/TP53/PTGS2/GSK3B/IFNG | 6 |
| BP | GO:0051169 | nuclear transport | 6/32 | 328/18614 | 1.76E-05 | 0.00012 | 3.62E-05 | AKT1/IL1B/TP53/PTGS2/GSK3B/IFNG | 6 |
| BP | GO:0097421 | liver regeneration | 3/32 | 30/18614 | 1.82E-05 | 0.000123 | 3.72E-05 | CCND1/IL10/HMOX1 | 3 |
| BP | GO:0032386 | regulation of intracellular transport | 6/32 | 332/18614 | 1.89E-05 | 0.000127 | 3.84E-05 | IL1B/PTGS2/GSK3B/ERBB2/IFNG/MAPK1 | 6 |
| BP | GO:0032436 | positive regulation of proteasomal ubiquitin-dependent protein catabolic process | 4/32 | 93/18614 | 1.89E-05 | 0.000127 | 3.84E-05 | AKT1/GSK3B/NFE2L2/CAV1 | 4 |
| BP | GO:1900542 | regulation of purine nucleotide metabolic process | 4/32 | 93/18614 | 1.89E-05 | 0.000127 | 3.84E-05 | TP53/HIF1A/IFNG/APP | 4 |
| BP | GO:0060562 | epithelial tube morphogenesis | 6/32 | 334/18614 | 1.95E-05 | 0.000131 | 3.96E-05 | ESR1/CASP3/BCL2/HIF1A/MYC/EGF | 6 |
| BP | GO:0006140 | regulation of nucleotide metabolic process | 4/32 | 94/18614 | 1.97E-05 | 0.000131 | 3.97E-05 | TP53/HIF1A/IFNG/APP | 4 |
| BP | GO:1900407 | regulation of cellular response to oxidative stress | 4/32 | 94/18614 | 1.97E-05 | 0.000131 | 3.97E-05 | AKT1/HIF1A/IL10/NFE2L2 | 4 |
| BP | GO:0032388 | positive regulation of intracellular transport | 5/32 | 197/18614 | 2.01E-05 | 0.000134 | 4.06E-05 | IL1B/PTGS2/GSK3B/ERBB2/IFNG | 5 |
| BP | GO:0015980 | energy derivation by oxidation of organic compounds | 6/32 | 337/18614 | 2.05E-05 | 0.000136 | 4.11E-05 | AKT1/TP53/HIF1A/MYC/GSK3B/IFNG | 6 |
| BP | GO:0019217 | regulation of fatty acid metabolic process | 4/32 | 95/18614 | 2.05E-05 | 0.000136 | 4.11E-05 | AKT1/IL1B/PTGS2/CAV1 | 4 |
| BP | GO:0032091 | negative regulation of protein binding | 4/32 | 96/18614 | 2.14E-05 | 0.000141 | 4.28E-05 | AKT1/GSK3B/IL10/CAV1 | 4 |
| BP | GO:0001933 | negative regulation of protein phosphorylation | 6/32 | 341/18614 | 2.19E-05 | 0.000145 | 4.37E-05 | AKT1/IL1B/CASP3/PPARG/IFNG/CAV1 | 6 |
| BP | GO:0010165 | response to X-ray | 3/32 | 32/18614 | 2.21E-05 | 0.000146 | 4.40E-05 | TP53/CASP3/CCND1 | 3 |
| BP | GO:1902807 | negative regulation of cell cycle G1/S phase transition | 4/32 | 97/18614 | 2.23E-05 | 0.000146 | 4.42E-05 | TP53/BCL2/CCND1/CCL2 | 4 |
| BP | GO:0071674 | mononuclear cell migration | 5/32 | 202/18614 | 2.27E-05 | 0.000149 | 4.50E-05 | AKT1/CCL2/APP/MAPK1/SERPINE1 | 5 |
| BP | GO:0001763 | morphogenesis of a branching structure | 5/32 | 203/18614 | 2.33E-05 | 0.000152 | 4.60E-05 | ESR1/BCL2/MYC/EGF/IL10 | 5 |
| BP | GO:0051346 | negative regulation of hydrolase activity | 6/32 | 345/18614 | 2.34E-05 | 0.000153 | 4.61E-05 | AKT1/PTGS2/MMP9/GSK3B/APP/SERPINE1 | 6 |
| BP | GO:1901992 | positive regulation of mitotic cell cycle phase transition | 4/32 | 99/18614 | 2.41E-05 | 0.000157 | 4.75E-05 | AKT1/EGFR/CCND1/APP | 4 |
| BP | GO:0048147 | negative regulation of fibroblast proliferation | 3/32 | 33/18614 | 2.43E-05 | 0.000157 | 4.76E-05 | TP53/MYC/CAV1 | 3 |
| BP | GO:2000191 | regulation of fatty acid transport | 3/32 | 33/18614 | 2.43E-05 | 0.000157 | 4.76E-05 | AKT1/IL1B/IL1A | 3 |
| BP | GO:0032755 | positive regulation of interleukin-6 production | 4/32 | 100/18614 | 2.51E-05 | 0.000162 | 4.90E-05 | IL1B/IFNG/IL1A/APP | 4 |
| BP | GO:0042594 | response to starvation | 5/32 | 207/18614 | 2.56E-05 | 0.000165 | 4.98E-05 | TP53/BCL2/FOS/NFE2L2/MAPK1 | 5 |
| BP | GO:1901654 | response to ketone | 5/32 | 208/18614 | 2.62E-05 | 0.000168 | 5.08E-05 | AKT1/FOS/CCND1/CASP9/CAV1 | 5 |
| BP | GO:0001782 | B cell homeostasis | 3/32 | 34/18614 | 2.66E-05 | 0.00017 | 5.14E-05 | CASP3/BCL2/HIF1A | 3 |
| BP | GO:0010661 | positive regulation of muscle cell apoptotic process | 3/32 | 34/18614 | 2.66E-05 | 0.00017 | 5.14E-05 | TP53/PPARG/IFNG | 3 |
| BP | GO:0016242 | negative regulation of macroautophagy | 3/32 | 34/18614 | 2.66E-05 | 0.00017 | 5.14E-05 | AKT1/TP53/HMOX1 | 3 |
| BP | GO:0008585 | female gonad development | 4/32 | 102/18614 | 2.72E-05 | 0.000172 | 5.22E-05 | ESR1/CASP3/BCL2/MMP2 | 4 |
| BP | GO:1905477 | positive regulation of protein localization to membrane | 4/32 | 102/18614 | 2.72E-05 | 0.000172 | 5.22E-05 | AKT1/EGFR/ERBB2/IFNG | 4 |
| BP | GO:0032869 | cellular response to insulin stimulus | 5/32 | 211/18614 | 2.80E-05 | 0.000177 | 5.34E-05 | AKT1/IL1B/PPARG/GSK3B/MAPK1 | 5 |
| BP | GO:0001776 | leukocyte homeostasis | 4/32 | 103/18614 | 2.82E-05 | 0.000177 | 5.34E-05 | AKT1/CASP3/BCL2/HIF1A | 4 |
| BP | GO:0008630 | intrinsic apoptotic signaling pathway in response to DNA damage | 4/32 | 103/18614 | 2.82E-05 | 0.000177 | 5.34E-05 | TP53/BCL2/HMOX1/CASP9 | 4 |
| BP | GO:0019233 | sensory perception of pain | 4/32 | 103/18614 | 2.82E-05 | 0.000177 | 5.34E-05 | PTGS2/IL10/CCL2/IL1A | 4 |
| BP | GO:0032677 | regulation of interleukin-8 production | 4/32 | 103/18614 | 2.82E-05 | 0.000177 | 5.34E-05 | IL1B/IL10/RELA/SERPINE1 | 4 |
| BP | GO:1902882 | regulation of response to oxidative stress | 4/32 | 103/18614 | 2.82E-05 | 0.000177 | 5.34E-05 | AKT1/HIF1A/IL10/NFE2L2 | 4 |
| BP | GO:0043276 | anoikis | 3/32 | 35/18614 | 2.91E-05 | 0.000181 | 5.47E-05 | AKT1/BCL2/CAV1 | 3 |
| BP | GO:1902253 | regulation of intrinsic apoptotic signaling pathway by p53 class mediator | 3/32 | 35/18614 | 2.91E-05 | 0.000181 | 5.47E-05 | TP53/BCL2/MYC | 3 |
| BP | GO:2000352 | negative regulation of endothelial cell apoptotic process | 3/32 | 35/18614 | 2.91E-05 | 0.000181 | 5.47E-05 | ICAM1/NFE2L2/SERPINE1 | 3 |
| BP | GO:0032637 | interleukin-8 production | 4/32 | 104/18614 | 2.93E-05 | 0.000182 | 5.50E-05 | IL1B/IL10/RELA/SERPINE1 | 4 |
| BP | GO:0046425 | regulation of receptor signaling pathway via JAK-STAT | 4/32 | 105/18614 | 3.04E-05 | 0.000188 | 5.70E-05 | EGF/IL10/IFNG/CAV1 | 4 |
| BP | GO:0009612 | response to mechanical stimulus | 5/32 | 215/18614 | 3.07E-05 | 0.000189 | 5.71E-05 | IL1B/PTGS2/FOS/RELA/MMP2 | 5 |
| BP | GO:0043491 | protein kinase B signaling | 5/32 | 215/18614 | 3.07E-05 | 0.000189 | 5.71E-05 | AKT1/IL1B/EGFR/EGF/CCL2 | 5 |
| BP | GO:0046545 | development of primary female sexual characteristics | 4/32 | 106/18614 | 3.16E-05 | 0.000193 | 5.84E-05 | ESR1/CASP3/BCL2/MMP2 | 4 |
| BP | GO:1901796 | regulation of signal transduction by p53 class mediator | 4/32 | 106/18614 | 3.16E-05 | 0.000193 | 5.84E-05 | AKT1/TP53/BCL2/MYC | 4 |
| BP | GO:0002068 | glandular epithelial cell development | 3/32 | 36/18614 | 3.17E-05 | 0.000193 | 5.84E-05 | AKT1/HIF1A/GSK3B | 3 |
| BP | GO:0060218 | hematopoietic stem cell differentiation | 3/32 | 36/18614 | 3.17E-05 | 0.000193 | 5.84E-05 | TP53/BCL2/NFE2L2 | 3 |
| BP | GO:1903580 | positive regulation of ATP metabolic process | 3/32 | 36/18614 | 3.17E-05 | 0.000193 | 5.84E-05 | HIF1A/IFNG/APP | 3 |
| BP | GO:0046822 | regulation of nucleocytoplasmic transport | 4/32 | 107/18614 | 3.28E-05 | 0.000199 | 6.03E-05 | IL1B/PTGS2/GSK3B/IFNG | 4 |
| BP | GO:0070374 | positive regulation of ERK1 and ERK2 cascade | 5/32 | 222/18614 | 3.57E-05 | 0.000217 | 6.55E-05 | EGFR/CCL2/IL1A/ICAM1/APP | 5 |
| BP | GO:1901222 | regulation of NIK/NF-kappaB signaling | 4/32 | 110/18614 | 3.65E-05 | 0.000221 | 6.69E-05 | IL1B/EGFR/RELA/APP | 4 |
| BP | GO:0033157 | regulation of intracellular protein transport | 5/32 | 224/18614 | 3.73E-05 | 0.000225 | 6.81E-05 | IL1B/PTGS2/GSK3B/ERBB2/IFNG | 5 |
| BP | GO:0032885 | regulation of polysaccharide biosynthetic process | 3/32 | 38/18614 | 3.74E-05 | 0.000225 | 6.81E-05 | AKT1/GSK3B/EGF | 3 |
| BP | GO:0000079 | regulation of cyclin-dependent protein serine/threonine kinase activity | 4/32 | 112/18614 | 3.92E-05 | 0.000235 | 7.11E-05 | AKT1/CASP3/EGFR/CCND1 | 4 |
| BP | GO:1905954 | positive regulation of lipid localization | 4/32 | 112/18614 | 3.92E-05 | 0.000235 | 7.11E-05 | IL1B/PPARG/IL1A/CAV1 | 4 |
| BP | GO:0046777 | protein autophosphorylation | 5/32 | 227/18614 | 3.97E-05 | 0.000238 | 7.19E-05 | AKT1/EGFR/GSK3B/ERBB2/CAV1 | 5 |
| BP | GO:0045737 | positive regulation of cyclin-dependent protein serine/threonine kinase activity | 3/32 | 39/18614 | 4.04E-05 | 0.000241 | 7.29E-05 | AKT1/EGFR/CCND1 | 3 |
| BP | GO:0070232 | regulation of T cell apoptotic process | 3/32 | 39/18614 | 4.04E-05 | 0.000241 | 7.29E-05 | TP53/BCL2/HIF1A | 3 |
| BP | GO:0042326 | negative regulation of phosphorylation | 6/32 | 383/18614 | 4.20E-05 | 0.00025 | 7.56E-05 | AKT1/IL1B/CASP3/PPARG/IFNG/CAV1 | 6 |
| BP | GO:1903050 | regulation of proteolysis involved in protein catabolic process | 5/32 | 230/18614 | 4.23E-05 | 0.000251 | 7.59E-05 | AKT1/GSK3B/EGF/NFE2L2/CAV1 | 5 |
| BP | GO:0042303 | molting cycle | 4/32 | 115/18614 | 4.35E-05 | 0.000257 | 7.77E-05 | PTGS2/EGFR/BCL2/RELA | 4 |
| BP | GO:0042633 | hair cycle | 4/32 | 115/18614 | 4.35E-05 | 0.000257 | 7.77E-05 | PTGS2/EGFR/BCL2/RELA | 4 |
| BP | GO:0071346 | cellular response to type II interferon | 4/32 | 116/18614 | 4.50E-05 | 0.000265 | 8.00E-05 | TP53/PPARG/CCL2/IFNG | 4 |
| BP | GO:1904029 | regulation of cyclin-dependent protein kinase activity | 4/32 | 116/18614 | 4.50E-05 | 0.000265 | 8.00E-05 | AKT1/CASP3/EGFR/CCND1 | 4 |
| BP | GO:0009743 | response to carbohydrate | 5/32 | 234/18614 | 4.59E-05 | 0.00027 | 8.15E-05 | IL1B/PTGS2/CASP3/HIF1A/IL1A | 5 |
| BP | GO:0043406 | positive regulation of MAP kinase activity | 4/32 | 117/18614 | 4.65E-05 | 0.000272 | 8.22E-05 | IL1B/EGFR/EGF/ERBB2 | 4 |
| BP | GO:1901800 | positive regulation of proteasomal protein catabolic process | 4/32 | 117/18614 | 4.65E-05 | 0.000272 | 8.22E-05 | AKT1/GSK3B/NFE2L2/CAV1 | 4 |
| BP | GO:0034764 | positive regulation of transmembrane transport | 5/32 | 235/18614 | 4.69E-05 | 0.000273 | 8.26E-05 | AKT1/CCL2/IFNG/NFE2L2/CAV1 | 5 |
| BP | GO:0048246 | macrophage chemotaxis | 3/32 | 41/18614 | 4.71E-05 | 0.000273 | 8.26E-05 | CCL2/MMP2/MAPK1 | 3 |
| BP | GO:0150077 | regulation of neuroinflammatory response | 3/32 | 41/18614 | 4.71E-05 | 0.000273 | 8.26E-05 | IL1B/PTGS2/MMP9 | 3 |
| BP | GO:0010466 | negative regulation of peptidase activity | 5/32 | 236/18614 | 4.78E-05 | 0.000277 | 8.38E-05 | AKT1/PTGS2/MMP9/APP/SERPINE1 | 5 |
| BP | GO:0021782 | glial cell development | 4/32 | 119/18614 | 4.97E-05 | 0.000288 | 8.69E-05 | AKT1/IL1B/IFNG/APP | 4 |
| BP | GO:0007595 | lactation | 3/32 | 42/18614 | 5.06E-05 | 0.00029 | 8.76E-05 | HIF1A/CCND1/CAV1 | 3 |
| BP | GO:0045981 | positive regulation of nucleotide metabolic process | 3/32 | 42/18614 | 5.06E-05 | 0.00029 | 8.76E-05 | HIF1A/IFNG/APP | 3 |
| BP | GO:0046688 | response to copper ion | 3/32 | 42/18614 | 5.06E-05 | 0.00029 | 8.76E-05 | IL1A/NFE2L2/APP | 3 |
| BP | GO:1900544 | positive regulation of purine nucleotide metabolic process | 3/32 | 42/18614 | 5.06E-05 | 0.00029 | 8.76E-05 | HIF1A/IFNG/APP | 3 |
| BP | GO:1904031 | positive regulation of cyclin-dependent protein kinase activity | 3/32 | 42/18614 | 5.06E-05 | 0.00029 | 8.76E-05 | AKT1/EGFR/CCND1 | 3 |
| BP | GO:0043200 | response to amino acid | 4/32 | 120/18614 | 5.14E-05 | 0.000293 | 8.87E-05 | CASP3/EGFR/BCL2/MMP2 | 4 |
| BP | GO:0002274 | myeloid leukocyte activation | 5/32 | 240/18614 | 5.18E-05 | 0.000295 | 8.92E-05 | IL10/IFNG/CXCL8/HMOX1/APP | 5 |
| BP | GO:0001701 | in utero embryonic development | 6/32 | 398/18614 | 5.21E-05 | 0.000296 | 8.95E-05 | AKT1/TP53/EGFR/HIF1A/IL10/MAPK1 | 6 |
| BP | GO:1901989 | positive regulation of cell cycle phase transition | 4/32 | 121/18614 | 5.31E-05 | 0.000301 | 9.10E-05 | AKT1/EGFR/CCND1/APP | 4 |
| BP | GO:0014002 | astrocyte development | 3/32 | 43/18614 | 5.44E-05 | 0.000307 | 9.29E-05 | IL1B/IFNG/APP | 3 |
| BP | GO:0045840 | positive regulation of mitotic nuclear division | 3/32 | 43/18614 | 5.44E-05 | 0.000307 | 9.29E-05 | IL1B/EGF/IL1A | 3 |
| BP | GO:0046660 | female sex differentiation | 4/32 | 122/18614 | 5.48E-05 | 0.000309 | 9.34E-05 | ESR1/CASP3/BCL2/MMP2 | 4 |
| BP | GO:0045930 | negative regulation of mitotic cell cycle | 5/32 | 243/18614 | 5.49E-05 | 0.000309 | 9.35E-05 | TP53/BCL2/CCND1/IL10/CCL2 | 5 |
| BP | GO:0006509 | membrane protein ectodomain proteolysis | 3/32 | 44/18614 | 5.83E-05 | 0.000325 | 9.83E-05 | IL1B/IL10/IFNG | 3 |
| BP | GO:0032881 | regulation of polysaccharide metabolic process | 3/32 | 44/18614 | 5.83E-05 | 0.000325 | 9.83E-05 | AKT1/GSK3B/EGF | 3 |
| BP | GO:0043029 | T cell homeostasis | 3/32 | 44/18614 | 5.83E-05 | 0.000325 | 9.83E-05 | AKT1/CASP3/BCL2 | 3 |
| BP | GO:1904646 | cellular response to amyloid-beta | 3/32 | 44/18614 | 5.83E-05 | 0.000325 | 9.83E-05 | GSK3B/ICAM1/APP | 3 |
| BP | GO:0071695 | anatomical structure maturation | 5/32 | 247/18614 | 5.94E-05 | 0.000331 | 1.00E-04 | BCL2/HIF1A/PPARG/MMP2/APP | 5 |
| BP | GO:0002688 | regulation of leukocyte chemotaxis | 4/32 | 126/18614 | 6.21E-05 | 0.000345 | 0.000104 | CCL2/CXCL8/MAPK1/SERPINE1 | 4 |
| BP | GO:0010799 | regulation of peptidyl-threonine phosphorylation | 3/32 | 45/18614 | 6.24E-05 | 0.000345 | 0.000104 | EGF/APP/MAPK1 | 3 |
| BP | GO:1904706 | negative regulation of vascular associated smooth muscle cell proliferation | 3/32 | 45/18614 | 6.24E-05 | 0.000345 | 0.000104 | PPARG/IL10/HMOX1 | 3 |
| BP | GO:0006959 | humoral immune response | 5/32 | 250/18614 | 6.29E-05 | 0.000347 | 0.000105 | IL1B/BCL2/CCL2/IFNG/CXCL8 | 5 |
| BP | GO:0031330 | negative regulation of cellular catabolic process | 5/32 | 251/18614 | 6.41E-05 | 0.000353 | 0.000107 | AKT1/TP53/BCL2/IL10/HMOX1 | 5 |
| BP | GO:1903706 | regulation of hemopoiesis | 6/32 | 415/18614 | 6.57E-05 | 0.000361 | 0.000109 | HIF1A/FOS/MYC/ERBB2/IL10/IFNG | 6 |
| BP | GO:0008286 | insulin receptor signaling pathway | 4/32 | 128/18614 | 6.61E-05 | 0.000362 | 0.000109 | AKT1/IL1B/GSK3B/MAPK1 | 4 |
| BP | GO:1990266 | neutrophil migration | 4/32 | 128/18614 | 6.61E-05 | 0.000362 | 0.000109 | IL1B/CCL2/CXCL8/IL1A | 4 |
| BP | GO:0045931 | positive regulation of mitotic cell cycle | 4/32 | 129/18614 | 6.81E-05 | 0.000372 | 0.000112 | AKT1/EGFR/CCND1/APP | 4 |
| BP | GO:0046683 | response to organophosphorus | 4/32 | 129/18614 | 6.81E-05 | 0.000372 | 0.000112 | IL1B/PTGS2/FOS/APP | 4 |
| BP | GO:0048762 | mesenchymal cell differentiation | 5/32 | 255/18614 | 6.90E-05 | 0.000376 | 0.000114 | IL1B/BCL2/HIF1A/GSK3B/MAPK1 | 5 |
| BP | GO:0001558 | regulation of cell growth | 6/32 | 420/18614 | 7.02E-05 | 0.000381 | 0.000115 | AKT1/TP53/EGFR/BCL2/GSK3B/ERBB2 | 6 |
| BP | GO:0006984 | ER-nucleus signaling pathway | 3/32 | 47/18614 | 7.11E-05 | 0.000382 | 0.000115 | TP53/GSK3B/NFE2L2 | 3 |
| BP | GO:0046677 | response to antibiotic | 3/32 | 47/18614 | 7.11E-05 | 0.000382 | 0.000115 | TP53/CASP3/CASP9 | 3 |
| BP | GO:0051972 | regulation of telomerase activity | 3/32 | 47/18614 | 7.11E-05 | 0.000382 | 0.000115 | TP53/MYC/MAPK1 | 3 |
| BP | GO:0060711 | labyrinthine layer development | 3/32 | 47/18614 | 7.11E-05 | 0.000382 | 0.000115 | AKT1/IL10/MAPK1 | 3 |
| BP | GO:0090199 | regulation of release of cytochrome c from mitochondria | 3/32 | 47/18614 | 7.11E-05 | 0.000382 | 0.000115 | AKT1/TP53/MMP9 | 3 |
| BP | GO:0140353 | lipid export from cell | 3/32 | 47/18614 | 7.11E-05 | 0.000382 | 0.000115 | IL1B/PTGS2/IL1A | 3 |
| BP | GO:0071621 | granulocyte chemotaxis | 4/32 | 131/18614 | 7.23E-05 | 0.000388 | 0.000117 | IL1B/CCL2/CXCL8/MAPK1 | 4 |
| BP | GO:0043122 | regulation of I-kappaB kinase/NF-kappaB signaling | 5/32 | 258/18614 | 7.30E-05 | 0.00039 | 0.000118 | IL1B/ESR1/IL1A/RELA/HMOX1 | 5 |
| BP | GO:0006110 | regulation of glycolytic process | 3/32 | 48/18614 | 7.57E-05 | 0.000402 | 0.000122 | HIF1A/IFNG/APP | 3 |
| BP | GO:0006953 | acute-phase response | 3/32 | 48/18614 | 7.57E-05 | 0.000402 | 0.000122 | IL1B/PTGS2/IL1A | 3 |
| BP | GO:0032309 | icosanoid secretion | 3/32 | 48/18614 | 7.57E-05 | 0.000402 | 0.000122 | IL1B/PTGS2/IL1A | 3 |
| BP | GO:1902003 | regulation of amyloid-beta formation | 3/32 | 48/18614 | 7.57E-05 | 0.000402 | 0.000122 | CASP3/IFNG/RELA | 3 |
| BP | GO:0048565 | digestive tract development | 4/32 | 134/18614 | 7.90E-05 | 0.000417 | 0.000126 | EGFR/BCL2/HIF1A/CXCL8 | 4 |
| BP | GO:1900180 | regulation of protein localization to nucleus | 4/32 | 134/18614 | 7.90E-05 | 0.000417 | 0.000126 | AKT1/PTGS2/GSK3B/IFNG | 4 |
| BP | GO:0042149 | cellular response to glucose starvation | 3/32 | 49/18614 | 8.06E-05 | 0.000425 | 0.000129 | TP53/BCL2/NFE2L2 | 3 |
| BP | GO:0010565 | regulation of cellular ketone metabolic process | 4/32 | 135/18614 | 8.13E-05 | 0.000428 | 0.000129 | AKT1/IL1B/PTGS2/CAV1 | 4 |
| BP | GO:0010720 | positive regulation of cell development | 6/32 | 432/18614 | 8.20E-05 | 0.000431 | 0.00013 | IL1B/BCL2/HIF1A/FOS/IL10/IFNG | 6 |
| BP | GO:0001101 | response to acid chemical | 4/32 | 136/18614 | 8.36E-05 | 0.000439 | 0.000133 | CASP3/EGFR/BCL2/MMP2 | 4 |
| BP | GO:0051924 | regulation of calcium ion transport | 5/32 | 266/18614 | 8.43E-05 | 0.000441 | 0.000133 | PTGS2/BCL2/EGF/CCL2/CAV1 | 5 |
| BP | GO:0002066 | columnar/cuboidal epithelial cell development | 3/32 | 50/18614 | 8.56E-05 | 0.000446 | 0.000135 | AKT1/HIF1A/GSK3B | 3 |
| BP | GO:0048546 | digestive tract morphogenesis | 3/32 | 50/18614 | 8.56E-05 | 0.000446 | 0.000135 | EGFR/BCL2/HIF1A | 3 |
| BP | GO:2000378 | negative regulation of reactive oxygen species metabolic process | 3/32 | 50/18614 | 8.56E-05 | 0.000446 | 0.000135 | TP53/BCL2/HIF1A | 3 |
| BP | GO:1904707 | positive regulation of vascular associated smooth muscle cell proliferation | 3/32 | 51/18614 | 9.09E-05 | 0.000471 | 0.000142 | MMP9/IL10/MMP2 | 3 |
| BP | GO:0034341 | response to type II interferon | 4/32 | 139/18614 | 9.10E-05 | 0.000471 | 0.000142 | TP53/PPARG/CCL2/IFNG | 4 |
| BP | GO:0046328 | regulation of JNK cascade | 4/32 | 139/18614 | 9.10E-05 | 0.000471 | 0.000142 | IL1B/EGFR/IL1A/APP | 4 |
| BP | GO:0043271 | negative regulation of monoatomic ion transport | 4/32 | 140/18614 | 9.36E-05 | 0.000483 | 0.000146 | PTGS2/MMP9/BCL2/CAV1 | 4 |
| BP | GO:0032434 | regulation of proteasomal ubiquitin-dependent protein catabolic process | 4/32 | 141/18614 | 9.62E-05 | 0.000496 | 0.00015 | AKT1/GSK3B/NFE2L2/CAV1 | 4 |
| BP | GO:0022602 | ovulation cycle process | 3/32 | 52/18614 | 9.63E-05 | 0.000496 | 0.00015 | ESR1/CASP3/MMP2 | 3 |
| BP | GO:0050708 | regulation of protein secretion | 5/32 | 275/18614 | 9.86E-05 | 0.000506 | 0.000153 | IL1B/HIF1A/PPARG/IFNG/IL1A | 5 |
| BP | GO:0001889 | liver development | 4/32 | 142/18614 | 9.89E-05 | 0.000507 | 0.000153 | CCND1/IL10/RELA/HMOX1 | 4 |
| BP | GO:0014074 | response to purine-containing compound | 4/32 | 143/18614 | 0.000102 | 0.000519 | 0.000157 | IL1B/PTGS2/FOS/APP | 4 |
| BP | GO:0010823 | negative regulation of mitochondrion organization | 3/32 | 53/18614 | 0.000102 | 0.000519 | 0.000157 | AKT1/TP53/PPARG | 3 |
| BP | GO:0010874 | regulation of cholesterol efflux | 3/32 | 53/18614 | 0.000102 | 0.000519 | 0.000157 | PPARG/EGF/CAV1 | 3 |
| BP | GO:0048146 | positive regulation of fibroblast proliferation | 3/32 | 53/18614 | 0.000102 | 0.000519 | 0.000157 | ESR1/EGFR/MYC | 3 |
| BP | GO:0032412 | regulation of monoatomic ion transmembrane transporter activity | 5/32 | 278/18614 | 0.000104 | 0.000527 | 0.000159 | MMP9/CCL2/IFNG/APP/CAV1 | 5 |
| BP | GO:0008584 | male gonad development | 4/32 | 144/18614 | 0.000104 | 0.000528 | 0.00016 | ESR1/BCL2/CCND1/IL1A | 4 |
| BP | GO:0014065 | phosphatidylinositol 3-kinase signaling | 4/32 | 144/18614 | 0.000104 | 0.000528 | 0.00016 | AKT1/EGFR/EGF/ERBB2 | 4 |
| BP | GO:0046546 | development of primary male sexual characteristics | 4/32 | 145/18614 | 0.000107 | 0.00054 | 0.000163 | ESR1/BCL2/CCND1/IL1A | 4 |
| BP | GO:0061008 | hepaticobiliary system development | 4/32 | 145/18614 | 0.000107 | 0.00054 | 0.000163 | CCND1/IL10/RELA/HMOX1 | 4 |
| BP | GO:0010508 | positive regulation of autophagy | 4/32 | 146/18614 | 0.00011 | 0.000553 | 0.000167 | HIF1A/GSK3B/IFNG/HMOX1 | 4 |
| BP | GO:0055123 | digestive system development | 4/32 | 146/18614 | 0.00011 | 0.000553 | 0.000167 | EGFR/BCL2/HIF1A/CXCL8 | 4 |
| BP | GO:0002686 | negative regulation of leukocyte migration | 3/32 | 55/18614 | 0.000114 | 0.000567 | 0.000171 | AKT1/CCL2/HMOX1 | 3 |
| BP | GO:0043525 | positive regulation of neuron apoptotic process | 3/32 | 55/18614 | 0.000114 | 0.000567 | 0.000171 | TP53/CASP3/CASP9 | 3 |
| BP | GO:0061900 | glial cell activation | 3/32 | 55/18614 | 0.000114 | 0.000567 | 0.000171 | IL1B/IFNG/APP | 3 |
| BP | GO:1900087 | positive regulation of G1/S transition of mitotic cell cycle | 3/32 | 55/18614 | 0.000114 | 0.000567 | 0.000171 | AKT1/EGFR/CCND1 | 3 |
| BP | GO:1902991 | regulation of amyloid precursor protein catabolic process | 3/32 | 55/18614 | 0.000114 | 0.000567 | 0.000171 | CASP3/IFNG/RELA | 3 |
| BP | GO:0016055 | Wnt signaling pathway | 6/32 | 461/18614 | 0.000117 | 0.000582 | 0.000176 | EGFR/GSK3B/CCND1/EGF/APP/CAV1 | 6 |
| BP | GO:0002831 | regulation of response to biotic stimulus | 6/32 | 463/18614 | 0.00012 | 0.000594 | 0.000179 | IL1B/ESR1/PPARG/RELA/NFE2L2/CAV1 | 6 |
| BP | GO:0198738 | cell-cell signaling by wnt | 6/32 | 463/18614 | 0.00012 | 0.000594 | 0.000179 | EGFR/GSK3B/CCND1/EGF/APP/CAV1 | 6 |
| BP | GO:0034205 | amyloid-beta formation | 3/32 | 56/18614 | 0.00012 | 0.000594 | 0.00018 | CASP3/IFNG/RELA | 3 |
| BP | GO:1903038 | negative regulation of leukocyte cell-cell adhesion | 4/32 | 150/18614 | 0.000122 | 0.000602 | 0.000182 | AKT1/CASP3/ERBB2/IL10 | 4 |
| BP | GO:2000377 | regulation of reactive oxygen species metabolic process | 4/32 | 150/18614 | 0.000122 | 0.000602 | 0.000182 | TP53/BCL2/HIF1A/NFE2L2 | 4 |
| BP | GO:1903532 | positive regulation of secretion by cell | 5/32 | 289/18614 | 0.000124 | 0.000611 | 0.000185 | IL1B/HIF1A/PPARG/IFNG/IL1A | 5 |
| BP | GO:0001541 | ovarian follicle development | 3/32 | 57/18614 | 0.000127 | 0.000622 | 0.000188 | ESR1/BCL2/MMP2 | 3 |
| BP | GO:0008627 | intrinsic apoptotic signaling pathway in response to osmotic stress | 2/32 | 10/18614 | 0.000128 | 0.000623 | 0.000188 | PTGS2/CASP3 | 2 |
| BP | GO:0032025 | response to cobalt ion | 2/32 | 10/18614 | 0.000128 | 0.000623 | 0.000188 | CASP3/CASP9 | 2 |
| BP | GO:1903596 | regulation of gap junction assembly | 2/32 | 10/18614 | 0.000128 | 0.000623 | 0.000188 | IL1B/CAV1 | 2 |
| BP | GO:0007612 | learning | 4/32 | 152/18614 | 0.000129 | 0.000626 | 0.000189 | PTGS2/HIF1A/FOS/APP | 4 |
| BP | GO:0030225 | macrophage differentiation | 3/32 | 58/18614 | 0.000134 | 0.000649 | 0.000196 | MMP9/IFNG/APP | 3 |
| BP | GO:0002366 | leukocyte activation involved in immune response | 5/32 | 296/18614 | 0.000139 | 0.000675 | 0.000204 | TP53/IL10/IFNG/ICAM1/HMOX1 | 5 |
| BP | GO:0033619 | membrane protein proteolysis | 3/32 | 59/18614 | 0.000141 | 0.000679 | 0.000205 | IL1B/IL10/IFNG | 3 |
| BP | GO:0051785 | positive regulation of nuclear division | 3/32 | 59/18614 | 0.000141 | 0.000679 | 0.000205 | IL1B/EGF/IL1A | 3 |
| BP | GO:0061564 | axon development | 6/32 | 479/18614 | 0.000144 | 0.000697 | 0.000211 | CASP3/BCL2/GSK3B/ERBB2/MMP2/APP | 6 |
| BP | GO:0048754 | branching morphogenesis of an epithelial tube | 4/32 | 157/18614 | 0.000146 | 0.000702 | 0.000212 | ESR1/BCL2/MYC/EGF | 4 |
| BP | GO:0043407 | negative regulation of MAP kinase activity | 3/32 | 60/18614 | 0.000148 | 0.000709 | 0.000214 | IL1B/PPARG/CAV1 | 3 |
| BP | GO:1903078 | positive regulation of protein localization to plasma membrane | 3/32 | 60/18614 | 0.000148 | 0.000709 | 0.000214 | AKT1/EGFR/IFNG | 3 |
| BP | GO:0023061 | signal release | 6/32 | 483/18614 | 0.000151 | 0.000724 | 0.000219 | IL1B/PTGS2/HIF1A/PPARG/IFNG/IL1A | 6 |
| BP | GO:0051092 | positive regulation of NF-kappaB transcription factor activity | 4/32 | 159/18614 | 0.000153 | 0.000732 | 0.000221 | IL1B/RELA/APP/CAV1 | 4 |
| BP | GO:0019229 | regulation of vasoconstriction | 3/32 | 61/18614 | 0.000155 | 0.000737 | 0.000223 | PTGS2/MMP2/CAV1 | 3 |
| BP | GO:0060135 | maternal process involved in female pregnancy | 3/32 | 61/18614 | 0.000155 | 0.000737 | 0.000223 | AKT1/PTGS2/ESR1 | 3 |
| BP | GO:1905517 | macrophage migration | 3/32 | 61/18614 | 0.000155 | 0.000737 | 0.000223 | CCL2/MMP2/MAPK1 | 3 |
| BP | GO:0051054 | positive regulation of DNA metabolic process | 5/32 | 303/18614 | 0.000155 | 0.000737 | 0.000223 | AKT1/EGFR/MYC/EGF/MAPK1 | 5 |
| BP | GO:0071492 | cellular response to UV-A | 2/32 | 11/18614 | 0.000156 | 0.000738 | 0.000223 | MMP9/MMP2 | 2 |
| BP | GO:0072584 | caveolin-mediated endocytosis | 2/32 | 11/18614 | 0.000156 | 0.000738 | 0.000223 | MAPK1/CAV1 | 2 |
| BP | GO:0010951 | negative regulation of endopeptidase activity | 4/32 | 160/18614 | 0.000157 | 0.000739 | 0.000223 | AKT1/PTGS2/MMP9/SERPINE1 | 4 |
| BP | GO:0044409 | entry into host | 4/32 | 160/18614 | 0.000157 | 0.000739 | 0.000223 | EGFR/CXCL8/ICAM1/CAV1 | 4 |
| BP | GO:0034504 | protein localization to nucleus | 5/32 | 304/18614 | 0.000158 | 0.000741 | 0.000224 | AKT1/TP53/PTGS2/GSK3B/IFNG | 5 |
| BP | GO:0048872 | homeostasis of number of cells | 5/32 | 304/18614 | 0.000158 | 0.000741 | 0.000224 | AKT1/CASP3/BCL2/HIF1A/HMOX1 | 5 |
| BP | GO:0050804 | modulation of chemical synaptic transmission | 6/32 | 487/18614 | 0.000158 | 0.000741 | 0.000224 | IL1B/PTGS2/GSK3B/CCL2/APP/MAPK1 | 6 |
| BP | GO:0022411 | cellular component disassembly | 6/32 | 488/18614 | 0.00016 | 0.000747 | 0.000226 | TP53/MMP9/HIF1A/MYC/GSK3B/MMP2 | 6 |
| BP | GO:0099177 | regulation of trans-synaptic signaling | 6/32 | 488/18614 | 0.00016 | 0.000747 | 0.000226 | IL1B/PTGS2/GSK3B/CCL2/APP/MAPK1 | 6 |
| BP | GO:0046902 | regulation of mitochondrial membrane permeability | 3/32 | 62/18614 | 0.000163 | 0.00076 | 0.00023 | TP53/BCL2/GSK3B | 3 |
| BP | GO:0016052 | carbohydrate catabolic process | 4/32 | 162/18614 | 0.000164 | 0.000766 | 0.000232 | TP53/HIF1A/IFNG/APP | 4 |
| BP | GO:0043588 | skin development | 5/32 | 308/18614 | 0.000168 | 0.000779 | 0.000236 | CASP3/EGFR/BCL2/IL1A/RELA | 5 |
| BP | GO:0006606 | protein import into nucleus | 4/32 | 163/18614 | 0.000168 | 0.000782 | 0.000236 | AKT1/TP53/PTGS2/IFNG | 4 |
| BP | GO:0032757 | positive regulation of interleukin-8 production | 3/32 | 63/18614 | 0.000171 | 0.000789 | 0.000239 | IL1B/RELA/SERPINE1 | 3 |
| BP | GO:0099601 | regulation of neurotransmitter receptor activity | 3/32 | 63/18614 | 0.000171 | 0.000789 | 0.000239 | CCL2/IFNG/APP | 3 |
| BP | GO:2001244 | positive regulation of intrinsic apoptotic signaling pathway | 3/32 | 63/18614 | 0.000171 | 0.000789 | 0.000239 | TP53/MYC/CAV1 | 3 |
| BP | GO:0030307 | positive regulation of cell growth | 4/32 | 164/18614 | 0.000172 | 0.000795 | 0.00024 | AKT1/EGFR/BCL2/ERBB2 | 4 |
| BP | GO:0002253 | activation of immune response | 6/32 | 495/18614 | 0.000173 | 0.000795 | 0.00024 | IL1B/ESR1/BCL2/RELA/MAPK1/CAV1 | 6 |
| BP | GO:2001020 | regulation of response to DNA damage stimulus | 5/32 | 311/18614 | 0.000175 | 0.000806 | 0.000244 | TP53/EGFR/BCL2/MYC/CASP9 | 5 |
| BP | GO:0032615 | interleukin-12 production | 3/32 | 64/18614 | 0.000179 | 0.000819 | 0.000248 | IL10/IFNG/RELA | 3 |
| BP | GO:0032655 | regulation of interleukin-12 production | 3/32 | 64/18614 | 0.000179 | 0.000819 | 0.000248 | IL10/IFNG/RELA | 3 |
| BP | GO:1905953 | negative regulation of lipid localization | 3/32 | 64/18614 | 0.000179 | 0.000819 | 0.000248 | AKT1/PPARG/EGF | 3 |
| BP | GO:0070665 | positive regulation of leukocyte proliferation | 4/32 | 166/18614 | 0.000181 | 0.000824 | 0.000249 | IL1B/BCL2/IL1A/MAPK1 | 4 |
| BP | GO:0006983 | ER overload response | 2/32 | 12/18614 | 0.000187 | 0.000849 | 0.000257 | TP53/GSK3B | 2 |
| BP | GO:0031650 | regulation of heat generation | 2/32 | 12/18614 | 0.000187 | 0.000849 | 0.000257 | IL1B/PTGS2 | 2 |
| BP | GO:0030888 | regulation of B cell proliferation | 3/32 | 65/18614 | 0.000188 | 0.000849 | 0.000257 | CASP3/BCL2/IL10 | 3 |
| BP | GO:0050435 | amyloid-beta metabolic process | 3/32 | 65/18614 | 0.000188 | 0.000849 | 0.000257 | CASP3/IFNG/RELA | 3 |
| BP | GO:0071715 | icosanoid transport | 3/32 | 65/18614 | 0.000188 | 0.000849 | 0.000257 | IL1B/PTGS2/IL1A | 3 |
| BP | GO:0046661 | male sex differentiation | 4/32 | 168/18614 | 0.000189 | 0.000853 | 0.000258 | ESR1/BCL2/CCND1/IL1A | 4 |
| BP | GO:0051170 | import into nucleus | 4/32 | 168/18614 | 0.000189 | 0.000853 | 0.000258 | AKT1/TP53/PTGS2/IFNG | 4 |
| BP | GO:0051047 | positive regulation of secretion | 5/32 | 317/18614 | 0.000192 | 0.000863 | 0.000261 | IL1B/HIF1A/PPARG/IFNG/IL1A | 5 |
| BP | GO:0031100 | animal organ regeneration | 3/32 | 66/18614 | 0.000196 | 0.000882 | 0.000267 | CCND1/IL10/HMOX1 | 3 |
| BP | GO:0021700 | developmental maturation | 5/32 | 319/18614 | 0.000197 | 0.000884 | 0.000267 | BCL2/HIF1A/PPARG/MMP2/APP | 5 |
| BP | GO:1902105 | regulation of leukocyte differentiation | 5/32 | 319/18614 | 0.000197 | 0.000884 | 0.000267 | FOS/MYC/ERBB2/IL10/IFNG | 5 |
| BP | GO:0007254 | JNK cascade | 4/32 | 170/18614 | 0.000198 | 0.000884 | 0.000267 | IL1B/EGFR/IL1A/APP | 4 |
| BP | GO:1903034 | regulation of response to wounding | 4/32 | 170/18614 | 0.000198 | 0.000884 | 0.000267 | IL10/NFE2L2/SERPINE1/CAV1 | 4 |
| BP | GO:0016236 | macroautophagy | 5/32 | 322/18614 | 0.000206 | 0.000919 | 0.000278 | AKT1/TP53/CASP3/HIF1A/HMOX1 | 5 |
| BP | GO:0048015 | phosphatidylinositol-mediated signaling | 4/32 | 172/18614 | 0.000207 | 0.000921 | 0.000279 | AKT1/EGFR/EGF/ERBB2 | 4 |
| BP | GO:0072331 | signal transduction by p53 class mediator | 4/32 | 173/18614 | 0.000211 | 0.00094 | 0.000284 | AKT1/TP53/BCL2/MYC | 4 |
| BP | GO:0042987 | amyloid precursor protein catabolic process | 3/32 | 68/18614 | 0.000214 | 0.000947 | 0.000286 | CASP3/IFNG/RELA | 3 |
| BP | GO:1902808 | positive regulation of cell cycle G1/S phase transition | 3/32 | 68/18614 | 0.000214 | 0.000947 | 0.000286 | AKT1/EGFR/CCND1 | 3 |
| BP | GO:1904377 | positive regulation of protein localization to cell periphery | 3/32 | 68/18614 | 0.000214 | 0.000947 | 0.000286 | AKT1/EGFR/IFNG | 3 |
| BP | GO:2000401 | regulation of lymphocyte migration | 3/32 | 68/18614 | 0.000214 | 0.000947 | 0.000286 | AKT1/CCL2/APP | 3 |
| BP | GO:0031392 | regulation of prostaglandin biosynthetic process | 2/32 | 13/18614 | 0.000221 | 0.000971 | 0.000294 | IL1B/PTGS2 | 2 |
| BP | GO:0070243 | regulation of thymocyte apoptotic process | 2/32 | 13/18614 | 0.000221 | 0.000971 | 0.000294 | TP53/HIF1A | 2 |
| BP | GO:1905475 | regulation of protein localization to membrane | 4/32 | 175/18614 | 0.000221 | 0.000971 | 0.000294 | AKT1/EGFR/ERBB2/IFNG | 4 |
| BP | GO:0048017 | inositol lipid-mediated signaling | 4/32 | 176/18614 | 0.000226 | 0.000991 | 0.0003 | AKT1/EGFR/EGF/ERBB2 | 4 |
| BP | GO:0000271 | polysaccharide biosynthetic process | 3/32 | 70/18614 | 0.000234 | 0.001022 | 0.000309 | AKT1/GSK3B/EGF | 3 |
| BP | GO:0030193 | regulation of blood coagulation | 3/32 | 70/18614 | 0.000234 | 0.001022 | 0.000309 | NFE2L2/SERPINE1/CAV1 | 3 |
| BP | GO:0001659 | temperature homeostasis | 4/32 | 179/18614 | 0.000241 | 0.001052 | 0.000318 | IL1B/PTGS2/IL1A/CAV1 | 4 |
| BP | GO:0033344 | cholesterol efflux | 3/32 | 71/18614 | 0.000244 | 0.001061 | 0.000321 | PPARG/EGF/CAV1 | 3 |
| BP | GO:0045600 | positive regulation of fat cell differentiation | 3/32 | 71/18614 | 0.000244 | 0.001061 | 0.000321 | AKT1/PTGS2/PPARG | 3 |
| BP | GO:0042130 | negative regulation of T cell proliferation | 3/32 | 72/18614 | 0.000254 | 0.001102 | 0.000333 | CASP3/ERBB2/IL10 | 3 |
| BP | GO:1900046 | regulation of hemostasis | 3/32 | 72/18614 | 0.000254 | 0.001102 | 0.000333 | NFE2L2/SERPINE1/CAV1 | 3 |
| BP | GO:0030213 | hyaluronan biosynthetic process | 2/32 | 14/18614 | 0.000257 | 0.001107 | 0.000335 | IL1B/EGF | 2 |
| BP | GO:0032306 | regulation of prostaglandin secretion | 2/32 | 14/18614 | 0.000257 | 0.001107 | 0.000335 | IL1B/IL1A | 2 |
| BP | GO:0032308 | positive regulation of prostaglandin secretion | 2/32 | 14/18614 | 0.000257 | 0.001107 | 0.000335 | IL1B/IL1A | 2 |
| BP | GO:0045348 | positive regulation of MHC class II biosynthetic process | 2/32 | 14/18614 | 0.000257 | 0.001107 | 0.000335 | IL10/IFNG | 2 |
| BP | GO:1904294 | positive regulation of ERAD pathway | 2/32 | 14/18614 | 0.000257 | 0.001107 | 0.000335 | NFE2L2/CAV1 | 2 |
| BP | GO:0030111 | regulation of Wnt signaling pathway | 5/32 | 338/18614 | 0.000258 | 0.001108 | 0.000335 | EGFR/GSK3B/EGF/APP/CAV1 | 5 |
| BP | GO:0043409 | negative regulation of MAPK cascade | 4/32 | 183/18614 | 0.000262 | 0.001125 | 0.00034 | IL1B/MYC/PPARG/CAV1 | 4 |
| BP | GO:0050766 | positive regulation of phagocytosis | 3/32 | 73/18614 | 0.000265 | 0.001133 | 0.000343 | IL1B/CCL2/IFNG | 3 |
| BP | GO:0021543 | pallium development | 4/32 | 184/18614 | 0.000268 | 0.001143 | 0.000346 | CASP3/EGFR/HIF1A/GSK3B | 4 |
| BP | GO:0048469 | cell maturation | 4/32 | 184/18614 | 0.000268 | 0.001143 | 0.000346 | BCL2/HIF1A/PPARG/APP | 4 |
| BP | GO:0032640 | tumor necrosis factor production | 4/32 | 185/18614 | 0.000273 | 0.001161 | 0.000351 | IL10/IFNG/IL1A/APP | 4 |
| BP | GO:0032680 | regulation of tumor necrosis factor production | 4/32 | 185/18614 | 0.000273 | 0.001161 | 0.000351 | IL10/IFNG/IL1A/APP | 4 |
| BP | GO:0044000 | movement in host | 4/32 | 185/18614 | 0.000273 | 0.001161 | 0.000351 | EGFR/CXCL8/ICAM1/CAV1 | 4 |
| BP | GO:1903578 | regulation of ATP metabolic process | 3/32 | 74/18614 | 0.000275 | 0.001169 | 0.000353 | HIF1A/IFNG/APP | 3 |
| BP | GO:0090287 | regulation of cellular response to growth factor stimulus | 5/32 | 345/18614 | 0.000283 | 0.001199 | 0.000363 | IL1B/TP53/HIF1A/PPARG/CAV1 | 5 |
| BP | GO:1990778 | protein localization to cell periphery | 5/32 | 345/18614 | 0.000283 | 0.001199 | 0.000363 | AKT1/EGFR/ERBB2/IFNG/CAV1 | 5 |
| BP | GO:0042058 | regulation of epidermal growth factor receptor signaling pathway | 3/32 | 75/18614 | 0.000287 | 0.001207 | 0.000365 | MMP9/EGFR/EGF | 3 |
| BP | GO:0042698 | ovulation cycle | 3/32 | 75/18614 | 0.000287 | 0.001207 | 0.000365 | ESR1/CASP3/MMP2 | 3 |
| BP | GO:0050818 | regulation of coagulation | 3/32 | 75/18614 | 0.000287 | 0.001207 | 0.000365 | NFE2L2/SERPINE1/CAV1 | 3 |
| BP | GO:0002040 | sprouting angiogenesis | 4/32 | 188/18614 | 0.00029 | 0.001221 | 0.000369 | AKT1/PTGS2/IL10/HMOX1 | 4 |
| BP | GO:0051235 | maintenance of location | 5/32 | 347/18614 | 0.000291 | 0.001222 | 0.00037 | AKT1/IL1B/PPARG/IL10/CAV1 | 5 |
| BP | GO:0071466 | cellular response to xenobiotic stimulus | 4/32 | 189/18614 | 0.000296 | 0.001237 | 0.000374 | IL1B/TP53/MYC/NFE2L2 | 4 |
| BP | GO:0034116 | positive regulation of heterotypic cell-cell adhesion | 2/32 | 15/18614 | 0.000296 | 0.001237 | 0.000374 | IL1B/IL10 | 2 |
| BP | GO:2000402 | negative regulation of lymphocyte migration | 2/32 | 15/18614 | 0.000296 | 0.001237 | 0.000374 | AKT1/CCL2 | 2 |
| BP | GO:2001279 | regulation of unsaturated fatty acid biosynthetic process | 2/32 | 15/18614 | 0.000296 | 0.001237 | 0.000374 | IL1B/PTGS2 | 2 |
| BP | GO:0071706 | tumor necrosis factor superfamily cytokine production | 4/32 | 190/18614 | 0.000302 | 0.001258 | 0.00038 | IL10/IFNG/IL1A/APP | 4 |
| BP | GO:1903555 | regulation of tumor necrosis factor superfamily cytokine production | 4/32 | 190/18614 | 0.000302 | 0.001258 | 0.00038 | IL10/IFNG/IL1A/APP | 4 |
| BP | GO:0001937 | negative regulation of endothelial cell proliferation | 3/32 | 77/18614 | 0.00031 | 0.001282 | 0.000388 | PPARG/CCL2/CAV1 | 3 |
| BP | GO:0010827 | regulation of glucose transmembrane transport | 3/32 | 77/18614 | 0.00031 | 0.001282 | 0.000388 | AKT1/IL1B/NFE2L2 | 3 |
| BP | GO:0090559 | regulation of membrane permeability | 3/32 | 77/18614 | 0.00031 | 0.001282 | 0.000388 | TP53/BCL2/GSK3B | 3 |
| BP | GO:0046942 | carboxylic acid transport | 5/32 | 352/18614 | 0.000311 | 0.001286 | 0.000389 | AKT1/IL1B/PTGS2/PPARG/IL1A | 5 |
| BP | GO:0043123 | positive regulation of I-kappaB kinase/NF-kappaB signaling | 4/32 | 192/18614 | 0.000315 | 0.001299 | 0.000393 | IL1B/IL1A/RELA/HMOX1 | 4 |
| BP | GO:0015849 | organic acid transport | 5/32 | 353/18614 | 0.000315 | 0.001299 | 0.000393 | AKT1/IL1B/PTGS2/PPARG/IL1A | 5 |
| BP | GO:0043154 | negative regulation of cysteine-type endopeptidase activity involved in apoptotic process | 3/32 | 78/18614 | 0.000322 | 0.001324 | 0.0004 | AKT1/PTGS2/MMP9 | 3 |
| BP | GO:0061136 | regulation of proteasomal protein catabolic process | 4/32 | 194/18614 | 0.000327 | 0.001343 | 0.000406 | AKT1/GSK3B/NFE2L2/CAV1 | 4 |
| BP | GO:1901991 | negative regulation of mitotic cell cycle phase transition | 4/32 | 194/18614 | 0.000327 | 0.001343 | 0.000406 | TP53/BCL2/CCND1/CCL2 | 4 |
| BP | GO:0031334 | positive regulation of protein-containing complex assembly | 4/32 | 195/18614 | 0.000334 | 0.001362 | 0.000412 | TP53/ESR1/GSK3B/IFNG | 4 |
| BP | GO:0032371 | regulation of sterol transport | 3/32 | 79/18614 | 0.000334 | 0.001362 | 0.000412 | PPARG/EGF/CAV1 | 3 |
| BP | GO:0032374 | regulation of cholesterol transport | 3/32 | 79/18614 | 0.000334 | 0.001362 | 0.000412 | PPARG/EGF/CAV1 | 3 |
| BP | GO:0042310 | vasoconstriction | 3/32 | 79/18614 | 0.000334 | 0.001362 | 0.000412 | PTGS2/MMP2/CAV1 | 3 |
| BP | GO:0034393 | positive regulation of smooth muscle cell apoptotic process | 2/32 | 16/18614 | 0.000338 | 0.001374 | 0.000415 | PPARG/IFNG | 2 |
| BP | GO:0051044 | positive regulation of membrane protein ectodomain proteolysis | 2/32 | 16/18614 | 0.000338 | 0.001374 | 0.000415 | IL1B/IFNG | 2 |
| BP | GO:0070230 | positive regulation of lymphocyte apoptotic process | 2/32 | 16/18614 | 0.000338 | 0.001374 | 0.000415 | TP53/IL10 | 2 |
| BP | GO:0007369 | gastrulation | 4/32 | 196/18614 | 0.00034 | 0.00138 | 0.000417 | TP53/MMP9/IL10/MMP2 | 4 |
| BP | GO:0032890 | regulation of organic acid transport | 3/32 | 80/18614 | 0.000347 | 0.001402 | 0.000424 | AKT1/IL1B/IL1A | 3 |
| BP | GO:0002520 | immune system development | 4/32 | 197/18614 | 0.000347 | 0.001402 | 0.000424 | TP53/BCL2/IL10/MAPK1 | 4 |
| BP | GO:0050878 | regulation of body fluid levels | 5/32 | 361/18614 | 0.000349 | 0.001409 | 0.000426 | HIF1A/CCND1/NFE2L2/SERPINE1/CAV1 | 5 |
| BP | GO:0061097 | regulation of protein tyrosine kinase activity | 3/32 | 82/18614 | 0.000373 | 0.0015 | 0.000453 | EGF/APP/CAV1 | 3 |
| BP | GO:1901184 | regulation of ERBB signaling pathway | 3/32 | 82/18614 | 0.000373 | 0.0015 | 0.000453 | MMP9/EGFR/EGF | 3 |
| BP | GO:0002695 | negative regulation of leukocyte activation | 4/32 | 202/18614 | 0.000382 | 0.001517 | 0.000459 | CASP3/ERBB2/IL10/HMOX1 | 4 |
| BP | GO:0006089 | lactate metabolic process | 2/32 | 17/18614 | 0.000383 | 0.001517 | 0.000459 | TP53/HIF1A | 2 |
| BP | GO:0030889 | negative regulation of B cell proliferation | 2/32 | 17/18614 | 0.000383 | 0.001517 | 0.000459 | CASP3/IL10 | 2 |
| BP | GO:0030949 | positive regulation of vascular endothelial growth factor receptor signaling pathway | 2/32 | 17/18614 | 0.000383 | 0.001517 | 0.000459 | IL1B/HIF1A | 2 |
| BP | GO:0035729 | cellular response to hepatocyte growth factor stimulus | 2/32 | 17/18614 | 0.000383 | 0.001517 | 0.000459 | IL10/RELA | 2 |
| BP | GO:0045019 | negative regulation of nitric oxide biosynthetic process | 2/32 | 17/18614 | 0.000383 | 0.001517 | 0.000459 | IL10/CAV1 | 2 |
| BP | GO:0046827 | positive regulation of protein export from nucleus | 2/32 | 17/18614 | 0.000383 | 0.001517 | 0.000459 | IL1B/GSK3B | 2 |
| BP | GO:0051412 | response to corticosterone | 2/32 | 17/18614 | 0.000383 | 0.001517 | 0.000459 | FOS/CCND1 | 2 |
| BP | GO:0060965 | negative regulation of miRNA-mediated gene silencing | 2/32 | 17/18614 | 0.000383 | 0.001517 | 0.000459 | TP53/PPARG | 2 |
| BP | GO:0090399 | replicative senescence | 2/32 | 17/18614 | 0.000383 | 0.001517 | 0.000459 | TP53/SERPINE1 | 2 |
| BP | GO:1904406 | negative regulation of nitric oxide metabolic process | 2/32 | 17/18614 | 0.000383 | 0.001517 | 0.000459 | IL10/CAV1 | 2 |
| BP | GO:0072332 | intrinsic apoptotic signaling pathway by p53 class mediator | 3/32 | 83/18614 | 0.000386 | 0.001527 | 0.000462 | TP53/BCL2/MYC | 3 |
| BP | GO:0022408 | negative regulation of cell-cell adhesion | 4/32 | 205/18614 | 0.000403 | 0.001593 | 0.000482 | AKT1/CASP3/ERBB2/IL10 | 4 |
| BP | GO:0009306 | protein secretion | 5/32 | 373/18614 | 0.000406 | 0.001597 | 0.000483 | IL1B/HIF1A/PPARG/IFNG/IL1A | 5 |
| BP | GO:0045088 | regulation of innate immune response | 5/32 | 373/18614 | 0.000406 | 0.001597 | 0.000483 | ESR1/PPARG/RELA/NFE2L2/CAV1 | 5 |
| BP | GO:0035592 | establishment of protein localization to extracellular region | 5/32 | 374/18614 | 0.000411 | 0.001613 | 0.000488 | IL1B/HIF1A/PPARG/IFNG/IL1A | 5 |
| BP | GO:0051701 | biological process involved in interaction with host | 4/32 | 206/18614 | 0.000411 | 0.001613 | 0.000488 | EGFR/CXCL8/ICAM1/CAV1 | 4 |
| BP | GO:0006096 | glycolytic process | 3/32 | 85/18614 | 0.000414 | 0.001623 | 0.000491 | HIF1A/IFNG/APP | 3 |
| BP | GO:0008544 | epidermis development | 5/32 | 375/18614 | 0.000416 | 0.001627 | 0.000492 | CASP3/EGFR/BCL2/IL1A/RELA | 5 |
| BP | GO:0034284 | response to monosaccharide | 4/32 | 207/18614 | 0.000419 | 0.001635 | 0.000495 | PTGS2/CASP3/HIF1A/IL1A | 4 |
| BP | GO:0002285 | lymphocyte activation involved in immune response | 4/32 | 208/18614 | 0.000426 | 0.001645 | 0.000497 | TP53/IL10/IFNG/ICAM1 | 4 |
| BP | GO:0006757 | ATP generation from ADP | 3/32 | 86/18614 | 0.000429 | 0.001645 | 0.000497 | HIF1A/IFNG/APP | 3 |
| BP | GO:0006970 | response to osmotic stress | 3/32 | 86/18614 | 0.000429 | 0.001645 | 0.000497 | TP53/PTGS2/CASP3 | 3 |
| BP | GO:0008306 | associative learning | 3/32 | 86/18614 | 0.000429 | 0.001645 | 0.000497 | HIF1A/FOS/APP | 3 |
| BP | GO:0019915 | lipid storage | 3/32 | 86/18614 | 0.000429 | 0.001645 | 0.000497 | IL1B/PPARG/CAV1 | 3 |
| BP | GO:1900182 | positive regulation of protein localization to nucleus | 3/32 | 86/18614 | 0.000429 | 0.001645 | 0.000497 | AKT1/PTGS2/IFNG | 3 |
| BP | GO:0010522 | regulation of calcium ion transport into cytosol | 2/32 | 18/18614 | 0.000431 | 0.001645 | 0.000497 | BCL2/CAV1 | 2 |
| BP | GO:0016264 | gap junction assembly | 2/32 | 18/18614 | 0.000431 | 0.001645 | 0.000497 | IL1B/CAV1 | 2 |
| BP | GO:0030730 | sequestering of triglyceride | 2/32 | 18/18614 | 0.000431 | 0.001645 | 0.000497 | IL1B/PPARG | 2 |
| BP | GO:0035234 | ectopic germ cell programmed cell death | 2/32 | 18/18614 | 0.000431 | 0.001645 | 0.000497 | IL1B/IL1A | 2 |
| BP | GO:0051770 | positive regulation of nitric-oxide synthase biosynthetic process | 2/32 | 18/18614 | 0.000431 | 0.001645 | 0.000497 | CCL2/IFNG | 2 |
| BP | GO:0060149 | negative regulation of post-transcriptional gene silencing | 2/32 | 18/18614 | 0.000431 | 0.001645 | 0.000497 | TP53/PPARG | 2 |
| BP | GO:0060644 | mammary gland epithelial cell differentiation | 2/32 | 18/18614 | 0.000431 | 0.001645 | 0.000497 | AKT1/HIF1A | 2 |
| BP | GO:0060967 | negative regulation of gene silencing by RNA | 2/32 | 18/18614 | 0.000431 | 0.001645 | 0.000497 | TP53/PPARG | 2 |
| BP | GO:1900369 | negative regulation of post-transcriptional gene silencing by RNA | 2/32 | 18/18614 | 0.000431 | 0.001645 | 0.000497 | TP53/PPARG | 2 |
| BP | GO:2000811 | negative regulation of anoikis | 2/32 | 18/18614 | 0.000431 | 0.001645 | 0.000497 | BCL2/CAV1 | 2 |
| BP | GO:0048167 | regulation of synaptic plasticity | 4/32 | 209/18614 | 0.000434 | 0.001655 | 0.000501 | PTGS2/GSK3B/APP/MAPK1 | 4 |
| BP | GO:0071692 | protein localization to extracellular region | 5/32 | 382/18614 | 0.000452 | 0.001723 | 0.000521 | IL1B/HIF1A/PPARG/IFNG/IL1A | 5 |
| BP | GO:0033273 | response to vitamin | 3/32 | 88/18614 | 0.000459 | 0.001739 | 0.000526 | PTGS2/CCND1/IL1A | 3 |
| BP | GO:0048864 | stem cell development | 3/32 | 88/18614 | 0.000459 | 0.001739 | 0.000526 | BCL2/HIF1A/MAPK1 | 3 |
| BP | GO:0050672 | negative regulation of lymphocyte proliferation | 3/32 | 88/18614 | 0.000459 | 0.001739 | 0.000526 | CASP3/ERBB2/IL10 | 3 |
| BP | GO:0002697 | regulation of immune effector process | 5/32 | 384/18614 | 0.000463 | 0.001755 | 0.000531 | IL1B/IL10/IFNG/ICAM1/HMOX1 | 5 |
| BP | GO:0006112 | energy reserve metabolic process | 3/32 | 89/18614 | 0.000474 | 0.001785 | 0.00054 | AKT1/MYC/GSK3B | 3 |
| BP | GO:0007589 | body fluid secretion | 3/32 | 89/18614 | 0.000474 | 0.001785 | 0.00054 | HIF1A/CCND1/CAV1 | 3 |
| BP | GO:0032945 | negative regulation of mononuclear cell proliferation | 3/32 | 89/18614 | 0.000474 | 0.001785 | 0.00054 | CASP3/ERBB2/IL10 | 3 |
| BP | GO:2000177 | regulation of neural precursor cell proliferation | 3/32 | 89/18614 | 0.000474 | 0.001785 | 0.00054 | TP53/HIF1A/EGF | 3 |
| BP | GO:0045637 | regulation of myeloid cell differentiation | 4/32 | 214/18614 | 0.000475 | 0.001785 | 0.00054 | HIF1A/FOS/MYC/IFNG | 4 |
| BP | GO:0035728 | response to hepatocyte growth factor | 2/32 | 19/18614 | 0.000481 | 0.001798 | 0.000544 | IL10/RELA | 2 |
| BP | GO:0045346 | regulation of MHC class II biosynthetic process | 2/32 | 19/18614 | 0.000481 | 0.001798 | 0.000544 | IL10/IFNG | 2 |
| BP | GO:0060716 | labyrinthine layer blood vessel development | 2/32 | 19/18614 | 0.000481 | 0.001798 | 0.000544 | AKT1/MAPK1 | 2 |
| BP | GO:0070242 | thymocyte apoptotic process | 2/32 | 19/18614 | 0.000481 | 0.001798 | 0.000544 | TP53/HIF1A | 2 |
| BP | GO:0014910 | regulation of smooth muscle cell migration | 3/32 | 90/18614 | 0.00049 | 0.00183 | 0.000553 | BCL2/NFE2L2/SERPINE1 | 3 |
| BP | GO:0042180 | cellular ketone metabolic process | 4/32 | 216/18614 | 0.000491 | 0.001833 | 0.000554 | AKT1/IL1B/PTGS2/CAV1 | 4 |
| BP | GO:0001942 | hair follicle development | 3/32 | 91/18614 | 0.000506 | 0.00188 | 0.000568 | EGFR/BCL2/RELA | 3 |
| BP | GO:0070098 | chemokine-mediated signaling pathway | 3/32 | 91/18614 | 0.000506 | 0.00188 | 0.000568 | HIF1A/CCL2/CXCL8 | 3 |
| BP | GO:2000117 | negative regulation of cysteine-type endopeptidase activity | 3/32 | 91/18614 | 0.000506 | 0.00188 | 0.000568 | AKT1/PTGS2/MMP9 | 3 |
| BP | GO:0007179 | transforming growth factor beta receptor signaling pathway | 4/32 | 218/18614 | 0.000509 | 0.001888 | 0.000571 | TP53/FOS/PPARG/CAV1 | 4 |
| BP | GO:0045185 | maintenance of protein location | 3/32 | 92/18614 | 0.000522 | 0.001935 | 0.000585 | AKT1/IL10/CAV1 | 3 |
| BP | GO:0045342 | MHC class II biosynthetic process | 2/32 | 20/18614 | 0.000534 | 0.001969 | 0.000595 | IL10/IFNG | 2 |
| BP | GO:0055093 | response to hyperoxia | 2/32 | 20/18614 | 0.000534 | 0.001969 | 0.000595 | MMP2/CAV1 | 2 |
| BP | GO:1904292 | regulation of ERAD pathway | 2/32 | 20/18614 | 0.000534 | 0.001969 | 0.000595 | NFE2L2/CAV1 | 2 |
| BP | GO:0030900 | forebrain development | 5/32 | 397/18614 | 0.000539 | 0.001984 | 0.0006 | CASP3/EGFR/HIF1A/GSK3B/APP | 5 |
| BP | GO:0070301 | cellular response to hydrogen peroxide | 3/32 | 93/18614 | 0.000539 | 0.001984 | 0.0006 | IL10/RELA/NFE2L2 | 3 |
| BP | GO:0022404 | molting cycle process | 3/32 | 94/18614 | 0.000556 | 0.002039 | 0.000616 | EGFR/BCL2/RELA | 3 |
| BP | GO:0022405 | hair cycle process | 3/32 | 94/18614 | 0.000556 | 0.002039 | 0.000616 | EGFR/BCL2/RELA | 3 |
| BP | GO:0046031 | ADP metabolic process | 3/32 | 94/18614 | 0.000556 | 0.002039 | 0.000616 | HIF1A/IFNG/APP | 3 |
| BP | GO:0050866 | negative regulation of cell activation | 4/32 | 225/18614 | 0.000573 | 0.002094 | 0.000633 | CASP3/ERBB2/IL10/HMOX1 | 4 |
| BP | GO:0042982 | amyloid precursor protein metabolic process | 3/32 | 95/18614 | 0.000574 | 0.002094 | 0.000633 | CASP3/IFNG/RELA | 3 |
| BP | GO:0046330 | positive regulation of JNK cascade | 3/32 | 95/18614 | 0.000574 | 0.002094 | 0.000633 | IL1B/IL1A/APP | 3 |
| BP | GO:0002902 | regulation of B cell apoptotic process | 2/32 | 21/18614 | 0.000589 | 0.002125 | 0.000642 | BCL2/IL10 | 2 |
| BP | GO:0010224 | response to UV-B | 2/32 | 21/18614 | 0.000589 | 0.002125 | 0.000642 | BCL2/RELA | 2 |
| BP | GO:0010288 | response to lead ion | 2/32 | 21/18614 | 0.000589 | 0.002125 | 0.000642 | PTGS2/APP | 2 |
| BP | GO:0030728 | ovulation | 2/32 | 21/18614 | 0.000589 | 0.002125 | 0.000642 | PTGS2/MMP2 | 2 |
| BP | GO:0032305 | positive regulation of icosanoid secretion | 2/32 | 21/18614 | 0.000589 | 0.002125 | 0.000642 | IL1B/IL1A | 2 |
| BP | GO:0038083 | peptidyl-tyrosine autophosphorylation | 2/32 | 21/18614 | 0.000589 | 0.002125 | 0.000642 | EGFR/CAV1 | 2 |
| BP | GO:1903204 | negative regulation of oxidative stress-induced neuron death | 2/32 | 21/18614 | 0.000589 | 0.002125 | 0.000642 | HIF1A/IL10 | 2 |
| BP | GO:1903206 | negative regulation of hydrogen peroxide-induced cell death | 2/32 | 21/18614 | 0.000589 | 0.002125 | 0.000642 | IL10/NFE2L2 | 2 |
| BP | GO:0002042 | cell migration involved in sprouting angiogenesis | 3/32 | 96/18614 | 0.000592 | 0.002125 | 0.000642 | AKT1/PTGS2/HMOX1 | 3 |
| BP | GO:0002690 | positive regulation of leukocyte chemotaxis | 3/32 | 96/18614 | 0.000592 | 0.002125 | 0.000642 | CXCL8/MAPK1/SERPINE1 | 3 |
| BP | GO:0048534 | hematopoietic or lymphoid organ development | 3/32 | 96/18614 | 0.000592 | 0.002125 | 0.000642 | TP53/BCL2/MAPK1 | 3 |
| BP | GO:0070664 | negative regulation of leukocyte proliferation | 3/32 | 96/18614 | 0.000592 | 0.002125 | 0.000642 | CASP3/ERBB2/IL10 | 3 |
| BP | GO:0014909 | smooth muscle cell migration | 3/32 | 97/18614 | 0.00061 | 0.002183 | 0.00066 | BCL2/NFE2L2/SERPINE1 | 3 |
| BP | GO:1903321 | negative regulation of protein modification by small protein conjugation or removal | 3/32 | 97/18614 | 0.00061 | 0.002183 | 0.00066 | AKT1/RELA/CAV1 | 3 |
| BP | GO:0009615 | response to virus | 5/32 | 408/18614 | 0.00061 | 0.002183 | 0.00066 | IL1B/BCL2/HIF1A/IFNG/RELA | 5 |
| BP | GO:0050920 | regulation of chemotaxis | 4/32 | 230/18614 | 0.000622 | 0.002224 | 0.000672 | CCL2/CXCL8/MAPK1/SERPINE1 | 4 |
| BP | GO:0043270 | positive regulation of monoatomic ion transport | 4/32 | 231/18614 | 0.000633 | 0.002257 | 0.000682 | AKT1/CCL2/IFNG/CAV1 | 4 |
| BP | GO:1990868 | response to chemokine | 3/32 | 99/18614 | 0.000647 | 0.002283 | 0.00069 | HIF1A/CCL2/CXCL8 | 3 |
| BP | GO:1990869 | cellular response to chemokine | 3/32 | 99/18614 | 0.000647 | 0.002283 | 0.00069 | HIF1A/CCL2/CXCL8 | 3 |
| BP | GO:0010042 | response to manganese ion | 2/32 | 22/18614 | 0.000647 | 0.002283 | 0.00069 | PTGS2/APP | 2 |
| BP | GO:0030220 | platelet formation | 2/32 | 22/18614 | 0.000647 | 0.002283 | 0.00069 | CASP3/CASP9 | 2 |
| BP | GO:0032303 | regulation of icosanoid secretion | 2/32 | 22/18614 | 0.000647 | 0.002283 | 0.00069 | IL1B/IL1A | 2 |
| BP | GO:0045723 | positive regulation of fatty acid biosynthetic process | 2/32 | 22/18614 | 0.000647 | 0.002283 | 0.00069 | IL1B/PTGS2 | 2 |
| BP | GO:0051000 | positive regulation of nitric-oxide synthase activity | 2/32 | 22/18614 | 0.000647 | 0.002283 | 0.00069 | AKT1/HIF1A | 2 |
| BP | GO:0051767 | nitric-oxide synthase biosynthetic process | 2/32 | 22/18614 | 0.000647 | 0.002283 | 0.00069 | CCL2/IFNG | 2 |
| BP | GO:0051769 | regulation of nitric-oxide synthase biosynthetic process | 2/32 | 22/18614 | 0.000647 | 0.002283 | 0.00069 | CCL2/IFNG | 2 |
| BP | GO:0030512 | negative regulation of transforming growth factor beta receptor signaling pathway | 3/32 | 100/18614 | 0.000666 | 0.002344 | 0.000709 | TP53/PPARG/CAV1 | 3 |
| BP | GO:0140888 | interferon-mediated signaling pathway | 3/32 | 100/18614 | 0.000666 | 0.002344 | 0.000709 | TP53/PPARG/IFNG | 3 |
| BP | GO:0043255 | regulation of carbohydrate biosynthetic process | 3/32 | 101/18614 | 0.000686 | 0.002407 | 0.000728 | AKT1/GSK3B/EGF | 3 |
| BP | GO:0050764 | regulation of phagocytosis | 3/32 | 101/18614 | 0.000686 | 0.002407 | 0.000728 | IL1B/CCL2/IFNG | 3 |
| BP | GO:0072593 | reactive oxygen species metabolic process | 4/32 | 237/18614 | 0.000696 | 0.00244 | 0.000738 | TP53/BCL2/HIF1A/NFE2L2 | 4 |
| BP | GO:0006165 | nucleoside diphosphate phosphorylation | 3/32 | 102/18614 | 0.000706 | 0.002462 | 0.000745 | HIF1A/IFNG/APP | 3 |
| BP | GO:1904063 | negative regulation of cation transmembrane transport | 3/32 | 102/18614 | 0.000706 | 0.002462 | 0.000745 | MMP9/BCL2/CAV1 | 3 |
| BP | GO:0010893 | positive regulation of steroid biosynthetic process | 2/32 | 23/18614 | 0.000708 | 0.002462 | 0.000745 | IFNG/IL1A | 2 |
| BP | GO:0046716 | muscle cell cellular homeostasis | 2/32 | 23/18614 | 0.000708 | 0.002462 | 0.000745 | HIF1A/CAV1 | 2 |
| BP | GO:1900120 | regulation of receptor binding | 2/32 | 23/18614 | 0.000708 | 0.002462 | 0.000745 | MMP9/IL10 | 2 |
| BP | GO:1901032 | negative regulation of response to reactive oxygen species | 2/32 | 23/18614 | 0.000708 | 0.002462 | 0.000745 | IL10/NFE2L2 | 2 |
| BP | GO:0002757 | immune response-activating signaling pathway | 5/32 | 423/18614 | 0.000718 | 0.002495 | 0.000754 | ESR1/BCL2/RELA/MAPK1/CAV1 | 5 |
| BP | GO:0042100 | B cell proliferation | 3/32 | 103/18614 | 0.000726 | 0.002512 | 0.00076 | CASP3/BCL2/IL10 | 3 |
| BP | GO:0045639 | positive regulation of myeloid cell differentiation | 3/32 | 103/18614 | 0.000726 | 0.002512 | 0.00076 | HIF1A/FOS/IFNG | 3 |
| BP | GO:1903076 | regulation of protein localization to plasma membrane | 3/32 | 103/18614 | 0.000726 | 0.002512 | 0.00076 | AKT1/EGFR/IFNG | 3 |
| BP | GO:0046939 | nucleotide phosphorylation | 3/32 | 104/18614 | 0.000747 | 0.002571 | 0.000777 | HIF1A/IFNG/APP | 3 |
| BP | GO:0060291 | long-term synaptic potentiation | 3/32 | 104/18614 | 0.000747 | 0.002571 | 0.000777 | GSK3B/APP/MAPK1 | 3 |
| BP | GO:0071868 | cellular response to monoamine stimulus | 3/32 | 104/18614 | 0.000747 | 0.002571 | 0.000777 | GSK3B/APP/MAPK1 | 3 |
| BP | GO:0071870 | cellular response to catecholamine stimulus | 3/32 | 104/18614 | 0.000747 | 0.002571 | 0.000777 | GSK3B/APP/MAPK1 | 3 |
| BP | GO:0009636 | response to toxic substance | 4/32 | 242/18614 | 0.000753 | 0.002587 | 0.000782 | PTGS2/BCL2/FOS/NFE2L2 | 4 |
| BP | GO:0032760 | positive regulation of tumor necrosis factor production | 3/32 | 105/18614 | 0.000768 | 0.002633 | 0.000796 | IFNG/IL1A/APP | 3 |
| BP | GO:0003323 | type B pancreatic cell development | 2/32 | 24/18614 | 0.000772 | 0.002633 | 0.000796 | AKT1/GSK3B | 2 |
| BP | GO:0036344 | platelet morphogenesis | 2/32 | 24/18614 | 0.000772 | 0.002633 | 0.000796 | CASP3/CASP9 | 2 |
| BP | GO:0060571 | morphogenesis of an epithelial fold | 2/32 | 24/18614 | 0.000772 | 0.002633 | 0.000796 | EGFR/HIF1A | 2 |
| BP | GO:0070233 | negative regulation of T cell apoptotic process | 2/32 | 24/18614 | 0.000772 | 0.002633 | 0.000796 | BCL2/HIF1A | 2 |
| BP | GO:0071676 | negative regulation of mononuclear cell migration | 2/32 | 24/18614 | 0.000772 | 0.002633 | 0.000796 | AKT1/CCL2 | 2 |
| BP | GO:0009135 | purine nucleoside diphosphate metabolic process | 3/32 | 106/18614 | 0.000789 | 0.002686 | 0.000812 | HIF1A/IFNG/APP | 3 |
| BP | GO:0009179 | purine ribonucleoside diphosphate metabolic process | 3/32 | 106/18614 | 0.000789 | 0.002686 | 0.000812 | HIF1A/IFNG/APP | 3 |
| BP | GO:0015711 | organic anion transport | 5/32 | 433/18614 | 0.000798 | 0.002709 | 0.000819 | AKT1/IL1B/PTGS2/PPARG/IL1A | 5 |
| BP | GO:0042391 | regulation of membrane potential | 5/32 | 433/18614 | 0.000798 | 0.002709 | 0.000819 | AKT1/BCL2/GSK3B/APP/CAV1 | 5 |
| BP | GO:0002703 | regulation of leukocyte mediated immunity | 4/32 | 246/18614 | 0.0008 | 0.002709 | 0.000819 | IL1B/IL10/ICAM1/HMOX1 | 4 |
| BP | GO:0045444 | fat cell differentiation | 4/32 | 246/18614 | 0.0008 | 0.002709 | 0.000819 | AKT1/PTGS2/PPARG/CCND1 | 4 |
| BP | GO:0005976 | polysaccharide metabolic process | 3/32 | 107/18614 | 0.000811 | 0.00273 | 0.000825 | AKT1/GSK3B/EGF | 3 |
| BP | GO:0030593 | neutrophil chemotaxis | 3/32 | 107/18614 | 0.000811 | 0.00273 | 0.000825 | IL1B/CCL2/CXCL8 | 3 |
| BP | GO:0071867 | response to monoamine | 3/32 | 107/18614 | 0.000811 | 0.00273 | 0.000825 | GSK3B/APP/MAPK1 | 3 |
| BP | GO:0071869 | response to catecholamine | 3/32 | 107/18614 | 0.000811 | 0.00273 | 0.000825 | GSK3B/APP/MAPK1 | 3 |
| BP | GO:1901655 | cellular response to ketone | 3/32 | 107/18614 | 0.000811 | 0.00273 | 0.000825 | AKT1/FOS/CASP9 | 3 |
| BP | GO:0046883 | regulation of hormone secretion | 4/32 | 247/18614 | 0.000812 | 0.00273 | 0.000826 | IL1B/HIF1A/PPARG/IFNG | 4 |
| BP | GO:0032963 | collagen metabolic process | 3/32 | 108/18614 | 0.000834 | 0.002795 | 0.000845 | MMP9/HIF1A/MMP2 | 3 |
| BP | GO:0010226 | response to lithium ion | 2/32 | 25/18614 | 0.000838 | 0.002795 | 0.000845 | PTGS2/NFE2L2 | 2 |
| BP | GO:0034114 | regulation of heterotypic cell-cell adhesion | 2/32 | 25/18614 | 0.000838 | 0.002795 | 0.000845 | IL1B/IL10 | 2 |
| BP | GO:1905563 | negative regulation of vascular endothelial cell proliferation | 2/32 | 25/18614 | 0.000838 | 0.002795 | 0.000845 | PPARG/CCL2 | 2 |
| BP | GO:2000193 | positive regulation of fatty acid transport | 2/32 | 25/18614 | 0.000838 | 0.002795 | 0.000845 | IL1B/IL1A | 2 |
| BP | GO:2000209 | regulation of anoikis | 2/32 | 25/18614 | 0.000838 | 0.002795 | 0.000845 | BCL2/CAV1 | 2 |
| BP | GO:0034766 | negative regulation of monoatomic ion transmembrane transport | 3/32 | 109/18614 | 0.000856 | 0.002845 | 0.00086 | MMP9/BCL2/CAV1 | 3 |
| BP | GO:0042116 | macrophage activation | 3/32 | 109/18614 | 0.000856 | 0.002845 | 0.00086 | IL10/IFNG/APP | 3 |
| BP | GO:1903557 | positive regulation of tumor necrosis factor superfamily cytokine production | 3/32 | 109/18614 | 0.000856 | 0.002845 | 0.00086 | IFNG/IL1A/APP | 3 |
| BP | GO:0014812 | muscle cell migration | 3/32 | 110/18614 | 0.000879 | 0.002915 | 0.000881 | BCL2/NFE2L2/SERPINE1 | 3 |
| BP | GO:0090263 | positive regulation of canonical Wnt signaling pathway | 3/32 | 110/18614 | 0.000879 | 0.002915 | 0.000881 | EGFR/EGF/CAV1 | 3 |
| BP | GO:0032469 | endoplasmic reticulum calcium ion homeostasis | 2/32 | 26/18614 | 0.000907 | 0.002988 | 0.000904 | BCL2/APP | 2 |
| BP | GO:0035994 | response to muscle stretch | 2/32 | 26/18614 | 0.000907 | 0.002988 | 0.000904 | FOS/RELA | 2 |
| BP | GO:0045672 | positive regulation of osteoclast differentiation | 2/32 | 26/18614 | 0.000907 | 0.002988 | 0.000904 | FOS/IFNG | 2 |
| BP | GO:0050995 | negative regulation of lipid catabolic process | 2/32 | 26/18614 | 0.000907 | 0.002988 | 0.000904 | AKT1/IL1B | 2 |
| BP | GO:0060740 | prostate gland epithelium morphogenesis | 2/32 | 26/18614 | 0.000907 | 0.002988 | 0.000904 | ESR1/MMP2 | 2 |
| BP | GO:1903320 | regulation of protein modification by small protein conjugation or removal | 4/32 | 255/18614 | 0.000915 | 0.003011 | 0.00091 | AKT1/HIF1A/RELA/CAV1 | 4 |
| BP | GO:0006090 | pyruvate metabolic process | 3/32 | 112/18614 | 0.000926 | 0.003037 | 0.000918 | HIF1A/IFNG/APP | 3 |
| BP | GO:0042177 | negative regulation of protein catabolic process | 3/32 | 112/18614 | 0.000926 | 0.003037 | 0.000918 | EGFR/IL10/RELA | 3 |
| BP | GO:1902749 | regulation of cell cycle G2/M phase transition | 3/32 | 112/18614 | 0.000926 | 0.003037 | 0.000918 | TP53/CCND1/APP | 3 |
| BP | GO:0006898 | receptor-mediated endocytosis | 4/32 | 256/18614 | 0.000928 | 0.00304 | 0.000919 | EGF/CXCL8/SERPINE1/CAV1 | 4 |
| BP | GO:0002764 | immune response-regulating signaling pathway | 5/32 | 450/18614 | 0.000949 | 0.003103 | 0.000938 | ESR1/BCL2/RELA/MAPK1/CAV1 | 5 |
| BP | GO:0045927 | positive regulation of growth | 4/32 | 258/18614 | 0.000955 | 0.003122 | 0.000944 | AKT1/EGFR/BCL2/ERBB2 | 4 |
| BP | GO:0003012 | muscle system process | 5/32 | 452/18614 | 0.000968 | 0.003155 | 0.000954 | IL1B/PTGS2/PPARG/HMOX1/CAV1 | 5 |
| BP | GO:0002526 | acute inflammatory response | 3/32 | 114/18614 | 0.000975 | 0.003155 | 0.000954 | IL1B/PTGS2/IL1A | 3 |
| BP | GO:1904659 | glucose transmembrane transport | 3/32 | 114/18614 | 0.000975 | 0.003155 | 0.000954 | AKT1/IL1B/NFE2L2 | 3 |
| BP | GO:0001783 | B cell apoptotic process | 2/32 | 27/18614 | 0.000978 | 0.003155 | 0.000954 | BCL2/IL10 | 2 |
| BP | GO:0010875 | positive regulation of cholesterol efflux | 2/32 | 27/18614 | 0.000978 | 0.003155 | 0.000954 | PPARG/CAV1 | 2 |
| BP | GO:0036296 | response to increased oxygen levels | 2/32 | 27/18614 | 0.000978 | 0.003155 | 0.000954 | MMP2/CAV1 | 2 |
| BP | GO:0045940 | positive regulation of steroid metabolic process | 2/32 | 27/18614 | 0.000978 | 0.003155 | 0.000954 | IFNG/IL1A | 2 |
| BP | GO:0071280 | cellular response to copper ion | 2/32 | 27/18614 | 0.000978 | 0.003155 | 0.000954 | NFE2L2/APP | 2 |
| BP | GO:0090200 | positive regulation of release of cytochrome c from mitochondria | 2/32 | 27/18614 | 0.000978 | 0.003155 | 0.000954 | TP53/MMP9 | 2 |
| BP | GO:2000108 | positive regulation of leukocyte apoptotic process | 2/32 | 27/18614 | 0.000978 | 0.003155 | 0.000954 | TP53/IL10 | 2 |
| BP | GO:2000737 | negative regulation of stem cell differentiation | 2/32 | 27/18614 | 0.000978 | 0.003155 | 0.000954 | GSK3B/NFE2L2 | 2 |
| BP | GO:0060828 | regulation of canonical Wnt signaling pathway | 4/32 | 260/18614 | 0.000983 | 0.003167 | 0.000958 | EGFR/GSK3B/EGF/CAV1 | 4 |
| BP | GO:0006816 | calcium ion transport | 5/32 | 455/18614 | 0.000997 | 0.003207 | 0.00097 | PTGS2/BCL2/EGF/CCL2/CAV1 | 5 |
| BP | GO:0009185 | ribonucleoside diphosphate metabolic process | 3/32 | 116/18614 | 0.001025 | 0.00329 | 0.000995 | HIF1A/IFNG/APP | 3 |
| BP | GO:0042752 | regulation of circadian rhythm | 3/32 | 116/18614 | 0.001025 | 0.00329 | 0.000995 | TP53/PPARG/GSK3B | 3 |
| BP | GO:0007088 | regulation of mitotic nuclear division | 3/32 | 117/18614 | 0.001051 | 0.003342 | 0.001011 | IL1B/EGF/IL1A | 3 |
| BP | GO:0008645 | hexose transmembrane transport | 3/32 | 117/18614 | 0.001051 | 0.003342 | 0.001011 | AKT1/IL1B/NFE2L2 | 3 |
| BP | GO:0002675 | positive regulation of acute inflammatory response | 2/32 | 28/18614 | 0.001052 | 0.003342 | 0.001011 | IL1B/PTGS2 | 2 |
| BP | GO:0010971 | positive regulation of G2/M transition of mitotic cell cycle | 2/32 | 28/18614 | 0.001052 | 0.003342 | 0.001011 | CCND1/APP | 2 |
| BP | GO:0030194 | positive regulation of blood coagulation | 2/32 | 28/18614 | 0.001052 | 0.003342 | 0.001011 | NFE2L2/SERPINE1 | 2 |
| BP | GO:0060402 | calcium ion transport into cytosol | 2/32 | 28/18614 | 0.001052 | 0.003342 | 0.001011 | BCL2/CAV1 | 2 |
| BP | GO:0060512 | prostate gland morphogenesis | 2/32 | 28/18614 | 0.001052 | 0.003342 | 0.001011 | ESR1/MMP2 | 2 |
| BP | GO:1900048 | positive regulation of hemostasis | 2/32 | 28/18614 | 0.001052 | 0.003342 | 0.001011 | NFE2L2/SERPINE1 | 2 |
| BP | GO:1902883 | negative regulation of response to oxidative stress | 2/32 | 28/18614 | 0.001052 | 0.003342 | 0.001011 | IL10/NFE2L2 | 2 |
| BP | GO:0051960 | regulation of nervous system development | 5/32 | 461/18614 | 0.001057 | 0.003352 | 0.001014 | AKT1/IL1B/TP53/HIF1A/IFNG | 5 |
| BP | GO:0002699 | positive regulation of immune effector process | 4/32 | 267/18614 | 0.001085 | 0.003437 | 0.001039 | IL1B/IL10/IFNG/HMOX1 | 4 |
| BP | GO:0002286 | T cell activation involved in immune response | 3/32 | 119/18614 | 0.001104 | 0.003485 | 0.001054 | TP53/IFNG/ICAM1 | 3 |
| BP | GO:0002718 | regulation of cytokine production involved in immune response | 3/32 | 119/18614 | 0.001104 | 0.003485 | 0.001054 | IL1B/IL10/HMOX1 | 3 |
| BP | GO:0030278 | regulation of ossification | 3/32 | 119/18614 | 0.001104 | 0.003485 | 0.001054 | BCL2/HIF1A/MAPK1 | 3 |
| BP | GO:0031571 | mitotic G1 DNA damage checkpoint signaling | 2/32 | 29/18614 | 0.001129 | 0.003545 | 0.001072 | TP53/CCND1 | 2 |
| BP | GO:0044819 | mitotic G1/S transition checkpoint signaling | 2/32 | 29/18614 | 0.001129 | 0.003545 | 0.001072 | TP53/CCND1 | 2 |
| BP | GO:1903205 | regulation of hydrogen peroxide-induced cell death | 2/32 | 29/18614 | 0.001129 | 0.003545 | 0.001072 | IL10/NFE2L2 | 2 |
| BP | GO:0007006 | mitochondrial membrane organization | 3/32 | 120/18614 | 0.001131 | 0.003545 | 0.001072 | TP53/BCL2/GSK3B | 3 |
| BP | GO:0015749 | monosaccharide transmembrane transport | 3/32 | 120/18614 | 0.001131 | 0.003545 | 0.001072 | AKT1/IL1B/NFE2L2 | 3 |
| BP | GO:0021537 | telencephalon development | 4/32 | 270/18614 | 0.001131 | 0.003545 | 0.001072 | CASP3/EGFR/HIF1A/GSK3B | 4 |
| BP | GO:0002367 | cytokine production involved in immune response | 3/32 | 121/18614 | 0.001158 | 0.003614 | 0.001093 | IL1B/IL10/HMOX1 | 3 |
| BP | GO:0043500 | muscle adaptation | 3/32 | 121/18614 | 0.001158 | 0.003614 | 0.001093 | IL1B/PPARG/HMOX1 | 3 |
| BP | GO:0098773 | skin epidermis development | 3/32 | 121/18614 | 0.001158 | 0.003614 | 0.001093 | EGFR/BCL2/RELA | 3 |
| BP | GO:2000278 | regulation of DNA biosynthetic process | 3/32 | 121/18614 | 0.001158 | 0.003614 | 0.001093 | TP53/MYC/MAPK1 | 3 |
| BP | GO:0072676 | lymphocyte migration | 3/32 | 122/18614 | 0.001186 | 0.003697 | 0.001118 | AKT1/CCL2/APP | 3 |
| BP | GO:0071560 | cellular response to transforming growth factor beta stimulus | 4/32 | 274/18614 | 0.001194 | 0.003713 | 0.001123 | TP53/FOS/PPARG/CAV1 | 4 |
| BP | GO:1901988 | negative regulation of cell cycle phase transition | 4/32 | 274/18614 | 0.001194 | 0.003713 | 0.001123 | TP53/BCL2/CCND1/CCL2 | 4 |
| BP | GO:0002360 | T cell lineage commitment | 2/32 | 30/18614 | 0.001209 | 0.003725 | 0.001126 | TP53/BCL2 | 2 |
| BP | GO:0005979 | regulation of glycogen biosynthetic process | 2/32 | 30/18614 | 0.001209 | 0.003725 | 0.001126 | AKT1/GSK3B | 2 |
| BP | GO:0007176 | regulation of epidermal growth factor-activated receptor activity | 2/32 | 30/18614 | 0.001209 | 0.003725 | 0.001126 | EGF/APP | 2 |
| BP | GO:0010962 | regulation of glucan biosynthetic process | 2/32 | 30/18614 | 0.001209 | 0.003725 | 0.001126 | AKT1/GSK3B | 2 |
| BP | GO:0045907 | positive regulation of vasoconstriction | 2/32 | 30/18614 | 0.001209 | 0.003725 | 0.001126 | PTGS2/CAV1 | 2 |
| BP | GO:0050820 | positive regulation of coagulation | 2/32 | 30/18614 | 0.001209 | 0.003725 | 0.001126 | NFE2L2/SERPINE1 | 2 |
| BP | GO:0070498 | interleukin-1-mediated signaling pathway | 2/32 | 30/18614 | 0.001209 | 0.003725 | 0.001126 | IL1B/RELA | 2 |
| BP | GO:0090312 | positive regulation of protein deacetylation | 2/32 | 30/18614 | 0.001209 | 0.003725 | 0.001126 | TP53/IFNG | 2 |
| BP | GO:0002761 | regulation of myeloid leukocyte differentiation | 3/32 | 123/18614 | 0.001214 | 0.003738 | 0.00113 | FOS/MYC/IFNG | 3 |
| BP | GO:0042113 | B cell activation | 4/32 | 276/18614 | 0.001226 | 0.003771 | 0.00114 | TP53/CASP3/BCL2/IL10 | 4 |
| BP | GO:0034765 | regulation of monoatomic ion transmembrane transport | 5/32 | 478/18614 | 0.001242 | 0.003804 | 0.00115 | MMP9/BCL2/CCL2/IFNG/CAV1 | 5 |
| BP | GO:0030301 | cholesterol transport | 3/32 | 124/18614 | 0.001243 | 0.003804 | 0.00115 | PPARG/EGF/CAV1 | 3 |
| BP | GO:0043280 | positive regulation of cysteine-type endopeptidase activity involved in apoptotic process | 3/32 | 124/18614 | 0.001243 | 0.003804 | 0.00115 | MYC/PPARG/CASP9 | 3 |
| BP | GO:0060079 | excitatory postsynaptic potential | 3/32 | 124/18614 | 0.001243 | 0.003804 | 0.00115 | AKT1/GSK3B/APP | 3 |
| BP | GO:0001952 | regulation of cell-matrix adhesion | 3/32 | 125/18614 | 0.001272 | 0.003884 | 0.001174 | BCL2/GSK3B/SERPINE1 | 3 |
| BP | GO:0031623 | receptor internalization | 3/32 | 125/18614 | 0.001272 | 0.003884 | 0.001174 | EGF/CXCL8/CAV1 | 3 |
| BP | GO:0003309 | type B pancreatic cell differentiation | 2/32 | 31/18614 | 0.001291 | 0.003924 | 0.001186 | AKT1/GSK3B | 2 |
| BP | GO:0060674 | placenta blood vessel development | 2/32 | 31/18614 | 0.001291 | 0.003924 | 0.001186 | AKT1/MAPK1 | 2 |
| BP | GO:1902751 | positive regulation of cell cycle G2/M phase transition | 2/32 | 31/18614 | 0.001291 | 0.003924 | 0.001186 | CCND1/APP | 2 |
| BP | GO:1903203 | regulation of oxidative stress-induced neuron death | 2/32 | 31/18614 | 0.001291 | 0.003924 | 0.001186 | HIF1A/IL10 | 2 |
| BP | GO:0071559 | response to transforming growth factor beta | 4/32 | 280/18614 | 0.001293 | 0.003927 | 0.001187 | TP53/FOS/PPARG/CAV1 | 4 |
| BP | GO:0021987 | cerebral cortex development | 3/32 | 126/18614 | 0.001301 | 0.003948 | 0.001194 | EGFR/HIF1A/GSK3B | 3 |
| BP | GO:0045165 | cell fate commitment | 4/32 | 281/18614 | 0.00131 | 0.00397 | 0.001201 | TP53/CASP3/BCL2/PPARG | 4 |
| BP | GO:1904375 | regulation of protein localization to cell periphery | 3/32 | 128/18614 | 0.001362 | 0.004122 | 0.001246 | AKT1/EGFR/IFNG | 3 |
| BP | GO:0001516 | prostaglandin biosynthetic process | 2/32 | 32/18614 | 0.001375 | 0.004135 | 0.00125 | IL1B/PTGS2 | 2 |
| BP | GO:0033198 | response to ATP | 2/32 | 32/18614 | 0.001375 | 0.004135 | 0.00125 | IL1B/PTGS2 | 2 |
| BP | GO:0034390 | smooth muscle cell apoptotic process | 2/32 | 32/18614 | 0.001375 | 0.004135 | 0.00125 | PPARG/IFNG | 2 |
| BP | GO:0034391 | regulation of smooth muscle cell apoptotic process | 2/32 | 32/18614 | 0.001375 | 0.004135 | 0.00125 | PPARG/IFNG | 2 |
| BP | GO:0046457 | prostanoid biosynthetic process | 2/32 | 32/18614 | 0.001375 | 0.004135 | 0.00125 | IL1B/PTGS2 | 2 |
| BP | GO:0046825 | regulation of protein export from nucleus | 2/32 | 32/18614 | 0.001375 | 0.004135 | 0.00125 | IL1B/GSK3B | 2 |
| BP | GO:0019079 | viral genome replication | 3/32 | 129/18614 | 0.001392 | 0.004177 | 0.001263 | BCL2/CCL2/CXCL8 | 3 |
| BP | GO:0032874 | positive regulation of stress-activated MAPK cascade | 3/32 | 129/18614 | 0.001392 | 0.004177 | 0.001263 | IL1B/IL1A/APP | 3 |
| BP | GO:0050864 | regulation of B cell activation | 3/32 | 130/18614 | 0.001424 | 0.004266 | 0.00129 | CASP3/BCL2/IL10 | 3 |
| BP | GO:0032612 | interleukin-1 production | 3/32 | 131/18614 | 0.001455 | 0.004339 | 0.001312 | IL10/IFNG/APP | 3 |
| BP | GO:0032652 | regulation of interleukin-1 production | 3/32 | 131/18614 | 0.001455 | 0.004339 | 0.001312 | IL10/IFNG/APP | 3 |
| BP | GO:0050868 | negative regulation of T cell activation | 3/32 | 131/18614 | 0.001455 | 0.004339 | 0.001312 | CASP3/ERBB2/IL10 | 3 |
| BP | GO:0070304 | positive regulation of stress-activated protein kinase signaling cascade | 3/32 | 131/18614 | 0.001455 | 0.004339 | 0.001312 | IL1B/IL1A/APP | 3 |
| BP | GO:0030947 | regulation of vascular endothelial growth factor receptor signaling pathway | 2/32 | 33/18614 | 0.001462 | 0.004339 | 0.001312 | IL1B/HIF1A | 2 |
| BP | GO:0036474 | cell death in response to hydrogen peroxide | 2/32 | 33/18614 | 0.001462 | 0.004339 | 0.001312 | IL10/NFE2L2 | 2 |
| BP | GO:0071404 | cellular response to low-density lipoprotein particle stimulus | 2/32 | 33/18614 | 0.001462 | 0.004339 | 0.001312 | AKT1/PPARG | 2 |
| BP | GO:0090279 | regulation of calcium ion import | 2/32 | 33/18614 | 0.001462 | 0.004339 | 0.001312 | EGF/CCL2 | 2 |
| BP | GO:1904893 | negative regulation of receptor signaling pathway via STAT | 2/32 | 33/18614 | 0.001462 | 0.004339 | 0.001312 | PPARG/CAV1 | 2 |
| BP | GO:0002244 | hematopoietic progenitor cell differentiation | 3/32 | 132/18614 | 0.001488 | 0.004404 | 0.001332 | TP53/BCL2/NFE2L2 | 3 |
| BP | GO:0099565 | chemical synaptic transmission, postsynaptic | 3/32 | 132/18614 | 0.001488 | 0.004404 | 0.001332 | AKT1/GSK3B/APP | 3 |
| BP | GO:0034219 | carbohydrate transmembrane transport | 3/32 | 133/18614 | 0.00152 | 0.004496 | 0.001359 | AKT1/IL1B/NFE2L2 | 3 |
| BP | GO:0032743 | positive regulation of interleukin-2 production | 2/32 | 34/18614 | 0.001552 | 0.004553 | 0.001377 | IL1B/IL1A | 2 |
| BP | GO:0036475 | neuron death in response to oxidative stress | 2/32 | 34/18614 | 0.001552 | 0.004553 | 0.001377 | HIF1A/IL10 | 2 |
| BP | GO:0050869 | negative regulation of B cell activation | 2/32 | 34/18614 | 0.001552 | 0.004553 | 0.001377 | CASP3/IL10 | 2 |
| BP | GO:0051973 | positive regulation of telomerase activity | 2/32 | 34/18614 | 0.001552 | 0.004553 | 0.001377 | MYC/MAPK1 | 2 |
| BP | GO:0055094 | response to lipoprotein particle | 2/32 | 34/18614 | 0.001552 | 0.004553 | 0.001377 | AKT1/PPARG | 2 |
| BP | GO:0060964 | regulation of miRNA-mediated gene silencing | 2/32 | 34/18614 | 0.001552 | 0.004553 | 0.001377 | TP53/PPARG | 2 |
| BP | GO:1902624 | positive regulation of neutrophil migration | 2/32 | 34/18614 | 0.001552 | 0.004553 | 0.001377 | CXCL8/IL1A | 2 |
| BP | GO:0009132 | nucleoside diphosphate metabolic process | 3/32 | 134/18614 | 0.001553 | 0.004553 | 0.001377 | HIF1A/IFNG/APP | 3 |
| BP | GO:0006275 | regulation of DNA replication | 3/32 | 136/18614 | 0.00162 | 0.004746 | 0.001435 | TP53/EGFR/EGF | 3 |
| BP | GO:0001893 | maternal placenta development | 2/32 | 35/18614 | 0.001644 | 0.004779 | 0.001445 | AKT1/PTGS2 | 2 |
| BP | GO:0002719 | negative regulation of cytokine production involved in immune response | 2/32 | 35/18614 | 0.001644 | 0.004779 | 0.001445 | IL10/HMOX1 | 2 |
| BP | GO:0030212 | hyaluronan metabolic process | 2/32 | 35/18614 | 0.001644 | 0.004779 | 0.001445 | IL1B/EGF | 2 |
| BP | GO:0051968 | positive regulation of synaptic transmission, glutamatergic | 2/32 | 35/18614 | 0.001644 | 0.004779 | 0.001445 | PTGS2/CCL2 | 2 |
| BP | GO:0062098 | regulation of programmed necrotic cell death | 2/32 | 35/18614 | 0.001644 | 0.004779 | 0.001445 | TP53/CAV1 | 2 |
| BP | GO:0090050 | positive regulation of cell migration involved in sprouting angiogenesis | 2/32 | 35/18614 | 0.001644 | 0.004779 | 0.001445 | PTGS2/HMOX1 | 2 |
| BP | GO:1905898 | positive regulation of response to endoplasmic reticulum stress | 2/32 | 35/18614 | 0.001644 | 0.004779 | 0.001445 | NFE2L2/CAV1 | 2 |
| BP | GO:0030522 | intracellular receptor signaling pathway | 4/32 | 299/18614 | 0.001646 | 0.004779 | 0.001445 | ESR1/PPARG/RELA/CAV1 | 4 |
| BP | GO:0035270 | endocrine system development | 3/32 | 137/18614 | 0.001655 | 0.004795 | 0.00145 | AKT1/GSK3B/MAPK1 | 3 |
| BP | GO:0061041 | regulation of wound healing | 3/32 | 137/18614 | 0.001655 | 0.004795 | 0.00145 | NFE2L2/SERPINE1/CAV1 | 3 |
| BP | GO:0015918 | sterol transport | 3/32 | 138/18614 | 0.00169 | 0.004885 | 0.001477 | PPARG/EGF/CAV1 | 3 |
| BP | GO:0072175 | epithelial tube formation | 3/32 | 138/18614 | 0.00169 | 0.004885 | 0.001477 | CASP3/HIF1A/EGF | 3 |
| BP | GO:0034101 | erythrocyte homeostasis | 3/32 | 139/18614 | 0.001725 | 0.004982 | 0.001506 | CASP3/HIF1A/HMOX1 | 3 |
| BP | GO:0051607 | defense response to virus | 4/32 | 303/18614 | 0.001728 | 0.004985 | 0.001507 | IL1B/BCL2/IFNG/RELA | 4 |
| BP | GO:0030224 | monocyte differentiation | 2/32 | 36/18614 | 0.001739 | 0.004991 | 0.001509 | MYC/PPARG | 2 |
| BP | GO:0051930 | regulation of sensory perception of pain | 2/32 | 36/18614 | 0.001739 | 0.004991 | 0.001509 | IL10/IL1A | 2 |
| BP | GO:0051931 | regulation of sensory perception | 2/32 | 36/18614 | 0.001739 | 0.004991 | 0.001509 | IL10/IL1A | 2 |
| BP | GO:0070873 | regulation of glycogen metabolic process | 2/32 | 36/18614 | 0.001739 | 0.004991 | 0.001509 | AKT1/GSK3B | 2 |
| BP | GO:1900368 | regulation of post-transcriptional gene silencing by RNA | 2/32 | 36/18614 | 0.001739 | 0.004991 | 0.001509 | TP53/PPARG | 2 |
| BP | GO:0140546 | defense response to symbiont | 4/32 | 304/18614 | 0.001749 | 0.005014 | 0.001516 | IL1B/BCL2/IFNG/RELA | 4 |
| BP | GO:2001235 | positive regulation of apoptotic signaling pathway | 3/32 | 140/18614 | 0.001761 | 0.005042 | 0.001524 | TP53/MYC/CAV1 | 3 |
| BP | GO:0046879 | hormone secretion | 4/32 | 305/18614 | 0.00177 | 0.005063 | 0.001531 | IL1B/HIF1A/PPARG/IFNG | 4 |
| BP | GO:0035296 | regulation of tube diameter | 3/32 | 142/18614 | 0.001833 | 0.00521 | 0.001575 | PTGS2/MMP2/CAV1 | 3 |
| BP | GO:0097746 | blood vessel diameter maintenance | 3/32 | 142/18614 | 0.001833 | 0.00521 | 0.001575 | PTGS2/MMP2/CAV1 | 3 |
| BP | GO:2001056 | positive regulation of cysteine-type endopeptidase activity | 3/32 | 142/18614 | 0.001833 | 0.00521 | 0.001575 | MYC/PPARG/CASP9 | 3 |
| BP | GO:0035883 | enteroendocrine cell differentiation | 2/32 | 37/18614 | 0.001837 | 0.00521 | 0.001575 | AKT1/GSK3B | 2 |
| BP | GO:0046326 | positive regulation of glucose import | 2/32 | 37/18614 | 0.001837 | 0.00521 | 0.001575 | AKT1/NFE2L2 | 2 |
| BP | GO:0060147 | regulation of post-transcriptional gene silencing | 2/32 | 37/18614 | 0.001837 | 0.00521 | 0.001575 | TP53/PPARG | 2 |
| BP | GO:0071402 | cellular response to lipoprotein particle stimulus | 2/32 | 37/18614 | 0.001837 | 0.00521 | 0.001575 | AKT1/PPARG | 2 |
| BP | GO:1903146 | regulation of autophagy of mitochondrion | 2/32 | 37/18614 | 0.001837 | 0.00521 | 0.001575 | TP53/HIF1A | 2 |
| BP | GO:0035150 | regulation of tube size | 3/32 | 143/18614 | 0.001871 | 0.005295 | 0.001601 | PTGS2/MMP2/CAV1 | 3 |
| BP | GO:0050921 | positive regulation of chemotaxis | 3/32 | 143/18614 | 0.001871 | 0.005295 | 0.001601 | CXCL8/MAPK1/SERPINE1 | 3 |
| BP | GO:0001822 | kidney development | 4/32 | 310/18614 | 0.001878 | 0.005311 | 0.001606 | MMP9/BCL2/MYC/CASP9 | 4 |
| BP | GO:0032373 | positive regulation of sterol transport | 2/32 | 38/18614 | 0.001937 | 0.005436 | 0.001643 | PPARG/CAV1 | 2 |
| BP | GO:0032376 | positive regulation of cholesterol transport | 2/32 | 38/18614 | 0.001937 | 0.005436 | 0.001643 | PPARG/CAV1 | 2 |
| BP | GO:0045742 | positive regulation of epidermal growth factor receptor signaling pathway | 2/32 | 38/18614 | 0.001937 | 0.005436 | 0.001643 | MMP9/EGF | 2 |
| BP | GO:0045923 | positive regulation of fatty acid metabolic process | 2/32 | 38/18614 | 0.001937 | 0.005436 | 0.001643 | IL1B/PTGS2 | 2 |
| BP | GO:0051385 | response to mineralocorticoid | 2/32 | 38/18614 | 0.001937 | 0.005436 | 0.001643 | FOS/CCND1 | 2 |
| BP | GO:0060966 | regulation of gene silencing by RNA | 2/32 | 38/18614 | 0.001937 | 0.005436 | 0.001643 | TP53/PPARG | 2 |
| BP | GO:0070229 | negative regulation of lymphocyte apoptotic process | 2/32 | 38/18614 | 0.001937 | 0.005436 | 0.001643 | BCL2/HIF1A | 2 |
| BP | GO:0044403 | biological process involved in symbiotic interaction | 4/32 | 313/18614 | 0.001945 | 0.005436 | 0.001643 | EGFR/CXCL8/ICAM1/CAV1 | 4 |
| BP | GO:0060070 | canonical Wnt signaling pathway | 4/32 | 313/18614 | 0.001945 | 0.005436 | 0.001643 | EGFR/GSK3B/EGF/CAV1 | 4 |
| BP | GO:0035264 | multicellular organism growth | 3/32 | 145/18614 | 0.001946 | 0.005436 | 0.001643 | TP53/BCL2/APP | 3 |
| BP | GO:0050671 | positive regulation of lymphocyte proliferation | 3/32 | 145/18614 | 0.001946 | 0.005436 | 0.001643 | IL1B/BCL2/IL1A | 3 |
| BP | GO:0051783 | regulation of nuclear division | 3/32 | 145/18614 | 0.001946 | 0.005436 | 0.001643 | IL1B/EGF/IL1A | 3 |
| BP | GO:0030198 | extracellular matrix organization | 4/32 | 314/18614 | 0.001968 | 0.005491 | 0.00166 | MMP9/MMP2/APP/CAV1 | 4 |
| BP | GO:0030177 | positive regulation of Wnt signaling pathway | 3/32 | 146/18614 | 0.001985 | 0.00552 | 0.001669 | EGFR/EGF/CAV1 | 3 |
| BP | GO:0045598 | regulation of fat cell differentiation | 3/32 | 146/18614 | 0.001985 | 0.00552 | 0.001669 | AKT1/PTGS2/PPARG | 3 |
| BP | GO:0050714 | positive regulation of protein secretion | 3/32 | 146/18614 | 0.001985 | 0.00552 | 0.001669 | HIF1A/PPARG/IL1A | 3 |
| BP | GO:0009914 | hormone transport | 4/32 | 315/18614 | 0.001991 | 0.005527 | 0.001671 | IL1B/HIF1A/PPARG/IFNG | 4 |
| BP | GO:0043062 | extracellular structure organization | 4/32 | 315/18614 | 0.001991 | 0.005527 | 0.001671 | MMP9/MMP2/APP/CAV1 | 4 |
| BP | GO:0010948 | negative regulation of cell cycle process | 4/32 | 317/18614 | 0.002037 | 0.005626 | 0.001701 | TP53/BCL2/CCND1/CCL2 | 4 |
| BP | GO:0045229 | external encapsulating structure organization | 4/32 | 317/18614 | 0.002037 | 0.005626 | 0.001701 | MMP9/MMP2/APP/CAV1 | 4 |
| BP | GO:0030279 | negative regulation of ossification | 2/32 | 39/18614 | 0.002039 | 0.005626 | 0.001701 | BCL2/HIF1A | 2 |
| BP | GO:0042307 | positive regulation of protein import into nucleus | 2/32 | 39/18614 | 0.002039 | 0.005626 | 0.001701 | PTGS2/IFNG | 2 |
| BP | GO:0045740 | positive regulation of DNA replication | 2/32 | 39/18614 | 0.002039 | 0.005626 | 0.001701 | EGFR/EGF | 2 |
| BP | GO:0060251 | regulation of glial cell proliferation | 2/32 | 39/18614 | 0.002039 | 0.005626 | 0.001701 | IL1B/TP53 | 2 |
| BP | GO:0017015 | regulation of transforming growth factor beta receptor signaling pathway | 3/32 | 148/18614 | 0.002063 | 0.005675 | 0.001716 | TP53/PPARG/CAV1 | 3 |
| BP | GO:0032946 | positive regulation of mononuclear cell proliferation | 3/32 | 148/18614 | 0.002063 | 0.005675 | 0.001716 | IL1B/BCL2/IL1A | 3 |
| BP | GO:0060078 | regulation of postsynaptic membrane potential | 3/32 | 148/18614 | 0.002063 | 0.005675 | 0.001716 | AKT1/GSK3B/APP | 3 |
| BP | GO:0051592 | response to calcium ion | 3/32 | 149/18614 | 0.002103 | 0.005779 | 0.001747 | FOS/CCND1/CAV1 | 3 |
| BP | GO:0072001 | renal system development | 4/32 | 320/18614 | 0.002108 | 0.005787 | 0.00175 | MMP9/BCL2/MYC/CASP9 | 4 |
| BP | GO:0031644 | regulation of nervous system process | 3/32 | 150/18614 | 0.002143 | 0.005844 | 0.001767 | IL10/IL1A/APP | 3 |
| BP | GO:0061351 | neural precursor cell proliferation | 3/32 | 150/18614 | 0.002143 | 0.005844 | 0.001767 | TP53/HIF1A/EGF | 3 |
| BP | GO:0010863 | positive regulation of phospholipase C activity | 2/32 | 40/18614 | 0.002144 | 0.005844 | 0.001767 | ESR1/EGFR | 2 |
| BP | GO:0032570 | response to progesterone | 2/32 | 40/18614 | 0.002144 | 0.005844 | 0.001767 | FOS/CAV1 | 2 |
| BP | GO:0071634 | regulation of transforming growth factor beta production | 2/32 | 40/18614 | 0.002144 | 0.005844 | 0.001767 | PTGS2/HIF1A | 2 |
| BP | GO:1901031 | regulation of response to reactive oxygen species | 2/32 | 40/18614 | 0.002144 | 0.005844 | 0.001767 | IL10/NFE2L2 | 2 |
| BP | GO:1901186 | positive regulation of ERBB signaling pathway | 2/32 | 40/18614 | 0.002144 | 0.005844 | 0.001767 | MMP9/EGF | 2 |
| BP | GO:0031647 | regulation of protein stability | 4/32 | 322/18614 | 0.002157 | 0.005872 | 0.001776 | TP53/CASP3/BCL2/MAPK1 | 4 |
| BP | GO:0008202 | steroid metabolic process | 4/32 | 323/18614 | 0.002181 | 0.00593 | 0.001793 | ESR1/IFNG/IL1A/APP | 4 |
| BP | GO:0044839 | cell cycle G2/M phase transition | 3/32 | 151/18614 | 0.002184 | 0.00593 | 0.001793 | TP53/CCND1/APP | 3 |
| BP | GO:1903844 | regulation of cellular response to transforming growth factor beta stimulus | 3/32 | 151/18614 | 0.002184 | 0.00593 | 0.001793 | TP53/PPARG/CAV1 | 3 |
| BP | GO:0010675 | regulation of cellular carbohydrate metabolic process | 3/32 | 152/18614 | 0.002226 | 0.006036 | 0.001825 | AKT1/TP53/GSK3B | 3 |
| BP | GO:0140467 | integrated stress response signaling | 2/32 | 41/18614 | 0.002252 | 0.0061 | 0.001844 | FOS/NFE2L2 | 2 |
| BP | GO:0007292 | female gamete generation | 3/32 | 153/18614 | 0.002268 | 0.006125 | 0.001852 | PTGS2/BCL2/MMP2 | 3 |
| BP | GO:0030183 | B cell differentiation | 3/32 | 153/18614 | 0.002268 | 0.006125 | 0.001852 | TP53/BCL2/IL10 | 3 |
| BP | GO:0046718 | viral entry into host cell | 3/32 | 153/18614 | 0.002268 | 0.006125 | 0.001852 | EGFR/ICAM1/CAV1 | 3 |
| BP | GO:0048638 | regulation of developmental growth | 4/32 | 328/18614 | 0.002306 | 0.006223 | 0.001882 | AKT1/BCL2/GSK3B/APP | 4 |
| BP | GO:0035148 | tube formation | 3/32 | 154/18614 | 0.00231 | 0.006227 | 0.001883 | CASP3/HIF1A/EGF | 3 |
| BP | GO:0014912 | negative regulation of smooth muscle cell migration | 2/32 | 42/18614 | 0.002362 | 0.006335 | 0.001915 | NFE2L2/SERPINE1 | 2 |
| BP | GO:0071542 | dopaminergic neuron differentiation | 2/32 | 42/18614 | 0.002362 | 0.006335 | 0.001915 | HIF1A/GSK3B | 2 |
| BP | GO:1900274 | regulation of phospholipase C activity | 2/32 | 42/18614 | 0.002362 | 0.006335 | 0.001915 | ESR1/EGFR | 2 |
| BP | GO:1904037 | positive regulation of epithelial cell apoptotic process | 2/32 | 42/18614 | 0.002362 | 0.006335 | 0.001915 | CCL2/HMOX1 | 2 |
| BP | GO:2000008 | regulation of protein localization to cell surface | 2/32 | 42/18614 | 0.002362 | 0.006335 | 0.001915 | AKT1/EGF | 2 |
| BP | GO:0008643 | carbohydrate transport | 3/32 | 156/18614 | 0.002396 | 0.006421 | 0.001942 | AKT1/IL1B/NFE2L2 | 3 |
| BP | GO:0030902 | hindbrain development | 3/32 | 157/18614 | 0.00244 | 0.006533 | 0.001975 | TP53/BCL2/EGF | 3 |
| BP | GO:0032735 | positive regulation of interleukin-12 production | 2/32 | 43/18614 | 0.002474 | 0.006611 | 0.001999 | IFNG/RELA | 2 |
| BP | GO:0071604 | transforming growth factor beta production | 2/32 | 43/18614 | 0.002474 | 0.006611 | 0.001999 | PTGS2/HIF1A | 2 |
| BP | GO:0010828 | positive regulation of glucose transmembrane transport | 2/32 | 44/18614 | 0.002589 | 0.006905 | 0.002088 | AKT1/NFE2L2 | 2 |
| BP | GO:0042771 | intrinsic apoptotic signaling pathway in response to DNA damage by p53 class mediator | 2/32 | 44/18614 | 0.002589 | 0.006905 | 0.002088 | TP53/BCL2 | 2 |
| BP | GO:0005978 | glycogen biosynthetic process | 2/32 | 45/18614 | 0.002707 | 0.007169 | 0.002168 | AKT1/GSK3B | 2 |
| BP | GO:0009250 | glucan biosynthetic process | 2/32 | 45/18614 | 0.002707 | 0.007169 | 0.002168 | AKT1/GSK3B | 2 |
| BP | GO:0030574 | collagen catabolic process | 2/32 | 45/18614 | 0.002707 | 0.007169 | 0.002168 | MMP9/MMP2 | 2 |
| BP | GO:0031295 | T cell costimulation | 2/32 | 45/18614 | 0.002707 | 0.007169 | 0.002168 | AKT1/CAV1 | 2 |
| BP | GO:0060443 | mammary gland morphogenesis | 2/32 | 45/18614 | 0.002707 | 0.007169 | 0.002168 | ESR1/CAV1 | 2 |
| BP | GO:1902622 | regulation of neutrophil migration | 2/32 | 45/18614 | 0.002707 | 0.007169 | 0.002168 | CXCL8/IL1A | 2 |
| BP | GO:1902692 | regulation of neuroblast proliferation | 2/32 | 45/18614 | 0.002707 | 0.007169 | 0.002168 | TP53/HIF1A | 2 |
| BP | GO:0050796 | regulation of insulin secretion | 3/32 | 163/18614 | 0.002714 | 0.00718 | 0.002171 | IL1B/HIF1A/IFNG | 3 |
| BP | GO:1904062 | regulation of monoatomic cation transmembrane transport | 4/32 | 346/18614 | 0.002797 | 0.007393 | 0.002235 | MMP9/BCL2/IFNG/CAV1 | 4 |
| BP | GO:0001959 | regulation of cytokine-mediated signaling pathway | 3/32 | 165/18614 | 0.002809 | 0.00741 | 0.00224 | HIF1A/PPARG/CAV1 | 3 |
| BP | GO:0007519 | skeletal muscle tissue development | 3/32 | 165/18614 | 0.002809 | 0.00741 | 0.00224 | BCL2/FOS/CAV1 | 3 |
| BP | GO:0008038 | neuron recognition | 2/32 | 46/18614 | 0.002827 | 0.007429 | 0.002246 | CASP3/APP | 2 |
| BP | GO:0042551 | neuron maturation | 2/32 | 46/18614 | 0.002827 | 0.007429 | 0.002246 | BCL2/APP | 2 |
| BP | GO:0070266 | necroptotic process | 2/32 | 46/18614 | 0.002827 | 0.007429 | 0.002246 | TP53/CAV1 | 2 |
| BP | GO:1990090 | cellular response to nerve growth factor stimulus | 2/32 | 46/18614 | 0.002827 | 0.007429 | 0.002246 | AKT1/APP | 2 |
| BP | GO:0001774 | microglial cell activation | 2/32 | 47/18614 | 0.002949 | 0.007706 | 0.00233 | IFNG/APP | 2 |
| BP | GO:0002701 | negative regulation of production of molecular mediator of immune response | 2/32 | 47/18614 | 0.002949 | 0.007706 | 0.00233 | IL10/HMOX1 | 2 |
| BP | GO:0031018 | endocrine pancreas development | 2/32 | 47/18614 | 0.002949 | 0.007706 | 0.00233 | AKT1/GSK3B | 2 |
| BP | GO:0031294 | lymphocyte costimulation | 2/32 | 47/18614 | 0.002949 | 0.007706 | 0.00233 | AKT1/CAV1 | 2 |
| BP | GO:0032369 | negative regulation of lipid transport | 2/32 | 47/18614 | 0.002949 | 0.007706 | 0.00233 | AKT1/EGF | 2 |
| BP | GO:1900271 | regulation of long-term synaptic potentiation | 2/32 | 47/18614 | 0.002949 | 0.007706 | 0.00233 | GSK3B/APP | 2 |
| BP | GO:0051250 | negative regulation of lymphocyte activation | 3/32 | 168/18614 | 0.002955 | 0.007714 | 0.002333 | CASP3/ERBB2/IL10 | 3 |
| BP | GO:0090101 | negative regulation of transmembrane receptor protein serine/threonine kinase signaling pathway | 3/32 | 169/18614 | 0.003005 | 0.007837 | 0.00237 | TP53/PPARG/CAV1 | 3 |
| BP | GO:0002673 | regulation of acute inflammatory response | 2/32 | 48/18614 | 0.003074 | 0.007955 | 0.002405 | IL1B/PTGS2 | 2 |
| BP | GO:0008542 | visual learning | 2/32 | 48/18614 | 0.003074 | 0.007955 | 0.002405 | HIF1A/APP | 2 |
| BP | GO:0030850 | prostate gland development | 2/32 | 48/18614 | 0.003074 | 0.007955 | 0.002405 | ESR1/MMP2 | 2 |
| BP | GO:0035794 | positive regulation of mitochondrial membrane permeability | 2/32 | 48/18614 | 0.003074 | 0.007955 | 0.002405 | TP53/GSK3B | 2 |
| BP | GO:0035987 | endodermal cell differentiation | 2/32 | 48/18614 | 0.003074 | 0.007955 | 0.002405 | MMP9/MMP2 | 2 |
| BP | GO:0048538 | thymus development | 2/32 | 48/18614 | 0.003074 | 0.007955 | 0.002405 | BCL2/MAPK1 | 2 |
| BP | GO:0070509 | calcium ion import | 2/32 | 48/18614 | 0.003074 | 0.007955 | 0.002405 | EGF/CCL2 | 2 |
| BP | GO:1990089 | response to nerve growth factor | 2/32 | 48/18614 | 0.003074 | 0.007955 | 0.002405 | AKT1/APP | 2 |
| BP | GO:0002262 | myeloid cell homeostasis | 3/32 | 171/18614 | 0.003107 | 0.008024 | 0.002426 | CASP3/HIF1A/HMOX1 | 3 |
| BP | GO:0010950 | positive regulation of endopeptidase activity | 3/32 | 171/18614 | 0.003107 | 0.008024 | 0.002426 | MYC/PPARG/CASP9 | 3 |
| BP | GO:0001837 | epithelial to mesenchymal transition | 3/32 | 172/18614 | 0.003158 | 0.00815 | 0.002464 | IL1B/HIF1A/GSK3B | 3 |
| BP | GO:0007528 | neuromuscular junction development | 2/32 | 49/18614 | 0.003202 | 0.008207 | 0.002481 | ERBB2/APP | 2 |
| BP | GO:0010665 | regulation of cardiac muscle cell apoptotic process | 2/32 | 49/18614 | 0.003202 | 0.008207 | 0.002481 | TP53/NFE2L2 | 2 |
| BP | GO:0032892 | positive regulation of organic acid transport | 2/32 | 49/18614 | 0.003202 | 0.008207 | 0.002481 | IL1B/IL1A | 2 |
| BP | GO:0043330 | response to exogenous dsRNA | 2/32 | 49/18614 | 0.003202 | 0.008207 | 0.002481 | MAPK1/CAV1 | 2 |
| BP | GO:0061028 | establishment of endothelial barrier | 2/32 | 49/18614 | 0.003202 | 0.008207 | 0.002481 | IL1B/ICAM1 | 2 |
| BP | GO:0071470 | cellular response to osmotic stress | 2/32 | 49/18614 | 0.003202 | 0.008207 | 0.002481 | PTGS2/CASP3 | 2 |
| BP | GO:1902108 | regulation of mitochondrial membrane permeability involved in apoptotic process | 2/32 | 49/18614 | 0.003202 | 0.008207 | 0.002481 | TP53/GSK3B | 2 |
| BP | GO:0034767 | positive regulation of monoatomic ion transmembrane transport | 3/32 | 175/18614 | 0.003316 | 0.008483 | 0.002565 | CCL2/IFNG/CAV1 | 3 |
| BP | GO:0002269 | leukocyte activation involved in inflammatory response | 2/32 | 50/18614 | 0.003331 | 0.008483 | 0.002565 | IFNG/APP | 2 |
| BP | GO:0006692 | prostanoid metabolic process | 2/32 | 50/18614 | 0.003331 | 0.008483 | 0.002565 | IL1B/PTGS2 | 2 |
| BP | GO:0006693 | prostaglandin metabolic process | 2/32 | 50/18614 | 0.003331 | 0.008483 | 0.002565 | IL1B/PTGS2 | 2 |
| BP | GO:0042304 | regulation of fatty acid biosynthetic process | 2/32 | 50/18614 | 0.003331 | 0.008483 | 0.002565 | IL1B/PTGS2 | 2 |
| BP | GO:0043457 | regulation of cellular respiration | 2/32 | 50/18614 | 0.003331 | 0.008483 | 0.002565 | HIF1A/IFNG | 2 |
| BP | GO:0048009 | insulin-like growth factor receptor signaling pathway | 2/32 | 50/18614 | 0.003331 | 0.008483 | 0.002565 | AKT1/MAPK1 | 2 |
| BP | GO:0050680 | negative regulation of epithelial cell proliferation | 3/32 | 177/18614 | 0.003424 | 0.008702 | 0.002631 | PPARG/CCL2/CAV1 | 3 |
| BP | GO:0060759 | regulation of response to cytokine stimulus | 3/32 | 177/18614 | 0.003424 | 0.008702 | 0.002631 | HIF1A/PPARG/CAV1 | 3 |
| BP | GO:0009299 | mRNA transcription | 2/32 | 51/18614 | 0.003464 | 0.008771 | 0.002652 | TP53/PPARG | 2 |
| BP | GO:0010662 | regulation of striated muscle cell apoptotic process | 2/32 | 51/18614 | 0.003464 | 0.008771 | 0.002652 | TP53/NFE2L2 | 2 |
| BP | GO:0010939 | regulation of necrotic cell death | 2/32 | 51/18614 | 0.003464 | 0.008771 | 0.002652 | TP53/CAV1 | 2 |
| BP | GO:0045912 | negative regulation of carbohydrate metabolic process | 2/32 | 51/18614 | 0.003464 | 0.008771 | 0.002652 | TP53/GSK3B | 2 |
| BP | GO:0060538 | skeletal muscle organ development | 3/32 | 178/18614 | 0.003478 | 0.008799 | 0.002661 | BCL2/FOS/CAV1 | 3 |
| BP | GO:0007204 | positive regulation of cytosolic calcium ion concentration | 3/32 | 179/18614 | 0.003533 | 0.008931 | 0.0027 | ESR1/BCL2/CAV1 | 3 |
| BP | GO:0035265 | organ growth | 3/32 | 180/18614 | 0.003589 | 0.009019 | 0.002727 | AKT1/ESR1/BCL2 | 3 |
| BP | GO:0051048 | negative regulation of secretion | 3/32 | 180/18614 | 0.003589 | 0.009019 | 0.002727 | IL1B/EGF/HMOX1 | 3 |
| BP | GO:0006636 | unsaturated fatty acid biosynthetic process | 2/32 | 52/18614 | 0.003598 | 0.009019 | 0.002727 | IL1B/PTGS2 | 2 |
| BP | GO:0010656 | negative regulation of muscle cell apoptotic process | 2/32 | 52/18614 | 0.003598 | 0.009019 | 0.002727 | HMOX1/NFE2L2 | 2 |
| BP | GO:0031103 | axon regeneration | 2/32 | 52/18614 | 0.003598 | 0.009019 | 0.002727 | BCL2/MMP2 | 2 |
| BP | GO:0048013 | ephrin receptor signaling pathway | 2/32 | 52/18614 | 0.003598 | 0.009019 | 0.002727 | MMP9/MMP2 | 2 |
| BP | GO:0060324 | face development | 2/32 | 52/18614 | 0.003598 | 0.009019 | 0.002727 | MMP2/MAPK1 | 2 |
| BP | GO:0101023 | vascular endothelial cell proliferation | 2/32 | 52/18614 | 0.003598 | 0.009019 | 0.002727 | PPARG/CCL2 | 2 |
| BP | GO:1905562 | regulation of vascular endothelial cell proliferation | 2/32 | 52/18614 | 0.003598 | 0.009019 | 0.002727 | PPARG/CCL2 | 2 |
| BP | GO:0050731 | positive regulation of peptidyl-tyrosine phosphorylation | 3/32 | 181/18614 | 0.003645 | 0.009128 | 0.00276 | TP53/EGF/IFNG | 3 |
| BP | GO:0010659 | cardiac muscle cell apoptotic process | 2/32 | 53/18614 | 0.003735 | 0.009311 | 0.002815 | TP53/NFE2L2 | 2 |
| BP | GO:0048260 | positive regulation of receptor-mediated endocytosis | 2/32 | 53/18614 | 0.003735 | 0.009311 | 0.002815 | EGF/SERPINE1 | 2 |
| BP | GO:0071622 | regulation of granulocyte chemotaxis | 2/32 | 53/18614 | 0.003735 | 0.009311 | 0.002815 | CXCL8/MAPK1 | 2 |
| BP | GO:0097300 | programmed necrotic cell death | 2/32 | 53/18614 | 0.003735 | 0.009311 | 0.002815 | TP53/CAV1 | 2 |
| BP | GO:1905710 | positive regulation of membrane permeability | 2/32 | 53/18614 | 0.003735 | 0.009311 | 0.002815 | TP53/GSK3B | 2 |
| BP | GO:0042770 | signal transduction in response to DNA damage | 3/32 | 184/18614 | 0.003817 | 0.009489 | 0.002869 | TP53/CCND1/CASP9 | 3 |
| BP | GO:1902107 | positive regulation of leukocyte differentiation | 3/32 | 184/18614 | 0.003817 | 0.009489 | 0.002869 | FOS/IL10/IFNG | 3 |
| BP | GO:1903708 | positive regulation of hemopoiesis | 3/32 | 184/18614 | 0.003817 | 0.009489 | 0.002869 | FOS/IL10/IFNG | 3 |
| BP | GO:0007632 | visual behavior | 2/32 | 54/18614 | 0.003875 | 0.009599 | 0.002902 | HIF1A/APP | 2 |
| BP | GO:0014009 | glial cell proliferation | 2/32 | 54/18614 | 0.003875 | 0.009599 | 0.002902 | IL1B/TP53 | 2 |
| BP | GO:2000179 | positive regulation of neural precursor cell proliferation | 2/32 | 54/18614 | 0.003875 | 0.009599 | 0.002902 | HIF1A/EGF | 2 |
| BP | GO:0010952 | positive regulation of peptidase activity | 3/32 | 185/18614 | 0.003876 | 0.009599 | 0.002902 | MYC/PPARG/CASP9 | 3 |
| BP | GO:0050767 | regulation of neurogenesis | 4/32 | 381/18614 | 0.00395 | 0.009773 | 0.002955 | IL1B/TP53/HIF1A/IFNG | 4 |
| BP | GO:0008217 | regulation of blood pressure | 3/32 | 187/18614 | 0.003994 | 0.009866 | 0.002983 | PTGS2/PPARG/HMOX1 | 3 |
| BP | GO:0051302 | regulation of cell division | 3/32 | 187/18614 | 0.003994 | 0.009866 | 0.002983 | IL1B/MYC/IL1A | 3 |
| BP | GO:0010518 | positive regulation of phospholipase activity | 2/32 | 55/18614 | 0.004017 | 0.009886 | 0.002989 | ESR1/EGFR | 2 |
| BP | GO:0010658 | striated muscle cell apoptotic process | 2/32 | 55/18614 | 0.004017 | 0.009886 | 0.002989 | TP53/NFE2L2 | 2 |
| BP | GO:0050805 | negative regulation of synaptic transmission | 2/32 | 55/18614 | 0.004017 | 0.009886 | 0.002989 | IL1B/PTGS2 | 2 |
| BP | GO:0052372 | modulation by symbiont of entry into host | 2/32 | 55/18614 | 0.004017 | 0.009886 | 0.002989 | CXCL8/CAV1 | 2 |
| BP | GO:0002221 | pattern recognition receptor signaling pathway | 3/32 | 189/18614 | 0.004115 | 0.010099 | 0.003054 | ESR1/RELA/CAV1 | 3 |
| BP | GO:0002700 | regulation of production of molecular mediator of immune response | 3/32 | 189/18614 | 0.004115 | 0.010099 | 0.003054 | IL1B/IL10/HMOX1 | 3 |
| BP | GO:0006839 | mitochondrial transport | 3/32 | 189/18614 | 0.004115 | 0.010099 | 0.003054 | TP53/BCL2/GSK3B | 3 |
| BP | GO:0043331 | response to dsRNA | 2/32 | 56/18614 | 0.004161 | 0.010185 | 0.00308 | MAPK1/CAV1 | 2 |
| BP | GO:0060986 | endocrine hormone secretion | 2/32 | 56/18614 | 0.004161 | 0.010185 | 0.00308 | IL1B/PPARG | 2 |
| BP | GO:2000107 | negative regulation of leukocyte apoptotic process | 2/32 | 56/18614 | 0.004161 | 0.010185 | 0.00308 | BCL2/HIF1A | 2 |
| BP | GO:0050777 | negative regulation of immune response | 3/32 | 191/18614 | 0.004238 | 0.010363 | 0.003133 | PPARG/IL10/HMOX1 | 3 |
| BP | GO:0090276 | regulation of peptide hormone secretion | 3/32 | 192/18614 | 0.0043 | 0.010506 | 0.003177 | IL1B/HIF1A/IFNG | 3 |
| BP | GO:0001706 | endoderm formation | 2/32 | 57/18614 | 0.004308 | 0.010516 | 0.00318 | MMP9/MMP2 | 2 |
| BP | GO:0043433 | negative regulation of DNA-binding transcription factor activity | 3/32 | 194/18614 | 0.004426 | 0.010785 | 0.003261 | ESR1/IL10/HMOX1 | 3 |
| BP | GO:0045664 | regulation of neuron differentiation | 3/32 | 194/18614 | 0.004426 | 0.010785 | 0.003261 | BCL2/GSK3B/APP | 3 |
| BP | GO:0006631 | fatty acid metabolic process | 4/32 | 394/18614 | 0.004449 | 0.010821 | 0.003272 | AKT1/IL1B/PTGS2/CAV1 | 4 |
| BP | GO:0042306 | regulation of protein import into nucleus | 2/32 | 58/18614 | 0.004457 | 0.010821 | 0.003272 | PTGS2/IFNG | 2 |
| BP | GO:0048010 | vascular endothelial growth factor receptor signaling pathway | 2/32 | 58/18614 | 0.004457 | 0.010821 | 0.003272 | IL1B/HIF1A | 2 |
| BP | GO:0090311 | regulation of protein deacetylation | 2/32 | 58/18614 | 0.004457 | 0.010821 | 0.003272 | TP53/IFNG | 2 |
| BP | GO:0002791 | regulation of peptide secretion | 3/32 | 195/18614 | 0.00449 | 0.010892 | 0.003293 | IL1B/HIF1A/IFNG | 3 |
| BP | GO:0002763 | positive regulation of myeloid leukocyte differentiation | 2/32 | 59/18614 | 0.004609 | 0.011126 | 0.003364 | FOS/IFNG | 2 |
| BP | GO:0006611 | protein export from nucleus | 2/32 | 59/18614 | 0.004609 | 0.011126 | 0.003364 | IL1B/GSK3B | 2 |
| BP | GO:0031102 | neuron projection regeneration | 2/32 | 59/18614 | 0.004609 | 0.011126 | 0.003364 | BCL2/MMP2 | 2 |
| BP | GO:0046324 | regulation of glucose import | 2/32 | 59/18614 | 0.004609 | 0.011126 | 0.003364 | AKT1/NFE2L2 | 2 |
| BP | GO:2001258 | negative regulation of cation channel activity | 2/32 | 59/18614 | 0.004609 | 0.011126 | 0.003364 | MMP9/CAV1 | 2 |
| BP | GO:0007178 | transmembrane receptor protein serine/threonine kinase signaling pathway | 4/32 | 398/18614 | 0.004611 | 0.011126 | 0.003364 | TP53/FOS/PPARG/CAV1 | 4 |
| BP | GO:0030073 | insulin secretion | 3/32 | 197/18614 | 0.00462 | 0.011127 | 0.003364 | IL1B/HIF1A/IFNG | 3 |
| BP | GO:0090087 | regulation of peptide transport | 3/32 | 197/18614 | 0.00462 | 0.011127 | 0.003364 | IL1B/HIF1A/IFNG | 3 |
| BP | GO:0071897 | DNA biosynthetic process | 3/32 | 198/18614 | 0.004685 | 0.011275 | 0.003409 | TP53/MYC/MAPK1 | 3 |
| BP | GO:0002443 | leukocyte mediated immunity | 4/32 | 401/18614 | 0.004735 | 0.011385 | 0.003442 | IL1B/IL10/ICAM1/HMOX1 | 4 |
| BP | GO:0009746 | response to hexose | 3/32 | 199/18614 | 0.004752 | 0.011414 | 0.003451 | PTGS2/CASP3/HIF1A | 3 |
| BP | GO:0001658 | branching involved in ureteric bud morphogenesis | 2/32 | 60/18614 | 0.004762 | 0.01142 | 0.003453 | BCL2/MYC | 2 |
| BP | GO:0019748 | secondary metabolic process | 2/32 | 60/18614 | 0.004762 | 0.01142 | 0.003453 | BCL2/NFE2L2 | 2 |
| BP | GO:0050851 | antigen receptor-mediated signaling pathway | 3/32 | 201/18614 | 0.004886 | 0.011705 | 0.003539 | BCL2/RELA/MAPK1 | 3 |
| BP | GO:0034113 | heterotypic cell-cell adhesion | 2/32 | 61/18614 | 0.004919 | 0.011742 | 0.00355 | IL1B/IL10 | 2 |
| BP | GO:0043903 | regulation of biological process involved in symbiotic interaction | 2/32 | 61/18614 | 0.004919 | 0.011742 | 0.00355 | CXCL8/CAV1 | 2 |
| BP | GO:0046456 | icosanoid biosynthetic process | 2/32 | 61/18614 | 0.004919 | 0.011742 | 0.00355 | IL1B/PTGS2 | 2 |
| BP | GO:0050994 | regulation of lipid catabolic process | 2/32 | 61/18614 | 0.004919 | 0.011742 | 0.00355 | AKT1/IL1B | 2 |
| BP | GO:0022617 | extracellular matrix disassembly | 2/32 | 62/18614 | 0.005077 | 0.012078 | 0.003652 | MMP9/MMP2 | 2 |
| BP | GO:0060261 | positive regulation of transcription initiation by RNA polymerase II | 2/32 | 62/18614 | 0.005077 | 0.012078 | 0.003652 | TP53/ESR1 | 2 |
| BP | GO:0090303 | positive regulation of wound healing | 2/32 | 62/18614 | 0.005077 | 0.012078 | 0.003652 | NFE2L2/SERPINE1 | 2 |
| BP | GO:0043254 | regulation of protein-containing complex assembly | 4/32 | 409/18614 | 0.005077 | 0.012078 | 0.003652 | TP53/ESR1/GSK3B/IFNG | 4 |
| BP | GO:0001885 | endothelial cell development | 2/32 | 63/18614 | 0.005238 | 0.012385 | 0.003745 | IL1B/ICAM1 | 2 |
| BP | GO:0031638 | zymogen activation | 2/32 | 63/18614 | 0.005238 | 0.012385 | 0.003745 | CASP9/SERPINE1 | 2 |
| BP | GO:0032623 | interleukin-2 production | 2/32 | 63/18614 | 0.005238 | 0.012385 | 0.003745 | IL1B/IL1A | 2 |
| BP | GO:0032663 | regulation of interleukin-2 production | 2/32 | 63/18614 | 0.005238 | 0.012385 | 0.003745 | IL1B/IL1A | 2 |
| BP | GO:0032731 | positive regulation of interleukin-1 beta production | 2/32 | 63/18614 | 0.005238 | 0.012385 | 0.003745 | IFNG/APP | 2 |
| BP | GO:0033692 | cellular polysaccharide biosynthetic process | 2/32 | 63/18614 | 0.005238 | 0.012385 | 0.003745 | AKT1/GSK3B | 2 |
| BP | GO:0070059 | intrinsic apoptotic signaling pathway in response to endoplasmic reticulum stress | 2/32 | 63/18614 | 0.005238 | 0.012385 | 0.003745 | TP53/BCL2 | 2 |
| BP | GO:0007623 | circadian rhythm | 3/32 | 207/18614 | 0.005302 | 0.012525 | 0.003787 | TP53/PPARG/GSK3B | 3 |
| BP | GO:0006940 | regulation of smooth muscle contraction | 2/32 | 64/18614 | 0.005401 | 0.012737 | 0.003851 | PTGS2/CAV1 | 2 |
| BP | GO:0040014 | regulation of multicellular organism growth | 2/32 | 64/18614 | 0.005401 | 0.012737 | 0.003851 | BCL2/APP | 2 |
| BP | GO:0002758 | innate immune response-activating signaling pathway | 3/32 | 209/18614 | 0.005445 | 0.01283 | 0.003879 | ESR1/RELA/CAV1 | 3 |
| BP | GO:0032507 | maintenance of protein location in cell | 2/32 | 65/18614 | 0.005567 | 0.013093 | 0.003959 | AKT1/CAV1 | 2 |
| BP | GO:1905330 | regulation of morphogenesis of an epithelium | 2/32 | 65/18614 | 0.005567 | 0.013093 | 0.003959 | ESR1/EGF | 2 |
| BP | GO:0045619 | regulation of lymphocyte differentiation | 3/32 | 212/18614 | 0.005664 | 0.013312 | 0.004025 | ERBB2/IL10/IFNG | 3 |
| BP | GO:0001655 | urogenital system development | 2/32 | 66/18614 | 0.005734 | 0.013441 | 0.004064 | ESR1/MMP2 | 2 |
| BP | GO:0010517 | regulation of phospholipase activity | 2/32 | 66/18614 | 0.005734 | 0.013441 | 0.004064 | ESR1/EGFR | 2 |
| BP | GO:0060675 | ureteric bud morphogenesis | 2/32 | 66/18614 | 0.005734 | 0.013441 | 0.004064 | BCL2/MYC | 2 |
| BP | GO:0016051 | carbohydrate biosynthetic process | 3/32 | 214/18614 | 0.005813 | 0.013591 | 0.004109 | AKT1/GSK3B/EGF | 3 |
| BP | GO:0030100 | regulation of endocytosis | 3/32 | 214/18614 | 0.005813 | 0.013591 | 0.004109 | EGF/SERPINE1/CAV1 | 3 |
| BP | GO:1901888 | regulation of cell junction assembly | 3/32 | 214/18614 | 0.005813 | 0.013591 | 0.004109 | IL1B/APP/CAV1 | 3 |
| BP | GO:0060193 | positive regulation of lipase activity | 2/32 | 67/18614 | 0.005905 | 0.013774 | 0.004165 | ESR1/EGFR | 2 |
| BP | GO:0072171 | mesonephric tubule morphogenesis | 2/32 | 67/18614 | 0.005905 | 0.013774 | 0.004165 | BCL2/MYC | 2 |
| BP | GO:0007409 | axonogenesis | 4/32 | 427/18614 | 0.005907 | 0.013774 | 0.004165 | BCL2/GSK3B/ERBB2/APP | 4 |
| BP | GO:0015909 | long-chain fatty acid transport | 2/32 | 68/18614 | 0.006077 | 0.014122 | 0.00427 | AKT1/PPARG | 2 |
| BP | GO:0045670 | regulation of osteoclast differentiation | 2/32 | 68/18614 | 0.006077 | 0.014122 | 0.00427 | FOS/IFNG | 2 |
| BP | GO:0051123 | RNA polymerase II preinitiation complex assembly | 2/32 | 68/18614 | 0.006077 | 0.014122 | 0.00427 | TP53/ESR1 | 2 |
| BP | GO:2000144 | positive regulation of DNA-templated transcription initiation | 2/32 | 68/18614 | 0.006077 | 0.014122 | 0.00427 | TP53/ESR1 | 2 |
| BP | GO:0046034 | ATP metabolic process | 3/32 | 219/18614 | 0.006196 | 0.014388 | 0.00435 | HIF1A/IFNG/APP | 3 |
| BP | GO:0006305 | DNA alkylation | 2/32 | 70/18614 | 0.006429 | 0.014861 | 0.004493 | FOS/MYC | 2 |
| BP | GO:0006306 | DNA methylation | 2/32 | 70/18614 | 0.006429 | 0.014861 | 0.004493 | FOS/MYC | 2 |
| BP | GO:0034394 | protein localization to cell surface | 2/32 | 70/18614 | 0.006429 | 0.014861 | 0.004493 | AKT1/EGF | 2 |
| BP | GO:0051881 | regulation of mitochondrial membrane potential | 2/32 | 70/18614 | 0.006429 | 0.014861 | 0.004493 | AKT1/BCL2 | 2 |
| BP | GO:2000379 | positive regulation of reactive oxygen species metabolic process | 2/32 | 70/18614 | 0.006429 | 0.014861 | 0.004493 | TP53/NFE2L2 | 2 |
| BP | GO:0010810 | regulation of cell-substrate adhesion | 3/32 | 222/18614 | 0.006433 | 0.014861 | 0.004493 | BCL2/GSK3B/SERPINE1 | 3 |
| BP | GO:0043161 | proteasome-mediated ubiquitin-dependent protein catabolic process | 4/32 | 439/18614 | 0.006507 | 0.015019 | 0.004541 | AKT1/GSK3B/NFE2L2/CAV1 | 4 |
| BP | GO:0007596 | blood coagulation | 3/32 | 224/18614 | 0.006594 | 0.0152 | 0.004596 | NFE2L2/SERPINE1/CAV1 | 3 |
| BP | GO:0002548 | monocyte chemotaxis | 2/32 | 71/18614 | 0.006608 | 0.0152 | 0.004596 | CCL2/SERPINE1 | 2 |
| BP | GO:0070265 | necrotic cell death | 2/32 | 71/18614 | 0.006608 | 0.0152 | 0.004596 | TP53/CAV1 | 2 |
| BP | GO:0072678 | T cell migration | 2/32 | 71/18614 | 0.006608 | 0.0152 | 0.004596 | CCL2/APP | 2 |
| BP | GO:0034329 | cell junction assembly | 4/32 | 444/18614 | 0.006769 | 0.015539 | 0.004698 | IL1B/BCL2/APP/CAV1 | 4 |
| BP | GO:0006024 | glycosaminoglycan biosynthetic process | 2/32 | 72/18614 | 0.00679 | 0.015539 | 0.004698 | IL1B/EGF | 2 |
| BP | GO:0045665 | negative regulation of neuron differentiation | 2/32 | 72/18614 | 0.00679 | 0.015539 | 0.004698 | GSK3B/APP | 2 |
| BP | GO:0051926 | negative regulation of calcium ion transport | 2/32 | 72/18614 | 0.00679 | 0.015539 | 0.004698 | PTGS2/BCL2 | 2 |
| BP | GO:0060260 | regulation of transcription initiation by RNA polymerase II | 2/32 | 72/18614 | 0.00679 | 0.015539 | 0.004698 | TP53/ESR1 | 2 |
| BP | GO:0071677 | positive regulation of mononuclear cell migration | 2/32 | 72/18614 | 0.00679 | 0.015539 | 0.004698 | APP/SERPINE1 | 2 |
| BP | GO:0032102 | negative regulation of response to external stimulus | 4/32 | 446/18614 | 0.006876 | 0.015723 | 0.004754 | PPARG/IL10/CCL2/SERPINE1 | 4 |
| BP | GO:0007405 | neuroblast proliferation | 2/32 | 73/18614 | 0.006973 | 0.015905 | 0.004809 | TP53/HIF1A | 2 |
| BP | GO:0071260 | cellular response to mechanical stimulus | 2/32 | 73/18614 | 0.006973 | 0.015905 | 0.004809 | IL1B/PTGS2 | 2 |
| BP | GO:1900076 | regulation of cellular response to insulin stimulus | 2/32 | 73/18614 | 0.006973 | 0.015905 | 0.004809 | IL1B/PPARG | 2 |
| BP | GO:0050817 | coagulation | 3/32 | 229/18614 | 0.007007 | 0.015969 | 0.004828 | NFE2L2/SERPINE1/CAV1 | 3 |
| BP | GO:0007599 | hemostasis | 3/32 | 230/18614 | 0.007092 | 0.016148 | 0.004883 | NFE2L2/SERPINE1/CAV1 | 3 |
| BP | GO:0006023 | aminoglycan biosynthetic process | 2/32 | 74/18614 | 0.007159 | 0.016234 | 0.004909 | IL1B/EGF | 2 |
| BP | GO:0032732 | positive regulation of interleukin-1 production | 2/32 | 74/18614 | 0.007159 | 0.016234 | 0.004909 | IFNG/APP | 2 |
| BP | GO:0035924 | cellular response to vascular endothelial growth factor stimulus | 2/32 | 74/18614 | 0.007159 | 0.016234 | 0.004909 | AKT1/RELA | 2 |
| BP | GO:0045123 | cellular extravasation | 2/32 | 74/18614 | 0.007159 | 0.016234 | 0.004909 | CCL2/ICAM1 | 2 |
| BP | GO:2000573 | positive regulation of DNA biosynthetic process | 2/32 | 74/18614 | 0.007159 | 0.016234 | 0.004909 | MYC/MAPK1 | 2 |
| BP | GO:0072594 | establishment of protein localization to organelle | 4/32 | 453/18614 | 0.007258 | 0.01644 | 0.004971 | AKT1/TP53/PTGS2/IFNG | 4 |
| BP | GO:0050769 | positive regulation of neurogenesis | 3/32 | 232/18614 | 0.007262 | 0.01644 | 0.004971 | IL1B/HIF1A/IFNG | 3 |
| BP | GO:0005977 | glycogen metabolic process | 2/32 | 75/18614 | 0.007348 | 0.016525 | 0.004996 | AKT1/GSK3B | 2 |
| BP | GO:0016239 | positive regulation of macroautophagy | 2/32 | 75/18614 | 0.007348 | 0.016525 | 0.004996 | HIF1A/HMOX1 | 2 |
| BP | GO:0030968 | endoplasmic reticulum unfolded protein response | 2/32 | 75/18614 | 0.007348 | 0.016525 | 0.004996 | CCND1/NFE2L2 | 2 |
| BP | GO:0034637 | cellular carbohydrate biosynthetic process | 2/32 | 75/18614 | 0.007348 | 0.016525 | 0.004996 | AKT1/GSK3B | 2 |
| BP | GO:0046323 | glucose import | 2/32 | 75/18614 | 0.007348 | 0.016525 | 0.004996 | AKT1/NFE2L2 | 2 |
| BP | GO:0051899 | membrane depolarization | 2/32 | 75/18614 | 0.007348 | 0.016525 | 0.004996 | BCL2/CAV1 | 2 |
| BP | GO:0072078 | nephron tubule morphogenesis | 2/32 | 75/18614 | 0.007348 | 0.016525 | 0.004996 | BCL2/MYC | 2 |
| BP | GO:0002218 | activation of innate immune response | 3/32 | 233/18614 | 0.007348 | 0.016525 | 0.004996 | ESR1/RELA/CAV1 | 3 |
| BP | GO:0050807 | regulation of synapse organization | 3/32 | 234/18614 | 0.007435 | 0.016692 | 0.005047 | AKT1/IL10/APP | 3 |
| BP | GO:0060560 | developmental growth involved in morphogenesis | 3/32 | 234/18614 | 0.007435 | 0.016692 | 0.005047 | ESR1/GSK3B/APP | 3 |
| BP | GO:0006073 | cellular glucan metabolic process | 2/32 | 76/18614 | 0.007538 | 0.01684 | 0.005092 | AKT1/GSK3B | 2 |
| BP | GO:0006919 | activation of cysteine-type endopeptidase activity involved in apoptotic process | 2/32 | 76/18614 | 0.007538 | 0.01684 | 0.005092 | PPARG/CASP9 | 2 |
| BP | GO:0007422 | peripheral nervous system development | 2/32 | 76/18614 | 0.007538 | 0.01684 | 0.005092 | AKT1/ERBB2 | 2 |
| BP | GO:0044042 | glucan metabolic process | 2/32 | 76/18614 | 0.007538 | 0.01684 | 0.005092 | AKT1/GSK3B | 2 |
| BP | GO:0050810 | regulation of steroid biosynthetic process | 2/32 | 76/18614 | 0.007538 | 0.01684 | 0.005092 | IFNG/IL1A | 2 |
| BP | GO:1903036 | positive regulation of response to wounding | 2/32 | 76/18614 | 0.007538 | 0.01684 | 0.005092 | NFE2L2/SERPINE1 | 2 |
| BP | GO:0006909 | phagocytosis | 3/32 | 236/18614 | 0.007611 | 0.016988 | 0.005136 | IL1B/CCL2/IFNG | 3 |
| BP | GO:0050853 | B cell receptor signaling pathway | 2/32 | 77/18614 | 0.007731 | 0.017228 | 0.005209 | BCL2/MAPK1 | 2 |
| BP | GO:0072088 | nephron epithelium morphogenesis | 2/32 | 77/18614 | 0.007731 | 0.017228 | 0.005209 | BCL2/MYC | 2 |
| BP | GO:0030072 | peptide hormone secretion | 3/32 | 238/18614 | 0.007788 | 0.017342 | 0.005243 | IL1B/HIF1A/IFNG | 3 |
| BP | GO:0007160 | cell-matrix adhesion | 3/32 | 239/18614 | 0.007878 | 0.017528 | 0.0053 | BCL2/GSK3B/SERPINE1 | 3 |
| BP | GO:0008088 | axo-dendritic transport | 2/32 | 78/18614 | 0.007926 | 0.017619 | 0.005327 | HIF1A/APP | 2 |
| BP | GO:0050803 | regulation of synapse structure or activity | 3/32 | 240/18614 | 0.007969 | 0.0177 | 0.005352 | AKT1/IL10/APP | 3 |
| BP | GO:0050808 | synapse organization | 4/32 | 466/18614 | 0.008005 | 0.017766 | 0.005372 | AKT1/ERBB2/IL10/APP | 4 |
| BP | GO:0061333 | renal tubule morphogenesis | 2/32 | 79/18614 | 0.008123 | 0.017998 | 0.005442 | BCL2/MYC | 2 |
| BP | GO:2000736 | regulation of stem cell differentiation | 2/32 | 79/18614 | 0.008123 | 0.017998 | 0.005442 | GSK3B/NFE2L2 | 2 |
| BP | GO:0002790 | peptide secretion | 3/32 | 243/18614 | 0.008244 | 0.018251 | 0.005518 | IL1B/HIF1A/IFNG | 3 |
| BP | GO:0042440 | pigment metabolic process | 2/32 | 80/18614 | 0.008323 | 0.018335 | 0.005544 | BCL2/HMOX1 | 2 |
| BP | GO:0051966 | regulation of synaptic transmission, glutamatergic | 2/32 | 80/18614 | 0.008323 | 0.018335 | 0.005544 | PTGS2/CCL2 | 2 |
| BP | GO:0071230 | cellular response to amino acid stimulus | 2/32 | 80/18614 | 0.008323 | 0.018335 | 0.005544 | EGFR/MMP2 | 2 |
| BP | GO:0072028 | nephron morphogenesis | 2/32 | 80/18614 | 0.008323 | 0.018335 | 0.005544 | BCL2/MYC | 2 |
| BP | GO:0090049 | regulation of cell migration involved in sprouting angiogenesis | 2/32 | 80/18614 | 0.008323 | 0.018335 | 0.005544 | PTGS2/HMOX1 | 2 |
| BP | GO:2000142 | regulation of DNA-templated transcription initiation | 2/32 | 80/18614 | 0.008323 | 0.018335 | 0.005544 | TP53/ESR1 | 2 |
| BP | GO:0009205 | purine ribonucleoside triphosphate metabolic process | 3/32 | 244/18614 | 0.008337 | 0.018352 | 0.005549 | HIF1A/IFNG/APP | 3 |
| BP | GO:0031016 | pancreas development | 2/32 | 81/18614 | 0.008524 | 0.018734 | 0.005664 | AKT1/GSK3B | 2 |
| BP | GO:0044773 | mitotic DNA damage checkpoint signaling | 2/32 | 81/18614 | 0.008524 | 0.018734 | 0.005664 | TP53/CCND1 | 2 |
| BP | GO:0042593 | glucose homeostasis | 3/32 | 248/18614 | 0.008715 | 0.019104 | 0.005776 | AKT1/HIF1A/PPARG | 3 |
| BP | GO:0010921 | regulation of phosphatase activity | 2/32 | 82/18614 | 0.008728 | 0.019104 | 0.005776 | GSK3B/IFNG | 2 |
| BP | GO:0014032 | neural crest cell development | 2/32 | 82/18614 | 0.008728 | 0.019104 | 0.005776 | HIF1A/MAPK1 | 2 |
| BP | GO:0042509 | regulation of tyrosine phosphorylation of STAT protein | 2/32 | 82/18614 | 0.008728 | 0.019104 | 0.005776 | IFNG/CAV1 | 2 |
| BP | GO:1905897 | regulation of response to endoplasmic reticulum stress | 2/32 | 82/18614 | 0.008728 | 0.019104 | 0.005776 | NFE2L2/CAV1 | 2 |
| BP | GO:0009144 | purine nucleoside triphosphate metabolic process | 3/32 | 249/18614 | 0.008811 | 0.019255 | 0.005822 | HIF1A/IFNG/APP | 3 |
| BP | GO:0033500 | carbohydrate homeostasis | 3/32 | 249/18614 | 0.008811 | 0.019255 | 0.005822 | AKT1/HIF1A/PPARG | 3 |
| BP | GO:0001649 | osteoblast differentiation | 3/32 | 250/18614 | 0.008908 | 0.01943 | 0.005875 | AKT1/PPARG/GSK3B | 3 |
| BP | GO:0016485 | protein processing | 3/32 | 250/18614 | 0.008908 | 0.01943 | 0.005875 | CASP3/CASP9/SERPINE1 | 3 |
| BP | GO:0007492 | endoderm development | 2/32 | 83/18614 | 0.008934 | 0.01943 | 0.005875 | MMP9/MMP2 | 2 |
| BP | GO:0030433 | ubiquitin-dependent ERAD pathway | 2/32 | 83/18614 | 0.008934 | 0.01943 | 0.005875 | NFE2L2/CAV1 | 2 |
| BP | GO:0050871 | positive regulation of B cell activation | 2/32 | 83/18614 | 0.008934 | 0.01943 | 0.005875 | BCL2/IL10 | 2 |
| BP | GO:0070897 | transcription preinitiation complex assembly | 2/32 | 83/18614 | 0.008934 | 0.01943 | 0.005875 | TP53/ESR1 | 2 |
| BP | GO:0009199 | ribonucleoside triphosphate metabolic process | 3/32 | 251/18614 | 0.009005 | 0.019569 | 0.005917 | HIF1A/IFNG/APP | 3 |
| BP | GO:0045926 | negative regulation of growth | 3/32 | 252/18614 | 0.009103 | 0.01975 | 0.005972 | TP53/BCL2/HIF1A | 3 |
| BP | GO:0097305 | response to alcohol | 3/32 | 252/18614 | 0.009103 | 0.01975 | 0.005972 | AKT1/FOS/CCND1 | 3 |
| BP | GO:0090257 | regulation of muscle system process | 3/32 | 254/18614 | 0.009301 | 0.020163 | 0.006096 | PTGS2/PPARG/CAV1 | 3 |
| BP | GO:0031397 | negative regulation of protein ubiquitination | 2/32 | 85/18614 | 0.009352 | 0.020226 | 0.006116 | AKT1/CAV1 | 2 |
| BP | GO:0044774 | mitotic DNA integrity checkpoint signaling | 2/32 | 85/18614 | 0.009352 | 0.020226 | 0.006116 | TP53/CCND1 | 2 |
| BP | GO:0098586 | cellular response to virus | 2/32 | 85/18614 | 0.009352 | 0.020226 | 0.006116 | HIF1A/IFNG | 2 |
| BP | GO:0007260 | tyrosine phosphorylation of STAT protein | 2/32 | 86/18614 | 0.009565 | 0.02062 | 0.006235 | IFNG/CAV1 | 2 |
| BP | GO:0021766 | hippocampus development | 2/32 | 86/18614 | 0.009565 | 0.02062 | 0.006235 | CASP3/GSK3B | 2 |
| BP | GO:0034103 | regulation of tissue remodeling | 2/32 | 86/18614 | 0.009565 | 0.02062 | 0.006235 | TP53/PPARG | 2 |
| BP | GO:0045069 | regulation of viral genome replication | 2/32 | 86/18614 | 0.009565 | 0.02062 | 0.006235 | BCL2/CXCL8 | 2 |
| BP | GO:0090066 | regulation of anatomical structure size | 4/32 | 492/18614 | 0.009648 | 0.020784 | 0.006284 | PTGS2/GSK3B/MMP2/CAV1 | 4 |
| BP | GO:0001960 | negative regulation of cytokine-mediated signaling pathway | 2/32 | 87/18614 | 0.009779 | 0.021032 | 0.006359 | PPARG/CAV1 | 2 |
| BP | GO:0048678 | response to axon injury | 2/32 | 87/18614 | 0.009779 | 0.021032 | 0.006359 | BCL2/MMP2 | 2 |
| BP | GO:0015833 | peptide transport | 3/32 | 259/18614 | 0.009806 | 0.021074 | 0.006372 | IL1B/HIF1A/IFNG | 3 |
| BP | GO:0048285 | organelle fission | 4/32 | 497/18614 | 0.009988 | 0.021447 | 0.006485 | IL1B/PPARG/EGF/IL1A | 4 |
| BP | GO:0032413 | negative regulation of ion transmembrane transporter activity | 2/32 | 88/18614 | 0.009996 | 0.021448 | 0.006485 | MMP9/CAV1 | 2 |
| BP | GO:1903522 | regulation of blood circulation | 3/32 | 261/18614 | 0.010013 | 0.021466 | 0.006491 | PTGS2/MMP2/CAV1 | 3 |
| BP | GO:0051591 | response to cAMP | 2/32 | 89/18614 | 0.010215 | 0.021846 | 0.006605 | FOS/APP | 2 |
| BP | GO:0060191 | regulation of lipase activity | 2/32 | 89/18614 | 0.010215 | 0.021846 | 0.006605 | ESR1/EGFR | 2 |
| BP | GO:0071229 | cellular response to acid chemical | 2/32 | 89/18614 | 0.010215 | 0.021846 | 0.006605 | EGFR/MMP2 | 2 |
| BP | GO:0071824 | protein-DNA complex subunit organization | 3/32 | 263/18614 | 0.010222 | 0.021846 | 0.006605 | TP53/ESR1/MYC | 3 |
| BP | GO:0003018 | vascular process in circulatory system | 3/32 | 265/18614 | 0.010434 | 0.022216 | 0.006717 | PTGS2/MMP2/CAV1 | 3 |
| BP | GO:0033555 | multicellular organismal response to stress | 2/32 | 90/18614 | 0.010436 | 0.022216 | 0.006717 | AKT1/BCL2 | 2 |
| BP | GO:0060395 | SMAD protein signal transduction | 2/32 | 90/18614 | 0.010436 | 0.022216 | 0.006717 | FOS/PPARG | 2 |
| BP | GO:0106027 | neuron projection organization | 2/32 | 90/18614 | 0.010436 | 0.022216 | 0.006717 | GSK3B/APP | 2 |
| BP | GO:1903351 | cellular response to dopamine | 2/32 | 90/18614 | 0.010436 | 0.022216 | 0.006717 | GSK3B/MAPK1 | 2 |
| BP | GO:1903350 | response to dopamine | 2/32 | 91/18614 | 0.010659 | 0.022673 | 0.006855 | GSK3B/MAPK1 | 2 |
| BP | GO:0009141 | nucleoside triphosphate metabolic process | 3/32 | 269/18614 | 0.010865 | 0.023044 | 0.006967 | HIF1A/IFNG/APP | 3 |
| BP | GO:0090596 | sensory organ morphogenesis | 3/32 | 269/18614 | 0.010865 | 0.023044 | 0.006967 | BCL2/HIF1A/MAPK1 | 3 |
| BP | GO:0000422 | autophagy of mitochondrion | 2/32 | 92/18614 | 0.010885 | 0.023044 | 0.006967 | TP53/HIF1A | 2 |
| BP | GO:0001656 | metanephros development | 2/32 | 92/18614 | 0.010885 | 0.023044 | 0.006967 | BCL2/MYC | 2 |
| BP | GO:0060761 | negative regulation of response to cytokine stimulus | 2/32 | 92/18614 | 0.010885 | 0.023044 | 0.006967 | PPARG/CAV1 | 2 |
| BP | GO:0061726 | mitochondrion disassembly | 2/32 | 92/18614 | 0.010885 | 0.023044 | 0.006967 | TP53/HIF1A | 2 |
| BP | GO:0030641 | regulation of cellular pH | 2/32 | 93/18614 | 0.011112 | 0.02347 | 0.007096 | BCL2/MAPK1 | 2 |
| BP | GO:0050886 | endocrine process | 2/32 | 93/18614 | 0.011112 | 0.02347 | 0.007096 | IL1B/PPARG | 2 |
| BP | GO:0051781 | positive regulation of cell division | 2/32 | 93/18614 | 0.011112 | 0.02347 | 0.007096 | IL1B/IL1A | 2 |
| BP | GO:0043547 | positive regulation of GTPase activity | 3/32 | 272/18614 | 0.011195 | 0.023627 | 0.007144 | GSK3B/ERBB2/CCL2 | 3 |
| BP | GO:0002753 | cytosolic pattern recognition receptor signaling pathway | 2/32 | 95/18614 | 0.011573 | 0.024387 | 0.007374 | RELA/CAV1 | 2 |
| BP | GO:0014033 | neural crest cell differentiation | 2/32 | 95/18614 | 0.011573 | 0.024387 | 0.007374 | HIF1A/MAPK1 | 2 |
| BP | GO:0044264 | cellular polysaccharide metabolic process | 2/32 | 96/18614 | 0.011807 | 0.024802 | 0.007499 | AKT1/GSK3B | 2 |
| BP | GO:0045807 | positive regulation of endocytosis | 2/32 | 96/18614 | 0.011807 | 0.024802 | 0.007499 | EGF/SERPINE1 | 2 |
| BP | GO:0072080 | nephron tubule development | 2/32 | 96/18614 | 0.011807 | 0.024802 | 0.007499 | BCL2/MYC | 2 |
| BP | GO:1903510 | mucopolysaccharide metabolic process | 2/32 | 96/18614 | 0.011807 | 0.024802 | 0.007499 | IL1B/EGF | 2 |
| BP | GO:0006260 | DNA replication | 3/32 | 278/18614 | 0.011872 | 0.024921 | 0.007535 | TP53/EGFR/EGF | 3 |
| BP | GO:0001657 | ureteric bud development | 2/32 | 97/18614 | 0.012042 | 0.025219 | 0.007625 | BCL2/MYC | 2 |
| BP | GO:0014020 | primary neural tube formation | 2/32 | 97/18614 | 0.012042 | 0.025219 | 0.007625 | CASP3/HIF1A | 2 |
| BP | GO:0060993 | kidney morphogenesis | 2/32 | 97/18614 | 0.012042 | 0.025219 | 0.007625 | BCL2/MYC | 2 |
| BP | GO:0051962 | positive regulation of nervous system development | 3/32 | 280/18614 | 0.012103 | 0.025328 | 0.007658 | IL1B/HIF1A/IFNG | 3 |
| BP | GO:0002275 | myeloid cell activation involved in immune response | 2/32 | 98/18614 | 0.01228 | 0.025598 | 0.00774 | IFNG/HMOX1 | 2 |
| BP | GO:0034620 | cellular response to unfolded protein | 2/32 | 98/18614 | 0.01228 | 0.025598 | 0.00774 | CCND1/NFE2L2 | 2 |
| BP | GO:0072163 | mesonephric epithelium development | 2/32 | 98/18614 | 0.01228 | 0.025598 | 0.00774 | BCL2/MYC | 2 |
| BP | GO:0072164 | mesonephric tubule development | 2/32 | 98/18614 | 0.01228 | 0.025598 | 0.00774 | BCL2/MYC | 2 |
| BP | GO:0097306 | cellular response to alcohol | 2/32 | 98/18614 | 0.01228 | 0.025598 | 0.00774 | AKT1/FOS | 2 |
| BP | GO:0019218 | regulation of steroid metabolic process | 2/32 | 99/18614 | 0.01252 | 0.026058 | 0.007879 | IFNG/IL1A | 2 |
| BP | GO:0098869 | cellular oxidant detoxification | 2/32 | 99/18614 | 0.01252 | 0.026058 | 0.007879 | PTGS2/NFE2L2 | 2 |
| BP | GO:0072659 | protein localization to plasma membrane | 3/32 | 285/18614 | 0.012692 | 0.026397 | 0.007981 | AKT1/EGFR/IFNG | 3 |
| BP | GO:0061326 | renal tubule development | 2/32 | 100/18614 | 0.012762 | 0.026521 | 0.008019 | BCL2/MYC | 2 |
| BP | GO:0140014 | mitotic nuclear division | 3/32 | 286/18614 | 0.012812 | 0.026605 | 0.008044 | IL1B/EGF/IL1A | 3 |
| BP | GO:0010389 | regulation of G2/M transition of mitotic cell cycle | 2/32 | 101/18614 | 0.013006 | 0.026987 | 0.00816 | CCND1/APP | 2 |
| BP | GO:0001823 | mesonephros development | 2/32 | 102/18614 | 0.013252 | 0.027434 | 0.008295 | BCL2/MYC | 2 |
| BP | GO:0002532 | production of molecular mediator involved in inflammatory response | 2/32 | 102/18614 | 0.013252 | 0.027434 | 0.008295 | HIF1A/SERPINE1 | 2 |
| BP | GO:0030316 | osteoclast differentiation | 2/32 | 102/18614 | 0.013252 | 0.027434 | 0.008295 | FOS/IFNG | 2 |
| BP | GO:0044262 | cellular carbohydrate metabolic process | 3/32 | 290/18614 | 0.013297 | 0.027508 | 0.008317 | AKT1/TP53/GSK3B | 3 |
| BP | GO:0006885 | regulation of pH | 2/32 | 103/18614 | 0.0135 | 0.027884 | 0.008431 | BCL2/MAPK1 | 2 |
| BP | GO:0010906 | regulation of glucose metabolic process | 2/32 | 103/18614 | 0.0135 | 0.027884 | 0.008431 | AKT1/TP53 | 2 |
| BP | GO:0019932 | second-messenger-mediated signaling | 3/32 | 292/18614 | 0.013544 | 0.027954 | 0.008452 | EGFR/GSK3B/CXCL8 | 3 |
| BP | GO:0032204 | regulation of telomere maintenance | 2/32 | 104/18614 | 0.01375 | 0.028357 | 0.008574 | MYC/MAPK1 | 2 |
| BP | GO:0001841 | neural tube formation | 2/32 | 105/18614 | 0.014002 | 0.028833 | 0.008718 | CASP3/HIF1A | 2 |
| BP | GO:0042102 | positive regulation of T cell proliferation | 2/32 | 105/18614 | 0.014002 | 0.028833 | 0.008718 | IL1B/IL1A | 2 |
| BP | GO:0015850 | organic hydroxy compound transport | 3/32 | 296/18614 | 0.014045 | 0.0289 | 0.008738 | PPARG/EGF/CAV1 | 3 |
| BP | GO:0051146 | striated muscle cell differentiation | 3/32 | 297/18614 | 0.014172 | 0.029139 | 0.008811 | AKT1/CASP3/BCL2 | 3 |
| BP | GO:0032526 | response to retinoic acid | 2/32 | 107/18614 | 0.014512 | 0.029771 | 0.009001 | GSK3B/MMP2 | 2 |
| BP | GO:0062014 | negative regulation of small molecule metabolic process | 2/32 | 107/18614 | 0.014512 | 0.029771 | 0.009001 | AKT1/TP53 | 2 |
| BP | GO:0062207 | regulation of pattern recognition receptor signaling pathway | 2/32 | 107/18614 | 0.014512 | 0.029771 | 0.009001 | ESR1/CAV1 | 2 |
| BP | GO:0045089 | positive regulation of innate immune response | 3/32 | 300/18614 | 0.014557 | 0.02984 | 0.009022 | ESR1/RELA/CAV1 | 3 |
| BP | GO:0090092 | regulation of transmembrane receptor protein serine/threonine kinase signaling pathway | 3/32 | 301/18614 | 0.014686 | 0.030083 | 0.009096 | TP53/PPARG/CAV1 | 3 |
| BP | GO:0014013 | regulation of gliogenesis | 2/32 | 108/18614 | 0.01477 | 0.030232 | 0.009141 | IL1B/TP53 | 2 |
| BP | GO:0002429 | immune response-activating cell surface receptor signaling pathway | 3/32 | 302/18614 | 0.014817 | 0.030304 | 0.009163 | BCL2/RELA/MAPK1 | 3 |
| BP | GO:0006874 | intracellular calcium ion homeostasis | 3/32 | 303/18614 | 0.014948 | 0.030549 | 0.009237 | BCL2/APP/CAV1 | 3 |
| BP | GO:0007631 | feeding behavior | 2/32 | 109/18614 | 0.01503 | 0.030602 | 0.009253 | FOS/APP | 2 |
| BP | GO:0033209 | tumor necrosis factor-mediated signaling pathway | 2/32 | 109/18614 | 0.01503 | 0.030602 | 0.009253 | TP53/RELA | 2 |
| BP | GO:0036503 | ERAD pathway | 2/32 | 109/18614 | 0.01503 | 0.030602 | 0.009253 | NFE2L2/CAV1 | 2 |
| BP | GO:0045833 | negative regulation of lipid metabolic process | 2/32 | 109/18614 | 0.01503 | 0.030602 | 0.009253 | AKT1/IL1B | 2 |
| BP | GO:1902106 | negative regulation of leukocyte differentiation | 2/32 | 109/18614 | 0.01503 | 0.030602 | 0.009253 | MYC/ERBB2 | 2 |
| BP | GO:0031341 | regulation of cell killing | 2/32 | 110/18614 | 0.015292 | 0.031112 | 0.009407 | IFNG/ICAM1 | 2 |
| BP | GO:0006367 | transcription initiation at RNA polymerase II promoter | 2/32 | 111/18614 | 0.015556 | 0.031626 | 0.009562 | TP53/ESR1 | 2 |
| BP | GO:0014066 | regulation of phosphatidylinositol 3-kinase signaling | 2/32 | 112/18614 | 0.015822 | 0.032071 | 0.009697 | EGFR/EGF | 2 |
| BP | GO:0032611 | interleukin-1 beta production | 2/32 | 112/18614 | 0.015822 | 0.032071 | 0.009697 | IFNG/APP | 2 |
| BP | GO:0032651 | regulation of interleukin-1 beta production | 2/32 | 112/18614 | 0.015822 | 0.032071 | 0.009697 | IFNG/APP | 2 |
| BP | GO:0035249 | synaptic transmission, glutamatergic | 2/32 | 112/18614 | 0.015822 | 0.032071 | 0.009697 | PTGS2/CCL2 | 2 |
| BP | GO:0006939 | smooth muscle contraction | 2/32 | 113/18614 | 0.01609 | 0.032541 | 0.009839 | PTGS2/CAV1 | 2 |
| BP | GO:0032414 | positive regulation of ion transmembrane transporter activity | 2/32 | 113/18614 | 0.01609 | 0.032541 | 0.009839 | CCL2/IFNG | 2 |
| BP | GO:0044344 | cellular response to fibroblast growth factor stimulus | 2/32 | 113/18614 | 0.01609 | 0.032541 | 0.009839 | CCL2/CXCL8 | 2 |
| BP | GO:0032410 | negative regulation of transporter activity | 2/32 | 114/18614 | 0.01636 | 0.033037 | 0.009989 | MMP9/CAV1 | 2 |
| BP | GO:0033559 | unsaturated fatty acid metabolic process | 2/32 | 114/18614 | 0.01636 | 0.033037 | 0.009989 | IL1B/PTGS2 | 2 |
| BP | GO:0042742 | defense response to bacterium | 3/32 | 314/18614 | 0.016431 | 0.033156 | 0.010025 | IL1B/IL10/SERPINE1 | 3 |
| BP | GO:0021761 | limbic system development | 2/32 | 115/18614 | 0.016632 | 0.033363 | 0.010088 | CASP3/GSK3B | 2 |
| BP | GO:0022037 | metencephalon development | 2/32 | 115/18614 | 0.016632 | 0.033363 | 0.010088 | TP53/BCL2 | 2 |
| BP | GO:0048259 | regulation of receptor-mediated endocytosis | 2/32 | 115/18614 | 0.016632 | 0.033363 | 0.010088 | EGF/SERPINE1 | 2 |
| BP | GO:1903707 | negative regulation of hemopoiesis | 2/32 | 115/18614 | 0.016632 | 0.033363 | 0.010088 | MYC/ERBB2 | 2 |
| BP | GO:1990748 | cellular detoxification | 2/32 | 115/18614 | 0.016632 | 0.033363 | 0.010088 | PTGS2/NFE2L2 | 2 |
| BP | GO:0006476 | protein deacetylation | 2/32 | 116/18614 | 0.016906 | 0.033363 | 0.010088 | TP53/IFNG | 2 |
| BP | GO:0072009 | nephron epithelium development | 2/32 | 116/18614 | 0.016906 | 0.033363 | 0.010088 | BCL2/MYC | 2 |
| BP | GO:0001765 | membrane raft assembly | 1/32 | 10/18614 | 0.017063 | 0.033363 | 0.010088 | CAV1 | 1 |
| BP | GO:0002291 | T cell activation via T cell receptor contact with antigen bound to MHC molecule on antigen presenting cell | 1/32 | 10/18614 | 0.017063 | 0.033363 | 0.010088 | ICAM1 | 1 |
| BP | GO:0002361 | CD4-positive, CD25-positive, alpha-beta regulatory T cell differentiation | 1/32 | 10/18614 | 0.017063 | 0.033363 | 0.010088 | IFNG | 1 |
| BP | GO:0002676 | regulation of chronic inflammatory response | 1/32 | 10/18614 | 0.017063 | 0.033363 | 0.010088 | IL10 | 1 |
| BP | GO:0009629 | response to gravity | 1/32 | 10/18614 | 0.017063 | 0.033363 | 0.010088 | FOS | 1 |
| BP | GO:0010887 | negative regulation of cholesterol storage | 1/32 | 10/18614 | 0.017063 | 0.033363 | 0.010088 | PPARG | 1 |
| BP | GO:0010918 | positive regulation of mitochondrial membrane potential | 1/32 | 10/18614 | 0.017063 | 0.033363 | 0.010088 | AKT1 | 1 |
| BP | GO:0014012 | peripheral nervous system axon regeneration | 1/32 | 10/18614 | 0.017063 | 0.033363 | 0.010088 | MMP2 | 1 |
| BP | GO:0015911 | long-chain fatty acid import across plasma membrane | 1/32 | 10/18614 | 0.017063 | 0.033363 | 0.010088 | AKT1 | 1 |
| BP | GO:0019062 | virion attachment to host cell | 1/32 | 10/18614 | 0.017063 | 0.033363 | 0.010088 | ICAM1 | 1 |
| BP | GO:0030388 | fructose 1,6-bisphosphate metabolic process | 1/32 | 10/18614 | 0.017063 | 0.033363 | 0.010088 | IFNG | 1 |
| BP | GO:0032070 | regulation of deoxyribonuclease activity | 1/32 | 10/18614 | 0.017063 | 0.033363 | 0.010088 | AKT1 | 1 |
| BP | GO:0033007 | negative regulation of mast cell activation involved in immune response | 1/32 | 10/18614 | 0.017063 | 0.033363 | 0.010088 | HMOX1 | 1 |
| BP | GO:0033085 | negative regulation of T cell differentiation in thymus | 1/32 | 10/18614 | 0.017063 | 0.033363 | 0.010088 | ERBB2 | 1 |
| BP | GO:0033197 | response to vitamin E | 1/32 | 10/18614 | 0.017063 | 0.033363 | 0.010088 | CCND1 | 1 |
| BP | GO:0033212 | iron import into cell | 1/32 | 10/18614 | 0.017063 | 0.033363 | 0.010088 | IFNG | 1 |
| BP | GO:0035747 | natural killer cell chemotaxis | 1/32 | 10/18614 | 0.017063 | 0.033363 | 0.010088 | CCL2 | 1 |
| BP | GO:0038130 | ERBB4 signaling pathway | 1/32 | 10/18614 | 0.017063 | 0.033363 | 0.010088 | ERBB2 | 1 |
| BP | GO:0042368 | vitamin D biosynthetic process | 1/32 | 10/18614 | 0.017063 | 0.033363 | 0.010088 | IFNG | 1 |
| BP | GO:0046886 | positive regulation of hormone biosynthetic process | 1/32 | 10/18614 | 0.017063 | 0.033363 | 0.010088 | HIF1A | 1 |
| BP | GO:0051974 | negative regulation of telomerase activity | 1/32 | 10/18614 | 0.017063 | 0.033363 | 0.010088 | TP53 | 1 |
| BP | GO:0060068 | vagina development | 1/32 | 10/18614 | 0.017063 | 0.033363 | 0.010088 | ESR1 | 1 |
| BP | GO:0060346 | bone trabecula formation | 1/32 | 10/18614 | 0.017063 | 0.033363 | 0.010088 | MMP2 | 1 |
| BP | GO:0060525 | prostate glandular acinus development | 1/32 | 10/18614 | 0.017063 | 0.033363 | 0.010088 | ESR1 | 1 |
| BP | GO:0070099 | regulation of chemokine-mediated signaling pathway | 1/32 | 10/18614 | 0.017063 | 0.033363 | 0.010088 | HIF1A | 1 |
| BP | GO:0070391 | response to lipoteichoic acid | 1/32 | 10/18614 | 0.017063 | 0.033363 | 0.010088 | RELA | 1 |
| BP | GO:0070874 | negative regulation of glycogen metabolic process | 1/32 | 10/18614 | 0.017063 | 0.033363 | 0.010088 | GSK3B | 1 |
| BP | GO:0071223 | cellular response to lipoteichoic acid | 1/32 | 10/18614 | 0.017063 | 0.033363 | 0.010088 | RELA | 1 |
| BP | GO:0071374 | cellular response to parathyroid hormone stimulus | 1/32 | 10/18614 | 0.017063 | 0.033363 | 0.010088 | FOS | 1 |
| BP | GO:0072203 | cell proliferation involved in metanephros development | 1/32 | 10/18614 | 0.017063 | 0.033363 | 0.010088 | MYC | 1 |
| BP | GO:0098903 | regulation of membrane repolarization during action potential | 1/32 | 10/18614 | 0.017063 | 0.033363 | 0.010088 | CAV1 | 1 |
| BP | GO:1904779 | regulation of protein localization to centrosome | 1/32 | 10/18614 | 0.017063 | 0.033363 | 0.010088 | GSK3B | 1 |
| BP | GO:1905461 | positive regulation of vascular associated smooth muscle cell apoptotic process | 1/32 | 10/18614 | 0.017063 | 0.033363 | 0.010088 | PPARG | 1 |
| BP | GO:2000121 | regulation of removal of superoxide radicals | 1/32 | 10/18614 | 0.017063 | 0.033363 | 0.010088 | NFE2L2 | 1 |
| BP | GO:2000425 | regulation of apoptotic cell clearance | 1/32 | 10/18614 | 0.017063 | 0.033363 | 0.010088 | CCL2 | 1 |
| BP | GO:2001054 | negative regulation of mesenchymal cell apoptotic process | 1/32 | 10/18614 | 0.017063 | 0.033363 | 0.010088 | HIF1A | 1 |
| BP | GO:0030203 | glycosaminoglycan metabolic process | 2/32 | 117/18614 | 0.017182 | 0.033571 | 0.010151 | IL1B/EGF | 2 |
| BP | GO:0032609 | type II interferon production | 2/32 | 118/18614 | 0.017459 | 0.034016 | 0.010285 | IL1B/IL10 | 2 |
| BP | GO:0032649 | regulation of type II interferon production | 2/32 | 118/18614 | 0.017459 | 0.034016 | 0.010285 | IL1B/IL10 | 2 |
| BP | GO:0035967 | cellular response to topologically incorrect protein | 2/32 | 118/18614 | 0.017459 | 0.034016 | 0.010285 | CCND1/NFE2L2 | 2 |
| BP | GO:0051897 | positive regulation of protein kinase B signaling | 2/32 | 118/18614 | 0.017459 | 0.034016 | 0.010285 | EGFR/EGF | 2 |
| BP | GO:0045446 | endothelial cell differentiation | 2/32 | 119/18614 | 0.017739 | 0.034511 | 0.010435 | IL1B/ICAM1 | 2 |
| BP | GO:0046632 | alpha-beta T cell differentiation | 2/32 | 119/18614 | 0.017739 | 0.034511 | 0.010435 | BCL2/IFNG | 2 |
| BP | GO:0006304 | DNA modification | 2/32 | 120/18614 | 0.01802 | 0.035008 | 0.010585 | FOS/MYC | 2 |
| BP | GO:0071774 | response to fibroblast growth factor | 2/32 | 120/18614 | 0.01802 | 0.035008 | 0.010585 | CCL2/CXCL8 | 2 |
| BP | GO:0002833 | positive regulation of response to biotic stimulus | 3/32 | 327/18614 | 0.018288 | 0.035477 | 0.010727 | ESR1/RELA/CAV1 | 3 |
| BP | GO:0055074 | calcium ion homeostasis | 3/32 | 327/18614 | 0.018288 | 0.035477 | 0.010727 | BCL2/APP/CAV1 | 3 |
| BP | GO:0030282 | bone mineralization | 2/32 | 121/18614 | 0.018304 | 0.035483 | 0.010728 | PTGS2/HIF1A | 2 |
| BP | GO:0002440 | production of molecular mediator of immune response | 3/32 | 328/18614 | 0.018436 | 0.035567 | 0.010754 | IL1B/IL10/HMOX1 | 3 |
| BP | GO:0002768 | immune response-regulating cell surface receptor signaling pathway | 3/32 | 329/18614 | 0.018584 | 0.035567 | 0.010754 | BCL2/RELA/MAPK1 | 3 |
| BP | GO:0002698 | negative regulation of immune effector process | 2/32 | 122/18614 | 0.018589 | 0.035567 | 0.010754 | IL10/HMOX1 | 2 |
| BP | GO:0001547 | antral ovarian follicle growth | 1/32 | 11/18614 | 0.018754 | 0.035567 | 0.010754 | ESR1 | 1 |
| BP | GO:0007406 | negative regulation of neuroblast proliferation | 1/32 | 11/18614 | 0.018754 | 0.035567 | 0.010754 | TP53 | 1 |
| BP | GO:0014745 | negative regulation of muscle adaptation | 1/32 | 11/18614 | 0.018754 | 0.035567 | 0.010754 | PPARG | 1 |
| BP | GO:0019065 | receptor-mediated endocytosis of virus by host cell | 1/32 | 11/18614 | 0.018754 | 0.035567 | 0.010754 | CAV1 | 1 |
| BP | GO:0021548 | pons development | 1/32 | 11/18614 | 0.018754 | 0.035567 | 0.010754 | BCL2 | 1 |
| BP | GO:0021936 | regulation of cerebellar granule cell precursor proliferation | 1/32 | 11/18614 | 0.018754 | 0.035567 | 0.010754 | EGF | 1 |
| BP | GO:0033234 | negative regulation of protein sumoylation | 1/32 | 11/18614 | 0.018754 | 0.035567 | 0.010754 | RELA | 1 |
| BP | GO:0034115 | negative regulation of heterotypic cell-cell adhesion | 1/32 | 11/18614 | 0.018754 | 0.035567 | 0.010754 | IL10 | 1 |
| BP | GO:0034350 | regulation of glial cell apoptotic process | 1/32 | 11/18614 | 0.018754 | 0.035567 | 0.010754 | CCL2 | 1 |
| BP | GO:0035821 | modulation of process of another organism | 1/32 | 11/18614 | 0.018754 | 0.035567 | 0.010754 | IFNG | 1 |
| BP | GO:0042167 | heme catabolic process | 1/32 | 11/18614 | 0.018754 | 0.035567 | 0.010754 | HMOX1 | 1 |
| BP | GO:0044857 | plasma membrane raft organization | 1/32 | 11/18614 | 0.018754 | 0.035567 | 0.010754 | CAV1 | 1 |
| BP | GO:0046149 | pigment catabolic process | 1/32 | 11/18614 | 0.018754 | 0.035567 | 0.010754 | HMOX1 | 1 |
| BP | GO:0048742 | regulation of skeletal muscle fiber development | 1/32 | 11/18614 | 0.018754 | 0.035567 | 0.010754 | BCL2 | 1 |
| BP | GO:0060020 | Bergmann glial cell differentiation | 1/32 | 11/18614 | 0.018754 | 0.035567 | 0.010754 | MAPK1 | 1 |
| BP | GO:0060439 | trachea morphogenesis | 1/32 | 11/18614 | 0.018754 | 0.035567 | 0.010754 | MAPK1 | 1 |
| BP | GO:0060736 | prostate gland growth | 1/32 | 11/18614 | 0.018754 | 0.035567 | 0.010754 | ESR1 | 1 |
| BP | GO:0070587 | regulation of cell-cell adhesion involved in gastrulation | 1/32 | 11/18614 | 0.018754 | 0.035567 | 0.010754 | IL10 | 1 |
| BP | GO:0070673 | response to interleukin-18 | 1/32 | 11/18614 | 0.018754 | 0.035567 | 0.010754 | AKT1 | 1 |
| BP | GO:0071609 | chemokine (C-C motif) ligand 5 production | 1/32 | 11/18614 | 0.018754 | 0.035567 | 0.010754 | IL10 | 1 |
| BP | GO:0071649 | regulation of chemokine (C-C motif) ligand 5 production | 1/32 | 11/18614 | 0.018754 | 0.035567 | 0.010754 | IL10 | 1 |
| BP | GO:0090184 | positive regulation of kidney development | 1/32 | 11/18614 | 0.018754 | 0.035567 | 0.010754 | MYC | 1 |
| BP | GO:1900222 | negative regulation of amyloid-beta clearance | 1/32 | 11/18614 | 0.018754 | 0.035567 | 0.010754 | IFNG | 1 |
| BP | GO:1901844 | regulation of cell communication by electrical coupling involved in cardiac conduction | 1/32 | 11/18614 | 0.018754 | 0.035567 | 0.010754 | CAV1 | 1 |
| BP | GO:1903564 | regulation of protein localization to cilium | 1/32 | 11/18614 | 0.018754 | 0.035567 | 0.010754 | GSK3B | 1 |
| BP | GO:1990440 | positive regulation of transcription from RNA polymerase II promoter in response to endoplasmic reticulum stress | 1/32 | 11/18614 | 0.018754 | 0.035567 | 0.010754 | TP53 | 1 |
| BP | GO:1990535 | neuron projection maintenance | 1/32 | 11/18614 | 0.018754 | 0.035567 | 0.010754 | APP | 1 |
| BP | GO:2000672 | negative regulation of motor neuron apoptotic process | 1/32 | 11/18614 | 0.018754 | 0.035567 | 0.010754 | BCL2 | 1 |
| BP | GO:0000077 | DNA damage checkpoint signaling | 2/32 | 123/18614 | 0.018876 | 0.035774 | 0.010817 | TP53/CCND1 | 2 |
| BP | GO:0051604 | protein maturation | 3/32 | 332/18614 | 0.019032 | 0.036045 | 0.010898 | CASP3/CASP9/SERPINE1 | 3 |
| BP | GO:0032411 | positive regulation of transporter activity | 2/32 | 124/18614 | 0.019165 | 0.036246 | 0.010959 | CCL2/IFNG | 2 |
| BP | GO:0097237 | cellular response to toxic substance | 2/32 | 124/18614 | 0.019165 | 0.036246 | 0.010959 | PTGS2/NFE2L2 | 2 |
| BP | GO:0006690 | icosanoid metabolic process | 2/32 | 125/18614 | 0.019456 | 0.036771 | 0.011118 | IL1B/PTGS2 | 2 |
| BP | GO:0001838 | embryonic epithelial tube formation | 2/32 | 126/18614 | 0.019749 | 0.03722 | 0.011254 | CASP3/HIF1A | 2 |
| BP | GO:0003206 | cardiac chamber morphogenesis | 2/32 | 126/18614 | 0.019749 | 0.03722 | 0.011254 | TP53/HIF1A | 2 |
| BP | GO:0006022 | aminoglycan metabolic process | 2/32 | 126/18614 | 0.019749 | 0.03722 | 0.011254 | IL1B/EGF | 2 |
| BP | GO:0045471 | response to ethanol | 2/32 | 126/18614 | 0.019749 | 0.03722 | 0.011254 | FOS/CCND1 | 2 |
| BP | GO:0001704 | formation of primary germ layer | 2/32 | 127/18614 | 0.020044 | 0.037559 | 0.011356 | MMP9/MMP2 | 2 |
| BP | GO:0035303 | regulation of dephosphorylation | 2/32 | 127/18614 | 0.020044 | 0.037559 | 0.011356 | GSK3B/IFNG | 2 |
| BP | GO:0035601 | protein deacylation | 2/32 | 127/18614 | 0.020044 | 0.037559 | 0.011356 | TP53/IFNG | 2 |
| BP | GO:0001553 | luteinization | 1/32 | 12/18614 | 0.020442 | 0.037559 | 0.011356 | MMP2 | 1 |
| BP | GO:0001967 | suckling behavior | 1/32 | 12/18614 | 0.020442 | 0.037559 | 0.011356 | APP | 1 |
| BP | GO:0002069 | columnar/cuboidal epithelial cell maturation | 1/32 | 12/18614 | 0.020442 | 0.037559 | 0.011356 | HIF1A | 1 |
| BP | GO:0002887 | negative regulation of myeloid leukocyte mediated immunity | 1/32 | 12/18614 | 0.020442 | 0.037559 | 0.011356 | HMOX1 | 1 |
| BP | GO:0010749 | regulation of nitric oxide mediated signal transduction | 1/32 | 12/18614 | 0.020442 | 0.037559 | 0.011356 | EGFR | 1 |
| BP | GO:0014854 | response to inactivity | 1/32 | 12/18614 | 0.020442 | 0.037559 | 0.011356 | IL10 | 1 |
| BP | GO:0030656 | regulation of vitamin metabolic process | 1/32 | 12/18614 | 0.020442 | 0.037559 | 0.011356 | IFNG | 1 |
| BP | GO:0031953 | negative regulation of protein autophosphorylation | 1/32 | 12/18614 | 0.020442 | 0.037559 | 0.011356 | CAV1 | 1 |
| BP | GO:0033327 | Leydig cell differentiation | 1/32 | 12/18614 | 0.020442 | 0.037559 | 0.011356 | CCND1 | 1 |
| BP | GO:0033629 | negative regulation of cell adhesion mediated by integrin | 1/32 | 12/18614 | 0.020442 | 0.037559 | 0.011356 | SERPINE1 | 1 |
| BP | GO:0034454 | microtubule anchoring at centrosome | 1/32 | 12/18614 | 0.020442 | 0.037559 | 0.011356 | GSK3B | 1 |
| BP | GO:0036295 | cellular response to increased oxygen levels | 1/32 | 12/18614 | 0.020442 | 0.037559 | 0.011356 | CAV1 | 1 |
| BP | GO:0044650 | adhesion of symbiont to host cell | 1/32 | 12/18614 | 0.020442 | 0.037559 | 0.011356 | ICAM1 | 1 |
| BP | GO:0048548 | regulation of pinocytosis | 1/32 | 12/18614 | 0.020442 | 0.037559 | 0.011356 | CAV1 | 1 |
| BP | GO:0051001 | negative regulation of nitric-oxide synthase activity | 1/32 | 12/18614 | 0.020442 | 0.037559 | 0.011356 | CAV1 | 1 |
| BP | GO:0051095 | regulation of helicase activity | 1/32 | 12/18614 | 0.020442 | 0.037559 | 0.011356 | TP53 | 1 |
| BP | GO:0051549 | positive regulation of keratinocyte migration | 1/32 | 12/18614 | 0.020442 | 0.037559 | 0.011356 | MMP9 | 1 |
| BP | GO:0060394 | negative regulation of pathway-restricted SMAD protein phosphorylation | 1/32 | 12/18614 | 0.020442 | 0.037559 | 0.011356 | PPARG | 1 |
| BP | GO:0060442 | branching involved in prostate gland morphogenesis | 1/32 | 12/18614 | 0.020442 | 0.037559 | 0.011356 | ESR1 | 1 |
| BP | GO:0061517 | macrophage proliferation | 1/32 | 12/18614 | 0.020442 | 0.037559 | 0.011356 | MAPK1 | 1 |
| BP | GO:0070586 | cell-cell adhesion involved in gastrulation | 1/32 | 12/18614 | 0.020442 | 0.037559 | 0.011356 | IL10 | 1 |
| BP | GO:0072497 | mesenchymal stem cell differentiation | 1/32 | 12/18614 | 0.020442 | 0.037559 | 0.011356 | GSK3B | 1 |
| BP | GO:0072683 | T cell extravasation | 1/32 | 12/18614 | 0.020442 | 0.037559 | 0.011356 | CCL2 | 1 |
| BP | GO:0097048 | dendritic cell apoptotic process | 1/32 | 12/18614 | 0.020442 | 0.037559 | 0.011356 | BCL2 | 1 |
| BP | GO:0098911 | regulation of ventricular cardiac muscle cell action potential | 1/32 | 12/18614 | 0.020442 | 0.037559 | 0.011356 | CAV1 | 1 |
| BP | GO:0140052 | cellular response to oxidised low-density lipoprotein particle stimulus | 1/32 | 12/18614 | 0.020442 | 0.037559 | 0.011356 | AKT1 | 1 |
| BP | GO:1900272 | negative regulation of long-term synaptic potentiation | 1/32 | 12/18614 | 0.020442 | 0.037559 | 0.011356 | APP | 1 |
| BP | GO:1901030 | positive regulation of mitochondrial outer membrane permeabilization involved in apoptotic signaling pathway | 1/32 | 12/18614 | 0.020442 | 0.037559 | 0.011356 | GSK3B | 1 |
| BP | GO:1902946 | protein localization to early endosome | 1/32 | 12/18614 | 0.020442 | 0.037559 | 0.011356 | EGF | 1 |
| BP | GO:1904177 | regulation of adipose tissue development | 1/32 | 12/18614 | 0.020442 | 0.037559 | 0.011356 | PPARG | 1 |
| BP | GO:1904338 | regulation of dopaminergic neuron differentiation | 1/32 | 12/18614 | 0.020442 | 0.037559 | 0.011356 | GSK3B | 1 |
| BP | GO:1904672 | regulation of somatic stem cell population maintenance | 1/32 | 12/18614 | 0.020442 | 0.037559 | 0.011356 | MYC | 1 |
| BP | GO:1905668 | positive regulation of protein localization to endosome | 1/32 | 12/18614 | 0.020442 | 0.037559 | 0.011356 | EGF | 1 |
| BP | GO:2000668 | regulation of dendritic cell apoptotic process | 1/32 | 12/18614 | 0.020442 | 0.037559 | 0.011356 | BCL2 | 1 |
| BP | GO:0016311 | dephosphorylation | 3/32 | 342/18614 | 0.020571 | 0.037772 | 0.011421 | BCL2/GSK3B/IFNG | 3 |
| BP | GO:0051928 | positive regulation of calcium ion transport | 2/32 | 129/18614 | 0.020639 | 0.03787 | 0.01145 | CCL2/CAV1 | 2 |
| BP | GO:0030218 | erythrocyte differentiation | 2/32 | 130/18614 | 0.020939 | 0.038369 | 0.011601 | CASP3/HIF1A | 2 |
| BP | GO:0045621 | positive regulation of lymphocyte differentiation | 2/32 | 130/18614 | 0.020939 | 0.038369 | 0.011601 | IL10/IFNG | 2 |
| BP | GO:0098732 | macromolecule deacylation | 2/32 | 131/18614 | 0.021241 | 0.038897 | 0.011761 | TP53/IFNG | 2 |
| BP | GO:0031570 | DNA integrity checkpoint signaling | 2/32 | 132/18614 | 0.021545 | 0.039427 | 0.011921 | TP53/CCND1 | 2 |
| BP | GO:0042886 | amide transport | 3/32 | 349/18614 | 0.021689 | 0.039661 | 0.011992 | IL1B/HIF1A/IFNG | 3 |
| BP | GO:0007517 | muscle organ development | 3/32 | 351/18614 | 0.022014 | 0.039661 | 0.011992 | BCL2/FOS/CAV1 | 3 |
| BP | GO:0002357 | defense response to tumor cell | 1/32 | 13/18614 | 0.022127 | 0.039661 | 0.011992 | RELA | 1 |
| BP | GO:0006787 | porphyrin-containing compound catabolic process | 1/32 | 13/18614 | 0.022127 | 0.039661 | 0.011992 | HMOX1 | 1 |
| BP | GO:0010524 | positive regulation of calcium ion transport into cytosol | 1/32 | 13/18614 | 0.022127 | 0.039661 | 0.011992 | CAV1 | 1 |
| BP | GO:0010745 | negative regulation of macrophage derived foam cell differentiation | 1/32 | 13/18614 | 0.022127 | 0.039661 | 0.011992 | PPARG | 1 |
| BP | GO:0032494 | response to peptidoglycan | 1/32 | 13/18614 | 0.022127 | 0.039661 | 0.011992 | RELA | 1 |
| BP | GO:0032621 | interleukin-18 production | 1/32 | 13/18614 | 0.022127 | 0.039661 | 0.011992 | IL10 | 1 |
| BP | GO:0032661 | regulation of interleukin-18 production | 1/32 | 13/18614 | 0.022127 | 0.039661 | 0.011992 | IL10 | 1 |
| BP | GO:0033015 | tetrapyrrole catabolic process | 1/32 | 13/18614 | 0.022127 | 0.039661 | 0.011992 | HMOX1 | 1 |
| BP | GO:0034616 | response to laminar fluid shear stress | 1/32 | 13/18614 | 0.022127 | 0.039661 | 0.011992 | NFE2L2 | 1 |
| BP | GO:0042362 | fat-soluble vitamin biosynthetic process | 1/32 | 13/18614 | 0.022127 | 0.039661 | 0.011992 | IFNG | 1 |
| BP | GO:0043471 | regulation of cellular carbohydrate catabolic process | 1/32 | 13/18614 | 0.022127 | 0.039661 | 0.011992 | TP53 | 1 |
| BP | GO:0045741 | positive regulation of epidermal growth factor-activated receptor activity | 1/32 | 13/18614 | 0.022127 | 0.039661 | 0.011992 | EGF | 1 |
| BP | GO:0051481 | negative regulation of cytosolic calcium ion concentration | 1/32 | 13/18614 | 0.022127 | 0.039661 | 0.011992 | BCL2 | 1 |
| BP | GO:0051918 | negative regulation of fibrinolysis | 1/32 | 13/18614 | 0.022127 | 0.039661 | 0.011992 | SERPINE1 | 1 |
| BP | GO:0060670 | branching involved in labyrinthine layer morphogenesis | 1/32 | 13/18614 | 0.022127 | 0.039661 | 0.011992 | IL10 | 1 |
| BP | GO:0061430 | bone trabecula morphogenesis | 1/32 | 13/18614 | 0.022127 | 0.039661 | 0.011992 | MMP2 | 1 |
| BP | GO:0070234 | positive regulation of T cell apoptotic process | 1/32 | 13/18614 | 0.022127 | 0.039661 | 0.011992 | TP53 | 1 |
| BP | GO:0070486 | leukocyte aggregation | 1/32 | 13/18614 | 0.022127 | 0.039661 | 0.011992 | IL1B | 1 |
| BP | GO:0071107 | response to parathyroid hormone | 1/32 | 13/18614 | 0.022127 | 0.039661 | 0.011992 | FOS | 1 |
| BP | GO:0071236 | cellular response to antibiotic | 1/32 | 13/18614 | 0.022127 | 0.039661 | 0.011992 | TP53 | 1 |
| BP | GO:0071287 | cellular response to manganese ion | 1/32 | 13/18614 | 0.022127 | 0.039661 | 0.011992 | APP | 1 |
| BP | GO:0075509 | endocytosis involved in viral entry into host cell | 1/32 | 13/18614 | 0.022127 | 0.039661 | 0.011992 | CAV1 | 1 |
| BP | GO:0106049 | regulation of cellular response to osmotic stress | 1/32 | 13/18614 | 0.022127 | 0.039661 | 0.011992 | PTGS2 | 1 |
| BP | GO:1900121 | negative regulation of receptor binding | 1/32 | 13/18614 | 0.022127 | 0.039661 | 0.011992 | IL10 | 1 |
| BP | GO:1901524 | regulation of mitophagy | 1/32 | 13/18614 | 0.022127 | 0.039661 | 0.011992 | TP53 | 1 |
| BP | GO:1901857 | positive regulation of cellular respiration | 1/32 | 13/18614 | 0.022127 | 0.039661 | 0.011992 | IFNG | 1 |
| BP | GO:1903599 | positive regulation of autophagy of mitochondrion | 1/32 | 13/18614 | 0.022127 | 0.039661 | 0.011992 | HIF1A | 1 |
| BP | GO:1905666 | regulation of protein localization to endosome | 1/32 | 13/18614 | 0.022127 | 0.039661 | 0.011992 | EGF | 1 |
| BP | GO:2001053 | regulation of mesenchymal cell apoptotic process | 1/32 | 13/18614 | 0.022127 | 0.039661 | 0.011992 | HIF1A | 1 |
| BP | GO:0002702 | positive regulation of production of molecular mediator of immune response | 2/32 | 134/18614 | 0.022158 | 0.039692 | 0.012001 | IL1B/IL10 | 2 |
| BP | GO:0000086 | G2/M transition of mitotic cell cycle | 2/32 | 136/18614 | 0.022779 | 0.040776 | 0.012329 | CCND1/APP | 2 |
| BP | GO:0003158 | endothelium development | 2/32 | 137/18614 | 0.023092 | 0.041282 | 0.012482 | IL1B/ICAM1 | 2 |
| BP | GO:0031929 | TOR signaling | 2/32 | 137/18614 | 0.023092 | 0.041282 | 0.012482 | AKT1/HIF1A | 2 |
| BP | GO:0031589 | cell-substrate adhesion | 3/32 | 359/18614 | 0.023342 | 0.041702 | 0.012609 | BCL2/GSK3B/SERPINE1 | 3 |
| BP | GO:0046887 | positive regulation of hormone secretion | 2/32 | 138/18614 | 0.023406 | 0.04179 | 0.012635 | HIF1A/PPARG | 2 |
| BP | GO:0090090 | negative regulation of canonical Wnt signaling pathway | 2/32 | 139/18614 | 0.023723 | 0.041929 | 0.012678 | GSK3B/CAV1 | 2 |
| BP | GO:0006878 | intracellular copper ion homeostasis | 1/32 | 14/18614 | 0.023809 | 0.041929 | 0.012678 | APP | 1 |
| BP | GO:0016322 | neuron remodeling | 1/32 | 14/18614 | 0.023809 | 0.041929 | 0.012678 | APP | 1 |
| BP | GO:0033004 | negative regulation of mast cell activation | 1/32 | 14/18614 | 0.023809 | 0.041929 | 0.012678 | HMOX1 | 1 |
| BP | GO:0042541 | hemoglobin biosynthetic process | 1/32 | 14/18614 | 0.023809 | 0.041929 | 0.012678 | HIF1A | 1 |
| BP | GO:0043301 | negative regulation of leukocyte degranulation | 1/32 | 14/18614 | 0.023809 | 0.041929 | 0.012678 | HMOX1 | 1 |
| BP | GO:0048308 | organelle inheritance | 1/32 | 14/18614 | 0.023809 | 0.041929 | 0.012678 | MAPK1 | 1 |
| BP | GO:0048313 | Golgi inheritance | 1/32 | 14/18614 | 0.023809 | 0.041929 | 0.012678 | MAPK1 | 1 |
| BP | GO:0050930 | induction of positive chemotaxis | 1/32 | 14/18614 | 0.023809 | 0.041929 | 0.012678 | CXCL8 | 1 |
| BP | GO:0051547 | regulation of keratinocyte migration | 1/32 | 14/18614 | 0.023809 | 0.041929 | 0.012678 | MMP9 | 1 |
| BP | GO:0060009 | Sertoli cell development | 1/32 | 14/18614 | 0.023809 | 0.041929 | 0.012678 | IL1A | 1 |
| BP | GO:0060330 | regulation of response to type II interferon | 1/32 | 14/18614 | 0.023809 | 0.041929 | 0.012678 | PPARG | 1 |
| BP | GO:0060334 | regulation of type II interferon-mediated signaling pathway | 1/32 | 14/18614 | 0.023809 | 0.041929 | 0.012678 | PPARG | 1 |
| BP | GO:0061029 | eyelid development in camera-type eye | 1/32 | 14/18614 | 0.023809 | 0.041929 | 0.012678 | EGFR | 1 |
| BP | GO:0061043 | regulation of vascular wound healing | 1/32 | 14/18614 | 0.023809 | 0.041929 | 0.012678 | SERPINE1 | 1 |
| BP | GO:0072393 | microtubule anchoring at microtubule organizing center | 1/32 | 14/18614 | 0.023809 | 0.041929 | 0.012678 | GSK3B | 1 |
| BP | GO:0098814 | spontaneous synaptic transmission | 1/32 | 14/18614 | 0.023809 | 0.041929 | 0.012678 | APP | 1 |
| BP | GO:1901533 | negative regulation of hematopoietic progenitor cell differentiation | 1/32 | 14/18614 | 0.023809 | 0.041929 | 0.012678 | NFE2L2 | 1 |
| BP | GO:1902947 | regulation of tau-protein kinase activity | 1/32 | 14/18614 | 0.023809 | 0.041929 | 0.012678 | IFNG | 1 |
| BP | GO:1903798 | regulation of miRNA maturation | 1/32 | 14/18614 | 0.023809 | 0.041929 | 0.012678 | TP53 | 1 |
| BP | GO:1905203 | regulation of connective tissue replacement | 1/32 | 14/18614 | 0.023809 | 0.041929 | 0.012678 | PPARG | 1 |
| BP | GO:0010639 | negative regulation of organelle organization | 3/32 | 362/18614 | 0.023851 | 0.041976 | 0.012692 | AKT1/TP53/PPARG | 3 |
| BP | GO:0007093 | mitotic cell cycle checkpoint signaling | 2/32 | 140/18614 | 0.024041 | 0.042228 | 0.012768 | TP53/CCND1 | 2 |
| BP | GO:0050852 | T cell receptor signaling pathway | 2/32 | 140/18614 | 0.024041 | 0.042228 | 0.012768 | RELA/MAPK1 | 2 |
| BP | GO:1903008 | organelle disassembly | 2/32 | 140/18614 | 0.024041 | 0.042228 | 0.012768 | TP53/HIF1A | 2 |
| BP | GO:0043087 | regulation of GTPase activity | 3/32 | 364/18614 | 0.024194 | 0.042469 | 0.012841 | GSK3B/ERBB2/CCL2 | 3 |
| BP | GO:0006986 | response to unfolded protein | 2/32 | 142/18614 | 0.024683 | 0.043299 | 0.013092 | CCND1/NFE2L2 | 2 |
| BP | GO:1903900 | regulation of viral life cycle | 2/32 | 143/18614 | 0.025007 | 0.043529 | 0.013162 | BCL2/CXCL8 | 2 |
| BP | GO:0042552 | myelination | 2/32 | 144/18614 | 0.025332 | 0.043529 | 0.013162 | AKT1/ERBB2 | 2 |
| BP | GO:0098754 | detoxification | 2/32 | 144/18614 | 0.025332 | 0.043529 | 0.013162 | PTGS2/NFE2L2 | 2 |
| BP | GO:0001818 | negative regulation of cytokine production | 3/32 | 371/18614 | 0.025415 | 0.043529 | 0.013162 | IL10/IFNG/HMOX1 | 3 |
| BP | GO:0003376 | sphingosine-1-phosphate receptor signaling pathway | 1/32 | 15/18614 | 0.025488 | 0.043529 | 0.013162 | AKT1 | 1 |
| BP | GO:0006206 | pyrimidine nucleobase metabolic process | 1/32 | 15/18614 | 0.025488 | 0.043529 | 0.013162 | MAPK1 | 1 |
| BP | GO:0007567 | parturition | 1/32 | 15/18614 | 0.025488 | 0.043529 | 0.013162 | MMP2 | 1 |
| BP | GO:0010225 | response to UV-C | 1/32 | 15/18614 | 0.025488 | 0.043529 | 0.013162 | TP53 | 1 |
| BP | GO:0010649 | regulation of cell communication by electrical coupling | 1/32 | 15/18614 | 0.025488 | 0.043529 | 0.013162 | CAV1 | 1 |
| BP | GO:0010889 | regulation of sequestering of triglyceride | 1/32 | 15/18614 | 0.025488 | 0.043529 | 0.013162 | PPARG | 1 |
| BP | GO:0032352 | positive regulation of hormone metabolic process | 1/32 | 15/18614 | 0.025488 | 0.043529 | 0.013162 | HIF1A | 1 |
| BP | GO:0032725 | positive regulation of granulocyte macrophage colony-stimulating factor production | 1/32 | 15/18614 | 0.025488 | 0.043529 | 0.013162 | IL1B | 1 |
| BP | GO:0033599 | regulation of mammary gland epithelial cell proliferation | 1/32 | 15/18614 | 0.025488 | 0.043529 | 0.013162 | CCND1 | 1 |
| BP | GO:0033689 | negative regulation of osteoblast proliferation | 1/32 | 15/18614 | 0.025488 | 0.043529 | 0.013162 | BCL2 | 1 |
| BP | GO:0038166 | angiotensin-activated signaling pathway | 1/32 | 15/18614 | 0.025488 | 0.043529 | 0.013162 | CAV1 | 1 |
| BP | GO:0042532 | negative regulation of tyrosine phosphorylation of STAT protein | 1/32 | 15/18614 | 0.025488 | 0.043529 | 0.013162 | CAV1 | 1 |
| BP | GO:0042994 | cytoplasmic sequestering of transcription factor | 1/32 | 15/18614 | 0.025488 | 0.043529 | 0.013162 | IL10 | 1 |
| BP | GO:0043374 | CD8-positive, alpha-beta T cell differentiation | 1/32 | 15/18614 | 0.025488 | 0.043529 | 0.013162 | BCL2 | 1 |
| BP | GO:0044406 | adhesion of symbiont to host | 1/32 | 15/18614 | 0.025488 | 0.043529 | 0.013162 | ICAM1 | 1 |
| BP | GO:0045725 | positive regulation of glycogen biosynthetic process | 1/32 | 15/18614 | 0.025488 | 0.043529 | 0.013162 | AKT1 | 1 |
| BP | GO:0045838 | positive regulation of membrane potential | 1/32 | 15/18614 | 0.025488 | 0.043529 | 0.013162 | AKT1 | 1 |
| BP | GO:0045986 | negative regulation of smooth muscle contraction | 1/32 | 15/18614 | 0.025488 | 0.043529 | 0.013162 | PTGS2 | 1 |
| BP | GO:0046007 | negative regulation of activated T cell proliferation | 1/32 | 15/18614 | 0.025488 | 0.043529 | 0.013162 | CASP3 | 1 |
| BP | GO:0046322 | negative regulation of fatty acid oxidation | 1/32 | 15/18614 | 0.025488 | 0.043529 | 0.013162 | AKT1 | 1 |
| BP | GO:0048070 | regulation of developmental pigmentation | 1/32 | 15/18614 | 0.025488 | 0.043529 | 0.013162 | BCL2 | 1 |
| BP | GO:0060354 | negative regulation of cell adhesion molecule production | 1/32 | 15/18614 | 0.025488 | 0.043529 | 0.013162 | CXCL8 | 1 |
| BP | GO:0061307 | cardiac neural crest cell differentiation involved in heart development | 1/32 | 15/18614 | 0.025488 | 0.043529 | 0.013162 | MAPK1 | 1 |
| BP | GO:0061308 | cardiac neural crest cell development involved in heart development | 1/32 | 15/18614 | 0.025488 | 0.043529 | 0.013162 | MAPK1 | 1 |
| BP | GO:0070885 | negative regulation of calcineurin-NFAT signaling cascade | 1/32 | 15/18614 | 0.025488 | 0.043529 | 0.013162 | GSK3B | 1 |
| BP | GO:0070920 | regulation of production of small RNA involved in gene silencing by RNA | 1/32 | 15/18614 | 0.025488 | 0.043529 | 0.013162 | TP53 | 1 |
| BP | GO:0071380 | cellular response to prostaglandin E stimulus | 1/32 | 15/18614 | 0.025488 | 0.043529 | 0.013162 | AKT1 | 1 |
| BP | GO:0071639 | positive regulation of monocyte chemotactic protein-1 production | 1/32 | 15/18614 | 0.025488 | 0.043529 | 0.013162 | IL1B | 1 |
| BP | GO:0072075 | metanephric mesenchyme development | 1/32 | 15/18614 | 0.025488 | 0.043529 | 0.013162 | MYC | 1 |
| BP | GO:0090280 | positive regulation of calcium ion import | 1/32 | 15/18614 | 0.025488 | 0.043529 | 0.013162 | CCL2 | 1 |
| BP | GO:0097152 | mesenchymal cell apoptotic process | 1/32 | 15/18614 | 0.025488 | 0.043529 | 0.013162 | HIF1A | 1 |
| BP | GO:0106057 | negative regulation of calcineurin-mediated signaling | 1/32 | 15/18614 | 0.025488 | 0.043529 | 0.013162 | GSK3B | 1 |
| BP | GO:0120305 | regulation of pigmentation | 1/32 | 15/18614 | 0.025488 | 0.043529 | 0.013162 | BCL2 | 1 |
| BP | GO:1901722 | regulation of cell proliferation involved in kidney development | 1/32 | 15/18614 | 0.025488 | 0.043529 | 0.013162 | MYC | 1 |
| BP | GO:1902166 | negative regulation of intrinsic apoptotic signaling pathway in response to DNA damage by p53 class mediator | 1/32 | 15/18614 | 0.025488 | 0.043529 | 0.013162 | BCL2 | 1 |
| BP | GO:1903543 | positive regulation of exosomal secretion | 1/32 | 15/18614 | 0.025488 | 0.043529 | 0.013162 | IFNG | 1 |
| BP | GO:1905208 | negative regulation of cardiocyte differentiation | 1/32 | 15/18614 | 0.025488 | 0.043529 | 0.013162 | EGFR | 1 |
| BP | GO:2000671 | regulation of motor neuron apoptotic process | 1/32 | 15/18614 | 0.025488 | 0.043529 | 0.013162 | BCL2 | 1 |
| BP | GO:0007272 | ensheathment of neurons | 2/32 | 146/18614 | 0.025988 | 0.044299 | 0.013394 | AKT1/ERBB2 | 2 |
| BP | GO:0008366 | axon ensheathment | 2/32 | 146/18614 | 0.025988 | 0.044299 | 0.013394 | AKT1/ERBB2 | 2 |
| BP | GO:0072073 | kidney epithelium development | 2/32 | 146/18614 | 0.025988 | 0.044299 | 0.013394 | BCL2/MYC | 2 |
| BP | GO:0002903 | negative regulation of B cell apoptotic process | 1/32 | 16/18614 | 0.027165 | 0.045873 | 0.01387 | BCL2 | 1 |
| BP | GO:0006750 | glutathione biosynthetic process | 1/32 | 16/18614 | 0.027165 | 0.045873 | 0.01387 | NFE2L2 | 1 |
| BP | GO:0015671 | oxygen transport | 1/32 | 16/18614 | 0.027165 | 0.045873 | 0.01387 | MYC | 1 |
| BP | GO:0021534 | cell proliferation in hindbrain | 1/32 | 16/18614 | 0.027165 | 0.045873 | 0.01387 | EGF | 1 |
| BP | GO:0021924 | cell proliferation in external granule layer | 1/32 | 16/18614 | 0.027165 | 0.045873 | 0.01387 | EGF | 1 |
| BP | GO:0021930 | cerebellar granule cell precursor proliferation | 1/32 | 16/18614 | 0.027165 | 0.045873 | 0.01387 | EGF | 1 |
| BP | GO:0045579 | positive regulation of B cell differentiation | 1/32 | 16/18614 | 0.027165 | 0.045873 | 0.01387 | IL10 | 1 |
| BP | GO:0047484 | regulation of response to osmotic stress | 1/32 | 16/18614 | 0.027165 | 0.045873 | 0.01387 | PTGS2 | 1 |
| BP | GO:0050862 | positive regulation of T cell receptor signaling pathway | 1/32 | 16/18614 | 0.027165 | 0.045873 | 0.01387 | RELA | 1 |
| BP | GO:0060392 | negative regulation of SMAD protein signal transduction | 1/32 | 16/18614 | 0.027165 | 0.045873 | 0.01387 | PPARG | 1 |
| BP | GO:0060576 | intestinal epithelial cell development | 1/32 | 16/18614 | 0.027165 | 0.045873 | 0.01387 | HIF1A | 1 |
| BP | GO:0070431 | nucleotide-binding oligomerization domain containing 2 signaling pathway | 1/32 | 16/18614 | 0.027165 | 0.045873 | 0.01387 | RELA | 1 |
| BP | GO:0070875 | positive regulation of glycogen metabolic process | 1/32 | 16/18614 | 0.027165 | 0.045873 | 0.01387 | AKT1 | 1 |
| BP | GO:0090336 | positive regulation of brown fat cell differentiation | 1/32 | 16/18614 | 0.027165 | 0.045873 | 0.01387 | PTGS2 | 1 |
| BP | GO:2000696 | regulation of epithelial cell differentiation involved in kidney development | 1/32 | 16/18614 | 0.027165 | 0.045873 | 0.01387 | MMP9 | 1 |
| BP | GO:0050768 | negative regulation of neurogenesis | 2/32 | 150/18614 | 0.02732 | 0.046107 | 0.013941 | IL1B/TP53 | 2 |
| BP | GO:0016525 | negative regulation of angiogenesis | 2/32 | 151/18614 | 0.027658 | 0.046647 | 0.014104 | PPARG/SERPINE1 | 2 |
| BP | GO:0001654 | eye development | 3/32 | 385/18614 | 0.027957 | 0.047102 | 0.014242 | EGFR/BCL2/HIF1A | 3 |
| BP | GO:0016331 | morphogenesis of embryonic epithelium | 2/32 | 152/18614 | 0.027997 | 0.047102 | 0.014242 | CASP3/HIF1A | 2 |
| BP | GO:0072006 | nephron development | 2/32 | 152/18614 | 0.027997 | 0.047102 | 0.014242 | BCL2/MYC | 2 |
| BP | GO:1904064 | positive regulation of cation transmembrane transport | 2/32 | 152/18614 | 0.027997 | 0.047102 | 0.014242 | IFNG/CAV1 | 2 |
| BP | GO:0006352 | DNA-templated transcription initiation | 2/32 | 153/18614 | 0.028338 | 0.047616 | 0.014397 | TP53/ESR1 | 2 |
| BP | GO:2000181 | negative regulation of blood vessel morphogenesis | 2/32 | 153/18614 | 0.028338 | 0.047616 | 0.014397 | PPARG/SERPINE1 | 2 |
| BP | GO:1901343 | negative regulation of vasculature development | 2/32 | 154/18614 | 0.02868 | 0.04769 | 0.01442 | PPARG/SERPINE1 | 2 |
| BP | GO:2001257 | regulation of cation channel activity | 2/32 | 154/18614 | 0.02868 | 0.04769 | 0.01442 | MMP9/CAV1 | 2 |
| BP | GO:0150063 | visual system development | 3/32 | 389/18614 | 0.028707 | 0.04769 | 0.01442 | EGFR/BCL2/HIF1A | 3 |
| BP | GO:0009404 | toxin metabolic process | 1/32 | 17/18614 | 0.028839 | 0.04769 | 0.01442 | NFE2L2 | 1 |
| BP | GO:0010763 | positive regulation of fibroblast migration | 1/32 | 17/18614 | 0.028839 | 0.04769 | 0.01442 | AKT1 | 1 |
| BP | GO:0019184 | nonribosomal peptide biosynthetic process | 1/32 | 17/18614 | 0.028839 | 0.04769 | 0.01442 | NFE2L2 | 1 |
| BP | GO:0034138 | toll-like receptor 3 signaling pathway | 1/32 | 17/18614 | 0.028839 | 0.04769 | 0.01442 | CAV1 | 1 |
| BP | GO:0044539 | long-chain fatty acid import into cell | 1/32 | 17/18614 | 0.028839 | 0.04769 | 0.01442 | AKT1 | 1 |
| BP | GO:0050872 | white fat cell differentiation | 1/32 | 17/18614 | 0.028839 | 0.04769 | 0.01442 | PPARG | 1 |
| BP | GO:0055070 | copper ion homeostasis | 1/32 | 17/18614 | 0.028839 | 0.04769 | 0.01442 | APP | 1 |
| BP | GO:0060253 | negative regulation of glial cell proliferation | 1/32 | 17/18614 | 0.028839 | 0.04769 | 0.01442 | TP53 | 1 |
| BP | GO:0071360 | cellular response to exogenous dsRNA | 1/32 | 17/18614 | 0.028839 | 0.04769 | 0.01442 | CAV1 | 1 |
| BP | GO:0097396 | response to interleukin-17 | 1/32 | 17/18614 | 0.028839 | 0.04769 | 0.01442 | IL1B | 1 |
| BP | GO:0097398 | cellular response to interleukin-17 | 1/32 | 17/18614 | 0.028839 | 0.04769 | 0.01442 | IL1B | 1 |
| BP | GO:1900034 | regulation of cellular response to heat | 1/32 | 17/18614 | 0.028839 | 0.04769 | 0.01442 | GSK3B | 1 |
| BP | GO:1901550 | regulation of endothelial cell development | 1/32 | 17/18614 | 0.028839 | 0.04769 | 0.01442 | IL1B | 1 |
| BP | GO:1902036 | regulation of hematopoietic stem cell differentiation | 1/32 | 17/18614 | 0.028839 | 0.04769 | 0.01442 | NFE2L2 | 1 |
| BP | GO:1902165 | regulation of intrinsic apoptotic signaling pathway in response to DNA damage by p53 class mediator | 1/32 | 17/18614 | 0.028839 | 0.04769 | 0.01442 | BCL2 | 1 |
| BP | GO:1903140 | regulation of establishment of endothelial barrier | 1/32 | 17/18614 | 0.028839 | 0.04769 | 0.01442 | IL1B | 1 |
| BP | GO:1903358 | regulation of Golgi organization | 1/32 | 17/18614 | 0.028839 | 0.04769 | 0.01442 | MAPK1 | 1 |
| BP | GO:1903541 | regulation of exosomal secretion | 1/32 | 17/18614 | 0.028839 | 0.04769 | 0.01442 | IFNG | 1 |
| BP | GO:1904355 | positive regulation of telomere capping | 1/32 | 17/18614 | 0.028839 | 0.04769 | 0.01442 | MAPK1 | 1 |
| BP | GO:1905288 | vascular associated smooth muscle cell apoptotic process | 1/32 | 17/18614 | 0.028839 | 0.04769 | 0.01442 | PPARG | 1 |
| BP | GO:1905459 | regulation of vascular associated smooth muscle cell apoptotic process | 1/32 | 17/18614 | 0.028839 | 0.04769 | 0.01442 | PPARG | 1 |
| BP | GO:1905906 | regulation of amyloid fibril formation | 1/32 | 17/18614 | 0.028839 | 0.04769 | 0.01442 | APP | 1 |
| BP | GO:2001267 | regulation of cysteine-type endopeptidase activity involved in apoptotic signaling pathway | 1/32 | 17/18614 | 0.028839 | 0.04769 | 0.01442 | MMP9 | 1 |
| BP | GO:0008037 | cell recognition | 2/32 | 155/18614 | 0.029024 | 0.047967 | 0.014503 | CASP3/APP | 2 |
| BP | GO:0000723 | telomere maintenance | 2/32 | 156/18614 | 0.02937 | 0.04845 | 0.014649 | MYC/MAPK1 | 2 |
| BP | GO:0051961 | negative regulation of nervous system development | 2/32 | 156/18614 | 0.02937 | 0.04845 | 0.014649 | IL1B/TP53 | 2 |
| BP | GO:1903531 | negative regulation of secretion by cell | 2/32 | 156/18614 | 0.02937 | 0.04845 | 0.014649 | IL1B/HMOX1 | 2 |
| BP | GO:0007043 | cell-cell junction assembly | 2/32 | 157/18614 | 0.029718 | 0.048964 | 0.014805 | IL1B/CAV1 | 2 |
| BP | GO:0048592 | eye morphogenesis | 2/32 | 157/18614 | 0.029718 | 0.048964 | 0.014805 | BCL2/HIF1A | 2 |
| BP | GO:0048880 | sensory system development | 3/32 | 395/18614 | 0.029853 | 0.049156 | 0.014863 | EGFR/BCL2/HIF1A | 3 |
| BP | GO:0002693 | positive regulation of cellular extravasation | 1/32 | 18/18614 | 0.03051 | 0.04937 | 0.014927 | ICAM1 | 1 |
| BP | GO:0002827 | positive regulation of T-helper 1 type immune response | 1/32 | 18/18614 | 0.03051 | 0.04937 | 0.014927 | IL1B | 1 |
| BP | GO:0006977 | DNA damage response, signal transduction by p53 class mediator resulting in cell cycle arrest | 1/32 | 18/18614 | 0.03051 | 0.04937 | 0.014927 | TP53 | 1 |
| BP | GO:0006978 | DNA damage response, signal transduction by p53 class mediator resulting in transcription of p21 class mediator | 1/32 | 18/18614 | 0.03051 | 0.04937 | 0.014927 | TP53 | 1 |
| BP | GO:0010612 | regulation of cardiac muscle adaptation | 1/32 | 18/18614 | 0.03051 | 0.04937 | 0.014927 | PPARG | 1 |
| BP | GO:0010663 | positive regulation of striated muscle cell apoptotic process | 1/32 | 18/18614 | 0.03051 | 0.04937 | 0.014927 | TP53 | 1 |
| BP | GO:0010666 | positive regulation of cardiac muscle cell apoptotic process | 1/32 | 18/18614 | 0.03051 | 0.04937 | 0.014927 | TP53 | 1 |
| BP | GO:0010755 | regulation of plasminogen activation | 1/32 | 18/18614 | 0.03051 | 0.04937 | 0.014927 | SERPINE1 | 1 |
| BP | GO:0010885 | regulation of cholesterol storage | 1/32 | 18/18614 | 0.03051 | 0.04937 | 0.014927 | PPARG | 1 |
| BP | GO:0019896 | axonal transport of mitochondrion | 1/32 | 18/18614 | 0.03051 | 0.04937 | 0.014927 | HIF1A | 1 |
| BP | GO:0020027 | hemoglobin metabolic process | 1/32 | 18/18614 | 0.03051 | 0.04937 | 0.014927 | HIF1A | 1 |
| BP | GO:0030011 | maintenance of cell polarity | 1/32 | 18/18614 | 0.03051 | 0.04937 | 0.014927 | GSK3B | 1 |
| BP | GO:0031645 | negative regulation of nervous system process | 1/32 | 18/18614 | 0.03051 | 0.04937 | 0.014927 | IL10 | 1 |
| BP | GO:0032604 | granulocyte macrophage colony-stimulating factor production | 1/32 | 18/18614 | 0.03051 | 0.04937 | 0.014927 | IL1B | 1 |
| BP | GO:0032645 | regulation of granulocyte macrophage colony-stimulating factor production | 1/32 | 18/18614 | 0.03051 | 0.04937 | 0.014927 | IL1B | 1 |
| BP | GO:0032769 | negative regulation of monooxygenase activity | 1/32 | 18/18614 | 0.03051 | 0.04937 | 0.014927 | CAV1 | 1 |
| BP | GO:0033033 | negative regulation of myeloid cell apoptotic process | 1/32 | 18/18614 | 0.03051 | 0.04937 | 0.014927 | BCL2 | 1 |
| BP | GO:0036499 | PERK-mediated unfolded protein response | 1/32 | 18/18614 | 0.03051 | 0.04937 | 0.014927 | NFE2L2 | 1 |
| BP | GO:0043217 | myelin maintenance | 1/32 | 18/18614 | 0.03051 | 0.04937 | 0.014927 | AKT1 | 1 |
| BP | GO:0051782 | negative regulation of cell division | 1/32 | 18/18614 | 0.03051 | 0.04937 | 0.014927 | MYC | 1 |
| BP | GO:0051917 | regulation of fibrinolysis | 1/32 | 18/18614 | 0.03051 | 0.04937 | 0.014927 | SERPINE1 | 1 |
| BP | GO:0060602 | branch elongation of an epithelium | 1/32 | 18/18614 | 0.03051 | 0.04937 | 0.014927 | ESR1 | 1 |
| BP | GO:0061298 | retina vasculature development in camera-type eye | 1/32 | 18/18614 | 0.03051 | 0.04937 | 0.014927 | HIF1A | 1 |
| BP | GO:0071318 | cellular response to ATP | 1/32 | 18/18614 | 0.03051 | 0.04937 | 0.014927 | PTGS2 | 1 |
| BP | GO:0071391 | cellular response to estrogen stimulus | 1/32 | 18/18614 | 0.03051 | 0.04937 | 0.014927 | ESR1 | 1 |
| BP | GO:0086103 | G protein-coupled receptor signaling pathway involved in heart process | 1/32 | 18/18614 | 0.03051 | 0.04937 | 0.014927 | CAV1 | 1 |
| BP | GO:0090520 | sphingolipid mediated signaling pathway | 1/32 | 18/18614 | 0.03051 | 0.04937 | 0.014927 | AKT1 | 1 |
| BP | GO:0150078 | positive regulation of neuroinflammatory response | 1/32 | 18/18614 | 0.03051 | 0.04937 | 0.014927 | IL1B | 1 |
| BP | GO:1903242 | regulation of cardiac muscle hypertrophy in response to stress | 1/32 | 18/18614 | 0.03051 | 0.04937 | 0.014927 | PPARG | 1 |
| BP | GO:0006633 | fatty acid biosynthetic process | 2/32 | 161/18614 | 0.031124 | 0.050303 | 0.01521 | IL1B/PTGS2 | 2 |
| BP | GO:0035051 | cardiocyte differentiation | 2/32 | 161/18614 | 0.031124 | 0.050303 | 0.01521 | EGFR/MAPK1 | 2 |
| BP | GO:0006338 | chromatin remodeling | 3/32 | 402/18614 | 0.03122 | 0.050428 | 0.015247 | TP53/ESR1/MYC | 3 |
| BP | GO:0021915 | neural tube development | 2/32 | 163/18614 | 0.031837 | 0.051362 | 0.01553 | CASP3/HIF1A | 2 |
| BP | GO:0002281 | macrophage activation involved in immune response | 1/32 | 19/18614 | 0.032178 | 0.051362 | 0.01553 | IFNG | 1 |
| BP | GO:0006098 | pentose-phosphate shunt | 1/32 | 19/18614 | 0.032178 | 0.051362 | 0.01553 | TP53 | 1 |
| BP | GO:0010759 | positive regulation of macrophage chemotaxis | 1/32 | 19/18614 | 0.032178 | 0.051362 | 0.01553 | MAPK1 | 1 |
| BP | GO:0030540 | female genitalia development | 1/32 | 19/18614 | 0.032178 | 0.051362 | 0.01553 | ESR1 | 1 |
| BP | GO:0032026 | response to magnesium ion | 1/32 | 19/18614 | 0.032178 | 0.051362 | 0.01553 | CCND1 | 1 |
| BP | GO:0032695 | negative regulation of interleukin-12 production | 1/32 | 19/18614 | 0.032178 | 0.051362 | 0.01553 | IL10 | 1 |
| BP | GO:0035743 | CD4-positive, alpha-beta T cell cytokine production | 1/32 | 19/18614 | 0.032178 | 0.051362 | 0.01553 | IL1B | 1 |
| BP | GO:0042772 | DNA damage response, signal transduction resulting in transcription | 1/32 | 19/18614 | 0.032178 | 0.051362 | 0.01553 | TP53 | 1 |
| BP | GO:0045091 | regulation of single stranded viral RNA replication via double stranded DNA intermediate | 1/32 | 19/18614 | 0.032178 | 0.051362 | 0.01553 | CXCL8 | 1 |
| BP | GO:0048266 | behavioral response to pain | 1/32 | 19/18614 | 0.032178 | 0.051362 | 0.01553 | AKT1 | 1 |
| BP | GO:0051900 | regulation of mitochondrial depolarization | 1/32 | 19/18614 | 0.032178 | 0.051362 | 0.01553 | BCL2 | 1 |
| BP | GO:0060391 | positive regulation of SMAD protein signal transduction | 1/32 | 19/18614 | 0.032178 | 0.051362 | 0.01553 | PPARG | 1 |
| BP | GO:0060438 | trachea development | 1/32 | 19/18614 | 0.032178 | 0.051362 | 0.01553 | MAPK1 | 1 |
| BP | GO:0072074 | kidney mesenchyme development | 1/32 | 19/18614 | 0.032178 | 0.051362 | 0.01553 | MYC | 1 |
| BP | GO:0097202 | activation of cysteine-type endopeptidase activity | 1/32 | 19/18614 | 0.032178 | 0.051362 | 0.01553 | CASP9 | 1 |
| BP | GO:0140354 | lipid import into cell | 1/32 | 19/18614 | 0.032178 | 0.051362 | 0.01553 | AKT1 | 1 |
| BP | GO:2000434 | regulation of protein neddylation | 1/32 | 19/18614 | 0.032178 | 0.051362 | 0.01553 | HIF1A | 1 |
| BP | GO:2000641 | regulation of early endosome to late endosome transport | 1/32 | 19/18614 | 0.032178 | 0.051362 | 0.01553 | MAPK1 | 1 |
| BP | GO:2000774 | positive regulation of cellular senescence | 1/32 | 19/18614 | 0.032178 | 0.051362 | 0.01553 | TP53 | 1 |
| BP | GO:0035966 | response to topologically incorrect protein | 2/32 | 164/18614 | 0.032196 | 0.051362 | 0.01553 | CCND1/NFE2L2 | 2 |
| BP | GO:0048639 | positive regulation of developmental growth | 2/32 | 165/18614 | 0.032557 | 0.051877 | 0.015685 | AKT1/BCL2 | 2 |
| BP | GO:0050792 | regulation of viral process | 2/32 | 165/18614 | 0.032557 | 0.051877 | 0.015685 | BCL2/CXCL8 | 2 |
| BP | GO:0042692 | muscle cell differentiation | 3/32 | 410/18614 | 0.032823 | 0.05227 | 0.015804 | AKT1/CASP3/BCL2 | 3 |
| BP | GO:0003205 | cardiac chamber development | 2/32 | 166/18614 | 0.032919 | 0.052361 | 0.015832 | TP53/HIF1A | 2 |
| BP | GO:0051168 | nuclear export | 2/32 | 166/18614 | 0.032919 | 0.052361 | 0.015832 | IL1B/GSK3B | 2 |
| BP | GO:2001022 | positive regulation of response to DNA damage stimulus | 2/32 | 167/18614 | 0.033283 | 0.052909 | 0.015998 | EGFR/MYC | 2 |
| BP | GO:0002544 | chronic inflammatory response | 1/32 | 20/18614 | 0.033844 | 0.053085 | 0.016051 | IL10 | 1 |
| BP | GO:0008340 | determination of adult lifespan | 1/32 | 20/18614 | 0.033844 | 0.053085 | 0.016051 | TP53 | 1 |
| BP | GO:0009110 | vitamin biosynthetic process | 1/32 | 20/18614 | 0.033844 | 0.053085 | 0.016051 | IFNG | 1 |
| BP | GO:0010878 | cholesterol storage | 1/32 | 20/18614 | 0.033844 | 0.053085 | 0.016051 | PPARG | 1 |
| BP | GO:0010940 | positive regulation of necrotic cell death | 1/32 | 20/18614 | 0.033844 | 0.053085 | 0.016051 | TP53 | 1 |
| BP | GO:0031998 | regulation of fatty acid beta-oxidation | 1/32 | 20/18614 | 0.033844 | 0.053085 | 0.016051 | AKT1 | 1 |
| BP | GO:0032495 | response to muramyl dipeptide | 1/32 | 20/18614 | 0.033844 | 0.053085 | 0.016051 | RELA | 1 |
| BP | GO:0033141 | positive regulation of peptidyl-serine phosphorylation of STAT protein | 1/32 | 20/18614 | 0.033844 | 0.053085 | 0.016051 | IFNG | 1 |
| BP | GO:0034755 | iron ion transmembrane transport | 1/32 | 20/18614 | 0.033844 | 0.053085 | 0.016051 | IFNG | 1 |
| BP | GO:0035902 | response to immobilization stress | 1/32 | 20/18614 | 0.033844 | 0.053085 | 0.016051 | FOS | 1 |
| BP | GO:0039692 | single stranded viral RNA replication via double stranded DNA intermediate | 1/32 | 20/18614 | 0.033844 | 0.053085 | 0.016051 | CXCL8 | 1 |
| BP | GO:0042359 | vitamin D metabolic process | 1/32 | 20/18614 | 0.033844 | 0.053085 | 0.016051 | IFNG | 1 |
| BP | GO:0042953 | lipoprotein transport | 1/32 | 20/18614 | 0.033844 | 0.053085 | 0.016051 | PPARG | 1 |
| BP | GO:0045655 | regulation of monocyte differentiation | 1/32 | 20/18614 | 0.033844 | 0.053085 | 0.016051 | MYC | 1 |
| BP | GO:0048643 | positive regulation of skeletal muscle tissue development | 1/32 | 20/18614 | 0.033844 | 0.053085 | 0.016051 | BCL2 | 1 |
| BP | GO:0051546 | keratinocyte migration | 1/32 | 20/18614 | 0.033844 | 0.053085 | 0.016051 | MMP9 | 1 |
| BP | GO:0070314 | G1 to G0 transition | 1/32 | 20/18614 | 0.033844 | 0.053085 | 0.016051 | TP53 | 1 |
| BP | GO:0071243 | cellular response to arsenic-containing substance | 1/32 | 20/18614 | 0.033844 | 0.053085 | 0.016051 | HMOX1 | 1 |
| BP | GO:0071379 | cellular response to prostaglandin stimulus | 1/32 | 20/18614 | 0.033844 | 0.053085 | 0.016051 | AKT1 | 1 |
| BP | GO:1900221 | regulation of amyloid-beta clearance | 1/32 | 20/18614 | 0.033844 | 0.053085 | 0.016051 | IFNG | 1 |
| BP | GO:1902001 | fatty acid transmembrane transport | 1/32 | 20/18614 | 0.033844 | 0.053085 | 0.016051 | AKT1 | 1 |
| BP | GO:1990182 | exosomal secretion | 1/32 | 20/18614 | 0.033844 | 0.053085 | 0.016051 | IFNG | 1 |
| BP | GO:2000269 | regulation of fibroblast apoptotic process | 1/32 | 20/18614 | 0.033844 | 0.053085 | 0.016051 | TP53 | 1 |
| BP | GO:0007009 | plasma membrane organization | 2/32 | 170/18614 | 0.034384 | 0.053901 | 0.016297 | AKT1/CAV1 | 2 |
| BP | GO:0010970 | transport along microtubule | 2/32 | 172/18614 | 0.035126 | 0.054713 | 0.016543 | HIF1A/APP | 2 |
| BP | GO:0055088 | lipid homeostasis | 2/32 | 172/18614 | 0.035126 | 0.054713 | 0.016543 | PPARG/CAV1 | 2 |
| BP | GO:0006937 | regulation of muscle contraction | 2/32 | 173/18614 | 0.035499 | 0.054713 | 0.016543 | PTGS2/CAV1 | 2 |
| BP | GO:0031214 | biomineral tissue development | 2/32 | 173/18614 | 0.035499 | 0.054713 | 0.016543 | PTGS2/HIF1A | 2 |
| BP | GO:0007252 | I-kappaB phosphorylation | 1/32 | 21/18614 | 0.035507 | 0.054713 | 0.016543 | AKT1 | 1 |
| BP | GO:0007413 | axonal fasciculation | 1/32 | 21/18614 | 0.035507 | 0.054713 | 0.016543 | CASP3 | 1 |
| BP | GO:0010829 | negative regulation of glucose transmembrane transport | 1/32 | 21/18614 | 0.035507 | 0.054713 | 0.016543 | IL1B | 1 |
| BP | GO:0010888 | negative regulation of lipid storage | 1/32 | 21/18614 | 0.035507 | 0.054713 | 0.016543 | PPARG | 1 |
| BP | GO:0014856 | skeletal muscle cell proliferation | 1/32 | 21/18614 | 0.035507 | 0.054713 | 0.016543 | FOS | 1 |
| BP | GO:0030502 | negative regulation of bone mineralization | 1/32 | 21/18614 | 0.035507 | 0.054713 | 0.016543 | HIF1A | 1 |
| BP | GO:0032986 | protein-DNA complex disassembly | 1/32 | 21/18614 | 0.035507 | 0.054713 | 0.016543 | MYC | 1 |
| BP | GO:0044321 | response to leptin | 1/32 | 21/18614 | 0.035507 | 0.054713 | 0.016543 | CCND1 | 1 |
| BP | GO:0044872 | lipoprotein localization | 1/32 | 21/18614 | 0.035507 | 0.054713 | 0.016543 | PPARG | 1 |
| BP | GO:0050849 | negative regulation of calcium-mediated signaling | 1/32 | 21/18614 | 0.035507 | 0.054713 | 0.016543 | GSK3B | 1 |
| BP | GO:0051220 | cytoplasmic sequestering of protein | 1/32 | 21/18614 | 0.035507 | 0.054713 | 0.016543 | IL10 | 1 |
| BP | GO:0051882 | mitochondrial depolarization | 1/32 | 21/18614 | 0.035507 | 0.054713 | 0.016543 | BCL2 | 1 |
| BP | GO:0060008 | Sertoli cell differentiation | 1/32 | 21/18614 | 0.035507 | 0.054713 | 0.016543 | IL1A | 1 |
| BP | GO:0060252 | positive regulation of glial cell proliferation | 1/32 | 21/18614 | 0.035507 | 0.054713 | 0.016543 | IL1B | 1 |
| BP | GO:0060546 | negative regulation of necroptotic process | 1/32 | 21/18614 | 0.035507 | 0.054713 | 0.016543 | CAV1 | 1 |
| BP | GO:0071605 | monocyte chemotactic protein-1 production | 1/32 | 21/18614 | 0.035507 | 0.054713 | 0.016543 | IL1B | 1 |
| BP | GO:0071637 | regulation of monocyte chemotactic protein-1 production | 1/32 | 21/18614 | 0.035507 | 0.054713 | 0.016543 | IL1B | 1 |
| BP | GO:0090026 | positive regulation of monocyte chemotaxis | 1/32 | 21/18614 | 0.035507 | 0.054713 | 0.016543 | SERPINE1 | 1 |
| BP | GO:0090201 | negative regulation of release of cytochrome c from mitochondria | 1/32 | 21/18614 | 0.035507 | 0.054713 | 0.016543 | AKT1 | 1 |
| BP | GO:0090370 | negative regulation of cholesterol efflux | 1/32 | 21/18614 | 0.035507 | 0.054713 | 0.016543 | EGF | 1 |
| BP | GO:0097049 | motor neuron apoptotic process | 1/32 | 21/18614 | 0.035507 | 0.054713 | 0.016543 | BCL2 | 1 |
| BP | GO:0097734 | extracellular exosome biogenesis | 1/32 | 21/18614 | 0.035507 | 0.054713 | 0.016543 | IFNG | 1 |
| BP | GO:0106030 | neuron projection fasciculation | 1/32 | 21/18614 | 0.035507 | 0.054713 | 0.016543 | CASP3 | 1 |
| BP | GO:1903429 | regulation of cell maturation | 1/32 | 21/18614 | 0.035507 | 0.054713 | 0.016543 | BCL2 | 1 |
| BP | GO:1903817 | negative regulation of voltage-gated potassium channel activity | 1/32 | 21/18614 | 0.035507 | 0.054713 | 0.016543 | CAV1 | 1 |
| BP | GO:2000010 | positive regulation of protein localization to cell surface | 1/32 | 21/18614 | 0.035507 | 0.054713 | 0.016543 | AKT1 | 1 |
| BP | GO:0030178 | negative regulation of Wnt signaling pathway | 2/32 | 174/18614 | 0.035874 | 0.055215 | 0.016695 | GSK3B/CAV1 | 2 |
| BP | GO:0030216 | keratinocyte differentiation | 2/32 | 174/18614 | 0.035874 | 0.055215 | 0.016695 | CASP3/IL1A | 2 |
| BP | GO:0060537 | muscle tissue development | 3/32 | 426/18614 | 0.036157 | 0.055619 | 0.016817 | BCL2/FOS/CAV1 | 3 |
| BP | GO:0006694 | steroid biosynthetic process | 2/32 | 175/18614 | 0.03625 | 0.0557 | 0.016841 | IFNG/IL1A | 2 |
| BP | GO:0046631 | alpha-beta T cell activation | 2/32 | 175/18614 | 0.03625 | 0.0557 | 0.016841 | BCL2/IFNG | 2 |
| BP | GO:0006740 | NADPH regeneration | 1/32 | 22/18614 | 0.037167 | 0.056595 | 0.017112 | TP53 | 1 |
| BP | GO:0006907 | pinocytosis | 1/32 | 22/18614 | 0.037167 | 0.056595 | 0.017112 | CAV1 | 1 |
| BP | GO:0015669 | gas transport | 1/32 | 22/18614 | 0.037167 | 0.056595 | 0.017112 | MYC | 1 |
| BP | GO:0030449 | regulation of complement activation | 1/32 | 22/18614 | 0.037167 | 0.056595 | 0.017112 | IL1B | 1 |
| BP | GO:0032042 | mitochondrial DNA metabolic process | 1/32 | 22/18614 | 0.037167 | 0.056595 | 0.017112 | TP53 | 1 |
| BP | GO:0032069 | regulation of nuclease activity | 1/32 | 22/18614 | 0.037167 | 0.056595 | 0.017112 | AKT1 | 1 |
| BP | GO:0032700 | negative regulation of interleukin-17 production | 1/32 | 22/18614 | 0.037167 | 0.056595 | 0.017112 | IFNG | 1 |
| BP | GO:0032780 | negative regulation of ATP-dependent activity | 1/32 | 22/18614 | 0.037167 | 0.056595 | 0.017112 | TP53 | 1 |
| BP | GO:0034104 | negative regulation of tissue remodeling | 1/32 | 22/18614 | 0.037167 | 0.056595 | 0.017112 | PPARG | 1 |
| BP | GO:0060444 | branching involved in mammary gland duct morphogenesis | 1/32 | 22/18614 | 0.037167 | 0.056595 | 0.017112 | ESR1 | 1 |
| BP | GO:0060713 | labyrinthine layer morphogenesis | 1/32 | 22/18614 | 0.037167 | 0.056595 | 0.017112 | IL10 | 1 |
| BP | GO:0062099 | negative regulation of programmed necrotic cell death | 1/32 | 22/18614 | 0.037167 | 0.056595 | 0.017112 | CAV1 | 1 |
| BP | GO:0071359 | cellular response to dsRNA | 1/32 | 22/18614 | 0.037167 | 0.056595 | 0.017112 | CAV1 | 1 |
| BP | GO:0071636 | positive regulation of transforming growth factor beta production | 1/32 | 22/18614 | 0.037167 | 0.056595 | 0.017112 | PTGS2 | 1 |
| BP | GO:0072111 | cell proliferation involved in kidney development | 1/32 | 22/18614 | 0.037167 | 0.056595 | 0.017112 | MYC | 1 |
| BP | GO:1903589 | positive regulation of blood vessel endothelial cell proliferation involved in sprouting angiogenesis | 1/32 | 22/18614 | 0.037167 | 0.056595 | 0.017112 | HMOX1 | 1 |
| BP | GO:0007219 | Notch signaling pathway | 2/32 | 179/18614 | 0.037771 | 0.057483 | 0.017381 | AKT1/APP | 2 |
| BP | GO:0045580 | regulation of T cell differentiation | 2/32 | 180/18614 | 0.038155 | 0.058035 | 0.017547 | ERBB2/IFNG | 2 |
| BP | GO:0002070 | epithelial cell maturation | 1/32 | 23/18614 | 0.038824 | 0.058462 | 0.017677 | HIF1A | 1 |
| BP | GO:0002363 | alpha-beta T cell lineage commitment | 1/32 | 23/18614 | 0.038824 | 0.058462 | 0.017677 | BCL2 | 1 |
| BP | GO:0002689 | negative regulation of leukocyte chemotaxis | 1/32 | 23/18614 | 0.038824 | 0.058462 | 0.017677 | CCL2 | 1 |
| BP | GO:0006883 | intracellular sodium ion homeostasis | 1/32 | 23/18614 | 0.038824 | 0.058462 | 0.017677 | IL1A | 1 |
| BP | GO:0019430 | removal of superoxide radicals | 1/32 | 23/18614 | 0.038824 | 0.058462 | 0.017677 | NFE2L2 | 1 |
| BP | GO:0031065 | positive regulation of histone deacetylation | 1/32 | 23/18614 | 0.038824 | 0.058462 | 0.017677 | TP53 | 1 |
| BP | GO:0032891 | negative regulation of organic acid transport | 1/32 | 23/18614 | 0.038824 | 0.058462 | 0.017677 | AKT1 | 1 |
| BP | GO:0033139 | regulation of peptidyl-serine phosphorylation of STAT protein | 1/32 | 23/18614 | 0.038824 | 0.058462 | 0.017677 | IFNG | 1 |
| BP | GO:0045591 | positive regulation of regulatory T cell differentiation | 1/32 | 23/18614 | 0.038824 | 0.058462 | 0.017677 | IFNG | 1 |
| BP | GO:0046794 | transport of virus | 1/32 | 23/18614 | 0.038824 | 0.058462 | 0.017677 | CAV1 | 1 |
| BP | GO:0048169 | regulation of long-term neuronal synaptic plasticity | 1/32 | 23/18614 | 0.038824 | 0.058462 | 0.017677 | APP | 1 |
| BP | GO:0060065 | uterus development | 1/32 | 23/18614 | 0.038824 | 0.058462 | 0.017677 | ESR1 | 1 |
| BP | GO:0061042 | vascular wound healing | 1/32 | 23/18614 | 0.038824 | 0.058462 | 0.017677 | SERPINE1 | 1 |
| BP | GO:0140112 | extracellular vesicle biogenesis | 1/32 | 23/18614 | 0.038824 | 0.058462 | 0.017677 | IFNG | 1 |
| BP | GO:1901984 | negative regulation of protein acetylation | 1/32 | 23/18614 | 0.038824 | 0.058462 | 0.017677 | GSK3B | 1 |
| BP | GO:1902254 | negative regulation of intrinsic apoptotic signaling pathway by p53 class mediator | 1/32 | 23/18614 | 0.038824 | 0.058462 | 0.017677 | BCL2 | 1 |
| BP | GO:2000178 | negative regulation of neural precursor cell proliferation | 1/32 | 23/18614 | 0.038824 | 0.058462 | 0.017677 | TP53 | 1 |
| BP | GO:2000353 | positive regulation of endothelial cell apoptotic process | 1/32 | 23/18614 | 0.038824 | 0.058462 | 0.017677 | CCL2 | 1 |
| BP | GO:0002706 | regulation of lymphocyte mediated immunity | 2/32 | 184/18614 | 0.039706 | 0.059758 | 0.018068 | IL1B/IL10 | 2 |
| BP | GO:0009150 | purine ribonucleotide metabolic process | 3/32 | 445/18614 | 0.040336 | 0.060237 | 0.018213 | HIF1A/IFNG/APP | 3 |
| BP | GO:0051656 | establishment of organelle localization | 3/32 | 445/18614 | 0.040336 | 0.060237 | 0.018213 | HIF1A/IFNG/HMOX1 | 3 |
| BP | GO:0002053 | positive regulation of mesenchymal cell proliferation | 1/32 | 24/18614 | 0.040478 | 0.060237 | 0.018213 | MYC | 1 |
| BP | GO:0002320 | lymphoid progenitor cell differentiation | 1/32 | 24/18614 | 0.040478 | 0.060237 | 0.018213 | BCL2 | 1 |
| BP | GO:0002922 | positive regulation of humoral immune response | 1/32 | 24/18614 | 0.040478 | 0.060237 | 0.018213 | IL1B | 1 |
| BP | GO:0008045 | motor neuron axon guidance | 1/32 | 24/18614 | 0.040478 | 0.060237 | 0.018213 | ERBB2 | 1 |
| BP | GO:0014850 | response to muscle activity | 1/32 | 24/18614 | 0.040478 | 0.060237 | 0.018213 | HIF1A | 1 |
| BP | GO:0032515 | negative regulation of phosphoprotein phosphatase activity | 1/32 | 24/18614 | 0.040478 | 0.060237 | 0.018213 | GSK3B | 1 |
| BP | GO:0034695 | response to prostaglandin E | 1/32 | 24/18614 | 0.040478 | 0.060237 | 0.018213 | AKT1 | 1 |
| BP | GO:0035162 | embryonic hemopoiesis | 1/32 | 24/18614 | 0.040478 | 0.060237 | 0.018213 | HIF1A | 1 |
| BP | GO:0035357 | peroxisome proliferator activated receptor signaling pathway | 1/32 | 24/18614 | 0.040478 | 0.060237 | 0.018213 | PPARG | 1 |
| BP | GO:0043369 | CD4-positive or CD8-positive, alpha-beta T cell lineage commitment | 1/32 | 24/18614 | 0.040478 | 0.060237 | 0.018213 | BCL2 | 1 |
| BP | GO:0044030 | regulation of DNA methylation | 1/32 | 24/18614 | 0.040478 | 0.060237 | 0.018213 | MYC | 1 |
| BP | GO:0045932 | negative regulation of muscle contraction | 1/32 | 24/18614 | 0.040478 | 0.060237 | 0.018213 | PTGS2 | 1 |
| BP | GO:0048011 | neurotrophin TRK receptor signaling pathway | 1/32 | 24/18614 | 0.040478 | 0.060237 | 0.018213 | CASP3 | 1 |
| BP | GO:0090335 | regulation of brown fat cell differentiation | 1/32 | 24/18614 | 0.040478 | 0.060237 | 0.018213 | PTGS2 | 1 |
| BP | GO:1900273 | positive regulation of long-term synaptic potentiation | 1/32 | 24/18614 | 0.040478 | 0.060237 | 0.018213 | APP | 1 |
| BP | GO:1900543 | negative regulation of purine nucleotide metabolic process | 1/32 | 24/18614 | 0.040478 | 0.060237 | 0.018213 | TP53 | 1 |
| BP | GO:1901863 | positive regulation of muscle tissue development | 1/32 | 24/18614 | 0.040478 | 0.060237 | 0.018213 | BCL2 | 1 |
| BP | GO:0051896 | regulation of protein kinase B signaling | 2/32 | 186/18614 | 0.040491 | 0.060237 | 0.018213 | EGFR/EGF | 2 |
| BP | GO:1903169 | regulation of calcium ion transmembrane transport | 2/32 | 186/18614 | 0.040491 | 0.060237 | 0.018213 | BCL2/CAV1 | 2 |
| BP | GO:0006006 | glucose metabolic process | 2/32 | 187/18614 | 0.040886 | 0.060691 | 0.01835 | AKT1/TP53 | 2 |
| BP | GO:0019722 | calcium-mediated signaling | 2/32 | 187/18614 | 0.040886 | 0.060691 | 0.01835 | GSK3B/CXCL8 | 2 |
| BP | GO:0032200 | telomere organization | 2/32 | 187/18614 | 0.040886 | 0.060691 | 0.01835 | MYC/MAPK1 | 2 |
| BP | GO:0050728 | negative regulation of inflammatory response | 2/32 | 187/18614 | 0.040886 | 0.060691 | 0.01835 | PPARG/IL10 | 2 |
| BP | GO:0000075 | cell cycle checkpoint signaling | 2/32 | 188/18614 | 0.041282 | 0.061245 | 0.018518 | TP53/CCND1 | 2 |
| BP | GO:0000280 | nuclear division | 3/32 | 450/18614 | 0.041476 | 0.0615 | 0.018595 | IL1B/EGF/IL1A | 3 |
| BP | GO:0010975 | regulation of neuron projection development | 3/32 | 452/18614 | 0.041936 | 0.061727 | 0.018664 | AKT1/GSK3B/NFE2L2 | 3 |
| BP | GO:0002822 | regulation of adaptive immune response based on somatic recombination of immune receptors built from immunoglobulin superfamily domains | 2/32 | 190/18614 | 0.042078 | 0.061727 | 0.018664 | IL1B/IL10 | 2 |
| BP | GO:0006007 | glucose catabolic process | 1/32 | 25/18614 | 0.04213 | 0.061727 | 0.018664 | TP53 | 1 |
| BP | GO:0006582 | melanin metabolic process | 1/32 | 25/18614 | 0.04213 | 0.061727 | 0.018664 | BCL2 | 1 |
| BP | GO:0030318 | melanocyte differentiation | 1/32 | 25/18614 | 0.04213 | 0.061727 | 0.018664 | BCL2 | 1 |
| BP | GO:0031579 | membrane raft organization | 1/32 | 25/18614 | 0.04213 | 0.061727 | 0.018664 | CAV1 | 1 |
| BP | GO:0033233 | regulation of protein sumoylation | 1/32 | 25/18614 | 0.04213 | 0.061727 | 0.018664 | RELA | 1 |
| BP | GO:0034453 | microtubule anchoring | 1/32 | 25/18614 | 0.04213 | 0.061727 | 0.018664 | GSK3B | 1 |
| BP | GO:0043153 | entrainment of circadian clock by photoperiod | 1/32 | 25/18614 | 0.04213 | 0.061727 | 0.018664 | TP53 | 1 |
| BP | GO:0045980 | negative regulation of nucleotide metabolic process | 1/32 | 25/18614 | 0.04213 | 0.061727 | 0.018664 | TP53 | 1 |
| BP | GO:0046697 | decidualization | 1/32 | 25/18614 | 0.04213 | 0.061727 | 0.018664 | PTGS2 | 1 |
| BP | GO:0046885 | regulation of hormone biosynthetic process | 1/32 | 25/18614 | 0.04213 | 0.061727 | 0.018664 | HIF1A | 1 |
| BP | GO:0050927 | positive regulation of positive chemotaxis | 1/32 | 25/18614 | 0.04213 | 0.061727 | 0.018664 | CXCL8 | 1 |
| BP | GO:0070423 | nucleotide-binding oligomerization domain containing signaling pathway | 1/32 | 25/18614 | 0.04213 | 0.061727 | 0.018664 | RELA | 1 |
| BP | GO:0071450 | cellular response to oxygen radical | 1/32 | 25/18614 | 0.04213 | 0.061727 | 0.018664 | NFE2L2 | 1 |
| BP | GO:0071451 | cellular response to superoxide | 1/32 | 25/18614 | 0.04213 | 0.061727 | 0.018664 | NFE2L2 | 1 |
| BP | GO:0086064 | cell communication by electrical coupling involved in cardiac conduction | 1/32 | 25/18614 | 0.04213 | 0.061727 | 0.018664 | CAV1 | 1 |
| BP | GO:0090023 | positive regulation of neutrophil chemotaxis | 1/32 | 25/18614 | 0.04213 | 0.061727 | 0.018664 | CXCL8 | 1 |
| BP | GO:0099633 | protein localization to postsynaptic specialization membrane | 1/32 | 25/18614 | 0.04213 | 0.061727 | 0.018664 | ERBB2 | 1 |
| BP | GO:0099645 | neurotransmitter receptor localization to postsynaptic specialization membrane | 1/32 | 25/18614 | 0.04213 | 0.061727 | 0.018664 | ERBB2 | 1 |
| BP | GO:1904996 | positive regulation of leukocyte adhesion to vascular endothelial cell | 1/32 | 25/18614 | 0.04213 | 0.061727 | 0.018664 | RELA | 1 |
| BP | GO:2000679 | positive regulation of transcription regulatory region DNA binding | 1/32 | 25/18614 | 0.04213 | 0.061727 | 0.018664 | IFNG | 1 |
| BP | GO:0030308 | negative regulation of cell growth | 2/32 | 191/18614 | 0.042479 | 0.062204 | 0.018808 | TP53/BCL2 | 2 |
| BP | GO:0009755 | hormone-mediated signaling pathway | 2/32 | 192/18614 | 0.042881 | 0.062759 | 0.018976 | ESR1/PPARG | 2 |
| BP | GO:0009749 | response to glucose | 2/32 | 194/18614 | 0.043689 | 0.063457 | 0.019187 | CASP3/HIF1A | 2 |
| BP | GO:0017157 | regulation of exocytosis | 2/32 | 194/18614 | 0.043689 | 0.063457 | 0.019187 | IFNG/HMOX1 | 2 |
| BP | GO:0002026 | regulation of the force of heart contraction | 1/32 | 26/18614 | 0.043779 | 0.063457 | 0.019187 | CAV1 | 1 |
| BP | GO:0002726 | positive regulation of T cell cytokine production | 1/32 | 26/18614 | 0.043779 | 0.063457 | 0.019187 | IL1B | 1 |
| BP | GO:0031639 | plasminogen activation | 1/32 | 26/18614 | 0.043779 | 0.063457 | 0.019187 | SERPINE1 | 1 |
| BP | GO:0035235 | ionotropic glutamate receptor signaling pathway | 1/32 | 26/18614 | 0.043779 | 0.063457 | 0.019187 | APP | 1 |
| BP | GO:0035872 | nucleotide-binding domain, leucine rich repeat containing receptor signaling pathway | 1/32 | 26/18614 | 0.043779 | 0.063457 | 0.019187 | RELA | 1 |
| BP | GO:0046426 | negative regulation of receptor signaling pathway via JAK-STAT | 1/32 | 26/18614 | 0.043779 | 0.063457 | 0.019187 | CAV1 | 1 |
| BP | GO:0050857 | positive regulation of antigen receptor-mediated signaling pathway | 1/32 | 26/18614 | 0.043779 | 0.063457 | 0.019187 | RELA | 1 |
| BP | GO:0050926 | regulation of positive chemotaxis | 1/32 | 26/18614 | 0.043779 | 0.063457 | 0.019187 | CXCL8 | 1 |
| BP | GO:0060343 | trabecula formation | 1/32 | 26/18614 | 0.043779 | 0.063457 | 0.019187 | MMP2 | 1 |
| BP | GO:0060575 | intestinal epithelial cell differentiation | 1/32 | 26/18614 | 0.043779 | 0.063457 | 0.019187 | HIF1A | 1 |
| BP | GO:1901028 | regulation of mitochondrial outer membrane permeabilization involved in apoptotic signaling pathway | 1/32 | 26/18614 | 0.043779 | 0.063457 | 0.019187 | GSK3B | 1 |
| BP | GO:1901623 | regulation of lymphocyte chemotaxis | 1/32 | 26/18614 | 0.043779 | 0.063457 | 0.019187 | CCL2 | 1 |
| BP | GO:1903306 | negative regulation of regulated secretory pathway | 1/32 | 26/18614 | 0.043779 | 0.063457 | 0.019187 | HMOX1 | 1 |
| BP | GO:1904353 | regulation of telomere capping | 1/32 | 26/18614 | 0.043779 | 0.063457 | 0.019187 | MAPK1 | 1 |
| BP | GO:1905523 | positive regulation of macrophage migration | 1/32 | 26/18614 | 0.043779 | 0.063457 | 0.019187 | MAPK1 | 1 |
| BP | GO:2000647 | negative regulation of stem cell proliferation | 1/32 | 26/18614 | 0.043779 | 0.063457 | 0.019187 | TP53 | 1 |
| BP | GO:0120032 | regulation of plasma membrane bounded cell projection assembly | 2/32 | 195/18614 | 0.044095 | 0.063881 | 0.019315 | GSK3B/CAV1 | 2 |
| BP | GO:0009259 | ribonucleotide metabolic process | 3/32 | 464/18614 | 0.044754 | 0.064801 | 0.019593 | HIF1A/IFNG/APP | 3 |
| BP | GO:0001906 | cell killing | 2/32 | 197/18614 | 0.044911 | 0.06496 | 0.019641 | IFNG/ICAM1 | 2 |
| BP | GO:0060491 | regulation of cell projection assembly | 2/32 | 197/18614 | 0.044911 | 0.06496 | 0.019641 | GSK3B/CAV1 | 2 |
| BP | GO:0001963 | synaptic transmission, dopaminergic | 1/32 | 27/18614 | 0.045425 | 0.065044 | 0.019667 | PTGS2 | 1 |
| BP | GO:0002092 | positive regulation of receptor internalization | 1/32 | 27/18614 | 0.045425 | 0.065044 | 0.019667 | EGF | 1 |
| BP | GO:0009651 | response to salt stress | 1/32 | 27/18614 | 0.045425 | 0.065044 | 0.019667 | TP53 | 1 |
| BP | GO:0032682 | negative regulation of chemokine production | 1/32 | 27/18614 | 0.045425 | 0.065044 | 0.019667 | IL10 | 1 |
| BP | GO:0033081 | regulation of T cell differentiation in thymus | 1/32 | 27/18614 | 0.045425 | 0.065044 | 0.019667 | ERBB2 | 1 |
| BP | GO:0034643 | establishment of mitochondrion localization, microtubule-mediated | 1/32 | 27/18614 | 0.045425 | 0.065044 | 0.019667 | HIF1A | 1 |
| BP | GO:0036010 | protein localization to endosome | 1/32 | 27/18614 | 0.045425 | 0.065044 | 0.019667 | EGF | 1 |
| BP | GO:0042501 | serine phosphorylation of STAT protein | 1/32 | 27/18614 | 0.045425 | 0.065044 | 0.019667 | IFNG | 1 |
| BP | GO:0042730 | fibrinolysis | 1/32 | 27/18614 | 0.045425 | 0.065044 | 0.019667 | SERPINE1 | 1 |
| BP | GO:0042832 | defense response to protozoan | 1/32 | 27/18614 | 0.045425 | 0.065044 | 0.019667 | IL10 | 1 |
| BP | GO:0045815 | transcription initiation-coupled chromatin remodeling | 1/32 | 27/18614 | 0.045425 | 0.065044 | 0.019667 | TP53 | 1 |
| BP | GO:0047497 | mitochondrion transport along microtubule | 1/32 | 27/18614 | 0.045425 | 0.065044 | 0.019667 | HIF1A | 1 |
| BP | GO:0048641 | regulation of skeletal muscle tissue development | 1/32 | 27/18614 | 0.045425 | 0.065044 | 0.019667 | BCL2 | 1 |
| BP | GO:0048668 | collateral sprouting | 1/32 | 27/18614 | 0.045425 | 0.065044 | 0.019667 | APP | 1 |
| BP | GO:0050996 | positive regulation of lipid catabolic process | 1/32 | 27/18614 | 0.045425 | 0.065044 | 0.019667 | IL1B | 1 |
| BP | GO:0060547 | negative regulation of necrotic cell death | 1/32 | 27/18614 | 0.045425 | 0.065044 | 0.019667 | CAV1 | 1 |
| BP | GO:0060669 | embryonic placenta morphogenesis | 1/32 | 27/18614 | 0.045425 | 0.065044 | 0.019667 | IL10 | 1 |
| BP | GO:0071549 | cellular response to dexamethasone stimulus | 1/32 | 27/18614 | 0.045425 | 0.065044 | 0.019667 | CASP9 | 1 |
| BP | GO:1904753 | negative regulation of vascular associated smooth muscle cell migration | 1/32 | 27/18614 | 0.045425 | 0.065044 | 0.019667 | NFE2L2 | 1 |
| BP | GO:0097553 | calcium ion transmembrane import into cytosol | 2/32 | 201/18614 | 0.046562 | 0.066636 | 0.020148 | BCL2/CAV1 | 2 |
| BP | GO:0019693 | ribose phosphate metabolic process | 3/32 | 473/18614 | 0.046928 | 0.066797 | 0.020197 | HIF1A/IFNG/APP | 3 |
| BP | GO:0000303 | response to superoxide | 1/32 | 28/18614 | 0.047068 | 0.066797 | 0.020197 | NFE2L2 | 1 |
| BP | GO:0003401 | axis elongation | 1/32 | 28/18614 | 0.047068 | 0.066797 | 0.020197 | ESR1 | 1 |
| BP | GO:0010758 | regulation of macrophage chemotaxis | 1/32 | 28/18614 | 0.047068 | 0.066797 | 0.020197 | MAPK1 | 1 |
| BP | GO:0022011 | myelination in peripheral nervous system | 1/32 | 28/18614 | 0.047068 | 0.066797 | 0.020197 | AKT1 | 1 |
| BP | GO:0032104 | regulation of response to extracellular stimulus | 1/32 | 28/18614 | 0.047068 | 0.066797 | 0.020197 | BCL2 | 1 |
| BP | GO:0032107 | regulation of response to nutrient levels | 1/32 | 28/18614 | 0.047068 | 0.066797 | 0.020197 | BCL2 | 1 |
| BP | GO:0032292 | peripheral nervous system axon ensheathment | 1/32 | 28/18614 | 0.047068 | 0.066797 | 0.020197 | AKT1 | 1 |
| BP | GO:0036037 | CD8-positive, alpha-beta T cell activation | 1/32 | 28/18614 | 0.047068 | 0.066797 | 0.020197 | BCL2 | 1 |
| BP | GO:0042059 | negative regulation of epidermal growth factor receptor signaling pathway | 1/32 | 28/18614 | 0.047068 | 0.066797 | 0.020197 | EGFR | 1 |
| BP | GO:0045116 | protein neddylation | 1/32 | 28/18614 | 0.047068 | 0.066797 | 0.020197 | HIF1A | 1 |
| BP | GO:0045724 | positive regulation of cilium assembly | 1/32 | 28/18614 | 0.047068 | 0.066797 | 0.020197 | GSK3B | 1 |
| BP | GO:0051156 | glucose 6-phosphate metabolic process | 1/32 | 28/18614 | 0.047068 | 0.066797 | 0.020197 | TP53 | 1 |
| BP | GO:0071624 | positive regulation of granulocyte chemotaxis | 1/32 | 28/18614 | 0.047068 | 0.066797 | 0.020197 | CXCL8 | 1 |
| BP | GO:0090314 | positive regulation of protein targeting to membrane | 1/32 | 28/18614 | 0.047068 | 0.066797 | 0.020197 | ERBB2 | 1 |
| BP | GO:1903672 | positive regulation of sprouting angiogenesis | 1/32 | 28/18614 | 0.047068 | 0.066797 | 0.020197 | IL10 | 1 |
| BP | GO:1902075 | cellular response to salt | 2/32 | 204/18614 | 0.047814 | 0.06782 | 0.020506 | PTGS2/FOS | 2 |
| BP | GO:0002819 | regulation of adaptive immune response | 2/32 | 205/18614 | 0.048234 | 0.068374 | 0.020674 | IL1B/IL10 | 2 |
| BP | GO:2000241 | regulation of reproductive process | 2/32 | 206/18614 | 0.048656 | 0.068374 | 0.020674 | ESR1/IL1A | 2 |
| BP | GO:0000305 | response to oxygen radical | 1/32 | 29/18614 | 0.048709 | 0.068374 | 0.020674 | NFE2L2 | 1 |
| BP | GO:0001562 | response to protozoan | 1/32 | 29/18614 | 0.048709 | 0.068374 | 0.020674 | IL10 | 1 |
| BP | GO:0001773 | myeloid dendritic cell activation | 1/32 | 29/18614 | 0.048709 | 0.068374 | 0.020674 | IL10 | 1 |
| BP | GO:0002052 | positive regulation of neuroblast proliferation | 1/32 | 29/18614 | 0.048709 | 0.068374 | 0.020674 | HIF1A | 1 |
| BP | GO:0007263 | nitric oxide mediated signal transduction | 1/32 | 29/18614 | 0.048709 | 0.068374 | 0.020674 | EGFR | 1 |
| BP | GO:0009648 | photoperiodism | 1/32 | 29/18614 | 0.048709 | 0.068374 | 0.020674 | TP53 | 1 |
| BP | GO:0010955 | negative regulation of protein processing | 1/32 | 29/18614 | 0.048709 | 0.068374 | 0.020674 | SERPINE1 | 1 |
| BP | GO:0030431 | sleep | 1/32 | 29/18614 | 0.048709 | 0.068374 | 0.020674 | FOS | 1 |
| BP | GO:0030878 | thyroid gland development | 1/32 | 29/18614 | 0.048709 | 0.068374 | 0.020674 | MAPK1 | 1 |
| BP | GO:0032607 | interferon-alpha production | 1/32 | 29/18614 | 0.048709 | 0.068374 | 0.020674 | IL10 | 1 |
| BP | GO:0032647 | regulation of interferon-alpha production | 1/32 | 29/18614 | 0.048709 | 0.068374 | 0.020674 | IL10 | 1 |
| BP | GO:0048245 | eosinophil chemotaxis | 1/32 | 29/18614 | 0.048709 | 0.068374 | 0.020674 | CCL2 | 1 |
| BP | GO:0090025 | regulation of monocyte chemotaxis | 1/32 | 29/18614 | 0.048709 | 0.068374 | 0.020674 | SERPINE1 | 1 |
| BP | GO:1900017 | positive regulation of cytokine production involved in inflammatory response | 1/32 | 29/18614 | 0.048709 | 0.068374 | 0.020674 | HIF1A | 1 |
| BP | GO:1900117 | regulation of execution phase of apoptosis | 1/32 | 29/18614 | 0.048709 | 0.068374 | 0.020674 | TP53 | 1 |
| BP | GO:1901017 | negative regulation of potassium ion transmembrane transporter activity | 1/32 | 29/18614 | 0.048709 | 0.068374 | 0.020674 | CAV1 | 1 |
| BP | GO:1903318 | negative regulation of protein maturation | 1/32 | 29/18614 | 0.048709 | 0.068374 | 0.020674 | SERPINE1 | 1 |
| BP | GO:1903649 | regulation of cytoplasmic transport | 1/32 | 29/18614 | 0.048709 | 0.068374 | 0.020674 | MAPK1 | 1 |
| BP | GO:0071383 | cellular response to steroid hormone stimulus | 2/32 | 209/18614 | 0.049929 | 0.069998 | 0.021165 | ESR1/CASP9 | 2 |
| CC | GO:0045121 | membrane raft | 7/32 | 323/19518 | 7.51E-07 | 5.64E-05 | 3.63E-05 | PTGS2/CASP3/EGFR/HMOX1/APP/MAPK1/CAV1 | 7 |
| CC | GO:0098857 | membrane microdomain | 7/32 | 324/19518 | 7.67E-07 | 5.64E-05 | 3.63E-05 | PTGS2/CASP3/EGFR/HMOX1/APP/MAPK1/CAV1 | 7 |
| CC | GO:0005901 | caveola | 4/32 | 83/19518 | 9.98E-06 | 0.000489 | 0.000315 | PTGS2/HMOX1/MAPK1/CAV1 | 4 |
| CC | GO:0044853 | plasma membrane raft | 4/32 | 114/19518 | 3.50E-05 | 0.001286 | 0.000829 | PTGS2/HMOX1/MAPK1/CAV1 | 4 |
| CC | GO:0090575 | RNA polymerase II transcription regulator complex | 5/32 | 254/19518 | 5.42E-05 | 0.001594 | 0.001027 | HIF1A/FOS/MYC/PPARG/NFE2L2 | 5 |
| CC | GO:0031983 | vesicle lumen | 5/32 | 327/19518 | 0.000178 | 0.00375 | 0.002417 | EGFR/EGF/APP/MAPK1/SERPINE1 | 5 |
| CC | GO:0031093 | platelet alpha granule lumen | 3/32 | 67/19518 | 0.000179 | 0.00375 | 0.002417 | EGF/APP/SERPINE1 | 3 |
| CC | GO:0017053 | transcription repressor complex | 3/32 | 78/19518 | 0.00028 | 0.005147 | 0.003317 | TP53/MYC/CCND1 | 3 |
| CC | GO:0031091 | platelet alpha granule | 3/32 | 91/19518 | 0.000441 | 0.007202 | 0.004641 | EGF/APP/SERPINE1 | 3 |
| CC | GO:0005769 | early endosome | 5/32 | 423/19518 | 0.00058 | 0.008529 | 0.005497 | EGFR/ERBB2/APP/MAPK1/CAV1 | 5 |
| CC | GO:0005635 | nuclear envelope | 5/32 | 490/19518 | 0.001124 | 0.015015 | 0.009677 | PTGS2/EGFR/BCL2/CCND1/APP | 5 |
| CC | GO:0031965 | nuclear membrane | 4/32 | 308/19518 | 0.001543 | 0.018905 | 0.012184 | PTGS2/EGFR/BCL2/CCND1 | 4 |
| CC | GO:0034774 | secretory granule lumen | 4/32 | 322/19518 | 0.001816 | 0.019722 | 0.01271 | EGF/APP/MAPK1/SERPINE1 | 4 |
| CC | GO:0060205 | cytoplasmic vesicle lumen | 4/32 | 325/19518 | 0.001878 | 0.019722 | 0.01271 | EGF/APP/MAPK1/SERPINE1 | 4 |
| CC | GO:0043209 | myelin sheath | 2/32 | 49/19518 | 0.002919 | 0.028603 | 0.018434 | BCL2/ERBB2 | 2 |
| CC | GO:0030666 | endocytic vesicle membrane | 3/32 | 196/19518 | 0.003992 | 0.035269 | 0.02273 | EGFR/EGF/CAV1 | 3 |
| CC | GO:0000791 | euchromatin | 2/32 | 60/19518 | 0.004344 | 0.035269 | 0.02273 | ESR1/HIF1A | 2 |
| CC | GO:0062023 | collagen-containing extracellular matrix | 4/32 | 415/19518 | 0.004523 | 0.035269 | 0.02273 | MMP9/ICAM1/MMP2/SERPINE1 | 4 |
| CC | GO:0005925 | focal adhesion | 4/32 | 422/19518 | 0.004799 | 0.035269 | 0.02273 | EGFR/ICAM1/MAPK1/CAV1 | 4 |
| CC | GO:0031252 | cell leading edge | 4/32 | 422/19518 | 0.004799 | 0.035269 | 0.02273 | AKT1/EGFR/ERBB2/APP | 4 |
| CC | GO:0030055 | cell-substrate junction | 4/32 | 432/19518 | 0.005212 | 0.036481 | 0.023511 | EGFR/ICAM1/MAPK1/CAV1 | 4 |
| CC | GO:0032993 | protein-DNA complex | 3/32 | 225/19518 | 0.00586 | 0.03868 | 0.024928 | ESR1/FOS/NFE2L2 | 3 |
| CC | GO:0030669 | clathrin-coated endocytic vesicle membrane | 2/32 | 73/19518 | 0.006365 | 0.03868 | 0.024928 | EGFR/EGF | 2 |
| CC | GO:0031594 | neuromuscular junction | 2/32 | 73/19518 | 0.006365 | 0.03868 | 0.024928 | ERBB2/APP | 2 |
| CC | GO:0031968 | organelle outer membrane | 3/32 | 236/19518 | 0.006684 | 0.03868 | 0.024928 | PTGS2/BCL2/HMOX1 | 3 |
| CC | GO:0019867 | outer membrane | 3/32 | 238/19518 | 0.006841 | 0.03868 | 0.024928 | PTGS2/BCL2/HMOX1 | 3 |
| CC | GO:0045334 | clathrin-coated endocytic vesicle | 2/32 | 92/19518 | 0.009944 | 0.054138 | 0.03489 | EGFR/EGF | 2 |
| CC | GO:0032587 | ruffle membrane | 2/32 | 101/19518 | 0.011887 | 0.062406 | 0.040219 | EGFR/ERBB2 | 2 |
| CC | GO:0005788 | endoplasmic reticulum lumen | 3/32 | 312/19518 | 0.014247 | 0.069809 | 0.044989 | PTGS2/APP/MAPK1 | 3 |
| CC | GO:0030135 | coated vesicle | 3/32 | 312/19518 | 0.014247 | 0.069809 | 0.044989 | EGFR/EGF/APP | 3 |
| CC | GO:0005641 | nuclear envelope lumen | 1/32 | 10/19518 | 0.016278 | 0.071085 | 0.045812 | APP | 1 |
| CC | GO:0008303 | caspase complex | 1/32 | 10/19518 | 0.016278 | 0.071085 | 0.045812 | CASP9 | 1 |
| CC | GO:0097136 | Bcl-2 family protein complex | 1/32 | 10/19518 | 0.016278 | 0.071085 | 0.045812 | BCL2 | 1 |
| CC | GO:1904813 | ficolin-1-rich granule lumen | 2/32 | 124/19518 | 0.017536 | 0.071085 | 0.045812 | MMP9/MAPK1 | 2 |
| CC | GO:0030877 | beta-catenin destruction complex | 1/32 | 11/19518 | 0.017892 | 0.071085 | 0.045812 | GSK3B | 1 |
| CC | GO:0097550 | transcription preinitiation complex | 1/32 | 11/19518 | 0.017892 | 0.071085 | 0.045812 | ESR1 | 1 |
| CC | GO:1904090 | peptidase inhibitor complex | 1/32 | 11/19518 | 0.017892 | 0.071085 | 0.045812 | SERPINE1 | 1 |
| CC | GO:0030139 | endocytic vesicle | 3/32 | 347/19518 | 0.018877 | 0.073022 | 0.047061 | EGFR/EGF/CAV1 | 3 |
| CC | GO:0030665 | clathrin-coated vesicle membrane | 2/32 | 136/19518 | 0.020855 | 0.075693 | 0.048782 | EGFR/EGF | 2 |
| CC | GO:0035253 | ciliary rootlet | 1/32 | 13/19518 | 0.021112 | 0.075693 | 0.048782 | APP | 1 |
| CC | GO:1990909 | Wnt signalosome | 1/32 | 13/19518 | 0.021112 | 0.075693 | 0.048782 | GSK3B | 1 |
| CC | GO:0090571 | RNA polymerase II transcription repressor complex | 1/32 | 15/19518 | 0.024321 | 0.085124 | 0.05486 | MYC | 1 |
| CC | GO:0098978 | glutamatergic synapse | 3/32 | 410/19518 | 0.029094 | 0.097274 | 0.06269 | AKT1/GSK3B/RELA | 3 |
| CC | GO:0031143 | pseudopodium | 1/32 | 18/19518 | 0.029116 | 0.097274 | 0.06269 | MAPK1 | 1 |
| CC | GO:0097449 | astrocyte projection | 1/32 | 19/19518 | 0.030709 | 0.099472 | 0.064107 | APP | 1 |
| CC | GO:0005819 | spindle | 3/32 | 421/19518 | 0.031127 | 0.099472 | 0.064107 | AKT1/APP/MAPK1 | 3 |
| CC | GO:0031256 | leading edge membrane | 2/32 | 180/19518 | 0.035006 | 0.108295 | 0.069793 | EGFR/ERBB2 | 2 |
| CC | GO:0001726 | ruffle | 2/32 | 181/19518 | 0.035362 | 0.108295 | 0.069793 | EGFR/ERBB2 | 2 |
| CC | GO:0101002 | ficolin-1-rich granule | 2/32 | 185/19518 | 0.036798 | 0.110321 | 0.071098 | MMP9/MAPK1 | 2 |
| CC | GO:0031901 | early endosome membrane | 2/32 | 187/19518 | 0.037524 | 0.110321 | 0.071098 | EGFR/CAV1 | 2 |
| CC | GO:0046930 | pore complex | 1/32 | 26/19518 | 0.041791 | 0.119671 | 0.077124 | BCL2 | 1 |
| CC | GO:0030027 | lamellipodium | 2/32 | 202/19518 | 0.043147 | 0.119671 | 0.077124 | AKT1/APP | 2 |
| CC | GO:0030662 | coated vesicle membrane | 2/32 | 202/19518 | 0.043147 | 0.119671 | 0.077124 | EGFR/EGF | 2 |
| CC | GO:0098992 | neuronal dense core vesicle | 1/32 | 28/19518 | 0.044935 | 0.121553 | 0.078337 | APP | 1 |
| CC | GO:0005741 | mitochondrial outer membrane | 2/32 | 208/19518 | 0.045479 | 0.121553 | 0.078337 | BCL2/HMOX1 | 2 |
| CC | GO:0005640 | nuclear outer membrane | 1/32 | 30/19518 | 0.048069 | 0.124456 | 0.080208 | PTGS2 | 1 |
| CC | GO:0030136 | clathrin-coated vesicle | 2/32 | 215/19518 | 0.048259 | 0.124456 | 0.080208 | EGFR/EGF | 2 |
| MF | GO:0001221 | transcription coregulator binding | 7/32 | 111/18369 | 7.22E-10 | 1.38E-07 | 5.24E-08 | ESR1/HIF1A/FOS/MYC/PPARG/RELA/NFE2L2 | 7 |
| MF | GO:0140297 | DNA-binding transcription factor binding | 10/32 | 478/18369 | 5.00E-09 | 4.78E-07 | 1.82E-07 | TP53/ESR1/BCL2/HIF1A/FOS/MYC/PPARG/GSK3B/RELA/NFE2L2 | 10 |
| MF | GO:0061629 | RNA polymerase II-specific DNA-binding transcription factor binding | 8/32 | 348/18369 | 1.08E-07 | 6.90E-06 | 2.62E-06 | TP53/ESR1/HIF1A/FOS/PPARG/GSK3B/RELA/NFE2L2 | 8 |
| MF | GO:0005126 | cytokine receptor binding | 7/32 | 270/18369 | 3.37E-07 | 1.61E-05 | 6.12E-06 | IL1B/CASP3/IL10/CCL2/IFNG/CXCL8/IL1A | 7 |
| MF | GO:0001046 | core promoter sequence-specific DNA binding | 4/32 | 43/18369 | 8.92E-07 | 3.41E-05 | 1.30E-05 | TP53/FOS/MYC/RELA | 4 |
| MF | GO:0005125 | cytokine activity | 6/32 | 235/18369 | 2.82E-06 | 8.98E-05 | 3.42E-05 | IL1B/IL10/CCL2/IFNG/CXCL8/IL1A | 6 |
| MF | GO:0070851 | growth factor receptor binding | 5/32 | 137/18369 | 3.67E-06 | 9.09E-05 | 3.46E-05 | IL1B/EGF/IL10/IL1A/APP | 5 |
| MF | GO:0002020 | protease binding | 5/32 | 138/18369 | 3.81E-06 | 9.09E-05 | 3.46E-05 | TP53/CASP3/BCL2/GSK3B/SERPINE1 | 5 |
| MF | GO:0016004 | phospholipase activator activity | 3/32 | 22/18369 | 7.23E-06 | 0.000153 | 5.84E-05 | CASP3/EGFR/EGF | 3 |
| MF | GO:0060229 | lipase activator activity | 3/32 | 25/18369 | 1.08E-05 | 0.0002 | 7.62E-05 | CASP3/EGFR/EGF | 3 |
| MF | GO:0031625 | ubiquitin protein ligase binding | 6/32 | 301/18369 | 1.17E-05 | 0.0002 | 7.62E-05 | TP53/EGFR/BCL2/HIF1A/GSK3B/RELA | 6 |
| MF | GO:0001228 | DNA-binding transcription activator activity, RNA polymerase II-specific | 7/32 | 468/18369 | 1.29E-05 | 0.0002 | 7.62E-05 | TP53/ESR1/HIF1A/FOS/MYC/RELA/NFE2L2 | 7 |
| MF | GO:0001216 | DNA-binding transcription activator activity | 7/32 | 472/18369 | 1.36E-05 | 0.0002 | 7.62E-05 | TP53/ESR1/HIF1A/FOS/MYC/RELA/NFE2L2 | 7 |
| MF | GO:0044389 | ubiquitin-like protein ligase binding | 6/32 | 320/18369 | 1.65E-05 | 0.000213 | 8.08E-05 | TP53/EGFR/BCL2/HIF1A/GSK3B/RELA | 6 |
| MF | GO:0019902 | phosphatase binding | 5/32 | 187/18369 | 1.67E-05 | 0.000213 | 8.08E-05 | TP53/EGFR/BCL2/ERBB2/MAPK1 | 5 |
| MF | GO:0048018 | receptor ligand activity | 7/32 | 497/18369 | 1.90E-05 | 0.000227 | 8.64E-05 | IL1B/EGF/IL10/CCL2/IFNG/CXCL8/IL1A | 7 |
| MF | GO:0001223 | transcription coactivator binding | 3/32 | 43/18369 | 5.65E-05 | 0.000635 | 0.000241 | ESR1/HIF1A/RELA | 3 |
| MF | GO:0042826 | histone deacetylase binding | 4/32 | 126/18369 | 6.54E-05 | 0.000694 | 0.000264 | TP53/HIF1A/CCND1/RELA | 4 |
| MF | GO:0070888 | E-box binding | 3/32 | 48/18369 | 7.88E-05 | 0.000792 | 0.000301 | HIF1A/MYC/PPARG | 3 |
| MF | GO:0019903 | protein phosphatase binding | 4/32 | 140/18369 | 9.85E-05 | 0.00094 | 0.000358 | TP53/EGFR/BCL2/ERBB2 | 4 |
| MF | GO:0140296 | general transcription initiation factor binding | 3/32 | 53/18369 | 0.000106 | 0.000964 | 0.000367 | TP53/ESR1/RELA | 3 |
| MF | GO:0097153 | cysteine-type endopeptidase activity involved in apoptotic process | 2/32 | 12/18369 | 0.000192 | 0.001666 | 0.000634 | CASP3/CASP9 | 2 |
| MF | GO:0002039 | p53 binding | 3/32 | 67/18369 | 0.000213 | 0.001771 | 0.000673 | TP53/HIF1A/GSK3B | 3 |
| MF | GO:0046982 | protein heterodimerization activity | 5/32 | 343/18369 | 0.000293 | 0.002334 | 0.000888 | TP53/BCL2/HIF1A/ERBB2/CAV1 | 5 |
| MF | GO:0030297 | transmembrane receptor protein tyrosine kinase activator activity | 2/32 | 16/18369 | 0.000347 | 0.002655 | 0.00101 | EGFR/EGF | 2 |
| MF | GO:0005149 | interleukin-1 receptor binding | 2/32 | 17/18369 | 0.000393 | 0.00289 | 0.001099 | IL1B/IL1A | 2 |
| MF | GO:0051117 | ATPase binding | 3/32 | 86/18369 | 0.000445 | 0.003151 | 0.001198 | ESR1/EGFR/CAV1 | 3 |
| MF | GO:0000979 | RNA polymerase II core promoter sequence-specific DNA binding | 2/32 | 19/18369 | 0.000494 | 0.003367 | 0.00128 | FOS/RELA | 2 |
| MF | GO:0070412 | R-SMAD binding | 2/32 | 24/18369 | 0.000792 | 0.005218 | 0.001984 | FOS/PPARG | 2 |
| MF | GO:0001091 | RNA polymerase II general transcription initiation factor binding | 2/32 | 26/18369 | 0.000931 | 0.005927 | 0.002254 | TP53/ESR1 | 2 |
| MF | GO:0051721 | protein phosphatase 2A binding | 2/32 | 28/18369 | 0.00108 | 0.006656 | 0.002531 | TP53/BCL2 | 2 |
| MF | GO:0051059 | NF-kappaB binding | 2/32 | 31/18369 | 0.001325 | 0.007907 | 0.003007 | GSK3B/RELA | 2 |
| MF | GO:0030296 | protein tyrosine kinase activator activity | 2/32 | 34/18369 | 0.001593 | 0.008949 | 0.003403 | EGFR/EGF | 2 |
| MF | GO:0071889 | 14-3-3 protein binding | 2/32 | 34/18369 | 0.001593 | 0.008949 | 0.003403 | AKT1/TP53 | 2 |
| MF | GO:0016922 | nuclear receptor binding | 3/32 | 138/18369 | 0.001754 | 0.009215 | 0.003504 | ESR1/HIF1A/PPARG | 3 |
| MF | GO:0030291 | protein serine/threonine kinase inhibitor activity | 2/32 | 36/18369 | 0.001785 | 0.009215 | 0.003504 | AKT1/CASP3 | 2 |
| MF | GO:0042805 | actinin binding | 2/32 | 36/18369 | 0.001785 | 0.009215 | 0.003504 | PPARG/RELA | 2 |
| MF | GO:0016247 | channel regulator activity | 3/32 | 152/18369 | 0.002311 | 0.011317 | 0.004303 | AKT1/BCL2/CAV1 | 3 |
| MF | GO:0016248 | channel inhibitor activity | 2/32 | 41/18369 | 0.002311 | 0.011317 | 0.004303 | BCL2/CAV1 | 2 |
| MF | GO:0004712 | protein serine/threonine/tyrosine kinase activity | 2/32 | 45/18369 | 0.002778 | 0.013192 | 0.005017 | AKT1/MAPK1 | 2 |
| MF | GO:0004879 | nuclear receptor activity | 2/32 | 46/18369 | 0.002901 | 0.013192 | 0.005017 | ESR1/PPARG | 2 |
| MF | GO:0098531 | ligand-activated transcription factor activity | 2/32 | 46/18369 | 0.002901 | 0.013192 | 0.005017 | ESR1/PPARG | 2 |
| MF | GO:0008009 | chemokine activity | 2/32 | 49/18369 | 0.003285 | 0.01451 | 0.005518 | CCL2/CXCL8 | 2 |
| MF | GO:0016504 | peptidase activator activity | 2/32 | 50/18369 | 0.003419 | 0.01451 | 0.005518 | APP/CAV1 | 2 |
| MF | GO:0016538 | cyclin-dependent protein serine/threonine kinase regulator activity | 2/32 | 50/18369 | 0.003419 | 0.01451 | 0.005518 | CASP3/CCND1 | 2 |
| MF | GO:0015459 | potassium channel regulator activity | 2/32 | 53/18369 | 0.003833 | 0.015915 | 0.006052 | AKT1/CAV1 | 2 |
| MF | GO:0004714 | transmembrane receptor protein tyrosine kinase activity | 2/32 | 60/18369 | 0.004886 | 0.01967 | 0.00748 | EGFR/ERBB2 | 2 |
| MF | GO:0001098 | basal transcription machinery binding | 2/32 | 61/18369 | 0.005046 | 0.01967 | 0.00748 | TP53/ESR1 | 2 |
| MF | GO:0001099 | basal RNA polymerase II transcription machinery binding | 2/32 | 61/18369 | 0.005046 | 0.01967 | 0.00748 | TP53/ESR1 | 2 |
| MF | GO:0005507 | copper ion binding | 2/32 | 62/18369 | 0.005209 | 0.019898 | 0.007567 | TP53/IL1A | 2 |
| MF | GO:0004674 | protein serine/threonine kinase activity | 4/32 | 421/18369 | 0.005887 | 0.022048 | 0.008384 | AKT1/EGFR/GSK3B/MAPK1 | 4 |
| MF | GO:0004175 | endopeptidase activity | 4/32 | 428/18369 | 0.006237 | 0.022908 | 0.008711 | CASP3/MMP9/MMP2/CASP9 | 4 |
| MF | GO:0043621 | protein self-association | 2/32 | 72/18369 | 0.006965 | 0.0251 | 0.009545 | TP53/PPARG | 2 |
| MF | GO:0042379 | chemokine receptor binding | 2/32 | 74/18369 | 0.007344 | 0.025536 | 0.009711 | CCL2/CXCL8 | 2 |
| MF | GO:0061134 | peptidase regulator activity | 3/32 | 230/18369 | 0.007353 | 0.025536 | 0.009711 | APP/SERPINE1/CAV1 | 3 |
| MF | GO:0001618 | virus receptor activity | 2/32 | 77/18369 | 0.00793 | 0.026115 | 0.009931 | EGFR/ICAM1 | 2 |
| MF | GO:0019199 | transmembrane receptor protein kinase activity | 2/32 | 77/18369 | 0.00793 | 0.026115 | 0.009931 | EGFR/ERBB2 | 2 |
| MF | GO:0046332 | SMAD binding | 2/32 | 77/18369 | 0.00793 | 0.026115 | 0.009931 | FOS/PPARG | 2 |
| MF | GO:0140272 | exogenous protein binding | 2/32 | 78/18369 | 0.00813 | 0.026319 | 0.010008 | EGFR/ICAM1 | 2 |
| MF | GO:0008013 | beta-catenin binding | 2/32 | 88/18369 | 0.010252 | 0.032636 | 0.01241 | ESR1/GSK3B | 2 |
| MF | GO:0004867 | serine-type endopeptidase inhibitor activity | 2/32 | 92/18369 | 0.011163 | 0.034952 | 0.013291 | APP/SERPINE1 | 2 |
| MF | GO:0005496 | steroid binding | 2/32 | 99/18369 | 0.012838 | 0.039551 | 0.01504 | ESR1/CAV1 | 2 |
| MF | GO:0001227 | DNA-binding transcription repressor activity, RNA polymerase II-specific | 3/32 | 303/18369 | 0.015482 | 0.046937 | 0.017849 | TP53/MYC/RELA | 3 |
| MF | GO:0001217 | DNA-binding transcription repressor activity | 3/32 | 308/18369 | 0.016169 | 0.048254 | 0.01835 | TP53/MYC/RELA | 3 |
| MF | GO:0001094 | TFIID-class transcription factor complex binding | 1/32 | 10/18369 | 0.017289 | 0.048562 | 0.018466 | TP53 | 1 |
| MF | GO:0004954 | prostanoid receptor activity | 1/32 | 10/18369 | 0.017289 | 0.048562 | 0.018466 | PPARG | 1 |
| MF | GO:0004955 | prostaglandin receptor activity | 1/32 | 10/18369 | 0.017289 | 0.048562 | 0.018466 | PPARG | 1 |
| MF | GO:0008353 | RNA polymerase II CTD heptapeptide repeat kinase activity | 1/32 | 10/18369 | 0.017289 | 0.048562 | 0.018466 | MAPK1 | 1 |
| MF | GO:0004197 | cysteine-type endopeptidase activity | 2/32 | 118/18369 | 0.017899 | 0.049546 | 0.018841 | CASP3/CASP9 | 2 |
| MF | GO:0070513 | death domain binding | 1/32 | 11/18369 | 0.019002 | 0.049859 | 0.01896 | BCL2 | 1 |
| MF | GO:0097371 | MDM2/MDM4 family protein binding | 1/32 | 11/18369 | 0.019002 | 0.049859 | 0.01896 | TP53 | 1 |
| MF | GO:0140666 | annealing activity | 1/32 | 11/18369 | 0.019002 | 0.049859 | 0.01896 | TP53 | 1 |
| MF | GO:0004222 | metalloendopeptidase activity | 2/32 | 122/18369 | 0.019056 | 0.049859 | 0.01896 | MMP9/MMP2 | 2 |
| MF | GO:0004861 | cyclin-dependent protein serine/threonine kinase inhibitor activity | 1/32 | 12/18369 | 0.020712 | 0.051376 | 0.019537 | CASP3 | 1 |
| MF | GO:0034452 | dynactin binding | 1/32 | 12/18369 | 0.020712 | 0.051376 | 0.019537 | GSK3B | 1 |
| MF | GO:0042301 | phosphate ion binding | 1/32 | 12/18369 | 0.020712 | 0.051376 | 0.019537 | RELA | 1 |
| MF | GO:0097677 | STAT family protein binding | 1/32 | 12/18369 | 0.020712 | 0.051376 | 0.019537 | PPARG | 1 |
| MF | GO:0019838 | growth factor binding | 2/32 | 132/18369 | 0.022083 | 0.05222 | 0.019858 | EGFR/ERBB2 | 2 |
| MF | GO:0004707 | MAP kinase activity | 1/32 | 13/18369 | 0.022419 | 0.05222 | 0.019858 | MAPK1 | 1 |
| MF | GO:0034236 | protein kinase A catalytic subunit binding | 1/32 | 13/18369 | 0.022419 | 0.05222 | 0.019858 | GSK3B | 1 |
| MF | GO:0050998 | nitric-oxide synthase binding | 1/32 | 13/18369 | 0.022419 | 0.05222 | 0.019858 | CAV1 | 1 |
| MF | GO:0140537 | transcription regulator activator activity | 1/32 | 13/18369 | 0.022419 | 0.05222 | 0.019858 | HIF1A | 1 |
| MF | GO:0030295 | protein kinase activator activity | 2/32 | 135/18369 | 0.023028 | 0.052991 | 0.020151 | EGFR/EGF | 2 |
| MF | GO:0004713 | protein tyrosine kinase activity | 2/32 | 137/18369 | 0.023667 | 0.05318 | 0.020223 | EGFR/ERBB2 | 2 |
| MF | GO:0020037 | heme binding | 2/32 | 137/18369 | 0.023667 | 0.05318 | 0.020223 | PTGS2/HMOX1 | 2 |
| MF | GO:0004953 | icosanoid receptor activity | 1/32 | 14/18369 | 0.024123 | 0.053576 | 0.020373 | PPARG | 1 |
| MF | GO:0106310 | protein serine kinase activity | 3/32 | 363/18369 | 0.024859 | 0.054188 | 0.020606 | AKT1/GSK3B/MAPK1 | 3 |
| MF | GO:0019209 | kinase activator activity | 2/32 | 141/18369 | 0.024966 | 0.054188 | 0.020606 | EGFR/EGF | 2 |
| MF | GO:0046965 | nuclear retinoid X receptor binding | 1/32 | 15/18369 | 0.025824 | 0.054805 | 0.020841 | PPARG | 1 |
| MF | GO:0099103 | channel activator activity | 1/32 | 15/18369 | 0.025824 | 0.054805 | 0.020841 | AKT1 | 1 |
| MF | GO:0046906 | tetrapyrrole binding | 2/32 | 147/18369 | 0.02697 | 0.055992 | 0.021292 | PTGS2/HMOX1 | 2 |
| MF | GO:0099106 | ion channel regulator activity | 2/32 | 147/18369 | 0.02697 | 0.055992 | 0.021292 | AKT1/CAV1 | 2 |
| MF | GO:0005178 | integrin binding | 2/32 | 151/18369 | 0.028341 | 0.058205 | 0.022134 | IL1B/ICAM1 | 2 |
| MF | GO:0070064 | proline-rich region binding | 1/32 | 17/18369 | 0.029219 | 0.05937 | 0.022576 | CCND1 | 1 |
| MF | GO:0004708 | MAP kinase kinase activity | 1/32 | 18/18369 | 0.030911 | 0.0615 | 0.023387 | MAPK1 | 1 |
| MF | GO:0045236 | CXCR chemokine receptor binding | 1/32 | 18/18369 | 0.030911 | 0.0615 | 0.023387 | CXCL8 | 1 |
| MF | GO:0008083 | growth factor activity | 2/32 | 160/18369 | 0.031526 | 0.062077 | 0.023606 | EGF/IL10 | 2 |
| MF | GO:0036041 | long-chain fatty acid binding | 1/32 | 19/18369 | 0.032601 | 0.063539 | 0.024162 | PPARG | 1 |
| MF | GO:0005123 | death receptor binding | 1/32 | 20/18369 | 0.034288 | 0.065839 | 0.025037 | CASP3 | 1 |
| MF | GO:0008201 | heparin binding | 2/32 | 168/18369 | 0.034471 | 0.065839 | 0.025037 | CXCL8/APP | 2 |
| MF | GO:0004252 | serine-type endopeptidase activity | 2/32 | 170/18369 | 0.035223 | 0.066611 | 0.02533 | MMP9/MMP2 | 2 |
| MF | GO:0004866 | endopeptidase inhibitor activity | 2/32 | 173/18369 | 0.036364 | 0.068094 | 0.025894 | APP/SERPINE1 | 2 |
| MF | GO:0004709 | MAP kinase kinase kinase activity | 1/32 | 22/18369 | 0.037654 | 0.068956 | 0.026222 | EGFR | 1 |
| MF | GO:0050321 | tau-protein kinase activity | 1/32 | 22/18369 | 0.037654 | 0.068956 | 0.026222 | GSK3B | 1 |
| MF | GO:0016705 | oxidoreductase activity, acting on paired donors, with incorporation or reduction of molecular oxygen | 2/32 | 177/18369 | 0.037908 | 0.068956 | 0.026222 | PTGS2/HMOX1 | 2 |
| MF | GO:0030414 | peptidase inhibitor activity | 2/32 | 180/18369 | 0.039082 | 0.070037 | 0.026633 | APP/SERPINE1 | 2 |
| MF | GO:0035035 | histone acetyltransferase binding | 1/32 | 23/18369 | 0.039332 | 0.070037 | 0.026633 | TP53 | 1 |
| MF | GO:0019887 | protein kinase regulator activity | 2/32 | 184/18369 | 0.040668 | 0.070037 | 0.026633 | EGFR/EGF | 2 |
| MF | GO:0003707 | nuclear steroid receptor activity | 1/32 | 24/18369 | 0.041008 | 0.070037 | 0.026633 | ESR1 | 1 |
| MF | GO:0016702 | oxidoreductase activity, acting on single donors with incorporation of molecular oxygen, incorporation of two atoms of oxygen | 1/32 | 24/18369 | 0.041008 | 0.070037 | 0.026633 | PTGS2 | 1 |
| MF | GO:0042974 | nuclear retinoic acid receptor binding | 1/32 | 24/18369 | 0.041008 | 0.070037 | 0.026633 | PPARG | 1 |
| MF | GO:0008234 | cysteine-type peptidase activity | 2/32 | 185/18369 | 0.041069 | 0.070037 | 0.026633 | CASP3/CASP9 | 2 |
| MF | GO:0061135 | endopeptidase regulator activity | 2/32 | 187/18369 | 0.041874 | 0.070779 | 0.026915 | APP/SERPINE1 | 2 |
| MF | GO:0016701 | oxidoreductase activity, acting on single donors with incorporation of molecular oxygen | 1/32 | 25/18369 | 0.042681 | 0.070887 | 0.026956 | PTGS2 | 1 |
| MF | GO:0017025 | TBP-class protein binding | 1/32 | 25/18369 | 0.042681 | 0.070887 | 0.026956 | ESR1 | 1 |
| MF | GO:0008236 | serine-type peptidase activity | 2/32 | 190/18369 | 0.043094 | 0.070956 | 0.026983 | MMP9/MMP2 | 2 |
| MF | GO:0004190 | aspartic-type endopeptidase activity | 1/32 | 26/18369 | 0.044351 | 0.072401 | 0.027532 | CASP3 | 1 |
| MF | GO:0017171 | serine hydrolase activity | 2/32 | 194/18369 | 0.044741 | 0.072419 | 0.027539 | MMP9/MMP2 | 2 |
| MF | GO:0008237 | metallopeptidase activity | 2/32 | 197/18369 | 0.045991 | 0.073245 | 0.027853 | MMP9/MMP2 | 2 |
| MF | GO:0070001 | aspartic-type peptidase activity | 1/32 | 27/18369 | 0.046018 | 0.073245 | 0.027853 | CASP3 | 1 |
| MF | GO:0051393 | alpha-actinin binding | 1/32 | 28/18369 | 0.047682 | 0.075267 | 0.028622 | PPARG | 1 |
| MF | GO:0043325 | phosphatidylinositol-3,4-bisphosphate binding | 1/32 | 29/18369 | 0.049344 | 0.077251 | 0.029376 | AKT1 | 1 |

**Supplementary Table S5** – KEGG analysis

| ID | Description | GeneRatio | BgRatio | pvalue | p.adjust | qvalue | geneID | Count |
| --- | --- | --- | --- | --- | --- | --- | --- | --- |
| hsa05418 | Fluid shear stress and atherosclerosis | 15/32 | 139/8753 | 2.15E-19 | 4.14E-17 | 9.71E-18 | AKT1/IL1B/TP53/MMP9/BCL2/FOS/CCL2/IFNG/IL1A/ICAM1/RELA/MMP2/HMOX1/NFE2L2/CAV1 | 15 |
| hsa05417 | Lipid and atherosclerosis | 16/32 | 215/8753 | 4.27E-18 | 4.12E-16 | 9.66E-17 | AKT1/IL1B/TP53/CASP3/MMP9/BCL2/FOS/PPARG/GSK3B/CCL2/CXCL8/ICAM1/RELA/NFE2L2/CASP9/MAPK1 | 16 |
| hsa04933 | AGE-RAGE signaling pathway in diabetic complications | 13/32 | 100/8753 | 7.35E-18 | 4.73E-16 | 1.11E-16 | AKT1/IL1B/CASP3/BCL2/CCND1/CCL2/CXCL8/IL1A/ICAM1/RELA/MMP2/MAPK1/SERPINE1 | 13 |
| hsa05210 | Colorectal cancer | 12/32 | 86/8753 | 7.04E-17 | 3.40E-15 | 7.97E-16 | AKT1/TP53/CASP3/EGFR/BCL2/FOS/MYC/GSK3B/CCND1/EGF/CASP9/MAPK1 | 12 |
| hsa05219 | Bladder cancer | 10/32 | 41/8753 | 9.31E-17 | 3.59E-15 | 8.43E-16 | TP53/MMP9/EGFR/MYC/CCND1/EGF/ERBB2/CXCL8/MMP2/MAPK1 | 10 |
| hsa05215 | Prostate cancer | 12/32 | 97/8753 | 3.21E-16 | 1.03E-14 | 2.42E-15 | AKT1/TP53/MMP9/EGFR/BCL2/GSK3B/CCND1/EGF/ERBB2/RELA/CASP9/MAPK1 | 12 |
| hsa05167 | Kaposi sarcoma-associated herpesvirus infection | 14/32 | 194/8753 | 1.43E-15 | 3.95E-14 | 9.27E-15 | AKT1/TP53/PTGS2/CASP3/HIF1A/FOS/MYC/GSK3B/CCND1/CXCL8/ICAM1/RELA/CASP9/MAPK1 | 14 |
| hsa05213 | Endometrial cancer | 10/32 | 58/8753 | 4.17E-15 | 1.01E-13 | 2.36E-14 | AKT1/TP53/EGFR/MYC/GSK3B/CCND1/EGF/ERBB2/CASP9/MAPK1 | 10 |
| hsa05163 | Human cytomegalovirus infection | 14/32 | 225/8753 | 1.15E-14 | 2.47E-13 | 5.79E-14 | AKT1/IL1B/TP53/PTGS2/CASP3/EGFR/MYC/GSK3B/CCND1/CCL2/CXCL8/RELA/CASP9/MAPK1 | 14 |
| hsa04657 | IL-17 signaling pathway | 11/32 | 94/8753 | 1.29E-14 | 2.49E-13 | 5.83E-14 | IL1B/PTGS2/CASP3/MMP9/FOS/GSK3B/CCL2/IFNG/CXCL8/RELA/MAPK1 | 11 |
| hsa01522 | Endocrine resistance | 11/32 | 98/8753 | 2.07E-14 | 3.64E-13 | 8.53E-14 | AKT1/TP53/ESR1/MMP9/EGFR/BCL2/FOS/CCND1/ERBB2/MMP2/MAPK1 | 11 |
| hsa04066 | HIF-1 signaling pathway | 11/32 | 108/8753 | 6.24E-14 | 1.00E-12 | 2.35E-13 | AKT1/EGFR/BCL2/HIF1A/EGF/ERBB2/IFNG/RELA/HMOX1/MAPK1/SERPINE1 | 11 |
| hsa05205 | Proteoglycans in cancer | 13/32 | 203/8753 | 9.02E-14 | 1.34E-12 | 3.14E-13 | AKT1/TP53/ESR1/CASP3/MMP9/EGFR/HIF1A/MYC/CCND1/ERBB2/MMP2/MAPK1/CAV1 | 13 |
| hsa05160 | Hepatitis C | 12/32 | 158/8753 | 1.30E-13 | 1.79E-12 | 4.20E-13 | AKT1/TP53/CASP3/EGFR/MYC/GSK3B/CCND1/EGF/IFNG/RELA/CASP9/MAPK1 | 12 |
| hsa05162 | Measles | 11/32 | 138/8753 | 9.74E-13 | 1.25E-11 | 2.94E-12 | AKT1/IL1B/TP53/CASP3/BCL2/FOS/GSK3B/CCND1/IL1A/RELA/CASP9 | 11 |
| hsa05142 | Chagas disease | 10/32 | 102/8753 | 1.54E-12 | 1.85E-11 | 4.35E-12 | AKT1/IL1B/FOS/IL10/CCL2/IFNG/CXCL8/RELA/MAPK1/SERPINE1 | 10 |
| hsa05224 | Breast cancer | 11/32 | 147/8753 | 1.96E-12 | 2.23E-11 | 5.22E-12 | AKT1/TP53/ESR1/EGFR/FOS/MYC/GSK3B/CCND1/EGF/ERBB2/MAPK1 | 11 |
| hsa05212 | Pancreatic cancer | 9/32 | 76/8753 | 4.12E-12 | 4.41E-11 | 1.04E-11 | AKT1/TP53/EGFR/CCND1/EGF/ERBB2/RELA/CASP9/MAPK1 | 9 |
| hsa04668 | TNF signaling pathway | 10/32 | 114/8753 | 4.78E-12 | 4.85E-11 | 1.14E-11 | AKT1/IL1B/PTGS2/CASP3/MMP9/FOS/CCL2/ICAM1/RELA/MAPK1 | 10 |
| hsa05161 | Hepatitis B | 11/32 | 162/8753 | 5.73E-12 | 5.53E-11 | 1.30E-11 | AKT1/TP53/CASP3/MMP9/BCL2/FOS/MYC/CXCL8/RELA/CASP9/MAPK1 | 11 |
| hsa05164 | Influenza A | 11/32 | 171/8753 | 1.04E-11 | 9.53E-11 | 2.23E-11 | AKT1/IL1B/CASP3/CCL2/IFNG/CXCL8/IL1A/ICAM1/RELA/CASP9/MAPK1 | 11 |
| hsa05222 | Small cell lung cancer | 9/32 | 92/8753 | 2.42E-11 | 2.12E-10 | 4.97E-11 | AKT1/TP53/PTGS2/CASP3/BCL2/MYC/CCND1/RELA/CASP9 | 9 |
| hsa05226 | Gastric cancer | 10/32 | 149/8753 | 7.06E-11 | 5.93E-10 | 1.39E-10 | AKT1/TP53/EGFR/BCL2/MYC/GSK3B/CCND1/EGF/ERBB2/MAPK1 | 10 |
| hsa05223 | Non-small cell lung cancer | 8/32 | 72/8753 | 1.26E-10 | 1.02E-09 | 2.38E-10 | AKT1/TP53/EGFR/CCND1/EGF/ERBB2/CASP9/MAPK1 | 8 |
| hsa05133 | Pertussis | 8/32 | 76/8753 | 1.97E-10 | 1.52E-09 | 3.57E-10 | IL1B/CASP3/FOS/IL10/CXCL8/IL1A/RELA/MAPK1 | 8 |
| hsa05140 | Leishmaniasis | 8/32 | 77/8753 | 2.19E-10 | 1.63E-09 | 3.82E-10 | IL1B/PTGS2/FOS/IL10/IFNG/IL1A/RELA/MAPK1 | 8 |
| hsa04010 | MAPK signaling pathway | 12/32 | 299/8753 | 2.48E-10 | 1.77E-09 | 4.16E-10 | AKT1/IL1B/TP53/CASP3/EGFR/FOS/MYC/EGF/ERBB2/IL1A/RELA/MAPK1 | 12 |
| hsa04919 | Thyroid hormone signaling pathway | 9/32 | 121/8753 | 2.93E-10 | 2.02E-09 | 4.74E-10 | AKT1/TP53/ESR1/HIF1A/MYC/GSK3B/CCND1/CASP9/MAPK1 | 9 |
| hsa05152 | Tuberculosis | 10/32 | 180/8753 | 4.59E-10 | 3.06E-09 | 7.17E-10 | AKT1/IL1B/CASP3/BCL2/IL10/IFNG/IL1A/RELA/CASP9/MAPK1 | 10 |
| hsa05235 | PD-L1 expression and PD-1 checkpoint pathway in cancer | 8/32 | 89/8753 | 7.15E-10 | 4.60E-09 | 1.08E-09 | AKT1/EGFR/HIF1A/FOS/EGF/IFNG/RELA/MAPK1 | 8 |
| hsa05135 | Yersinia infection | 9/32 | 137/8753 | 8.95E-10 | 5.57E-09 | 1.31E-09 | AKT1/IL1B/FOS/GSK3B/IL10/CCL2/CXCL8/RELA/MAPK1 | 9 |
| hsa04151 | PI3K-Akt signaling pathway | 12/32 | 359/8753 | 2.03E-09 | 1.22E-08 | 2.87E-09 | AKT1/TP53/EGFR/BCL2/MYC/GSK3B/CCND1/EGF/ERBB2/RELA/CASP9/MAPK1 | 12 |
| hsa05207 | Chemical carcinogenesis - receptor activation | 10/32 | 212/8753 | 2.28E-09 | 1.33E-08 | 3.12E-09 | AKT1/ESR1/EGFR/BCL2/FOS/MYC/CCND1/EGF/RELA/MAPK1 | 10 |
| hsa04932 | Non-alcoholic fatty liver disease | 9/32 | 155/8753 | 2.69E-09 | 1.53E-08 | 3.58E-09 | AKT1/IL1B/CASP3/FOS/PPARG/GSK3B/CXCL8/IL1A/RELA | 9 |
| hsa04218 | Cellular senescence | 9/32 | 156/8753 | 2.85E-09 | 1.57E-08 | 3.68E-09 | AKT1/TP53/MYC/CCND1/CXCL8/IL1A/RELA/MAPK1/SERPINE1 | 9 |
| hsa05145 | Toxoplasmosis | 8/32 | 111/8753 | 4.24E-09 | 2.25E-08 | 5.27E-09 | AKT1/CASP3/BCL2/IL10/IFNG/RELA/CASP9/MAPK1 | 8 |
| hsa04917 | Prolactin signaling pathway | 7/32 | 70/8753 | 4.42E-09 | 2.25E-08 | 5.27E-09 | AKT1/ESR1/FOS/GSK3B/CCND1/RELA/MAPK1 | 7 |
| hsa05230 | Central carbon metabolism in cancer | 7/32 | 70/8753 | 4.42E-09 | 2.25E-08 | 5.27E-09 | AKT1/TP53/EGFR/HIF1A/MYC/ERBB2/MAPK1 | 7 |
| hsa05225 | Hepatocellular carcinoma | 9/32 | 168/8753 | 5.48E-09 | 2.71E-08 | 6.36E-09 | AKT1/TP53/EGFR/MYC/GSK3B/CCND1/HMOX1/NFE2L2/MAPK1 | 9 |
| hsa01524 | Platinum drug resistance | 7/32 | 73/8753 | 5.96E-09 | 2.88E-08 | 6.75E-09 | AKT1/TP53/CASP3/BCL2/ERBB2/CASP9/MAPK1 | 7 |
| hsa05206 | MicroRNAs in cancer | 11/32 | 310/8753 | 6.12E-09 | 2.88E-08 | 6.75E-09 | TP53/PTGS2/CASP3/MMP9/EGFR/BCL2/MYC/CCND1/ERBB2/HMOX1/MAPK1 | 11 |
| hsa01521 | EGFR tyrosine kinase inhibitor resistance | 7/32 | 79/8753 | 1.05E-08 | 4.80E-08 | 1.13E-08 | AKT1/EGFR/BCL2/GSK3B/EGF/ERBB2/MAPK1 | 7 |
| hsa04012 | ErbB signaling pathway | 7/32 | 85/8753 | 1.75E-08 | 7.87E-08 | 1.85E-08 | AKT1/EGFR/MYC/GSK3B/EGF/ERBB2/MAPK1 | 7 |
| hsa04210 | Apoptosis | 8/32 | 135/8753 | 2.00E-08 | 8.78E-08 | 2.06E-08 | AKT1/TP53/CASP3/BCL2/FOS/RELA/CASP9/MAPK1 | 8 |
| hsa05144 | Malaria | 6/32 | 50/8753 | 2.06E-08 | 8.85E-08 | 2.08E-08 | IL1B/IL10/CCL2/IFNG/CXCL8/ICAM1 | 6 |
| hsa04915 | Estrogen signaling pathway | 8/32 | 138/8753 | 2.38E-08 | 9.99E-08 | 2.34E-08 | AKT1/ESR1/MMP9/EGFR/BCL2/FOS/MMP2/MAPK1 | 8 |
| hsa04510 | Focal adhesion | 9/32 | 202/8753 | 2.75E-08 | 1.11E-07 | 2.60E-08 | AKT1/EGFR/BCL2/GSK3B/CCND1/EGF/ERBB2/MAPK1/CAV1 | 9 |
| hsa05169 | Epstein-Barr virus infection | 9/32 | 202/8753 | 2.75E-08 | 1.11E-07 | 2.60E-08 | AKT1/TP53/CASP3/BCL2/MYC/CCND1/ICAM1/RELA/CASP9 | 9 |
| hsa04380 | Osteoclast differentiation | 8/32 | 141/8753 | 2.82E-08 | 1.11E-07 | 2.61E-08 | AKT1/IL1B/FOS/PPARG/IFNG/IL1A/RELA/MAPK1 | 8 |
| hsa05323 | Rheumatoid arthritis | 7/32 | 93/8753 | 3.30E-08 | 1.27E-07 | 2.99E-08 | IL1B/FOS/CCL2/IFNG/CXCL8/IL1A/ICAM1 | 7 |
| hsa05208 | Chemical carcinogenesis - reactive oxygen species | 9/32 | 223/8753 | 6.48E-08 | 2.45E-07 | 5.75E-08 | AKT1/EGFR/HIF1A/FOS/EGF/RELA/HMOX1/NFE2L2/MAPK1 | 9 |
| hsa04630 | JAK-STAT signaling pathway | 8/32 | 166/8753 | 1.01E-07 | 3.75E-07 | 8.79E-08 | AKT1/EGFR/BCL2/MYC/CCND1/EGF/IL10/IFNG | 8 |
| hsa05131 | Shigellosis | 9/32 | 247/8753 | 1.56E-07 | 5.58E-07 | 1.31E-07 | AKT1/IL1B/TP53/EGFR/BCL2/GSK3B/CXCL8/RELA/MAPK1 | 9 |
| hsa05132 | Salmonella infection | 9/32 | 247/8753 | 1.56E-07 | 5.58E-07 | 1.31E-07 | AKT1/IL1B/CASP3/BCL2/FOS/MYC/CXCL8/RELA/MAPK1 | 9 |
| hsa05165 | Human papillomavirus infection | 10/32 | 331/8753 | 1.60E-07 | 5.63E-07 | 1.32E-07 | AKT1/TP53/PTGS2/CASP3/EGFR/GSK3B/CCND1/EGF/RELA/MAPK1 | 10 |
| hsa05216 | Thyroid cancer | 5/32 | 37/8753 | 1.89E-07 | 6.50E-07 | 1.52E-07 | TP53/MYC/PPARG/CCND1/MAPK1 | 5 |
| hsa05218 | Melanoma | 6/32 | 72/8753 | 1.92E-07 | 6.50E-07 | 1.52E-07 | AKT1/TP53/EGFR/CCND1/EGF/MAPK1 | 6 |
| hsa04660 | T cell receptor signaling pathway | 7/32 | 121/8753 | 2.05E-07 | 6.82E-07 | 1.60E-07 | AKT1/FOS/GSK3B/IL10/IFNG/RELA/MAPK1 | 7 |
| hsa04115 | p53 signaling pathway | 6/32 | 75/8753 | 2.45E-07 | 7.89E-07 | 1.85E-07 | TP53/CASP3/BCL2/CCND1/CASP9/SERPINE1 | 6 |
| hsa05214 | Glioma | 6/32 | 75/8753 | 2.45E-07 | 7.89E-07 | 1.85E-07 | AKT1/TP53/EGFR/CCND1/EGF/MAPK1 | 6 |
| hsa05220 | Chronic myeloid leukemia | 6/32 | 76/8753 | 2.66E-07 | 8.41E-07 | 1.97E-07 | AKT1/TP53/MYC/CCND1/RELA/MAPK1 | 6 |
| hsa04926 | Relaxin signaling pathway | 7/32 | 129/8753 | 3.18E-07 | 9.89E-07 | 2.32E-07 | AKT1/MMP9/EGFR/FOS/RELA/MMP2/MAPK1 | 7 |
| hsa04936 | Alcoholic liver disease | 7/32 | 142/8753 | 6.12E-07 | 1.87E-06 | 4.39E-07 | AKT1/IL1B/CASP3/GSK3B/CCND1/CXCL8/RELA | 7 |
| hsa05010 | Alzheimer disease | 10/32 | 384/8753 | 6.37E-07 | 1.92E-06 | 4.51E-07 | AKT1/IL1B/PTGS2/CASP3/GSK3B/IL1A/RELA/APP/CASP9/MAPK1 | 10 |
| hsa05166 | Human T-cell leukemia virus 1 infection | 8/32 | 222/8753 | 9.39E-07 | 2.79E-06 | 6.54E-07 | AKT1/TP53/FOS/MYC/CCND1/ICAM1/RELA/MAPK1 | 8 |
| hsa05231 | Choline metabolism in cancer | 6/32 | 98/8753 | 1.21E-06 | 3.54E-06 | 8.29E-07 | AKT1/EGFR/HIF1A/FOS/EGF/MAPK1 | 6 |
| hsa05146 | Amoebiasis | 6/32 | 102/8753 | 1.53E-06 | 4.41E-06 | 1.03E-06 | IL1B/CASP3/IL10/IFNG/CXCL8/RELA | 6 |
| hsa05134 | Legionellosis | 5/32 | 56/8753 | 1.58E-06 | 4.48E-06 | 1.05E-06 | IL1B/CASP3/CXCL8/RELA/CASP9 | 5 |
| hsa04625 | C-type lectin receptor signaling pathway | 6/32 | 104/8753 | 1.72E-06 | 4.80E-06 | 1.13E-06 | AKT1/IL1B/PTGS2/IL10/RELA/MAPK1 | 6 |
| hsa04064 | NF-kappa B signaling pathway | 6/32 | 105/8753 | 1.82E-06 | 5.01E-06 | 1.17E-06 | IL1B/PTGS2/BCL2/CXCL8/ICAM1/RELA | 6 |
| hsa04620 | Toll-like receptor signaling pathway | 6/32 | 108/8753 | 2.14E-06 | 5.74E-06 | 1.35E-06 | AKT1/IL1B/FOS/CXCL8/RELA/MAPK1 | 6 |
| hsa04659 | Th17 cell differentiation | 6/32 | 108/8753 | 2.14E-06 | 5.74E-06 | 1.35E-06 | IL1B/HIF1A/FOS/IFNG/RELA/MAPK1 | 6 |
| hsa05321 | Inflammatory bowel disease | 5/32 | 65/8753 | 3.33E-06 | 8.81E-06 | 2.07E-06 | IL1B/IL10/IFNG/IL1A/RELA | 5 |
| hsa04722 | Neurotrophin signaling pathway | 6/32 | 119/8753 | 3.78E-06 | 9.85E-06 | 2.31E-06 | AKT1/TP53/BCL2/GSK3B/RELA/MAPK1 | 6 |
| hsa05221 | Acute myeloid leukemia | 5/32 | 67/8753 | 3.88E-06 | 9.97E-06 | 2.34E-06 | AKT1/MYC/CCND1/RELA/MAPK1 | 5 |
| hsa05416 | Viral myocarditis | 5/32 | 69/8753 | 4.49E-06 | 1.13E-05 | 2.64E-06 | CASP3/CCND1/ICAM1/CASP9/CAV1 | 5 |
| hsa05022 | Pathways of neurodegeneration - multiple diseases | 10/32 | 476/8753 | 4.49E-06 | 1.13E-05 | 2.64E-06 | IL1B/PTGS2/CASP3/BCL2/GSK3B/IL1A/RELA/APP/CASP9/MAPK1 | 10 |
| hsa05130 | Pathogenic Escherichia coli infection | 7/32 | 198/8753 | 5.68E-06 | 1.40E-05 | 3.29E-06 | IL1B/CASP3/FOS/CXCL8/RELA/CASP9/MAPK1 | 7 |
| hsa04068 | FoxO signaling pathway | 6/32 | 131/8753 | 6.60E-06 | 1.61E-05 | 3.78E-06 | AKT1/EGFR/CCND1/EGF/IL10/MAPK1 | 6 |
| hsa05170 | Human immunodeficiency virus 1 infection | 7/32 | 212/8753 | 8.90E-06 | 2.13E-05 | 4.99E-06 | AKT1/CASP3/BCL2/FOS/RELA/CASP9/MAPK1 | 7 |
| hsa05143 | African trypanosomiasis | 4/32 | 37/8753 | 8.93E-06 | 2.13E-05 | 4.99E-06 | IL1B/IL10/IFNG/ICAM1 | 4 |
| hsa05171 | Coronavirus disease - COVID-19 | 7/32 | 233/8753 | 1.65E-05 | 3.87E-05 | 9.06E-06 | IL1B/EGFR/FOS/CCL2/CXCL8/RELA/MAPK1 | 7 |
| hsa04662 | B cell receptor signaling pathway | 5/32 | 90/8753 | 1.66E-05 | 3.87E-05 | 9.06E-06 | AKT1/FOS/GSK3B/RELA/MAPK1 | 5 |
| hsa04148 | Efferocytosis | 6/32 | 156/8753 | 1.80E-05 | 4.13E-05 | 9.69E-06 | PTGS2/CASP3/HIF1A/PPARG/IL10/MAPK1 | 6 |
| hsa05020 | Prion disease | 7/32 | 272/8753 | 4.47E-05 | 0.000102 | 2.38E-05 | IL1B/CASP3/GSK3B/IL1A/CASP9/MAPK1/CAV1 | 7 |
| hsa04621 | NOD-like receptor signaling pathway | 6/32 | 186/8753 | 4.86E-05 | 0.000109 | 2.56E-05 | IL1B/BCL2/CCL2/CXCL8/RELA/MAPK1 | 6 |
| hsa04062 | Chemokine signaling pathway | 6/32 | 192/8753 | 5.80E-05 | 0.000128 | 2.99E-05 | AKT1/GSK3B/CCL2/CXCL8/RELA/MAPK1 | 6 |
| hsa04370 | VEGF signaling pathway | 4/32 | 59/8753 | 5.82E-05 | 0.000128 | 2.99E-05 | AKT1/PTGS2/CASP9/MAPK1 | 4 |
| hsa05202 | Transcriptional misregulation in cancer | 6/32 | 193/8753 | 5.97E-05 | 0.00013 | 3.04E-05 | TP53/MMP9/MYC/PPARG/CXCL8/RELA | 6 |
| hsa04071 | Sphingolipid signaling pathway | 5/32 | 121/8753 | 6.94E-05 | 0.000149 | 3.49E-05 | AKT1/TP53/BCL2/RELA/MAPK1 | 5 |
| hsa05120 | Epithelial cell signaling in Helicobacter pylori infection | 4/32 | 70/8753 | 0.000114 | 0.000242 | 5.67E-05 | CASP3/EGFR/CXCL8/RELA | 4 |
| hsa04072 | Phospholipase D signaling pathway | 5/32 | 148/8753 | 0.00018 | 0.000378 | 8.85E-05 | AKT1/EGFR/EGF/CXCL8/MAPK1 | 5 |
| hsa04215 | Apoptosis - multiple species | 3/32 | 32/8753 | 0.000205 | 0.000425 | 9.97E-05 | CASP3/BCL2/CASP9 | 3 |
| hsa04921 | Oxytocin signaling pathway | 5/32 | 154/8753 | 0.000217 | 0.000445 | 0.000104 | PTGS2/EGFR/FOS/CCND1/MAPK1 | 5 |
| hsa04211 | Longevity regulating pathway | 4/32 | 89/8753 | 0.000289 | 0.000587 | 0.000138 | AKT1/TP53/PPARG/RELA | 4 |
| hsa04658 | Th1 and Th2 cell differentiation | 4/32 | 92/8753 | 0.000328 | 0.00066 | 0.000155 | FOS/IFNG/RELA/MAPK1 | 4 |
| hsa05332 | Graft-versus-host disease | 3/32 | 42/8753 | 0.000463 | 0.00092 | 0.000216 | IL1B/IFNG/IL1A | 3 |
| hsa04940 | Type I diabetes mellitus | 3/32 | 43/8753 | 0.000496 | 0.000977 | 0.000229 | IL1B/IFNG/IL1A | 3 |
| hsa04060 | Cytokine-cytokine receptor interaction | 6/32 | 297/8753 | 0.000624 | 0.001216 | 0.000285 | IL1B/IL10/CCL2/IFNG/CXCL8/IL1A | 6 |
| hsa04725 | Cholinergic synapse | 4/32 | 113/8753 | 0.000717 | 0.001383 | 0.000324 | AKT1/BCL2/FOS/MAPK1 | 4 |
| hsa04928 | Parathyroid hormone synthesis, secretion and action | 4/32 | 114/8753 | 0.000741 | 0.001415 | 0.000332 | EGFR/BCL2/FOS/MAPK1 | 4 |
| hsa04726 | Serotonergic synapse | 4/32 | 115/8753 | 0.000765 | 0.001447 | 0.000339 | PTGS2/CASP3/APP/MAPK1 | 4 |
| hsa05415 | Diabetic cardiomyopathy | 5/32 | 203/8753 | 0.000772 | 0.001447 | 0.000339 | AKT1/MMP9/GSK3B/RELA/MMP2 | 5 |
| hsa05203 | Viral carcinogenesis | 5/32 | 204/8753 | 0.00079 | 0.001465 | 0.000344 | TP53/CASP3/CCND1/RELA/MAPK1 | 5 |
| hsa04935 | Growth hormone synthesis, secretion and action | 4/32 | 120/8753 | 0.000898 | 0.001651 | 0.000387 | AKT1/FOS/GSK3B/MAPK1 | 4 |
| hsa04340 | Hedgehog signaling pathway | 3/32 | 56/8753 | 0.001079 | 0.001964 | 0.000461 | BCL2/GSK3B/CCND1 | 3 |
| hsa04650 | Natural killer cell mediated cytotoxicity | 4/32 | 132/8753 | 0.001281 | 0.00231 | 0.000542 | CASP3/IFNG/ICAM1/MAPK1 | 4 |
| hsa04014 | Ras signaling pathway | 5/32 | 236/8753 | 0.001516 | 0.00271 | 0.000636 | AKT1/EGFR/EGF/RELA/MAPK1 | 5 |
| hsa04371 | Apelin signaling pathway | 4/32 | 139/8753 | 0.001551 | 0.002746 | 0.000644 | AKT1/CCND1/MAPK1/SERPINE1 | 4 |
| hsa04550 | Signaling pathways regulating pluripotency of stem cells | 4/32 | 143/8753 | 0.001721 | 0.00302 | 0.000708 | AKT1/MYC/GSK3B/MAPK1 | 4 |
| hsa05211 | Renal cell carcinoma | 3/32 | 69/8753 | 0.001974 | 0.003433 | 0.000805 | AKT1/HIF1A/MAPK1 | 3 |
| hsa04934 | Cushing syndrome | 4/32 | 155/8753 | 0.002312 | 0.003984 | 0.000934 | EGFR/GSK3B/CCND1/MAPK1 | 4 |
| hsa04110 | Cell cycle | 4/32 | 157/8753 | 0.002422 | 0.004101 | 0.000962 | TP53/MYC/GSK3B/CCND1 | 4 |
| hsa04390 | Hippo signaling pathway | 4/32 | 157/8753 | 0.002422 | 0.004101 | 0.000962 | MYC/GSK3B/CCND1/SERPINE1 | 4 |
| hsa04217 | Necroptosis | 4/32 | 159/8753 | 0.002536 | 0.004257 | 0.000998 | IL1B/BCL2/IFNG/IL1A | 4 |
| hsa04140 | Autophagy - animal | 4/32 | 165/8753 | 0.002901 | 0.004826 | 0.001132 | AKT1/BCL2/HIF1A/MAPK1 | 4 |
| hsa04623 | Cytosolic DNA-sensing pathway | 3/32 | 83/8753 | 0.003345 | 0.005517 | 0.001294 | IL1B/CASP3/RELA | 3 |
| hsa04310 | Wnt signaling pathway | 4/32 | 174/8753 | 0.003512 | 0.005745 | 0.001347 | TP53/MYC/GSK3B/CCND1 | 4 |
| hsa04540 | Gap junction | 3/32 | 88/8753 | 0.003945 | 0.006399 | 0.001501 | EGFR/EGF/MAPK1 | 3 |
| hsa04520 | Adherens junction | 3/32 | 93/8753 | 0.004608 | 0.00735 | 0.001724 | EGFR/ERBB2/MAPK1 | 3 |
| hsa04912 | GnRH signaling pathway | 3/32 | 93/8753 | 0.004608 | 0.00735 | 0.001724 | EGFR/MMP2/MAPK1 | 3 |
| hsa01523 | Antifolate resistance | 2/32 | 30/8753 | 0.005284 | 0.00836 | 0.001961 | IL1B/RELA | 2 |
| hsa04061 | Viral protein interaction with cytokine and cytokine receptor | 3/32 | 100/8753 | 0.005644 | 0.008856 | 0.002077 | IL10/CCL2/CXCL8 | 3 |
| hsa04137 | Mitophagy - animal | 3/32 | 103/8753 | 0.006127 | 0.009537 | 0.002237 | TP53/HIF1A/RELA | 3 |
| hsa04015 | Rap1 signaling pathway | 4/32 | 210/8753 | 0.006839 | 0.010559 | 0.002476 | AKT1/EGFR/EGF/MAPK1 | 4 |
| hsa04350 | TGF-beta signaling pathway | 3/32 | 108/8753 | 0.006986 | 0.010617 | 0.00249 | MYC/IFNG/MAPK1 | 3 |
| hsa04931 | Insulin resistance | 3/32 | 108/8753 | 0.006986 | 0.010617 | 0.00249 | AKT1/GSK3B/RELA | 3 |
| hsa04670 | Leukocyte transendothelial migration | 3/32 | 115/8753 | 0.008305 | 0.012522 | 0.002937 | MMP9/ICAM1/MMP2 | 3 |
| hsa05330 | Allograft rejection | 2/32 | 38/8753 | 0.008386 | 0.012547 | 0.002942 | IL10/IFNG | 2 |
| hsa04024 | cAMP signaling pathway | 4/32 | 225/8753 | 0.00869 | 0.012902 | 0.003026 | AKT1/FOS/RELA/MAPK1 | 4 |
| hsa04810 | Regulation of actin cytoskeleton | 4/32 | 229/8753 | 0.009235 | 0.013605 | 0.003191 | AKT1/EGFR/EGF/MAPK1 | 4 |
| hsa04152 | AMPK signaling pathway | 3/32 | 121/8753 | 0.009544 | 0.013955 | 0.003273 | AKT1/PPARG/CCND1 | 3 |
| hsa04216 | Ferroptosis | 2/32 | 41/8753 | 0.009715 | 0.014098 | 0.003306 | TP53/HMOX1 | 2 |
| hsa04728 | Dopaminergic synapse | 3/32 | 132/8753 | 0.012085 | 0.017406 | 0.004082 | AKT1/FOS/GSK3B | 3 |
| hsa04910 | Insulin signaling pathway | 3/32 | 137/8753 | 0.013357 | 0.019095 | 0.004478 | AKT1/GSK3B/MAPK1 | 3 |
| hsa05012 | Parkinson disease | 4/32 | 266/8753 | 0.015355 | 0.021791 | 0.00511 | TP53/CASP3/NFE2L2/CASP9 | 4 |
| hsa04261 | Adrenergic signaling in cardiomyocytes | 3/32 | 154/8753 | 0.01824 | 0.025695 | 0.006026 | AKT1/BCL2/MAPK1 | 3 |
| hsa04923 | Regulation of lipolysis in adipocytes | 2/32 | 58/8753 | 0.018845 | 0.026203 | 0.006145 | AKT1/PTGS2 | 2 |
| hsa04150 | mTOR signaling pathway | 3/32 | 156/8753 | 0.018871 | 0.026203 | 0.006145 | AKT1/GSK3B/MAPK1 | 3 |
| hsa05217 | Basal cell carcinoma | 2/32 | 63/8753 | 0.022014 | 0.030349 | 0.007117 | TP53/GSK3B | 2 |
| hsa04929 | GnRH secretion | 2/32 | 64/8753 | 0.022673 | 0.031035 | 0.007278 | AKT1/MAPK1 | 2 |
| hsa05016 | Huntington disease | 4/32 | 306/8753 | 0.024361 | 0.03311 | 0.007765 | TP53/CASP3/PPARG/CASP9 | 4 |
| hsa04664 | Fc epsilon RI signaling pathway | 2/32 | 68/8753 | 0.02539 | 0.034267 | 0.008036 | AKT1/MAPK1 | 2 |
| hsa04920 | Adipocytokine signaling pathway | 2/32 | 70/8753 | 0.026795 | 0.035913 | 0.008423 | AKT1/RELA | 2 |
| hsa04622 | RIG-I-like receptor signaling pathway | 2/32 | 72/8753 | 0.028232 | 0.037578 | 0.008813 | CXCL8/RELA | 2 |
| hsa04613 | Neutrophil extracellular trap formation | 3/32 | 191/8753 | 0.031901 | 0.042171 | 0.00989 | AKT1/RELA/MAPK1 | 3 |
| hsa05014 | Amyotrophic lateral sclerosis | 4/32 | 364/8753 | 0.04223 | 0.055445 | 0.013003 | TP53/CASP3/BCL2/CASP9 | 4 |
| hsa05150 | Staphylococcus aureus infection | 2/32 | 96/8753 | 0.047721 | 0.062231 | 0.014595 | IL10/ICAM1 | 2 |
| hsa04666 | Fc gamma R-mediated phagocytosis | 2/32 | 97/8753 | 0.048616 | 0.062553 | 0.01467 | AKT1/MAPK1 | 2 |
| hsa04713 | Circadian entrainment | 2/32 | 97/8753 | 0.048616 | 0.062553 | 0.01467 | FOS/MAPK1 | 2 |
